# Asymmetric Total Syntheses of Eliglustat and C2-*epi*-Eliglustat

**DOI:** 10.1021/acs.joc.5c03229

**Published:** 2026-02-16

**Authors:** Miguel Mellado-Hidalgo, Anna M. Costa, Pedro Romea, Fèlix Urpí, Gabriel Aullón

**Affiliations:** † Department of Inorganic and Organic Chemistry, Section of Organic Chemistry, and Institut de Biomedicina de la Universitat de Barcelona, 16724 Universitat de Barcelona, Carrer Martí i Franqués 1−11, 08028 Barcelona, Catalonia, Spain; ‡ Department of Inorganic and Organic Chemistry, Section of Inorganic Chemistry, and Institut de Química Teòrica i Computacional de la Universitat de Barcelona, Universitat de Barcelona, Carrer Martí i Franqués 1−11, 08028 Barcelona, Catalonia, Spain

## Abstract

Parallel asymmetric total syntheses of eliglustat and C2-*epi*-eliglustat are reported. Both approaches feature a common key step, which involves a stereocontrolled TMSOTf-mediated aldol-like reaction of azidoacetyl thioimides with dibenzyl acetals of aromatic aldehydes catalyzed by chiral nickel­(II) complexes. It is worth noting that the structure of dibenzyl acetals plays a key role in the stereochemical outcome of some of these reactions, as supported by theoretical calculations. Overall, this strategy enables the independent manipulation of the amino and hydroxy functional groups and favors the isolation of enantiomerically pure products.

## Graphical abstract

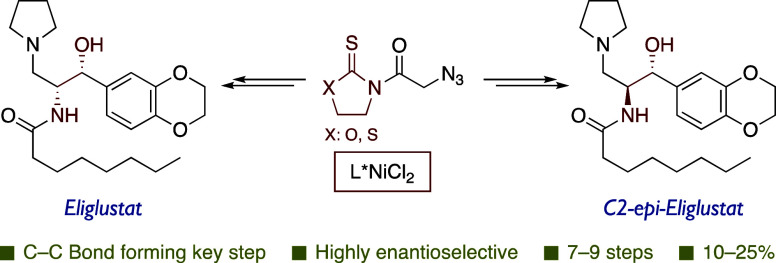

## Introduction

The structure of biologically active compounds determines their pharmacological, physicochemical, and toxicological properties.^1^ Notably, stereoisomers, whether enantiomers or diastereomers, often exhibit strikingly different interactions with biological targets and follow distinct metabolic pathways, despite sharing the same molecular formula and atomic connectivity.^2^ This selective recognition of stereoisomers by chiral biomolecules, such as enzymes and receptors, underscores the importance of accessing not only both enantiomers but also all relevant diastereomers of a compound to fully characterize its biological profile.^3–5^

Stereodivergent strategies,^6^ which enable the synthesis of multiple stereoisomers from common precursors, provide a powerful platform for the systematic investigation of bioactive targets. By integrating modular synthetic routes that incorporate switchable stereocontrol elements, the access to molecules possessing different relative and absolute configurations becomes possible, which is key for the oriented synthesis of specific stereoisomers and for conducting comprehensive structure–activity relationship studies.^7^

In this context, we envisioned that the parallel syntheses of both *syn* and *anti* stereoisomers of eliglustat would illustrate the benefits of this approach (Scheme 1). Eliglustat is a glucosylceramide synthase inhibitor that suppresses glycosphingolipid biosynthesis and has been identified as an effective long-term treatment for patients with Gaucher disease type 1.^8^ Several synthetic routes have been developed for its preparation, mostly including chemical resolution of racemates, substrate-controlled, and chiral auxiliary-based methods.^9,10^ Conversely, only a few of the reported approaches rely on catalytic and enantioselective reactions to install the two stereocenters, such as a copper-mediated Henry reaction^11^ or a ruthenium-mediated asymmetric reduction of an α-amino-β-keto ester.^12^

**Scheme 1:**
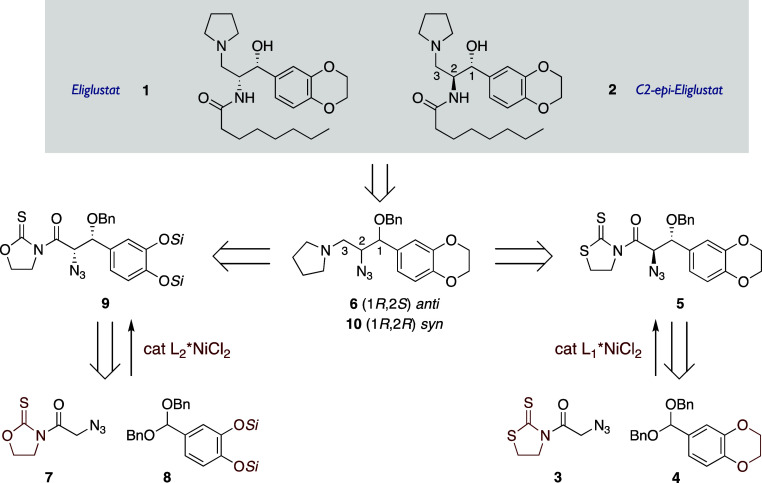
Retrosynthetic Analysis

Considering the increasing importance of asymmetric and catalytic methodologies in modern organic synthesis and building upon recent advances in stereoselective carbon–carbon bond-forming reactions, we describe herein the syntheses of eliglustat and its C2 epimer (**1** and **2**, respectively, in Scheme 1). Our approach takes advantage of highly stereocontrolled silyl triflate-mediated additions of *N*-azidoacetyl thioimides to dibenzyl acetals of aromatic aldehydes catalyzed by chiral nickel­(II) complexes, followed by appropriate transformations of the resulting enantiomerically pure adducts to enable the selective access to all the stereoisomers of eliglustat at will.^13–16^

## Results and Discussion

### Preliminary Results

Exploratory studies established that the suitable selection of the catalyst, the thioimide, and the aromatic substituents of the dialkyl acetals directs the aldol-like addition toward either the *syn* or the *anti* aldol adducts with remarkable stereocontrol.^17^

As the *anti* adducts can be consistently obtained from the reaction of *N*-azidoacetyl thiazolidinethione **3** with dialkyl acetals, regardless of the steric bulk of the alkyl group or the substituents of the aromatic ring, we anticipated that the TMSOTf-mediated addition of thioimide **3** to dibenzyl acetal **4**, catalyzed by [(*S*)-Tol-BINAP]­NiCl_2_, would afford enantiomerically pure *anti* adduct **5** with the absolute configuration of C2-*epi*-eliglustat (**2** in Scheme 1).^17^ Following such a key carbon–carbon bond-forming step, reductive removal of the heterocycle and further manipulations of the resulting alcohol would yield the advanced intermediate **6**, from which the azide could be chemoselectively converted into the desired octanamide without competing side reactions. Subsequent deprotection of the benzylic alcohol finally delivered the target C2-*epi*-eliglustat (**2**).

In a parallel approach, the reaction of oxazolidinethione **7** and dibenzyl acetal **8**, derived from silyl-protected 3,4-dihydroxybenzaldehyde, could be catalyzed by [(*R*)-DTBM-SEGPHOS]­NiCl_2_ to furnish the *syn* **9** adduct with the same absolute configuration as eliglustat (**1** in Scheme 1).^17^ As in the former sequence, removal of the heterocyclic scaffold would provide enantiomerically pure advanced intermediate **10**, from which the synthesis of eliglustat could be smoothly attained.

### Synthesis of C2-*epi*-Eliglustat

Based on the above considerations and with the aim of evaluating the feasibility of our strategy, we initially undertook the synthesis of C2-*epi*-eliglustat.^18^ As expected, the TMSOTf-mediated addition of thiazolidinethione **3** to dibenzyl acetal **4** catalyzed by 5 mol % of [(*S*)-Tol-BINAP]­NiCl_2_ at a 3 mmol scale led to a 76:24 mixture of *anti*/*syn* diastereomers from which the *anti* adduct **5** was isolated in an enantiomerically pure form (er 99:1) with a 61% yield (Scheme 2). Reductive removal of the heterocycle from **5** with LiBH_4_ gave alcohol **11** quantitatively, which was used in the next step without further purification. Treatment of **11** with mesyl chloride gave sulfonate **12** in 74% two-step yield, and the mesylate group was subsequently displaced with pyrrolidine to afford amine **6** in an 89% yield. The end game of the sequence involved the chemoselective Staudinger reduction of the azide,^19^ followed by the acylation of the resulting primary amine with octanoyl chloride to give amide **13**, and the final deprotection of the benzylic alcohol to deliver the desired C2-*epi*-eliglustat (**2** in Scheme 2).^20^ Therefore, the synthesis of **2** was achieved in seven steps and 25% overall yield from *N*-azidoacetyl thiazolidinethione **3**.

**Scheme 2:**
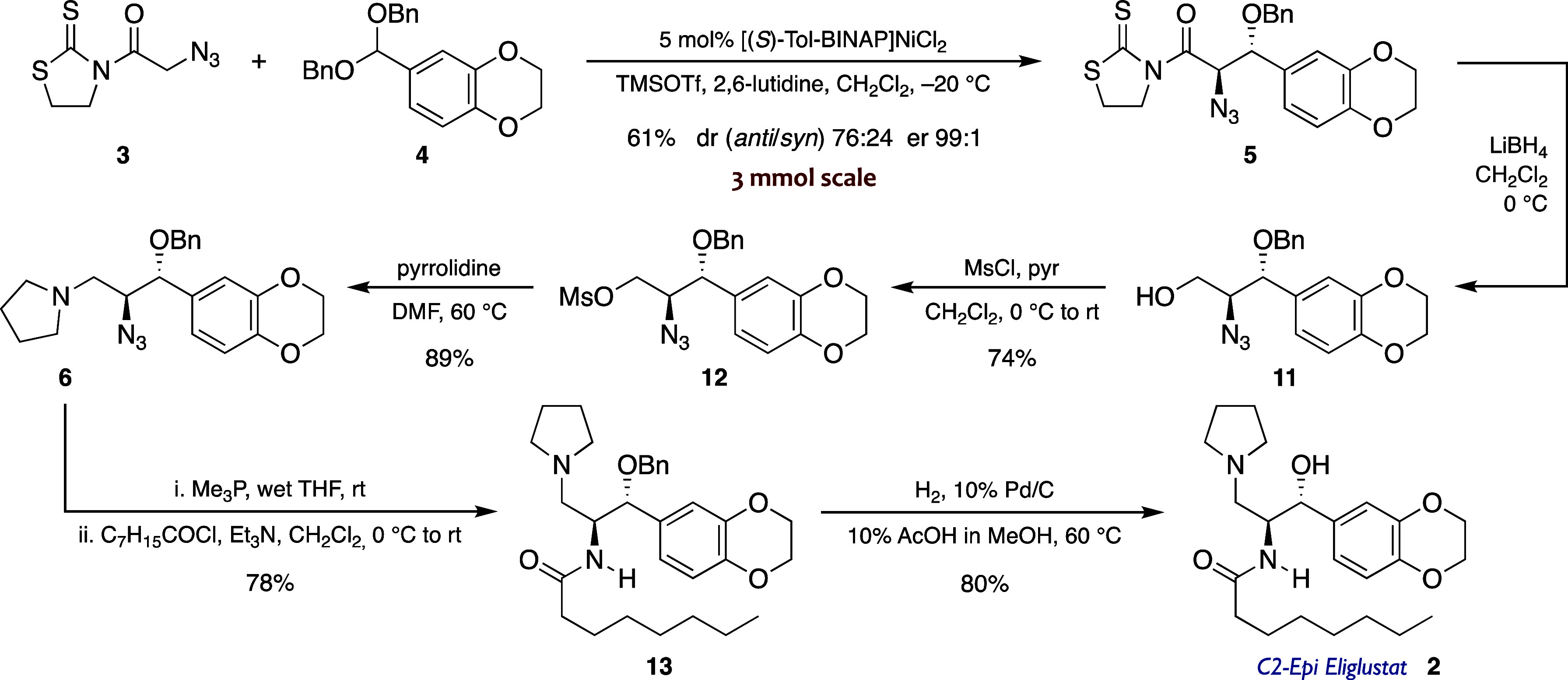
Synthesis of C2-*epi*-Eliglustat

### Studies toward the Synthesis of Eliglustat

Having completed the synthesis of *anti*-C2-*epi* eliglustat, we next focused on the diastereomeric *syn* eliglustat (**1** in Scheme 1). Given the pronounced influence of aromatic substituents of dibenzyl acetals in dictating the diastereoselectivity of the key aldol-like reaction from *N*-azidoacetyl oxazolidinethione **7**, we carried out exploratory studies to unequivocally determine the stereochemical outcome of the TMSOTf-mediated addition of **7** to dibenzyl acetals derived from 3,4-dihydroxybenzaldehyde, catalyzed by 2 mol % [(*R*)-DTBM–SEGPHOS]­NiCl_2_ (Scheme 3). Acetal **4**, featuring a sterically unhindered ethylene protecting group, delivered a 70:30 diastereomeric mixture, from which the major component **14** was obtained in an enantiomerically pure form (er 99:1) and 60% yield. However, analysis of the key coupling constant ^3^ *J*_H2–H3_ in the ^1^H NMR spectrum (^3^ *J*_H2–H3_ = 8.9 Hz) revealed that adduct **14** was the *anti* diastereomer rather than the required *syn* counterpart (eq 1 in Scheme 3). In contrast, use of dibenzyl acetal **15**, incorporating bulky silyl protecting groups, shifted the reaction toward a mixture (dr 79:21) in which the major diastereomer exhibited a significantly lower ^3^ *J*_H2–H3_ coupling constant (^3^ *J*_H2–H3_ = 4.5 Hz), consistent with the *syn* relative configuration. To our pleasure, the desired *syn* diastereomer **16** was subsequently isolated as a single enantiomer (er 99:1) in 66% yield on a 1.5 mmol scale (eq 2 in Scheme 3).

**Scheme 3:**
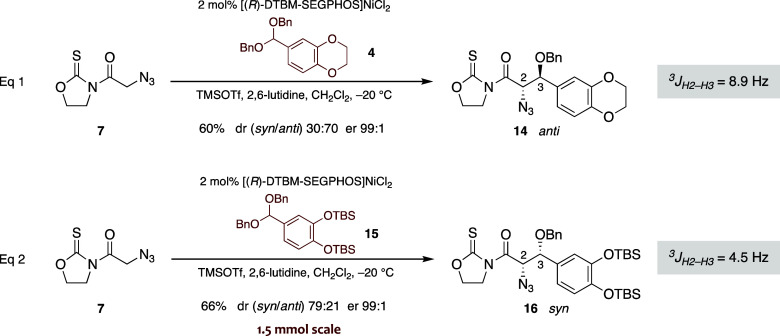
Key Carbon–Carbon Bond-Forming Reactions toward Eliglustat

### Computational Studies

The different stereochemical outcomes of the additions involving dibenzyl acetals **4** and **15** (Scheme 3) warranted further investigation. Accordingly, we conducted a detailed theoretical study of these reactions to elucidate the origin of the stereodivergent results.

Initially, we examined the structure of the putative nickel­(II) enolate derived from *N*-azidoacetyl oxazolidinethione **7**. Notably, the structure of the precatalyst [(*R*)-DTBM-SEGPHOS]­NiCl_2_ had previously been determined by X-ray diffraction,^21^ which provides a useful point of comparison for evaluating the structures arising from theoretical calculations (Figure 1). Indeed, the calculated Ni–Cl and Ni–P distances (2.23 and 2.19 Å, respectively) are in good agreement with those observed in the X-ray diffraction (2.22 and 2.15 Å, respectively). The experimental P–Ni–P angle is 94°, closely matching the calculated value of 95°, which is consistent with the constraints imposed by its chelating arrangement. Likewise, the Cl–Ni–Cl and Cl–Ni–P angles are 98° and ≈150°, respectively, whereas the calculated values are 95° and 158°. Taken together, the close correspondence between the experimental and the calculated geometric parameters underscores the strong agreement between the X-ray and the theoretical structures and lends support to our computational model. Furthermore, all these data indicate that the geometry around the nickel atom is more accurately described as distorted square-planar rather than tetrahedral, as is further supported by continuous shape measures (*S*_SQ–4_ = 7.0 and *S*_TT–4_ = 11.7).

**Figure 1:**
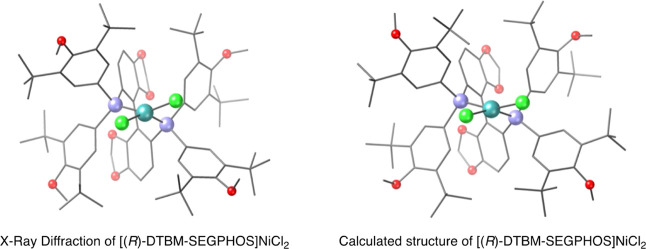
X-ray diffraction and optimized geometries of [(*R*)-DTBM-SEGPHOS]­NiCl_2_.

The substitution of both chlorides in precatalyst [(*R*)-DTBM-SEGPHOS]­NiCl_2_ by the exocyclic sulfur and the oxygen atoms of the nickel­(II) enolate generates four different conformers depending on the relative arrangement of the chelate and the oxazolidinethione ring. As these conformers exhibit very similar geometries, only the most stable one was considered for the analysis of their reactivity. From a structural point of view, the coordination environment around the nickel center became more planar (*S*_SQ–4_ = 2.8), with S–Ni–P and O–Ni–P angles of 160° and 167°, respectively, approaching ideal square-planar geometry (Figure 2). The reduced distortion around the nickel atom is associated with a small S–Ni–O angle of 89°, imposed by the chelated arrangement, while the P–Ni–P angle slightly increases to 99°. Finally, the metal–ligand bond lengths are consistent with expected values, including the absence of a *trans* effect for the two non-equivalent Ni–P bonds.

**Figure 2:**
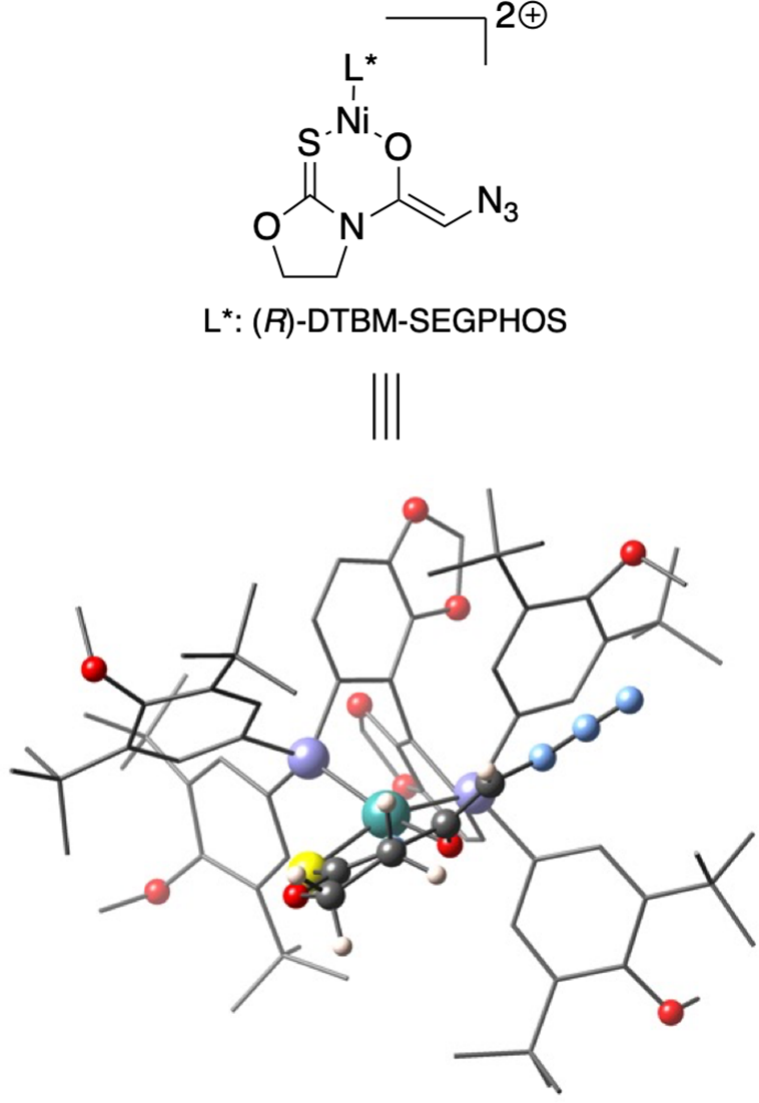
Structure of the nickel­(II) enolate.

Having established the structure of the enolate and aiming to understand the stereochemical outcomes observed for dibenzyl acetals **4** and **15**, we next carried out a comprehensive computational study of the reactions presented in Scheme 3. The calculations revealed that the corresponding oxocarbenium intermediates from **4** and **15** can approach only the *Si* π-face of the enolate, as access to the opposite *Re* π-face is sterically hindered by the bulky *tert*-butyl groups on the phosphine moieties. Accordingly, our analysis focused exclusively on the *Si* π-face approach using the most stable conformation of the oxazolidinethione ring. This approach generated two transition states, **TS**_**4**_ **–A1** and **TS**_**4**_ **–A2** for acetal **4** and **TS**_**15**_ **–A1** and **TS**_**15**_ **–A2** for acetal **15** in Figure 3, which differ in the relative disposition of the oxocarbenium substituents.

**Figure 3:**
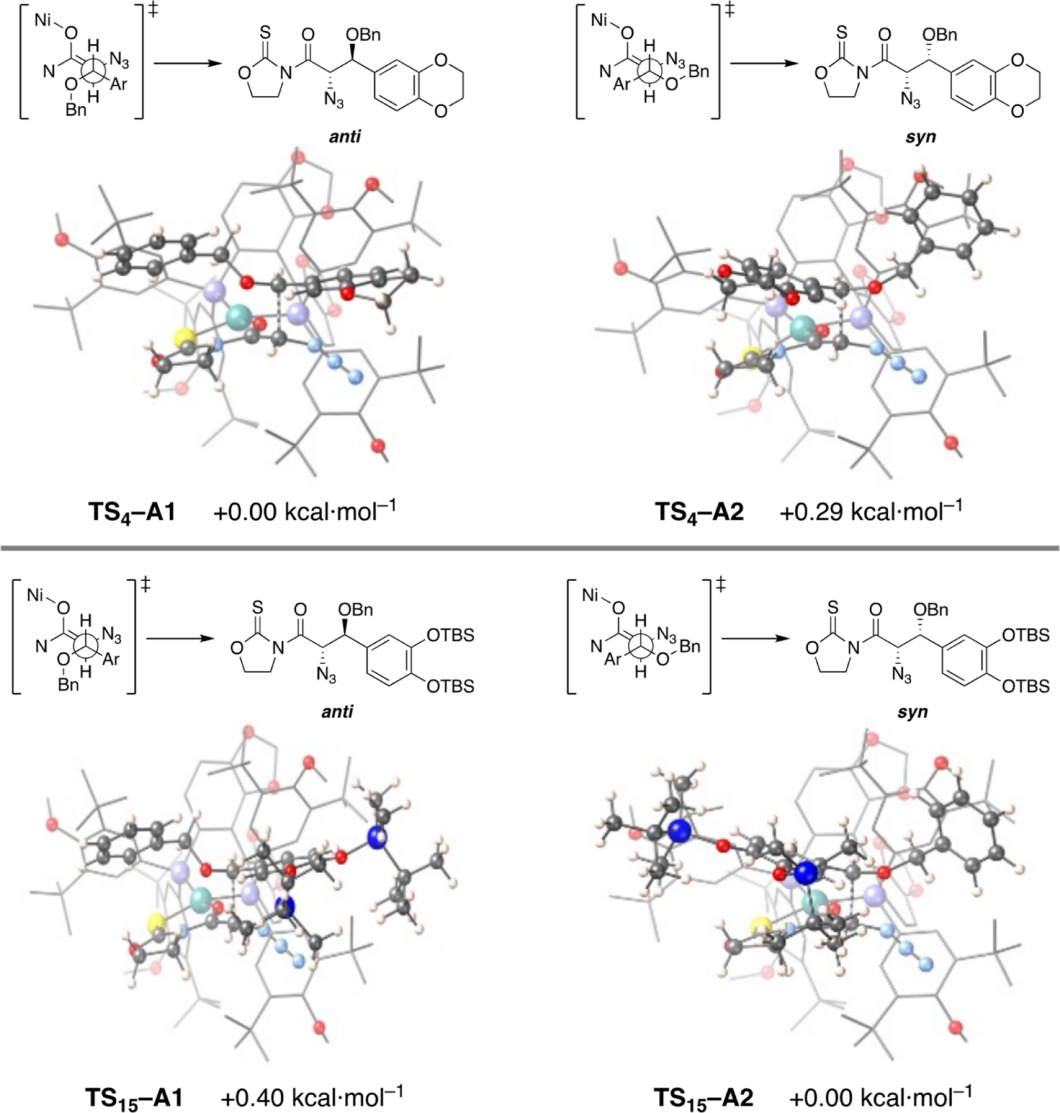
Calculated transition states for the reaction of oxocarbenium intermediates from acetals 4 (top) and 15 (bottom).

The geometries of **TS–A1** and **TS–A2** involve a distorted planar environment for the metal center (*S*_SQ–4_ = 3.0–3.4), with angles for the opposite atoms and the diphosphine close to 162° and 96°, respectively. Furthermore, distances for the new C···C bond in **TS–A1** are slightly longer than those in **TS–A2**, while the dihedral angle for the two methine groups ranges from 157° to 167°. Nevertheless, differences between approaches **TS–A1** and **TS–A2** became clear when analyzing their relative energies. The transition state **TS**_**4**_ **–A1** of the oxocarbenium intermediate from acetal **4** containing a dioxanyl moiety at the aromatic ring is more stable than the **TS**_**4**_ **–A2** counterpart by 0.29 kcal mol^–1^ (Figure 3 top). By application of the Boltzmann distribution, the predicted diastereomeric *syn*/*anti* ratio of 36:64 is consistent with the 30:70 value obtained experimentally. In comparison, the oxocarbenium arising from acetal **15**, which contains two TBS groups that dramatically increase the steric hindrance of the aromatic moiety, prefers to approach **A2**, so **TS**_**15**_ **–A2** turns out to be more stable than **TS**_**15**_ **–A1** by 0.40 kcal mol^–1^ (Figure 3 bottom). This reversal gives a diastereomeric *syn*/*anti* ratio of 69:31, in line with the 79:21 value obtained experimentally.

These calculations indicate that *tert*-butyl groups of the phosphine moieties in the chiral ligand determine the π-face of the enolate to which electrophilic oxocarbenium intermediates approach, while the observed diastereoselectivity (*syn* versus *anti*) arises from the optimal accommodation of the aryl and the benzyloxy substituents at the electrophilic center, minimizing steric interactions with the chiral environment.

### Synthesis of Eliglustat

Two main lessons were learned from former studies. First, dibenzyl acetal **4**, bearing the 1,4-dioxane motif, proved suitable exclusively for the synthesis of C2-*epi* eliglustat **2**, irrespective of the thioimide or catalyst employed. Second, successful synthesis of eliglustat required the stringent combination of oxazolidinethione **7**, [DTBM-SEGPHOS]­NiCl_2_ catalyst, and a dibenzyl acetal with bulky silyl-protected phenol groups on the aromatic ring, such as **15**. As a result, reconstruction of the characteristic oxygenated heterocycle^22^ became essential once the C2 and C3 stereocenters had been properly installed through the reaction of oxazolidinethione **7** with dibenzyl acetal **15**, in which both phenol groups are protected as silyl ethers.

Based on these findings, the synthesis of eliglustat was pursued, as outlined in Scheme 4. Reduction of *syn* adduct **16** and subsequent treatment of resulting alcohol **17** with TBAF afforded trihydroxy derivative **18** in an 85% two-step yield. Then, reconnection of both phenol groups with an ethylene handle was attempted. Initial efforts employing ethylene dibromide as the alkylating agent under different conditions consistently resulted in complex mixtures.^23^ The use of the more reactive ethylene ditosylate did not improve these results either. Finally, much more active ethylene bistriflate under phase transfer conditions enabled the formation of the desired dioxane **19** in 50% yield.^24^ Transformation of the hydroxyl group in **19** with mesyl chloride and displacement of the resulting mesylate with pyrrolidine provided advanced intermediate **10** in an 84% yield. Comparison of the spectra from amines **6** and **10** (Scheme 1) confirmed that they were indeed diastereomers, thereby validating the use of acetal **15** in the synthesis of eliglustat. As in the case of C2-*epi*-eliglustat (Scheme 2), the final steps involved Staudinger reduction of the azide, followed by acylation of the resulting primary amine with octanoyl chloride to give amide **20** in 69% two-step yield. Eventually, deprotection of the benzylic alcohol delivered the targeted eliglustat (**1**) in 75% yield (Scheme 4), with spectroscopic data matching those reported in the literature.^25^ Overall, the synthesis of eliglustat (**1** in Scheme 1) was accomplished in nine steps and 12% yield from *N*-azidoacetyl oxazolidinethione **7**.

**Scheme 4:**
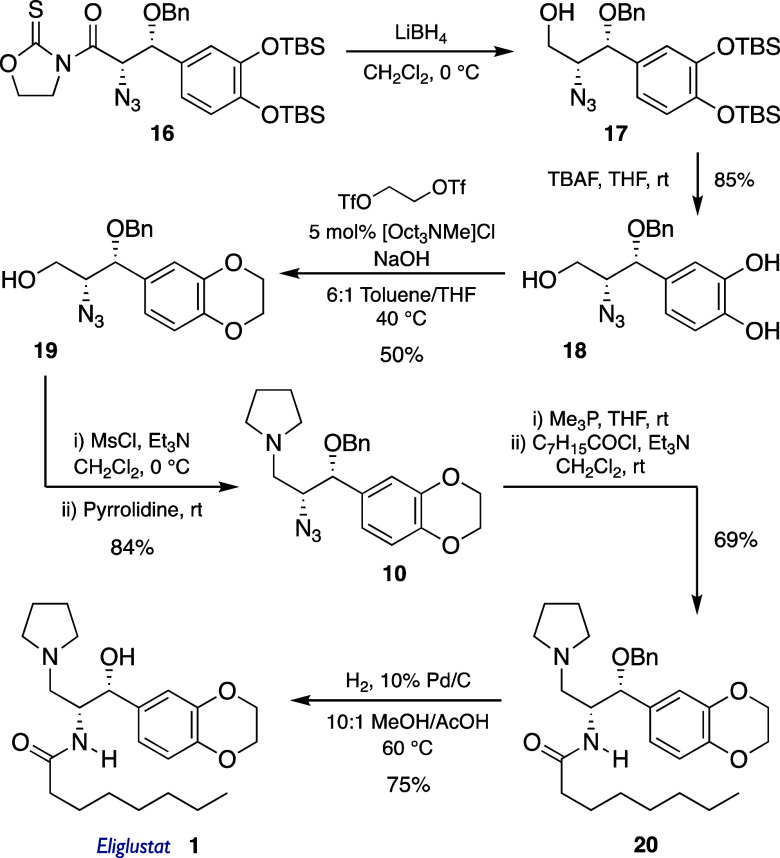
Synthesis of Eliglustat

At this point, considering both the experimental and theoretical results reported in this and earlier studies,^17^ it is reasonable to assert that the stereochemical outcome of aldol-like additions of thioimides to oxocarbenium intermediates generated from aromatic acetals catalyzed by chiral [DTBM-SEGPHOS]­NiCl_2_ complexes is strongly governed by the steric demands of the aromatic moiety. Accordingly, a rigorous analysis is necessary to delineate the synthetic route best suited to the desired *syn* diastereomers.

## Conclusions

In summary, we have successfully completed the asymmetric total syntheses of eliglustat (**1**) and its C2-epimer (**2**) in nine and seven steps, respectively, with overall yields of 10–25% using a common approach. The key step involves the forging of the C1–C2 carbon bond through Lewis acid-mediated, stereocontrolled additions of azidoacetyl thioimides to dibenzyl acetals catalyzed by chiral nickel­(II) complexes. Both experimental and theoretical studies indicate that the nature of the substituents on the aromatic moiety is crucial for determining the stereochemical outcome of the carbon–carbon bond-forming step; indeed, a dioxanyl-like substituent consistently leads to the *anti* relative configuration found in C2-*epi*-eliglustat, whereas bulky TBS protecting groups of phenols favor the *syn* configuration required for eliglustat. Importantly, this approach enables the chemoselective acylation of the amine at C2 without competition from the C1-hydroxyl counterpart in the final steps of the syntheses. Therefore, the appropriate choice of the starting thioimide, acetal, and catalyst gives access to any of the potential stereoisomers of eliglustat at will, providing a valuable platform for the accurate evaluation of their biological activity.

From a broader perspective, this approach complements previously reported strategies based on the asymmetric and catalytic construction of key carbon–carbon bonds^11,12^ while also enabling the enantioselective synthesis of all potential stereoisomers of targeted eliglustat through the use of robust nickel­(II) complexes containing commercially available ligands, rather than bespoke ligands as required in earlier approaches.^11,12^

## Supplementary Material



## Data Availability

The data underlying this study are available in the published article and its Supporting Information.

## Notes

Experimental procedures, details of computational calculations, and copies of the NMR spectra and chromatograms (PDF)
