# Supplementary material for: Asymmetric Total Syntheses of Eliglustat and C2-epi-Eliglustat
Source: J Org Chem. 2026 Feb 16;91(8):3329–35. doi: 10.1021/acs.joc.5c03229 (PMC12954755; doi:10.1021/acs.joc.5c03229)

Supporting Information

-

Experimental Procedures

Computational Calculations

NMR Spectra

Asymmetric Total Syntheses of Eliglustat and C2 *epi*-Eliglustat

Miguel Mellado-Hidalgo,[a] Anna M. Costa,[a,*] Pedro Romea, [a,*] Fèlix Urpí,[a,*] and Gabriel Aullón[b]

a Department of Inorganic and Organic Chemistry, Section of Organic Chemistry, and Institut de Biomedicina de la Universitat de Barcelona (IBUB), Universitat de Barcelona, Carrer Martí i Franqués 1–11, 08028 Barcelona, Catalonia, Spain

b Department of Inorganic and Organic Chemistry, Section of Inorganic Chemistry, and Institut de Química Teòrica i Computacional (IQTC) de la Universitat de Barcelona, Universitat de Barcelona, Carrer Martí i Franqués 1–11, 08028 Barcelona, Catalonia, Spain

Contents

1. General Experimental Methods S3
2. Summary of the Syntheses S4
    2.1. C2 *epi*-Eliglustat S4
    2.2. Eliglustat S4
3. Total Synthesis of C2 *epi*-Eliglustat S5
4. Total Synthesis of Eliglustat S13
5. Computational Calculations S25
6. References S52
7. Copies of the NMR Spectra S53

1. General Experimental Methods

Unless otherwise noted, reactions were conducted in oven-dried glassware under inert atmosphere of N_2 with anhydrous solvents. The solvents and reagents were dried and purified when necessary according to standard procedures. Commercially available reagents were used as received.

Analytical thin-layer chromatography (TLC) was carried out on Merck silica gel 60 F_{254} plates and analysed by UV (254 nm) and stained with *p*-anisaldehyde; column chromatographies were carried under low pressure (flash) conditions and performed on SDS silica gel 60 (35–70 μm). Eluents are indicated in brackets in each case. **R_f** values are approximate.

Chiral HPLC analyses were conducted on a Shimadzu LC-20 HPLC system, using chiral Phenomenex Lux® columns under isocratic conditions and UV-detected at 254 nm.

Melting points (**Mp**) were determined with a Gallenkamp MPD350.BM2.5 apparatus and are uncorrected.

Specific rotations (**$[\alpha]_D$**) were determined at 20 °C on a Perkin-Elmer 241 MC polarimeter equipped with a sodium lamp (λ 589 nm, D-line).

IR spectra (Attenuated Total Reflectance, ATR) were recorded on a Nicolet 6700 FT-IR Thermo Scientific spectrometer and only the more representative frequencies (ν) are reported in cm^{-1}.

^{1}H NMR (400 MHz) and **^{13}C NMR** (100.6 MHz) spectra were recorded at room temperature on a Varian Mercury 400. **^{1}H NMR** (500 MHz) spectra was recorded at room temperature on a Bruker 500. Chemical shifts (δ) are quoted in ppm and referenced to internal TMS (δ 0.00 for ^{1}H NMR) and $CDCl_3$ (δ 77.0 for ^{13}C NMR). Data are reported as follows: chemical shift (number of protons, multiplicity, coupling constants, proton); multiplicity is reported as follows: s, singlet; d, doublet; t, triplet; q, quartet; or m, multiplet (and their corresponding combinations); coupling constants (*J*) are quoted in Hz. When necessary, 2D techniques (NOESY, COSY, HSQC) were also used to assist on structure elucidation.

Reversed-phase UHPLC–MS analyses were conducted on a Waters Acquity ARC system coupled to a MS Acquity QDa and a 2998 PDA detector by the Serveis de la SQO, de la Universitat de Barcelona.

High resolution mass spectra (**HRMS**) were obtained with a LTQ Orbitrap Velos (Thermo Fisher Scientific) SN 03134B spectrometer by the Unitat d'Espectrometria de Masses, Universitat de Barcelona.

Caution! *Organic azides are sensitive compounds that may decompose violently under heat, friction, or impact. Azides reported along this manuscrit are oils and did not require any special handling, but they should still be kept away from metal surfaces and elevated temperatures. Contact with strong acids, reducing agents, or other incompatible materials should also be avoided.*

2. Summary of the Syntheses

2.1. C2 *epi*-Eliglustat

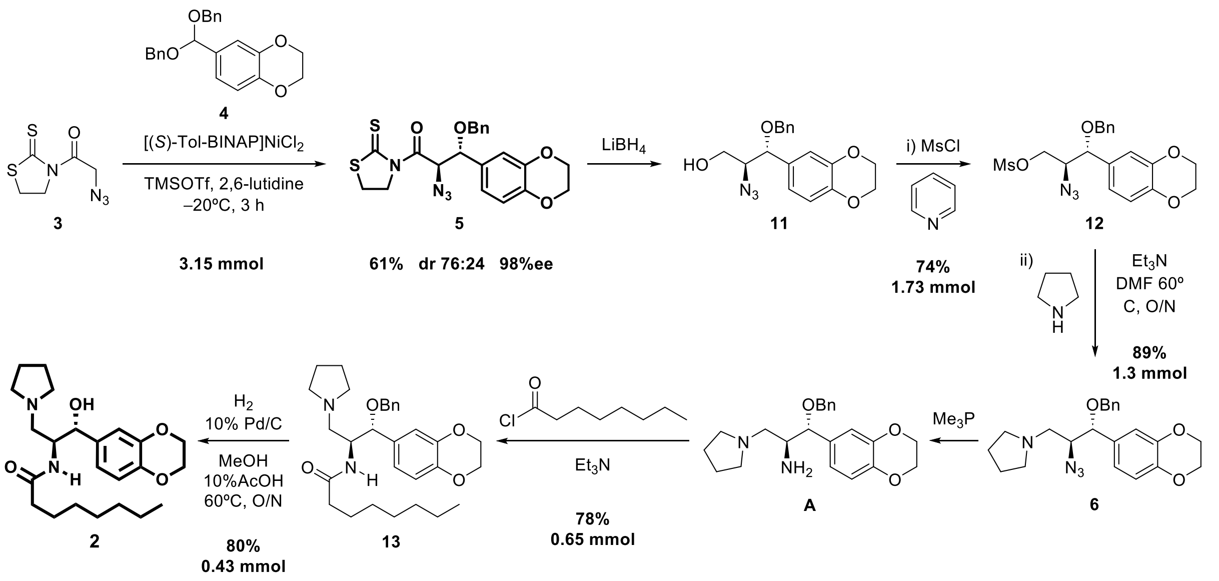

2.2. Eliglustat

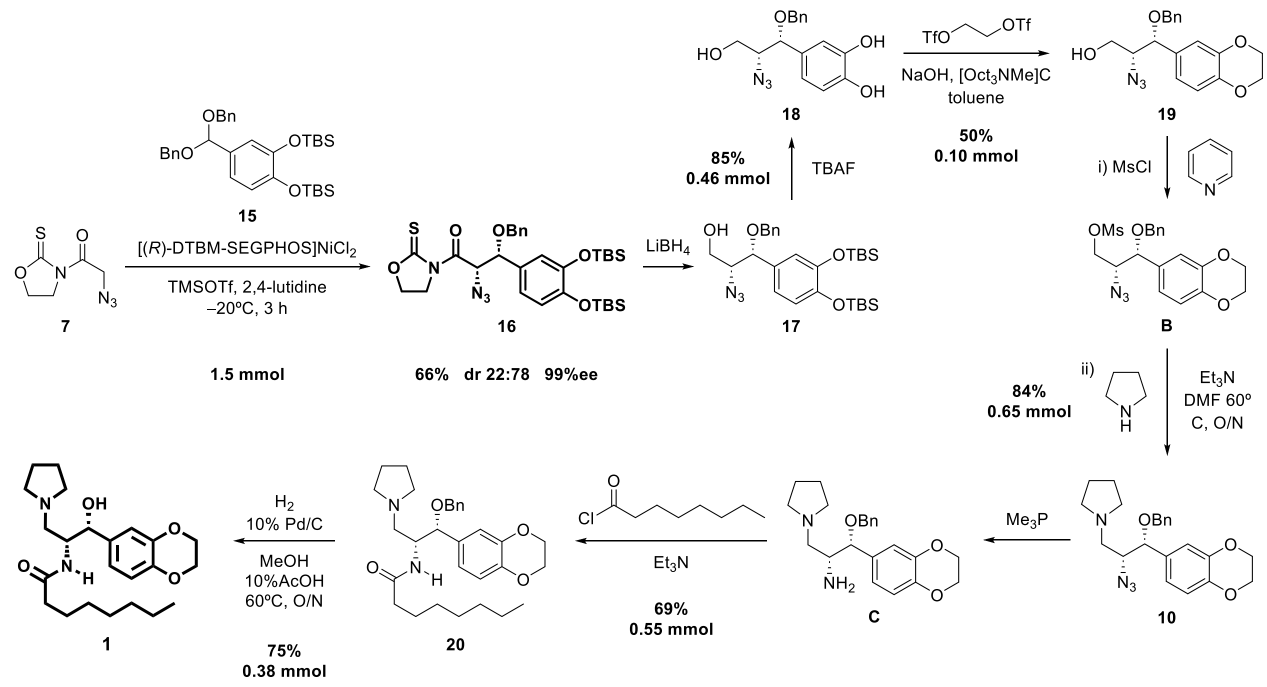

3. Total Synthesis of C2 *epi*-Eliglustat

N-(2-Azidoacetyl)-1,3-thiazolidine-2-thione (3)

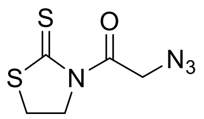

Neat 2-bromoacetic acid (3.1 g, 22.2 mmol, 1.0 equiv) in distilled H_2O (17 mL, 1.3 M) was added dropwise to an aqueous solution (15 mL, 3M) of sodium azide (2.9 g, 44.4 mmol, 2 equiv) at 0 °C. After 15 min, the ice bath was removed and the reaction mixture was stirred overnight at room temperature.

The reaction was then slowly acidified with 2 M HCl until pH 1 and the aqueous solution was extracted with Et_2O (3 × 20 mL). The combined organic extracts were dried over $NaSO_4$, filtered, and the solvent was removed under reduced pressure to afford 2-azidoacetic acid (99% yield) as a colourless oil which was used in the next step without further purification.

The formed 2-azidoacetic acid (2.24 g, 22.2 mmol, 1.1 equiv) in CH_2Cl_2 (22 mL, 1 M) was added dropwise to a solution of 1,3-thiazolidine-2-thione (2.38 g, 20.0 mmol, 1 equiv), EDC·HCl (4.6 g, 24.0 mmol, 1.2 equiv), and DMAP (125 g, 1.0 mmol, 0.05 equiv) in CH_2Cl_2 (40 mL, 0.5 M) at 0 °C and stirred for 15 min. The solution was then allowed to reach room temperature and was stirred overnight.

The reaction mixture was washed with deionized H_2O and brine. The organic layers were combined, dried over $NaSO_4$ and filtered. After removing the solvent *in vacuo*, the crude product was purified by flash column chromatography (70:30 Hexanes/EtOAc) to afford **3** (2.95 g, 14.6 mmol, 73% yield) as a yellow solid.

Yellow solid.
Mp 57–59 °C
R$_f$ 0.40 (70:30 Hexanes/EtOAc).
IR (ATR) ν 2904, 2097, 1698, 1395, 1349, 1264, 1220, 1157, 1043 cm^{-1}.
^{1}H NMR (400 MHz, $CDCl_3$) δ 4.84 (2H, s, C$\underline{H_2}$N$_3$), 4.64 (2H, t, *J* = 7.6 Hz, NC$\underline{H_2}$), 3.41 (2H, t, *J* = 7.6 Hz, SC$\underline{H_2}$).
^{13}C{^{1}H} NMR (100.6 MHz, $CDCl_3$) δ 201.5 (C), 169.4 (C), 55.6 (CH_2), 55.3 (CH_2), 29.2 (CH_2).
HRMS (+ESI): *m/z*calcd. for $[M + H]^+$ $C_5H_7N_4OS_2$: 203.0055; found: 203.0061.

Compound **3** has also been reported elsewhere. [1][2][3]

***N*-[(2*R*,3*R*)-2-Azido-3-(benzyloxy)-3-(2,3-dihydrobenzo[*b*][1,4]dioxin-6-yl)propanoyl]-1,3-thiazolidine-2-thione (5)**

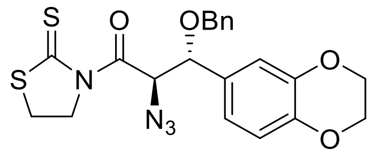

A solution of *N*-(2-azidoacetyl)-1,3-thiazolidine-2-thione (**3**, 102 mg, 0.50 mmol 1.0 equiv), 6-(bis(benzyloxy)methyl)-2,3-dihydrobenzo[b][1,4]dioxine (**4**, 200 mg, 0.55 mmol, 1.1 equiv) and [(*S*)-Tol-BINAP]NiCl$_2$ (20.4 mg, 25 µmol, 5 mol%) in CH$_2$Cl$_2$ (2 mL, 0.25 M) was cooled at –20 °C under a N$_2$ athmosphere. Then, neat TMSOTf (120 µL, 0.65 mmol 1.3 equiv) was added dropwise to the stirring green-brown solution followed by 2,6-lutidine (90 µL, 0.75 mmol 1.5 equiv) addition, and the resultant dark red mixture was stirred at –20 °C for 3 h.

The mixture was quenched with sat NH$_4$Cl (2 mL) and partitioned in CH$_2$Cl$_2$ (15 mL) and water (15 mL). The aqueous layer was then extracted with neat CH$_2$Cl$_2$ (2 × 15 mL), and the combined organic extracts were dried over Na$_2$SO$_4$ and concentrated *in vacuo*. Finally, the residue (dr 76:24) was purified by column chromatography (80:20 Hexanes/EtOAc) to afford 139 mg (0.30 mmol, 61% yield) of *anti* adduct **5** and 42 mg (0.09 mmol, 18% yield) of the *syn* diastereomer.

The reaction was also carried out at 3.15 mmol scale (dr 76:24) to afford 875 mg (61% yield) of the proper *anti* adduct.

Yellow oil.
R$_f$ 0.30 (80:20 Hexanes/EtOAc).
Chiral HPLC (Phenomenex Lux® Cellulose-1 column, 40% *i*-PrOH in hexane, flow rate 1.0 mL·min^{-1}): Rt 12.6 min (Major 2*R*,3*R*-isomer) [Rt 21.8 min (minor 2*S*,3*S*-isomer)], 98% ee.
[α]$_D^{20}$ –80.5 (*c* 1.00, CHCl$_3$).
IR (ATR) ν 2924, 2872, 2108, 17 02, 1506, 1359, 1284, 1169, 1066, 1003, 887 cm^{-1}.
^{1}H NMR (400 MHz, CDCl$_3$) δ 7.35–7.23 (6H, m, Ar<u>H</u>), 6.98 (1H, d, *J* = 1.7 Hz, Ar<u>H</u>), 6.92 (1H, dd, *J* = 2.3, 1.2 Hz, Ar<u>H</u>), 6.14 (1H, d, *J* = 8.8 Hz, C<u>H</u>N$_3$), 4.64 (1H, d, *J* = 8.8 Hz, C<u>H</u>OCH$_2$Ph), 4.45 (1H, d, *J* = 11.4 Hz,OC<u>H</u>$_a$H$_b$Ph), 4.46–4.34 (2H, m, OC<u>H</u>$_2$), 4.29(4H, s, OC<u>H</u>$_2$C<u>H</u>$_2$O), 4.20 (1H, d, *J* = 11.4 Hz, OCH$_a$<u>H</u>$_b$Ph), 3.12–3.00 (2H, m, NC<u>H</u>$_2$).
^{13}C{^{1}H} NMR (100.6 MHz, CDCl$_3$) δ 202.1 (C), 171.7 (C), 144.3 (C), 143.8 (C), 137.6 (C), 130.0 (C), 128.3 (CH), 127.8 (CH), 127.7 (CH), 120.9 (CH), 117.7 (CH), 116.5 (CH), 82.4 (CH), 70.9 (CH$_2$), 64.3 (CH$_2$), 64.3 (CH$_2$), 64.2 (CH), 56.3 (CH$_2$), 28.9 (CH$_2$).
HRMS (+ESI): *m/z* calcd. for [M – OBn – N$_2$]$^+$ C$_{14}$H$_{13}$N$_2$O$_3$S$_2$: 321.0362; found: 321.0368. *m/z* calcd. For [M + Na]$^+$ C$_{21}$H$_{20}$N$_4$NaO$_4$S$_2$: 479.0818; found: 479.0826.

(2*S*,3*R*)-2-Azido-3-benzyloxy-3-(2,3-dihydrobenzo[*b*][1,4]dioxin-6-yl)propan-1-ol (11)

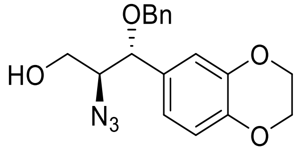

A 2 M solution of $LiBH_4$ in THF (285 μL, 0.57 mmol, 2.0 equiv) was added to a solution of adduct **5** (130 mg, 0.285 mmol, 1.0 equiv) in CH_2Cl_2 (3 mL) at 0 °C under N_2 atmosphere and the reaction mixture was stirred at 0 °C for 3 h.

The reaction mixture was carefully quenched with MeOH (2 mL), and the crude was then diluted with CH_2Cl_2 (2 mL) and washed with NaOH 0.5 M (10 mL). The aqueous layer was extracted with more CH_2Cl_2 (2 × 5 mL), and the combined organic extracts were dried over $MgSO_4$ and concentrated under vacuum. The crude mixture (100 mg) was analysed and used in the next step without further purification.

The reaction was also carried out at 1.73 mmol scale.

Pale yellow oil.
R$_f$ 0.25 (80:20 Hexanes/EtOAc).
$[\alpha]_D^{20}$ –107.6 (*c* 1.0, $CHCl_3$).
IR (ATR) ν 3416 (br), 2928, 2874, 2097, 1590, 1506, 1305, 1284, 1258, 1064, 887, 740 cm^{-1}.
^{1}H NMR (400 MHz, $CDCl_3$) δ 7.37–7.27 (5H, m, Ar<u>H</u>), 6.92 (1H, d, *J* = 2.0 Hz, Ar<u>H</u>), 6.91 (1H, d, *J* = 8.2 Hz, Ar<u>H</u>), 6.86 (1H, dd, *J* = 8.2, 2.0 Hz, Ar<u>H</u>), 4.52 (1H, d, *J* = 11.6 Hz, OC<u>H$_a$</u>H$_b$Ar), 4.35 (1H, d, *J* = 7.0 Hz, C<u>H</u>OBn), 4.29 (4H, s, OC<u>H$_2$</u>C<u>H$_2$</u>O), 4.27 (1H, d, *J* = 11.6 Hz, OCH$_a$<u>H$_b$</u>Ar), 3.83 (1H, dd, *J* = 11.6, 4.5 Hz, C<u>H$_a$</u>H$_b$OH), 3.75 (1H, dd, *J* = 11.6, 5.6 Hz, CH$_a$<u>H$_b$</u>OH), 3.61 (1H, ddd, *J* = 7.0, 5.6, 4.5 Hz, C<u>H</u>N$_3$), 2.15 (1H, br s, O<u>H</u>).
^{13}C{^{1}H} NMR (100.6 MHz, $CDCl_3$) δ 143.9 (C), 143.8 (C), 137.4 (C), 131.0 (C), 128.5 (CH), 127.9 (CH), 127.9 (CH), 120.6 (CH), 117.5 (CH), 116.3 (CH), 80.9 (CH), 70.6 (CH_2), 67.4 (CH), 64.3 (CH_2), 62.7 (CH_2).
HRMS (+ESI): *m/z* calcd. for $[M – OBn – N_2 + 2H]^+$ $C_{11}H_{14}NO_3$: 208.0968; found: 208.0960. *m/z* calcd. for $[M – N_2 + 3H]^+$ $C_{18}H_{22}NO_4$: 316.1543; found: 316.1532.

1-[(2*S*,3*R*)-2-Azido-3-benzyloxy-3-(2,3-dihydrobenzo[*b*][1,4]dioxin-6-yl)propyl]methanesulfonate (12)

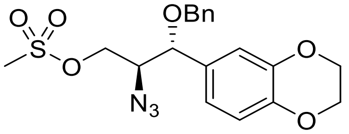

Neat mesyl chloride (45 µL, 0.57 mmol, 2.0 equiv) and pyridine (50 µL, 0.57 mmol, 2.0 equiv) were slowly added to a solution of residue **11** (0.285 mmol, 1.0 equiv) in CH_2Cl_2 (3 mL) at 0 °C under N_2 atmosphere. The reaction mixture was then stirred at room temperature until complete consumption of the initial alcohol.

After 4 h, the crude was quenched with H_2O (10 mL) and the resulting mixture was extracted with CH_2Cl_2 (3 × 5 mL). The combined organic layers were dried over $MgSO_4$ and concentrated *in vacuo*, and the resulting residue was purified via column chromatography (from 30:70 to 0:100 Hexanes/CH_2Cl_2) to afford 93 mg (0.22 mmol, 78% yield) of mesylate **12**.

The reaction was also carried out at 1.73 mmol scale to afford 539 mg (1.28 mmol, 74% yield) of the *anti* mesylate **12**.

Colorless oil.
R$_f$ 0.25 (30:70 Hexanes/CH_2Cl_2).
$[\alpha]_D^{20}$ –90.2 (*c* 1.0, $CHCl_3$).
IR (ATR) ν 3030, 2935, 2876, 2100, 1590, 1506, 1355, 1284, 1174, 1064, 965, 816 cm^{-1}.
^{1}H NMR (400 MHz, $CDCl_3$) δ 7.37–7.27 (5H, m, Ar<u>H</u>), 6.93–6.90 (2H, m, Ar<u>H</u>), 6.86–6.83 (1H, m, Ar<u>H</u>), 4.50 (1H, d, *J* = 11.6 Hz, OC<u>H</u>$_a$H$_b$Ph), 4.46 (1H, dd, *J* = 10.7, 3.2 Hz, C<u>H$_a$</u>H$_b$OSO$_2$CH$_3$), 4.32–4.27 (2H, m, C<u>H</u>OBn & CH$_a$<u>H$_b$</u>OSO$_2$CH$_3$), 4.29 (4H, s, OC<u>H</u>$_2$C<u>H</u>$_2$O), 4.26 (1H, d, *J* = 11.6 Hz, OCH$_a$<u>H</u>$_b$Ph), 3.82 (1H, td, *J* = 7.3, 3.1 Hz, C<u>H</u>N$_3$), 3.02 (3H, s, OSO$_2$C<u>H</u>$_3$).
^{13}C{^{1}H} NMR (100.6 MHz, $CDCl_3$) δ 144.1 (C), 143.9 (C), 137.2 (C), 130.0 (C), 128.5 (CH), 128.0 (CH), 127.9 (CH), 120.6 (CH), 117.7 (CH), 116.4 (CH), 79.1 (CH), 70.6 (CH_2), 68.3 (CH_2), 64.8 (CH), 64.3 (CH_2), 37.6 (CH_3).
HRMS (+ESI): *m/z* calcd. for $[M + Na]^+$ $C_{19}H_{21}N_3NaO_6S$: 442.1043; found: 442.1027. *m/z* calcd. for $[2M – OBn – N_2 – 2H]^+$ $C_{31}H_{33}N_4O_{11}S_2$: 701.1582; found: 701.2306.

1-[(2*S*,3*R*)-2-Azido-3-benzyloxy-3-(2,3-dihydrobenzo[*b*][1,4]dioxin-6-yl)propyl]pyrrolidine (6)

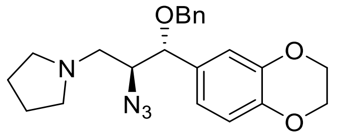

Neat pyrrolidine (72 µL, 0.86 mmol, 4.0 equiv) was added to a solution of the mesylate **12** (90 mg, 0.21 mmol, 1.0 equiv) in DMF (5 mL) in an oil bath at 60 °C under N_2 atmosphere and the reaction mixture was stirred at 60 °C for 16 h.

After stirring overnight, the reaction was cooled down to room temperature and a kind of precipitate was observed when quenching the crude with H_2O (5 mL). The mixture was then extracted with EtOAc (2 × 10 mL), and the combined organic extracts were dried over $MgSO_4$ and concentrated under reduced pressure. The crude was finally purified via column chromatography (from 80:20 to 70:30 CH_2Cl_2/EtOAc) to afford 75 mg (0.19 mmol, 90% yield) of tertiary amine **6**.

The reaction was also carried out at 1.3 mmol scale to afford 455 mg (1.15 mmol, 89% yield) of the *anti* tertiary amine **6**.

Pale orange oil.

R_f 0.10 (90:10 CH_2Cl_2/EtOAc).

$[\alpha]_D^{20}$ −102.4 (*c* 1.0, $CHCl_3$).

IR (ATR) ν 2958, 2926, 2874, 2792, 2100, 1506, 1284, 1258, 1068, 877 cm^{-1}.

^{1}H NMR (400 MHz, $CDCl_3$) δ 7.37–7.27 (5H, m, Ar$\underline{H}$), 6.92 (1H, d, *J* = 1.8 Hz, Ar$\underline{H}$), 6.89–6.84 (2H, m, Ar$\underline{H}$), 4.54 (1H, d, *J* = 11.7 Hz, OC$\underline{H}_a$$H_b$Ph), 4.40 (1H, d, *J* = 5.1 Hz, C$\underline{H}$OBn), 4.30 (1H, d, *J* = 11.7 Hz, OC$H_a$$\underline{H}_b$Ph), 4.28 (4H, s, OC$\underline{H}_2C\underline{H}_2$O), 3.79 (1H, dt, *J* = 9.3, 4.8 Hz, C$\underline{H}$$N_3$), 2.62–2.51 (6H, m, C$\underline{H}_2N(CH_2)_4$ & CH_2N(C$\underline{H}_2$$)_4$), 1.81–1.72 (4H, m, N(C$\underline{H}_2$$)_4$).

^{13}C{^{1}H} NMR (100.6 MHz, $CDCl_3$) δ 143.5 (C), 143.5 (C), 138.0 (C), 130.7 (C), 128.3 (CH), 127.6 (CH), 127.6 (CH), 120.9 (CH), 117.2 (CH), 116.6 (CH), 81.4 (CH), 70.5 (CH_2), 65.1 (CH), 64.3 (CH_2), 55.8 (CH_2), 54.3 (CH_2), 23.5 (CH_2).

HRMS (+ESI): *m*/*z* calcd. for $[M + H]^+$ $C_{22}H_{27}N_4O_3$: 395.2078; found: 395.2065.

(1*R*,2*S*)-1-Benzyloxy-1-(2,3-dihydrobenzo[*b*][1,4]dioxin-6-yl)-3-(pyrrolidin-1-yl)propan-2-amine (A)

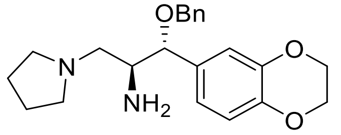

A 1 M solution of Me_3P in THF (880 μL, 0.88 mmol, 1.1 equiv) was added to a solution of tertiary amine **6** (321 mg, 0.81 mmol, 1.0 equiv) in THF (5 mL) and H_2O (250 μL) at 0 °C under N_2 atmosphere, and the reaction was stirred at 0 °C for 30 min and at room temperature for 16 h.

The mixture was diluted with CH_2Cl_2 (10 mL) and the crude was washed with 0.5 M NaOH (10 mL). The aqueous layer was then extracted with clean CH_2Cl_2 (2 × 10 mL) and the resulting combined organic layers were dried over $MgSO_4$ and concentrated under *vacuum*. The crude mixture was analysed and used in the next step without further purification.

Pale yellow oil.

R_f 0.25 (90:10 CH_2Cl_2/MeOH).

$[\alpha]_D^{20}$ –54.6 (*c* 1.0, $CHCl_3$).

IR (ATR) ν 3375 (br), 3060, 3030, 2958, 2926, 2872, 2784, 1590, 1504, 1282, 1258, 1066, 887 cm^{-1}.

^{1}H NMR (400 MHz, $CDCl_3$) δ 7.35–7.24 (5H, m, Ar<u>H</u>), 6.91 (1H, d, *J* = 1.4 Hz, Ar<u>H</u>), 6.88–6.83 (2H, m, Ar<u>H</u>), 4.48 (1H, d, *J* = 11.8 Hz, OC<u>H</u>$_a$H$_b$Ph), 4.27 (4H, s, OC<u>H</u>$_2$C<u>H</u>$_2$O), 4.24 (1H, d, *J* = 11.8 Hz, OCH$_a$<u>H</u>$_b$Ph), 4.18 (1H, d, *J* = 6.1 Hz, C<u>H</u>OBn), 3.18 (1H, ddd, *J* = 8.9, 6.1, 4.7 Hz, C<u>H</u>NH$_2$), 2.60–2.53 (2H, m, CH$_2$N(C<u>H</u>$_2$)$_4$), 2.51–2.46 (2H, m, C<u>H</u>$_2$N(CH$_2$)$_4$), 2.46–2.40 (2H, m, CH$_2$N(C<u>H</u>$_2$)$_4$), 1.77–1.70 (4H, m, N(C<u>H</u>$_2$)$_4$), 1.66 (2H, br s, CHN<u>H</u>$_2$).

^{13}C{^{1}H} NMR (100.6 MHz, $CDCl_3$) δ 143.4 (C), 143.1 (C), 138.5 (C), 132.3 (C), 128.3 (CH), 127.7 (CH), 127.4 (CH), 120.9 (CH), 117.0 (CH), 116.5 (CH), 83.4 (CH), 70.4 (CH_2), 64.3 (CH_2), 64.3 (CH_2), 59.2 (CH_2), 54.7 (CH), 54.3 (CH_2), 23.6 (CH_2).

HRMS (+ESI): *m/z* calcd. for $[M + H]^+$ $C_{22}H_{29}N_2O_3$: 369.2173; found: 369.2157.

***N*-[(1*R*,2*S*)-1-Benzyloxy-1-(2,3-dihydrobenzo[*b*][1,4]dioxin-6-yl)-3-(pyrrolidin-1-yl)propan-2-yl]octanamide (13)**

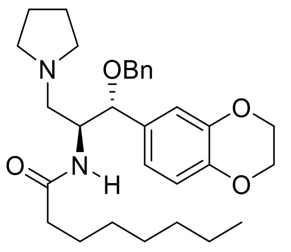

Neat Et_3N (115 μL, 0.845 mmol, 1.3 equiv) and octanoyl chloride (135 μL, 0.78 mmol, 1.2 equiv) were added at 0 °C to a solution of free amine **A** (0.65 mmol, 1.0 equiv) in CH_2Cl_2 (8 mL) at 0 °C under N_2 atmosphere, and the reaction mixture was stirred at 0 °C for 30 min and at room temperature for 16 h.

The crude solution was carefully quenched with H_2O (10 mL), and the aqueous layer was extracted with CH_2Cl_2 (3 × 10 mL). Then, the combined organic extracts were dried over $MgSO_4$ and concentrated *in vacuo*. Finally, the mixture was purified via column chromatography (95:5 CH_2Cl_2/MeOH) to give 252 mg (0.51 mmol, 78% yield) of the acylated product **13**.

Orange solid.
Mp 90–95 °C.
R_f 0.30 (95:5 CH_2Cl_2/MeOH).
$[\alpha]_D^{20}$ –51.5 (*c* 1.0, $CHCl_3$).
IR (ATR) ν 3308 (br), 3062, 3032, 2954, 2926, 2855, 1646, 1506, 1305, 1284, 1258, 1068, 889 cm^{-1}.
^{1}H NMR (400 MHz, $CDCl_3$) δ 7.36–7.27 (5H, m, Ar$\underline{H}$), 6.92 (1H, d, *J* = 1.7 Hz, Ar$\underline{H}$), 6.89–6.84 (2H, m, Ar$\underline{H}$), 5.85 (1H, d, *J* = 7.8 Hz, CHN$\underline{H}$CO), 4.70 (1H, d, *J* = 3.5 Hz, C$\underline{H}$OBn), 4.59 (1H, d, *J* = 11.9 Hz, OC$\underline{H}_a$$H_b$Ph), 4.28 (1H, d, *J* = 11.9 Hz, OC$H_a$$\underline{H}_b$Ph), 4.28 (4H, s, OC$\underline{H}_2C\underline{H}_2$O), 4.21 (1H, dddd, *J* = 10.0, 8.3, 4.9, 3.5 Hz, C$\underline{H}$NHCO), 2.84 (1H, dd, *J* = 12.4, 10.0 Hz, C$\underline{H}_a$$H_bN(CH_2)_4$), 2.44 (2H, dqd, *J* = 8.5, 5.4, 2.2 Hz, N(C$\underline{H}_2$)$_4$), 2.32 (2H, dtd, *J* = 10.0, 6.7, 5.5, 3.0 Hz, N(C$\underline{H}_2$)$_4$), 2.23 (1H, dd, *J* = 12.4, 4.9 Hz, C$H_a$$\underline{H}_bN(CH_2)_4$), 2.11–2.07 (2H, m, NHCO(C$\underline{H}_2$)$_6$$CH_3$), 1.67 (4H, td, *J* = 4.2, 2.0 Hz, N(C$\underline{H}_2$)$_4$), 1.60–1.51 (2H, m, NHCO(C$\underline{H}_2$)$_6$$CH_3$), 1.31–1.22 (8H, m, NHCO(C$\underline{H}_2$)$_6$$CH_3$), 0.89–0.85 (3H, m, NHCO$(CH_2)_6$C$\underline{H}_3$).
^{13}C{^{1}H} NMR (100.6 MHz, $CDCl_3$) δ 173.1 (C), 143.4 (C), 142.9 (C), 138.4 (C), 131.9 (C), 128.3 (CH), 127.6 (CH), 127.5 (CH), 119.9 (CH), 117.1 (CH), 115.6 (CH), 80.5 (CH_2), 71.3 (CH_2), 64.3 (CH_2), 64.3 (CH_2), 53.9 (CH_2), 53.1 (CH_2), 53.1 (CH), 36.9 (CH_2), 31.7 (CH_2), 29.1 (CH_2), 29.0 (CH_2), 25.7 (CH_2), 23.4 (CH_2), 22.6 (CH_2), 14.0 (CH_3).
HRMS (+ESI): *m/z* calcd. for $[M + H]^+$ $C_{30}H_{43}N_2O_4$: 495.3217; found: 495.3205.

N-[(1R,2S)-1-(2,3-Dihydrobenzo[b][1,4]dioxin-6-yl)-1-hydroxy-3-(pyrrolidin-1-yl)propan-2-yl]octanamide, "C2-*epi*-eliglustat" (2)

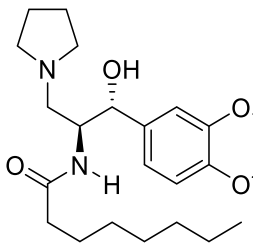

Neat powder catalyst 10% Pd/C (215 mg, 100 mol%) was added to a solution of the acylated product **13** (215 mg, 0.43 mmol, 1.0 equiv) in MeOH/10% CH_3COOH (14 mL) under bath oil at 60 °C, and the dark mixture was stirred at 60 °C for 16 h under H_2 atmosphere.

The reaction mixture was cooled down and carefully purged with N_2 until half of the initial volume before filtering the crude through a little path of celite and washing it with clean MeOH (3 × 10 mL). The filtrate was evaporated under reduced pressure and the residue was partitioned with EtOAc (10 mL) and H_2O (10 mL). The aqueous layer was extracted with EtOAc (2 × 10 mL) and the combined organic extracts were dried over $MgSO_4$ and concentrated again. The crude was finally purified via column chromatography (90:10 CH_2Cl_2/MeOH) to give 139 mg (0.34 mmol, 80% yield) of alcohol **2**, also known as **C2-*epi*-eliglustat**.

Yellowish thick wax
R_f 0.45 (90:10 CH_2Cl_2/MeOH).
$[\alpha]_D^{20}$ −10.1 (*c* 1.0, $CHCl_3$).
IR (ATR) ν 3278 (br), 3068, 2954, 2924, 2855, 2816, 1638, 1506, 1282, 1258, 1068, 887 cm^{-1}.
^{1}H NMR (400 MHz, $CDCl_3$) δ 6.86–6.84 (1H, m, Ar$\underline{H}$), 6.81–6.77 (2H, m, Ar$\underline{H}$), 6.13 (1H, br s, CHN$\underline{H}$CO), 4.69 (1H, d, *J* = 4.7 Hz, C$\underline{H}$OH), 4.21 (4H, s, $OC\underline{H}_2C\underline{H}_2O$), 4.07 (1H, dq, *J* = 6.9, 5.0 Hz, C$\underline{H}$NHCO), 2.69 (1H, dd, *J* = 12.7, 5.0 Hz, $NC\underline{H}_aH_bCH$), 2.64 (1H, dd, *J* = 12.7, 5.9 Hz, $NCH_a\underline{H}_bCH$), 2.62–2.49 (4H, m, $NC\underline{H}_2(CH_2)_2C\underline{H}_2$), 2.10 (2H, td, *J* = 7.4, 1.2 Hz, $NHCO(C\underline{H}_2)_6CH_3$), 1.78–1.68 (4H, m, $NCH_2(C\underline{H}_2)_2CH_2$), 1.53 (2H, p, *J* = 7.4 Hz, $NHCO(C\underline{H}_2)_6CH_3$), 1.28–1.16 (8H, m, $NHCO(C\underline{H}_2)_6CH_3$), 0.85 (3H, t, *J* = 6.8 Hz, $NHCO(CH_2)_6C\underline{H}_3$).
$^{13}C\{^1H\}$ NMR (100.6 MHz, $CDCl_3$) δ 173.4 (C), 143.3 (C), 142.7 (C), 135.0 (C), 119.0 (CH), 116.9 (CH), 115.1 (CH), 77.2 (CH), 64.2 (CH_2), 56.6 (CH_2), 54.7 (CH_2), 53.0 (CH), 36.6 (CH_2), 31.6 (CH_2), 29.1 (CH_2), 28.9 (CH_2), 25.6 (CH_2), 23.5 (CH_2), 22.5 (CH_2), 14.0 (CH_3).
HRMS (+ESI): *m/z* calcd. for $[M + H]^+$ $C_{23}H_{37}N_2O_4$: 405.2748; found: 405.2738.

4. Total Synthesis of Eliglustat

1,3-Oxazolidine-2-thione

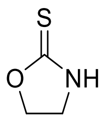

Anhydrous Et_3N (5.6 mL, 40 mmol, 1 equiv) was added dropwise to a solution of 2-aminoethanol (mL, 40 mmol, 1 equiv) in absolute methanol (40 mL) under a nitrogen atmosphere, and the mixture was then cooled to 0 °C. After complete the dropwise addition of CS_2 (3.6 mL, 60 mmol, 1.5 equiv), the mixture was stirred at 0 °C for 30 min, and at room temperature for another 30 min.

The resulting pale-yellow solution was then quenched with 30% (v/v) H_2O_2 (7 mL), until no more white solid is formed while its addition, and the yellow suspension was stirred for 30 min. Then, the mixture was filtered and concentrated under reduced pressure using a peroxide tramp, followed by the addition of 2 M NaOH (20 mL) and 2 M HCl (ca. 30 mL) to acidify the solution to pH 1.

The resulting bright yellow solution was extracted with CH_2Cl_2 (3 × 25 mL), and the combined organic layers were dried over $MgSO_4$, filtered, and concentrated *in vacuo*. The solid obtained was purified by recrystallization (cyclohexenes/CH_2Cl_2) to yield a white crystalline powder.

White solid.
Mp 98–100 °C .
R_f 0.15 (60:40 Hexanes/EtOAc).
IR (ATR) ν 3203, 2923, 1520, 1456, 1398, 1312, 1284, 1232, 1203, 1162, 1033 cm^{-1}.
^{1}H NMR (400 MHz, $CDCl_3$) δ 8.19 (1H, br s, N<u>H</u>), 4.73 (2H, t,*J* = 8.8 Hz, OC<u>H</u>$_2$), 3.85 (2H, t, *J* = 8.8 Hz, NHC<u>H</u>$_2$).
^{13}C{^{1}H} NMR (100.6 MHz, $CDCl_3$) δ 190.1 (C), 70.4 (CH_2), 44.1 (CH_2).
HRMS (+ESI): *m/z* calcd. for $[M + H]^+$ C_3H_6NOS: 104.0165; found 104.0167.

_N_-(2-Azidoacetyl)-1,3-oxazolidine-2-thione (7)

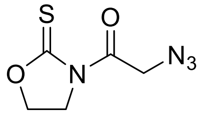

Neat 2-bromoacetic acid (4.6 g, 33.0 mmol, 1.0 equiv) in distilled H_2O (25 mL, 1.3 M) was added dropwise to an aqueous solution (22 mL, 3M) of sodium azide (4.3 g, 66.0 mmol, 2 equiv) at 0 °C. After 15 min, the ice bath was removed and the reaction mixture was stirred overnight at room temperature.

The reaction was then slowly acidified with 2 M HCl until pH 1 and the aqueous solution was extracted with Et_2O (3 × 30 mL). The combined organic extracts were dried over $NaSO_4$, filtered, and the solvent was removed under reduced pressure to afford 2-azidoacetic acid (99% yield) as a colourless oil which was used in the next step without further purification.

The formed 2-azidoacetic acid (3.3 g, 33.0 mmol, 1.1 equiv) in CH_2Cl_2 (33 mL, 1 M) was added dropwise to a solution of 1,3-oxazolidine-2-thione (3.1 g, 30.0 mmol, 1 equiv), EDC·HCl (6.9 g, 36.0 mmol, 1.2 equiv), and DMAP (185 mg, 1.5 mmol, 0.05 equiv) in CH_2Cl_2 (60 mL, 0.5 M) at 0 °C and stirred for 15 min. The solution was then allowed to reach room temperature and was stirred overnight.

The reaction mixture was washed with deionized H_2O and brine. The organic layers were combined, dried over $NaSO_4$ and filtered. After removing the solvent *in vacuo*, the crude product was purified by flash column chromatography (85:15 Hexanes/EtOAc) to afford **7** (3.75 g, 20.1 mmol, 67% yield) as a white solid.

White solid.
Mp 32–34 °C
R_f 0.25 (85:15 Hexanes/EtOAc).
IR (ATR) ν 2917, 2100, 1697, 1364, 1320, 1212, 1168, 1017 cm^{-1}.
^{1}H NMR (400 MHz, $CDCl_3$) δ 4.91 (2H, s, $C\underline{H_2}N_3$), 4.65 (2H, t, *J* = 8.5 Hz, $OC\underline{H_2}$), 4.29 (2H, t, *J* = 8.5 Hz, $NC\underline{H_2}$).
^{13}C{^{1}H} NMR (100.6 MHz, $CDCl_3$) δ 184.9 (C), 169.0 (C), 67.5 (CH_2), 54.5 (CH_2), 46.9 (CH_2).
HRMS (+ESI): *m/z* calcd. for $[M + H]^+$ $C_5H_7N_4O_2S$: 187.0284; found: 187.0286. *m/z* calcd. for $[M + Na]^+$ $C_5H_6N_4NaO_2S$: 209.0104; found: 209.0105.

Compound **7** has also been reported elsewhere. [1][2][3]

***N*-[(2*S*,3*R*)-2-Azido-3-(benzyloxy)-3-(2,3-dihydrobenzo[*b*][1,4]dioxin-6-yl)propanoyl]-1,3-oxazinane-2-thione (14)**

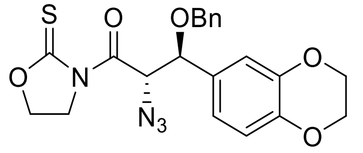

A solution of *N*-(2-azidoacetyl)-1,3-oxazolidine-2-thione (**7**, 93 mg, 0.50 mmol, 1.0 equiv), 6-(bis(benzyloxy)methyl)-2,3-dihydrobenzo[b][1,4]dioxine (**4**, 200 mg, 0.55 mmol, 1.1 equiv), and [(*R*)-DTBM-SEGPHOS]$NiCl_2$ (32.6 mg, 25 µmol, 5 mol%) in CH_2Cl_2 (2 mL, 0.25 M) was cooled at –20 °C under a N_2 athmosphere. Then, neat TMSOTf (120 µL, 0.65 mmol, 1.3 equiv) was added dropwise to the stirring green-brown solution followed by 2,6-lutidine (90 µL, 0.75 mmol, 1.5 equiv) addition, and the resultant dark green mixture was stirred at –20 °C for 5 h.

The mixture was quenched with sat NH_4Cl (2 mL) and partitioned in CH_2Cl_2 (15 mL) and water (15 mL). The aqueous layer was then extracted with neat CH_2Cl_2 (2 × 15 mL), and the combined organic extracts were dried over Na_2SO_4 and concentrated *in vacuo*. The residue (dr 70:30) was purified by column chromatography (80:20 Hexanes/EtOAc) to afford 132 mg (0.30 mmol, 60% yield) of *anti* adduct **14** and 58 mg (0.13 mmol, 26% yield) of the *syn* diastereomer.

Yellowish oil.

R_f 0.20 (80:20 Hexanes/EtOAc).

Chiral HPLC (Phenomenex Lux® Cellulose-1 column, 25% *i*-PrOH in hexane, flow rate 1.0 mL·min^{-1}): Rt 36.7 min (Major 2*S*,3*R*-isomer) [Rt 24.9 min (minor 2*R*,3*S*-isomer)], 99% ee.

$[\alpha]_D^{20}$ +41.4 (*c* 1.00, $CHCl_3$).

IR (ATR) ν 2926, 2108, 1702, 1506, 1368, 1288, 1206, 1066, 889 cm^{-1}.

^{1}H NMR (400 MHz, $CDCl_3$) δ 7.33–7.25 (3H, m, Ar$\underline{H}$), 7.22–7.20 (2H, m, Ar$\underline{H}$), 7.02 (1H, d, *J* = 2.0 Hz, Ar$\underline{H}$), 6.97 (1H, dd, *J* = 8.2, 2.0 Hz, Ar$\underline{H}$), 6.92 (1H, d, *J* = 8.2 Hz, Ar$\underline{H}$), 6.54 (1H, d, *J* = 8.9 Hz, C$\underline{H}N_3$), 4.67 (1H, d, *J* = 8.9 Hz, C$\underline{H}OCH_3$), 4.52 (1H, td, *J* = 8.9, 5.7 Hz, OC$\underline{H_a}H_b$), 4.46 (1H, d, *J* = 11.4 Hz, OC$\underline{H_a}H_b$Ar), 4.31–4.26 (1H, m, OC$H_a\underline{H_b}$), 4.29 (4H, s, O$C\underline{H_2}C\underline{H_2}$O), 4.24–4.18 (2H, m, OC$H_a\underline{H_b}$Ar & NC$\underline{H_a}H_b$), 4.08 (1H, ddd, *J* = 11.0, 9.0, 5.7 Hz, NC$H_a\underline{H_b}$).

$^{13}C\{^1H\}$ NMR (100.6 MHz, $CDCl_3$) δ 185.3 (C), 170.6 (C), 144.3 (C), 143.8 (C), 137.6 (C), 130.0 (C), 128.3 (CH), 127.7 (CH), 127.6 (CH), 121.0 (CH), 117.7 (CH), 116.7 (CH), 81.7 (CH), 70.8 (CH_2), 66.6 (CH_2), 64.3 (CH_2), 64.3 (CH_2), 62.3 (CH), 47.2 (CH_2).

HRMS (+ESI): *m/z* calcd. for $[M - OBn]^+$ $C_{14}H_{13}N_4O_4S$: 333.0652; found: 333.0657. *m/z* calcd. for $[M + Na]^+$ $C_{21}H_{20}N_4NaO_5S$: 463.1047; found: 463.1059.

***N*-[(2*S*,3*R*)-2-Azido-3-benzyloxy-3-(3,4-bis((*tert*-butylsilyl)oxy)phenyl)propanoyl]-1,3-oxazinane-2-thione (16)**

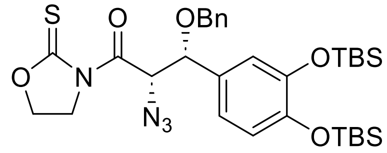

A solution of *N*-(2-azidoacetyl)-1,3-oxazolidine-2-thione (**7**, 47 mg, 0.25 mmol, 1.0 equiv), 3,4-bis((*tert*-butyldimethylsilyl)oxy)benzaldehyde dibenzyl acetal (**15**, 142 mg, 0.28 mmol, 1.1 equiv), and [(*R*)-DTBM-SEGPHOS]$NiCl_2$ (16.4 mg, 12.5 µmol, 5 mol%) in CH_2Cl_2 (1 mL, 0.25 M) was cooled at –20 °C under a N_2 athmosphere. Then, neat TMSOTf (115 µL, 0.63 mmol, 2.5 equiv) was added dropwise to the stirring green-brown solution followed by 2,6-lutidine (45 µL, 0.38 mmol, 1.5 equiv) addition, and the resultant dark green mixture was stirred at –20 °C for 3 h.

The mixture was quenched with sat NH_4Cl (2 mL) and partitioned in CH_2Cl_2 (15 mL) and water (15 mL). The aqueous layer was then extracted with neat CH_2Cl_2 (2 × 15 mL), and the combined organic extracts were dried over Na_2SO_4 and concentrated *in vacuo*. The residue (dr 21:79) was purified by column chromatography (from 90:10 to 70:30 Hexanes/EtOAc) to afford 113 mg (0.18 mmol, 70% yield) of *syn* adduct **16** and 27 mg (4 µmol, 17% yield) of the *anti* diastereomer.

The reaction was also carried out up to 1.5 mmol scale (dr 22:78) to afford 640 mg (66% yield) of the proper *syn* adduct.

Pale orange oil.
R_f 0.10 (70:30 Hexanes/EtOAc).
Chiral HPLC (Phenomenex Lux® Cellulose-1 column, 20% *i*-PrOH in hexane, flow rate 1.0 mL·min^{-1}): Rt 8.4 min (Major 2*S*,3*R*-isomer) [Rt 25.8 min (minor 2*R*,3*S*-isomer)], 99% ee.
$[\alpha]_D^{20}$ –100.5 (*c* 1.0, $CHCl_3$).
IR (ATR) ν 2954, 2928, 2857, 2110, 1707, 1508, 1377, 1327, 1295, 1254, 1210, 1167, 841, 781 cm^{-1}.
^{1}H NMR (400 MHz, $CDCl_3$) δ 7.37–7.26 (5H, m, ArH), 7.03 (1H, d, *J* = 2.2 Hz, ArH), 6.97 (1H, ddd, *J* = 8.2, 2.2, 0.5 Hz, ArH), 6.85 (1H, d, *J* = 8.20 Hz, ArH), 6.11 (1H, d, *J* = 4.5 Hz, CHN$_3$), 5.05 (1H, d, *J* = 4.5 Hz, CHOBn), 4.62 (1H, d, *J* = 12.2 Hz, OCH_aH_bPh), 4.47–4.43 (1H, m, OCH_aH_b), 4.18 (1H, d, *J* = 12.2 Hz, OCH_aH_bPh), 4.16–4.05 (2H, m, OCH_aH_b & NCH_aH_b), 3.79–3.74 (1H, m, NCH_aH_b), 1.00 (9H, s, SiC(CH_3)$_3$), 1.00 (9H, s, SiC(CH_3)$_3$), 0.23–0.22 (12H, m, Si(CH_3)$_2$).
^{13}C{^{1}H} NMR (100.6 MHz, $CDCl_3$) δ 184.7 (C), 169.3 (C), 147.2 (C), 147.0 (C), 137.5 (C), 129.3 (C), 128.4 (CH), 128.4 (CH), 127.9 (CH), 120.9 (CH), 120.5 (CH), 119.9 (CH), 79.2 (CH), 70.3 (CH_2), 66.6 (CH_2), 65.5 (CH), 47.3 (CH_2), 25.9 (CH_3), 25.9 (CH_3), 18.5 (C), 18.4 (C), -4.0 (CH_2), -4.1 (CH_2).
HRMS (+ESI): *m/z* calcd. for [M – OBn]$^+$ $C_{24}H_{39}N_4O_4SSi_2$: 535.2225; found: 535.2210. *m/z* calcd. for [M + Na]$^+$ $C_{31}H_{46}N_4NaO_5SSi_2$: 665.2620; found: 665.2602.

(2*R*,3*R*)-2-Azido-3-benzyloxy-3-[3,4-bis{(*tert*-butyldimethylsilyl)oxy}phenyl]propan-1-ol (17)

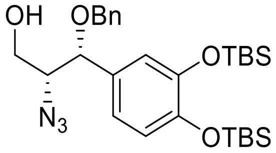

A 2 M solution of $LiBH_4$ in THF (150 µL, 0.30 mmol, 2.0 equiv) was added to a solution of adduct **16** (100 mg, 0.15 mmol, 1.0 equiv) in CH_2Cl_2 (1.5 mL) at 0 °C under N_2 atmosphere and the reaction mixture was stirred at 0 °C for 3 h.

The reaction mixture was carefully quenched with MeOH (2 mL) and the volatiles were removed under vacuum. The residue was then partitioned with CH_2Cl_2 (15 mL) and 2 M NaOH (15 mL), and the aqueous layer was extracted with clean CH_2Cl_2 (2 × 15 mL). The combined organic extracts were dried over $MgSO_4$ and concentrated under vacuum, and the crude mixture was analysed and used in the next step without further purification.

The reaction was also carried out up to 0.46 mmol scale.

Colorless oil.
R$_f$ 0.30 (90:10 Hexanes/EtOAc).
$[\alpha]_D^{20}$ –53.9 (*c* 1.0, $CHCl_3$).
IR (ATR) ν 3377 (br), 2928, 2857, 2097, 1508, 1292, 1252, 904, 837, 781 cm^{-1}.
^{1}H NMR (400 MHz, $CDCl_3$) δ 7.37–7.27 (5H, m, ArH), 6.85–6.83 (2H, m, ArH), 6.78 (1H, dd, *J* = 8.2, 2.1 Hz, ArH), 4.51 (1H, d, *J* = 11.7 Hz, OCH_aH_bPh), 4.37 (1H, d, *J* = 7.8 Hz, CHOBn), 4.30 (1H, d, *J* = 11.7 Hz, OCH_aH_bPh), 3.65 (1H, ddd, *J* = 7.8, 6.2, 3.7 Hz, CHN_3), 3.41 (1H, ddd, *J* = 10.5, 6.4, 3.7 Hz, CH_aH_bOH), 3.29 (1H, dt, *J* = 11.5, 5.7 Hz, CH_aH_bOH), 1.73 (1H, br s, OH), 1.00 (9H, s, $SiC(CH_3)_3$), 0.99 (9H, s, $SiC(CH_3)_3$), 0.22–0.20 (12H, m, $Si(CH_3)_2$).
^{13}C{^{1}H} NMR (100.6 MHz, $CDCl_3$) δ 147.3 (C), 147.2 (C), 137.6 (C), 130.6 (C), 128.4 (CH), 128.0 (CH), 127.7 (CH), 121.2 (CH), 120.6 (CH), 120.0 (CH), 81.4 (CH), 70.4 (CH_2), 68.3 (CH), 62.2 (CH_2), 25.9 (CH_3), 18.5 (C), -4.0 (CH_3), -4.1 (CH_3), -4.1 (CH_3).
HRMS (+ESI): *m/z* calcd. for $[M - OBn - N_2 + H]^+$ $C_{21}H_{40}NO_3Si_2$: 410.2541; found: 410.2527. *m/z* calcd. for $[M - N_2 + H]^+$ $C_{28}H_{46}NO_4Si_2$: 516.2960; found: 516.2943. *m/z* calcd. for $[M + Na]^+$ $C_{28}H_{45}N_3NaO_4Si_2$: 566.2841; found: 566.2820.

4-[(1*R*,2*R*)-2-Azido-1-benzyloxy-3-hydroxypropyl]benzene-1,2-diol (18)

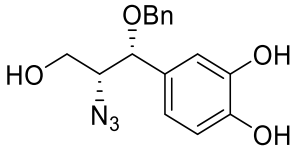

A solution (~1.1 mmol/g of resin) of TBAF in silica (550 mg, 0.60 mmol, 4.0 equiv) in THF (3 mL) was added dropwise to a solution of alcohol **17** (0.15 mmol, 1.0 equiv) in THF (4 mL) at room temperature under N_2 atmosphere, and the reaction mixture was then stirred for 1,5 h.

The volatiles of the mixture were removed under vacuum and the resulting crude was diluted with EtOAc (15mL) and washed with brine (10 mL). The combined organic layers were dried over $MgSO_4$ and concentrated in vacuo, and the resultant residue was purified via column chromatography (60:40 Hexanes/EtOAc) to give 42 mg (0.13 mmol, 88% yield) of product **18.**

The reaction was also carried out up to 0.46 mmol scale to afford 125 mg (85% yield) of the proper *syn* adduct.

Orange-brown oil.
R_f 0.20 (60:40 Hexanes/EtOAc).
$[\alpha]_D^{20}$ –75.6 (*c* 1.0, $CHCl_3$).
IR (ATR) ν 3336 (br), 3064, 3032, 2924, 2853, 2102, 1608, 1519, 1452, 1280, 1074, 1038, 753 cm^{-1}.
^{1}H NMR (400 MHz, $CDCl_3$) δ 7.36–7.27 (5H, m, ArH), 6.87–6.84 (2H, m, ArH), 6.76 (1H, dd, *J* = 8.2, 1.8 Hz, ArH), 4.49 (1H, d, *J* = 11.7 Hz, OCH_aH_bPh), 4.36 (1H, d, *J* = 8.0 Hz, CHOBn), 4.30 (1H, d, *J* = 11.7 Hz, OCH_aH_bPh), 3.66 (1H, ddd, *J* = 8.0, 6.0, 3.6 Hz, CHN_3), 3.42 (1H, dd, *J* = 11.7, 3.6 Hz, CH_aH_bOH), 3.29 (1H, dd, *J* = 11.7, 5.9 Hz, CH_aH_bOH).
$^{13}C\{^1H\}$ NMR (100.6 MHz, $CDCl_3$) δ 144.3 (C), 144.1 (C), 137.5 (C), 130.2 (C), 128.4 (CH), 127.9 (CH), 127.8 (CH), 120.2 (CH), 115.5 (CH), 114.1 (CH), 81.4 (CH), 70.4 (CH_2), 68.1 (CH), 62.1 (CH_2).
HRMS (+ESI): *m/z* calcd. for $[M + Na]^+$ $C_{16}H_{17}N_3NaO_4$: 338.1111; found: 338.1099.
HRMS (–ESI): *m/z* calcd. for $[M – H]^-$ $C_{16}H_{16}N_3O_4$: 314.1146; found: 314.1144.

(2*R*,3*R*)-2-Azido-3-benzyloxy-3-(2,3-dihydrobenzo[*b*][1,4]dioxin-6-yl)propan-1-ol (19)

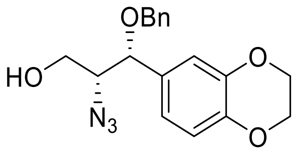

Diphenol **18** (31.5 mg, 0.10 mmol, 1.0 equiv) and powder NaOH (14 mg, 0.35 mmol, 3.5 equiv) were dissolved in toluene (2 mL) and THF (0.5 mL) under N_2 atmosphere in an oil bath at 40 °C. Then, neat ethyleneglycol bistriflate (40 mg, 0.12 mmol, 1.2 equiv) in toluene (1 mL) and *N*-methyl-*N*,*N*,*N*-trioctylammonium chloride (2 mg, 5 μmol, 5 mol%) in toluene (1 mL) were added dropwise to the initial solution and the reaction mixture was then stirred at 40 °C for 16 h.

After reaching room temperature, the reaction mixture was diluted with Et_2O (10 mL) and washed with H_2O (10 mL). The aqueous phase was extracted with Et_2O (2 × 10 mL), the combined organic layers were dried over $MgSO_4$ and concentrated *in vacuo*, and the resultant residue was purified via column chromatography (70:30 Hexanes/EtOAc) to give 17 mg (50 μmol, 50% yield) of fused ring compound **19**.

The reaction was also carried out at 0.33 mmol scale to afford 40 mg (117 μmol, 36% yield) of the proper *syn* adduct **19**.

Colorless oil.
R_f 0.30 (80:20 Hexanes/EtOAc).
$[\alpha]_D^{20}$ –64.2 (*c* 1.0, $CHCl_3$).
IR (ATR) ν 3366 (br), 2924, 2872, 2855, 2097, 1506, 1282, 1258, 1064, 1049, 885, 740 cm^{-1}.
^{1}H NMR (400 MHz, $CDCl_3$) δ 7.37–7.27 (5H, m, Ar<u>H</u>), 6.89–6.87 (2H, m, Ar<u>H</u>), 6.82 (1H, ddd, *J* = 8.2, 2.0, 0.4 Hz, Ar<u>H</u>), 4.51 (1H, d, *J* = 11.8 Hz, C$\underline{H}_a$$H_b$Ph), 4.39 (1H, d, *J* = 7.8 Hz, C<u>H</u>OBn), 4.30 (1H, d , *J* = 11.8 Hz, OC$H_a$$\underline{H}_b$Ph), 4.28 (4H, s, OC$\underline{H_2}C\underline{H_2}$O), 3.66 (1H, ddd, *J* = 7.8, 6.2, 3.7 Hz, C<u>H</u>N_3), 3.43 (1H, ddd, *J* = 11.6, 6.6, 3.7 Hz, C$\underline{H}_a$$H_b$OH), 3.30 (1H, dt, *J* = 11.8, 6.0 Hz, C$H_a$$\underline{H}_b$OH), 1.71 (1H, t, *J* = 6.4 Hz, O<u>H</u>).
^{13}C{^{1}H} NMR (100.6 MHz, $CDCl_3$) δ 143.8 (C), 143.8 (C), 137.6 (C), 130.9 (C), 128.4 (CH), 127.9 (CH), 127.7 (CH), 120.4 (CH), 117.6 (CH), 116.2 (CH), 81.3 (CH), 70.4 (CH_2), 68.2 (CH), 64.3 (CH_2), 62.1 (CH_2).
HRMS (+ESI): *m/z* calcd. for $[M + Na]^+$ $C_{18}H_{19}N_3NaO_4$: 364.1268; found: 364.1253.

1-[(2*R*,3*R*)-2-Azido-3-benzyloxy-3-(2,3-dihydrobenzo[*b*][1,4]dioxin-6-yl)propyl]methanesulfonate (B)

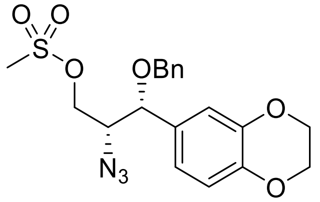

Neat mesyl chloride (35 µL, 0.45 mmol, 2.0 equiv) and pyridine (37 µL, 0.45 mmol, 2.0 equiv) were slowly added to a solution of residue **19** (77 mg, 0.22 mmol, 1.0 equiv) in CH_2Cl_2 (3 mL) at 0 °C under N_2 atmosphere. The reaction mixture was then stirred at room temperature until complete consumption of the initial alcohol.

After 4 h, the crude was quenched with H_2O (10 mL) and the resulting mixture was extracted with CH_2Cl_2 (3 × 5 mL). The combined organic layers were dried over $MgSO_4$ and concentrated *in vacuo*, and the resulting residue **B** was analyzed and used in the next step without further purification.

The reaction was also carried out at 0.65 mmol scale.

Colorless oil.
R$_f$ 0.30 (10:90 Hexanes/CH_2Cl_2).
[α]$_D^{20}$ –28.1 (*c* 1.0, $CHCl_3$).
IR (ATR) ν 2954, 2922, 2853, 2102, 1508, 1357, 1284, 1258, 1174, 1066, 813 cm^{-1}.
^{1}H NMR (400 MHz, $CDCl_3$) δ 7.37–7.27 (5H, m, ArH), 6.90 (1H, d, *J* = 8.2 Hz, ArH), 6.88 (1H, d, *J* = 2.1 Hz, ArH), 6.82 (1H, dd, *J* = 8.2, 2.1 Hz, ArH), 4.53 (1H, d, *J* = 11.6 Hz, CH_aH_bPh), 4.38 (1H, d, *J* = 7.3 Hz, CHOBn), 4.31 (1H, d, *J* = 11.6 Hz, CH_aH_bPh), 4.29 (4H, s, OCH_2CH_2O), 4.10 (1H, dd, *J* = 10.6, 3.2 Hz, $CH_aH_bOSO_2CH_3$), 3.91 (1H, dd, *J* = 10.6, 6.7 Hz, $CH_aH_bOSO_2CH_3$), 3.82 (1H, ddd, *J* = 7.3, 6.7, 3.2 Hz, CHN_3), 3.01 (3H, s, OSO_2CH_3).
^{13}C{^{1}H} NMR (100.6 MHz, $CDCl_3$) δ 144.2 (C), 144.0 (C), 137.2 (C), 129.8 (C), 128.5 (CH), 128.0 (CH), 127.9 (CH), 120.4 (CH), 117.8 (CH), 116.1 (CH), 80.2 (CH), 70.6 (CH_2), 68.4 (CH_2), 64.9 (CH), 64.3 (CH_2), 37.6 (CH_3).
HRMS (+ESI): *m/z* calcd. for [M – OBn – Ms – N_2 + H]$^+$ $C_{11}H_{14}NO_3$: 208.0968; found: 208.0963. *m/z* calcd. for [M + Na]$^+$ $C_{19}H_{21}N_3NaO_6S$: 442.1043; found: 442.1032.

1-[(2*R*,3*R*)-2-Azido-3-benzyloxy-3-(2,3-dihydrobenzo[*b*][1,4]dioxin-6-yl)propyl]pyrrolidine (10)

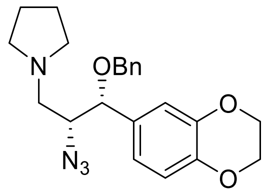

Neat pyrrolidine (75 µL, 0.90 mmol, 4.0 equiv) was added to a solution of the mesylate **B** (0.22 mmol, 1.0 equiv.) in DMF (3 mL) in an oil bath at 60 °C under N_2 atmosphere and the reaction mixture was stirred at 60 °C for 16 h.

After stirring overnight, the reaction was cooled down to room temperature and a kind of precipitate was observed when quenching the crude with H_2O (10 mL). The mixture was then extracted with EtOAc (2 × 10 mL), and the combined organic extracts were washed with brine (10 mL), dried over $MgSO_4$ and concentrated under reduced pressure. The crude was finally purified via column chromatography (70:30 CH_2Cl_2/EtOAc) to afford 69 mg (0.17 mmol, 80% yield) of tertiary amine **10**.

The reaction was also carried out at 0.65 mmol scale to afford 215 mg (0.54 mmol, 84% yield) of the proper *syn* adduct **10**.

Orange-brown oil.
R_f 0.30 (70:30 CH_2Cl_2/EtOAc).
$[\alpha]_D^{20}$ –32.6 (*c* 0.5, $CHCl_3$).
IR (ATR) ν 2956, 2924, 2872, 2853, 2794, 2097, 1506, 1457, 1284, 1258, 1068, 887 cm^{-1}.
^{1}H NMR (400 MHz, $CDCl_3$) δ 7.36–7.26 (5H, m, Ar<u>H</u>), 6.88–6.86 (2H, m, Ar<u>H</u>), 6.81 (1H, dd, *J* = 8.4, 2.0 Hz, Ar<u>H</u>), 4.52 (1H, d, *J* = 11.5 Hz, C<u>H</u>$_a$H$_b$Ph), 4.31 (1H, d, *J* = 6.4 Hz, C<u>H</u>OBn), 4.30 (1H, d, *J* = 11.5 Hz, CH$_a$<u>H</u>$_b$Ph), 4.28 (4H, s, OC<u>H</u>$_2$C<u>H</u>$_2$O), 3.66–3.61 (1H, m, C<u>H</u>N$_3$), 2.55 (1H, dd, *J* = 12.6, 9.1 Hz, CH$_a$<u>H</u>$_b$CHN$_3$), 2.57–2.49 (2H, m, NC<u>H</u>$_2$(CH$_2$)$_2$C<u>H</u>$_2$), 2.49–2.41 (2H, m, NC<u>H</u>$_2$(CH$_2$)$_2$C<u>H</u>$_2$), 2.32 (1H, dd, *J* = 12.6, 3.5 Hz, C<u>H</u>$_a$H$_b$CHN$_3$), 1.78–1.73 (4H, m, NCH$_2$(C<u>H</u>$_2$)$_2$CH$_2$).
^{13}C{^{1}H} NMR (100.6 MHz, $CDCl_3$) δ 143.6 (C), 143.6 (C), 137.9 (C), 131.8 (C), 128.3 (CH), 127.7 (CH), 127.6 (CH), 120.5 (CH), 117.4 (CH), 116.3 (CH), 82.0 (CH), 70.5 (CH_2), 65.8 (CH), 64.3 (CH_2), 56.4 (CH_2), 54.3 (CH_2), 23.5 (CH_2).
HRMS (+ESI): *m/z* calcd. for $[M - CH_2CH_2 + 3H]^+$ $C_{20}H_{25}N_4O_3$: 369.1921; found: 369.1910. *m/z* calcd. for $[M + H]^+$ $C_{22}H_{27}N_4O_3$: 395.2078; found: 395.2060.

(1*R*,2*R*)-1-Benzyloxy-1-(2,3-dihydrobenzo[*b*][1,4]dioxin-6-yl)-3-(pyrrolidin-1-yl)propan-2-amine (C)

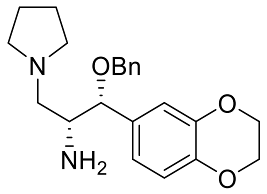

A 1 M solution of Me_3P in THF (190 μL, 0.19 mmol, 1.1 equiv) was added to a solution of tertiary amine **10** (69 mg, 0.17 mmol, 1.0 equiv) in THF (2 mL) and H_2O (50 μL) at 0 °C under N_2 atmosphere, and the reaction was stirred at 0 °C for 30 min and at room temperature for 16 h.

The mixture was diluted with CH_2Cl_2 (8 mL) and the crude was washed with 0.5 M NaOH (8 mL). The aqueous layer was then extracted with clean CH_2Cl_2 (2 × 10 mL) and the resulting combined organic layers were dried over $MgSO_4$ and concentrated under *vacuum*. The crude mixture **C** was analyzed and used in the next step without further purification.

The reaction was also carried out at 0.55 mmol scale.

Brownish oil.
R_f 0.30 (90:10 CH_2Cl_2/MeOH).
$[\alpha]_D^{20}$ –20.2 (*c* 0.5, $CHCl_3$).
IR (ATR) ν 3383 (br), 2958, 2926, 2872, 1590, 1504, 1456, 1305, 1284, 1258, 1066, 887 cm^{-1}.
^{1}H NMR (400 MHz, $CDCl_3$) δ 7.35–7.26 (5H, m, Ar<u>H</u>), 6.87–6.85 (2H, m, Ar<u>H</u>), 6.79 (1H, dd, *J* = 8.2, 2.1 Hz, Ar<u>H</u>), 4.45 (1H, d, *J* = 11.5 Hz, C<u>H</u>$_a$H$_b$Ph), 4.27 (1H, d, J = 11.5 Hz, CH$_a$<u>H</u>$_b$Ph), 4.28 (4H, s, OC<u>H</u>$_2$C<u>H</u>$_2$O), 4.12 (1H, d, *J* = 6.4 Hz, C<u>H</u>OBn), 3.08 (1H, ddd, *J* = 10.1, 6.4, 4.0 Hz, C<u>H</u>NH$_2$), 2.50–2.44 (3H, m, NC<u>H</u>$_2$(CH$_2$)$_2$C<u>H</u>$_2$ & NC<u>H</u>$_a$H$_b$), 2.35–2.34 (2H, m, NC<u>H</u>$_2$(CH$_2$)$_2$C<u>H</u>$_2$), 2.04 (1H, dd, *J* = 11.8, 4.0 Hz, NCH$_a$<u>H</u>$_b$), 1.97–1.82 (2H, br m, N<u>H</u>$_2$), 1.73–1.66 (4H, m, NCH$_2$(C<u>H</u>$_2$)$_2$CH$_2$).
^{13}C{^{1}H} NMR (100.6 MHz, $CDCl_3$) δ 143.5 (C), 143.1 (C), 138.5 (C), 133.2 (C), 128.3 (CH), 127.8 (CH), 127.5 (CH), 120.5 (CH), 117.1 (CH), 116.2 (CH), 84.1 (CH), 70.5 (CH_2), 64.3 (CH_2), 64.3 (CH_2), 59.2 (CH_2), 55.6 (CH), 54.2 (CH_2), 23.5 (CH_2).
HPLC–MS (ES+): Rt = 1.96 min. *m/z* calcd. for $[M + H]^+$ $C_{22}H_{29}N_2O_3$: 369.2173; found: 369.1.

***N*-[(1*R*,2*R*)-1-Benzyloxy-1-(2,3-dihydrobenzo[*b*][1,4]dioxin-6-yl)-3-(pyrrolidin-1-yl)propan-2-yl]octanamide (20)**

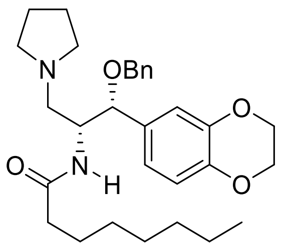

Neat Et_3N (30 µL, 0.22 mmol, 1.3 equiv) and octanoyl chloride (35 µL, 0.20 mmol, 1.2 equiv) were added at 0 °C to a solution of free amine **C** (0.17 mmol, 1.0 equiv) in CH_2Cl_2 (3 mL) at 0 °C under N_2 atmosphere, and the reaction mixture was stirred at 0 °C for 30 min and at room temperature for 16 h.

The crude solution was carefully quenched with H_2O (10 mL), and the aqueous layer was extracted with CH_2Cl_2 (3 × 10 mL). Then, the combined organic extracts were dried over $MgSO_4$ and concentrated *in vacuo*. Finally, the mixture was purified via column chromatography (95:5 CH_2Cl_2/MeOH) to give 57 mg (0.12 mmol, 68% yield) of the acylated product **20**.

The reaction was also carried out at 0.55 mmol scale to afford 186 mg (0.38 mmol, 69% yield) of the proper *syn* adduct **20**.

Brownish oil.
R_f 0.55 (95:5 CH_2Cl_2/MeOH).
$[\alpha]_D^{20}$ –10.4 (*c* 0.25, $CHCl_3$).
IR (ATR) ν 3373 (br), 2958, 2924, 2853, 1653, 1506, 1457, 1284, 1258, 1066, 1019, 800 cm^{-1}.
^1H NMR (400 MHz, $CDCl_3$) δ 7.38–7.28 (5H, m, Ar<u>H</u>), 6.82 (1H, d, *J* = 8.3 Hz, Ar<u>H</u>), 6.81 (1H, d, *J* = 2.0 Hz, Ar<u>H</u>), 6.76 (1H, dd, *J* = 8.3, 2.0 Hz, Ar<u>H</u>), 5.79 (1H, d, *J* = 8.8 Hz, CHN<u>H</u>CO), 4.76 (1H, d, *J* = 2.2 Hz, C<u>H</u>OBn), 4.55 (1H, d, *J* = 11.6 Hz, $C\underline{H}_aH_bPh$), 4.33 (1H, d, *J* = 11.6 Hz, $CH_a\underline{H}_bPh$), 4.25 (4H, s, $OC\underline{H}_2C\underline{H}_2O$), 4.15 (1H, ddd, *J* = 8.6, 6.5, 2.2 Hz, C<u>H</u>NHCO), 2.76 (1H, dd, *J* = 11.8, 8.6 Hz, $C\underline{H}_aH_bCHNHCO$), 2.55–2.36 (4H, m, $CH_2N(C\underline{H}_2)_4$), 2.38 (1H, dd, *J* = 11.8, 6.5 Hz, $CH_a\underline{H}_bCHNHCO$), 2.09 (2H, td, *J* = 7.4, 1.9 Hz, $NHCO(C\underline{H}_2)_6CH_3$), 1.74–1.70 (4H, m, $CH_2N(C\underline{H}_2)_4$), 1.52 (2H, p, *J* = 7.4 Hz, $NHCO(C\underline{H}_2)_6CH_3$), 1.30–1.20 (8H, m, $NHCO(C\underline{H}_2)_6CH_3$), 0.89–0.86 (3H, m, $NHCO(CH_2)_6C\underline{H}_3$).
$^{13}C\{^1H\}$ NMR (100.6 MHz, $CDCl_3$) δ 172.8 (C), 143.4 (C), 143.0 (C), 138.3 (C), 132.7 (C), 128.3 (CH), 127.9 (CH), 127.7 (CH), 119.8 (CH), 117.1 (CH), 115.6 (CH), 78.4 (CH), 71.2 (CH_2), 64.3 (CH_2), 64.3 (CH_2), 56.7 (CH_2), 54.1 (CH_2), 53.5 (CH), 36.9 (CH_2), 31.7 (CH_2), 29.2 (CH_2), 29.0 (CH_2), 25.7 (CH_2), 23.6 (CH_2), 22.6 (CH_2), 14.1 (CH_3).
HRMS (+ESI): *m/z* calcd. for $[M - CH_2CH_2 + 3H]^+$ $C_{28}H_{41}N_2O_4$: 469.3061; found: 469.3049. *m/z* calcd. for $[M + H]^+$ $C_{30}H_{43}N_2O_4$: 495.3217; found: 495.3202.

***N*-[(1*R*,2*R*)-1-(2,3-dihydrobenzo[*b*][1,4]dioxin-6-yl)-1-hydroxy-3-(pyrrolidin-1-yl)propan-2-yl]octanamide, "Eliglustat" (1)**

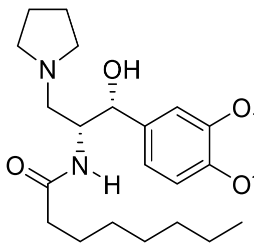

Neat powder catalyst 10% Pd/C (186 mg, 100 w%) was added to a solution of the acylated product **20** (186 mg, 0.38 mmol, 1.0 equiv) in MeOH/10% CH_3COOH (11 mL) under bath oil at 60 °C, and the dark mixture was stirred at 60 °C for 16 h under H_2 atmosphere.

The reaction mixture was cooled down and carefully purged with N_2 until half of the initial volume before filtering the crude through a little path of celite and washing it with clean MeOH (3 × 10 mL). The filtrate was evaporated under reduced pressure and the residue was partitioned with EtOAc (10 mL) and H_2O (10 mL). The aqueous layer was extracted with EtOAc (2 × 10 mL) and the combined organic extracts were dried over $MgSO_4$ and concentrated again. The crude was finally purified via column chromatography (90:10 CH_2Cl_2/MeOH) to give 115 mg (0.28 mmol, 75% yield) of alcohol **1**, also known as **Eliglustat**.

White solid.
Mp 82–86 °C (lit, Mp 85–87 °C)[4].
R_f 0.40 (90:10 CH_2Cl_2/MeOH).
$[\alpha]_D^{20}$ +8.0 (*c* 1.0, $CHCl_3$); **$[\alpha]_D^{20}$** +7.0 (*c* 0.23, $CHCl_3$) (lit, $[\alpha]_D^{20}$ +13 (c 0.23, $CHCl_3$))[4].
IR (ATR) ν 3308 (br), 2954, 2924, 2853, 2812, 1646, 1506, 1457, 1282, 1256, 1124, 1068, 887 cm^{-1}.
^{1}H NMR (400 MHz, $CDCl_3$) δ 6.86 (1H, d, *J* = 2.0 Hz, Ar<u>H</u>), 6.82 (1H, d, *J* = 8.3 Hz, Ar<u>H</u>), 6.77 (1H, dd, *J* = 8.3, 2.0 Hz, Ar<u>H</u>), 5.84 (1H, d, *J* = 7.4 Hz, CHN<u>H</u>CO), 4.90 (1H, d, *J* = 3.3 Hz, C<u>H</u>OH), 4.24 (4H, s, OC<u>H</u>₂C<u>H</u>₂O), 4.22–4.16 (1H, m, C<u>H</u>NHCO), 2.84–2.74 (2H, m, C<u>H</u>₂CHNHCO), 2.65 (4H, dtd, *J* = 13.0, 7.2, 2.7 Hz, $CH_2N(C\underline{H}_2)_4$), 2.12–2.08 (2H, m, $NHCO(C\underline{H}_2)_6CH_3$), 1.80–1.76 (4H, m, $CH_2N(C\underline{H}_2)_4$), 1.52 (2H, p, *J* = 7.7 Hz, $NHCO(C\underline{H}_2)_6CH_3$), 1.28–1.22 (8H, m, $NHCO(C\underline{H}_2)_6CH_3$), 0.87 (3H, t, *J* = 6.9 Hz, $NHCO(CH_2)_6C\underline{H}_3$).
$^{13}C\{^1H\}$ NMR (100.6 MHz, $CDCl_3$) δ 173.4 (C), 143.4 (C), 142.8 (C), 134.4 (C), 118.9 (CH), 117.0 (CH), 115.0 (CH), 75.6 (CH), 64.3 (CH_2), 57.9 (CH_2), 55.2 (CH_2), 52.2 (CH), 36.8 (CH_2), 31.6 (CH_2), 29.1 (CH_2), 29.0 (CH_2), 25.6 (CH_2), 23.6 (CH_2), 22.6 (CH_2), 14.0 (CH_3).
HRMS (+ESI): *m/z* calcd. for $[M + H]^+$ $C_{23}H_{37}N_2O_4$: 405.2748; found: 405.2731.

5. Computational Calculations

5.1. X-Ray and Calculated Geometries

Table S1. Experimental data for the [{(*R*)-DTBM-SEGPHOS}$NiCl_2$] complex, together optimized parameters for this molecular species and related [{(*R*)-DTBM-SEGPHOS}Ni(*N*-azidoacyl-1,3-oxazolidine-2-thione)]$^+$ one (**7**).

Comp.[a]	Exp. A[b]	Exp. B[b]	[(P^P)$NiCl_2$]	[(P^P)Ni(O^S)]$^+$
Ni-S (Å)	-	-	-	2.263
Ni-O (Å)	-	-	-	1.884
Ni-Cl (Å)	2.211, 2.219	2.221, 2.214	2.224, 2.226	-
Ni-P_S (Å)	2.140, 2.167	2.146, 2.147	2.189, 2.189	2.262
Ni-P_O (Å)	-	-	-	2.262
P_S-Ni-S (°)	-	-	-	160.2
P_O-Ni-O (°)	-	-	-	166.9
P-Ni-Cl (°)	147.8, 150.9	147.8, 150.8	158.3, 158.5	-
S-Ni-O (°)	-	-	-	89.3
Cl-Ni-Cl (°)	97.1	98.3	93.8	-
P-Ni-P (°)	93.5	94.3	94.7	99.3
S_{SQ-4}	7.01	7.07	3.55	2.75

[a] For the phosphorous atoms, the nickel-coordinated atom close to *trans* position is indicated.
[b] For experimental structure, two independent molecules A and B are determined.

Table S2. Relative energies (in kcal·mol^{-1}) for the transition states [{(*R*)-DTBM-SEGPHOS}Ni(*N*-azidoacyl-1,3-oxazolidine-2-thione)]$^{+}$ and different acetals. All energies are provided in CH_2Cl_2 solution, and its population distribution at –20 °C. Several geometric parameters are also shown.

(*a*) Acetal **4** containing unhindered ethylene fragment.

TS	**TS4-A1**	**TS4-A2**
E_{rel}	0.00	0.29
%	63.9	36.1
ν_{img}/cm^{-1}	330.8 *i*	353.1 *i*
C···C / Å	2.085	2.044
HC···CH (°)	164.9	157.7
Ni-S (Å)	2.228	2.229
Ni-O (Å)	1.888	1.894
Ni-P_S (Å)	2.280	2.270
Ni-P_O (Å)	2.265	2.253
P-Ni-S (°)	162.6	160.7
P-Ni-O (°)	162.1	160.5
P-Ni-P (°)	95.8	96.4
S_{SQ-4}	2.91	3.36

(*b*) Acetal **15**, incorporating TBS protecting groups.

TS	**TS15-A1**	**TS15-A2**
E_{rel}	0.40	0.00
%	31.2	68.8
ν_{img}/cm^{-1}	366.2 *i*	383.9 *i*
C···C / Å	2.027	1.999
HC···CH (º)	164.8	166.3
Ni-S (Å)	2.229	2.227
Ni-O (Å)	1.884	1.893
Ni-P_S (Å)	2.282	2.277
Ni-P_O (Å)	2.265	2.264
P-Ni-S (°)	162.5	162.2
P-Ni-O (°)	161.8	162.1
P-Ni-P (°)	96.1	96.6
S_{SQ-4}	2.98	2.97

5.2. Methodology

Computational details: ONIOM calculations were carried out using the Gaussian09 package.[5] High quantum layer is defined by nickel, phosphorous, and the α-azidoacetyl-1,3-oxazolidine-2-thione together the electrophile in the reaction pathway, while low layer includes organic frameworks of diphosphine ligand excluding coordinated phosphorous atoms by treated by universal field force.[6] The hybrid density functional known as B3LYP was applied.[7] The all-electron basis sets having triple-*ξ* quality with an extra polarization function were used for all elements (TZVP).[8] The geometries were fully optimized without restrictions and transition states were confirmed by vibrational analysis. Solvent effects of dichloromethane were taken into account by PCM algorithm,[9] keeping the optimized geometry for the gas phase (single-point calculations).

Structural Analysis: Continuous shape measures were calculated with the SHAPE program,[10] that provides quantitative information of how much the environment is deviated from an ideal polyhedron (SQ = square-planar, TT = tetrahedral).

5.3. Atomic Coordinates

Table S1. Atomic coordinates for calculated geometries of DTBM–SEGPHOS derivatives.

(*a*) [{(*R*)-DTBM-SEGPHOS}$NiCl_2$]

Ni	0.00410000	0.01426000	-1.53806200
Cl	-1.55716900	0.49187600	-3.04807400
Cl	1.55558700	-0.43989000	-3.06821300
P	-1.55528000	-0.36320600	-0.04883300
P	1.58215400	0.35898800	-0.06099700
C	-2.34379300	1.15074900	0.52465900
C	-1.05860200	-1.30752600	1.41793800
C	-2.85778000	-1.32051200	-0.89618800
C	1.11546200	1.26503600	1.43258300
C	2.86598100	1.34028700	-0.90323900
C	2.37310400	-1.16674100	0.47300600
C	0.17680800	0.68020900	2.43695300
C	-0.19451400	1.49483400	3.51636100
C	0.27978800	2.70575600	3.68187800
C	1.20045600	3.27964800	2.80575100
C	1.61973100	2.55849300	1.68003600
C	-1.04477900	2.29285100	5.33641700
C	-1.48461100	-2.65142000	1.57309100
C	-1.07367400	-3.41347700	2.68026200
C	-0.27381700	-2.85206800	3.59184900
C	0.14405600	-1.56616400	3.49388500
C	-0.23092200	-0.74079500	2.42333400
C	0.95604500	-2.41089300	5.31741800
C	2.01581800	-2.36521200	-0.13289800
C	2.68491100	-3.56742900	0.16727200
C	3.81827800	-3.48647500	1.03578600
C	4.01967200	-2.33073000	1.84850900
C	3.30743800	-1.16243600	1.50513200
C	4.98179600	-2.34830000	3.07164300
C	6.45581500	-2.35464000	2.60319900
C	4.70781700	-3.60499000	3.94101200
C	4.78625100	-1.11065800	3.99613300
C	2.12533600	-4.88290600	-0.44651500
C	2.82898100	-6.17544900	0.05038300
C	0.62896700	-5.02373600	-0.05702100
C	2.23864400	-4.82441300	-1.98936100
C	5.63675300	-4.29341700	-0.06386000
C	4.18869300	0.90790900	-0.99971700
C	2.47105600	2.49119800	-1.57882400
C	3.38543000	3.26142600	-2.31950100
C	4.77480800	2.94755000	-2.17941000
C	5.17276700	1.68407800	-1.64931700
C	6.64302900	1.18072400	-1.75746300
C	6.81706000	-0.29037300	-1.27572300
C	7.57837400	2.06145900	-0.89242700
C	5.75902900	4.96890000	-1.61237900
C	7.10118400	1.22616200	-3.23852600
C	2.80240200	4.27308500	-3.35069200
C	2.07510900	5.42099800	-2.60984500
C	3.85989500	4.88297800	-4.31299400

C	1.77003600	3.53482100	-4.25306700
C	-3.26380000	1.12485600	1.57057900
C	-3.99303900	2.27874100	1.92912000
C	-3.80441800	3.44790100	1.13523700
C	-2.66668100	3.55365400	0.27427000
C	-2.00040200	2.36122500	-0.06498300
C	-2.04210800	4.89595700	-0.20553700
C	-2.73291100	6.16467400	0.36822200
C	-2.06798900	4.96797200	-1.75231900
C	-0.56499900	4.95564300	0.27062000
C	-4.85910400	3.49163900	3.99567300
C	-5.51452000	4.43422600	-0.07766100
C	-5.02424300	2.23952000	3.09221800
C	-4.83740200	0.99522800	4.00893600
C	-4.76407300	-2.90148700	-2.13629900
C	-3.37679500	-3.20369900	-2.31078400
C	-3.58069100	-4.07525600	-4.68687900
C	-2.47648200	-2.43641100	-1.64634900
C	-4.18292700	-0.88631000	-0.94276600
C	-5.16247600	-1.62944100	-1.62831400
C	-6.03854400	-4.63967900	-1.32407600
C	-3.26466100	-5.72239700	-2.79317000
C	-7.68688400	-1.77584700	-1.19042200
C	-6.56358800	-1.01819400	-1.93596700
C	-6.66635500	0.48161500	-1.52632700
C	-1.36593900	-4.28905100	-3.56027400
C	-6.46177800	2.19421100	2.52314600
C	-2.90456800	-4.30932300	-3.30980100
C	-6.82302400	-1.08662000	-3.46354100
H	1.54551300	4.18460700	2.97236000
H	2.25962400	2.97826100	1.06816700
H	-1.98430400	2.65127200	5.40079900
H	-0.71707600	2.04031800	6.25639100
H	-2.06786800	-3.07567300	0.91057100
H	-1.36812300	-4.34584500	2.78073800
H	1.91183000	-2.71432400	5.41525700
H	0.57344500	-2.21743500	6.23042100
H	1.28754000	-2.35560000	-0.79056200
H	3.47860400	-0.31238000	1.96006400
H	6.61338400	-1.61992000	1.93097700
H	7.07744000	-2.20909800	3.38412100
H	6.70504200	-3.23484200	2.18773200
H	4.96059000	-4.45226000	3.46197700
H	5.24470900	-3.57420200	4.79438300
H	3.73073500	-3.65780400	4.18093200
H	3.82109100	-1.02581700	4.27664800
H	5.34647000	-1.19415800	4.83094600
H	5.06987400	-0.26260600	3.53042000
H	2.32835200	-6.99414700	-0.26094800
H	3.76096600	-6.25413400	-0.31767900
H	2.86110100	-6.19833500	1.05800600
H	0.23766400	-5.87475700	-0.43167400
H	0.53173900	-5.04452500	0.94611000
H	0.01688900	-4.18780900	-0.44889200
H	1.71001900	-4.05282200	-2.36428800

H	3.20006700	-4.71310300	-2.26870700
H	1.88391200	-5.67308000	-2.40344200
H	6.04448800	-3.36913600	-0.07138600
H	6.38753700	-4.96117800	-0.00325300
H	5.15509300	-4.44921200	-0.93499900
H	4.42329000	0.05413400	-0.59221500
H	1.51746800	2.72564700	-1.57810600
H	7.76546600	-0.60386600	-1.41733600
H	6.61646800	-0.37267600	-0.29106500
H	6.20800000	-0.90917100	-1.78933600
H	7.67964700	2.98425300	-1.27641300
H	7.21626500	2.13918800	0.04570800
H	8.50195100	1.65876600	-0.84395000
H	4.88952100	5.47150900	-1.60185400
H	5.98078700	4.68276100	-0.67056300
H	6.48179700	5.59314300	-1.93057100
H	7.12028300	2.16697700	-3.59398000
H	8.03155700	0.84849400	-3.33513700
H	6.47579200	0.68251400	-3.81370700
H	1.30974300	5.06484800	-2.06133000
H	2.70358300	5.90762700	-1.99326200
H	1.70069600	6.08629600	-3.26903000
H	3.40997000	5.39414400	-5.05724200
H	4.45980400	5.53711000	-3.84432700
H	4.41975600	4.15649100	-4.73197300
H	2.20223700	2.74106900	-4.70133200
H	0.97778300	3.20773900	-3.72394300
H	1.41398200	4.14909800	-4.96970100
H	-3.42597900	0.26572800	2.01143500
H	-1.26809900	2.37299700	-0.71878300
H	-3.64062800	6.31089100	-0.03478700
H	-2.81864200	6.10061700	1.37090900
H	-2.19131000	6.98919800	0.15703200
H	-1.67270200	5.83915800	-2.07216700
H	-1.53186700	4.21784900	-2.15947700
H	-3.01136800	4.90399700	-2.09715700
H	-0.12973000	5.82060600	-0.01214000
H	-0.52343200	4.89521200	1.27587800
H	-0.01951200	4.20381100	-0.11677700
H	-5.44107300	3.42007900	4.81639800
H	-3.90081700	3.58464100	4.29257200
H	-5.12541700	4.33562500	3.51846800
H	-4.93543300	4.62795300	-0.87596700
H	-6.00265700	3.56486300	-0.23374600
H	-6.19741200	5.16920000	0.00425000
H	-5.06401800	0.14456200	3.51772000
H	-3.88707400	0.93988300	4.34195900
H	-5.44687400	1.04616700	4.81119200
H	-3.37085000	-3.14857600	-5.02586900
H	-3.25064700	-4.74484000	-5.36539400
H	-4.58076900	-4.17167300	-4.62965000
H	-1.51840200	-2.63362800	-1.68540100
H	-4.41429200	-0.04230600	-0.51323600
H	-6.73928200	-5.29851300	-1.62110600
H	-5.24445500	-5.15322600	-0.97775800

H	-6.41194700	-4.09891000	-0.56137000
H	-2.78835200	-6.43198600	-3.32903600
H	-2.99393900	-5.82209300	-1.82681900
H	-4.24841100	-5.90544400	-2.88431600
H	-7.85748800	-2.67569000	-1.60426200
H	-7.44526500	-1.89427300	-0.21859600
H	-8.55568800	-1.26562500	-1.23937500
H	-7.55617300	0.86883000	-1.80221800
H	-6.58754400	0.58677800	-0.52673800
H	-5.94327700	1.02533000	-1.97272300
H	-1.06555600	-3.37836600	-3.87342500
H	-0.86345600	-4.53249800	-2.72184500
H	-1.10831600	-4.95847200	-4.26977400
H	-6.55736000	1.42726900	1.87551200
H	-7.13276200	2.06891100	3.26577700
H	-6.69566100	3.04749600	2.04638600
H	-7.70339500	-0.65235800	-3.69591000
H	-6.08972200	-0.60816200	-3.96460500
H	-6.85828900	-2.03898200	-3.78824200
O	-1.01413100	1.14690200	4.47562000
O	-0.17857400	3.28138800	4.76252800
O	0.89980700	-1.22549200	4.50498100
O	0.17908800	-3.43418200	4.66971400
O	4.77452300	-4.48393300	1.04445000
O	5.71670700	3.82918400	-2.48459100
O	-4.75502800	4.37184400	1.13952900
O	-5.68129600	-3.80678100	-2.44044700

(*b*) Transition state **TS_4-A1**, using acetal **4**

Ni	0.58001600	0.44317100	-0.73088100
S	1.18578300	2.20026200	-1.95898500
C	-0.23498400	2.95080800	-2.51743500
O	-0.07135000	4.00473700	-3.27403200
C	-1.36973500	4.58705200	-3.63642000
C	-2.37991600	3.51875600	-3.21908700
N	-1.54011200	2.62897700	-2.35918600
C	-2.02529000	1.45579600	-1.76357700
O	-1.25199200	0.69803300	-1.11094600
H	-1.34369100	4.78510600	-4.70356900
H	-1.46998600	5.51274500	-3.07507800
H	-2.75582600	2.94351500	-4.06521200
H	-3.20129500	3.92974200	-2.63902600
C	-3.41624400	1.19054400	-1.83652600
H	-4.00758800	1.80055800	-2.50576900
N	-3.74357000	-0.18819100	-1.77568200
N	-4.79087900	-0.50514200	-2.35939000
N	-5.70562400	-0.91815800	-2.86862400
P	2.57039100	0.39562900	0.34861600
P	-0.07668300	-1.64665800	-0.09767100
C	2.43328300	-0.23123900	2.06901600
C	3.23657200	2.10441600	0.45239800
C	3.89734700	-0.60705100	-0.38551300
C	1.24787600	-2.86320700	0.18804500
C	-1.16513500	-2.31389600	-1.41667100
C	-1.02064400	-1.64947700	1.46305800

C	2.16142900	-2.76098500	1.37229400
C	1.36757400	-3.98471200	-0.65361300
C	2.08398200	-1.66218100	2.37466400
C	1.84685200	-1.97639100	3.72486700
C	2.28895500	-4.99372700	-0.35791000
C	3.07063300	-4.88987800	0.79128400
C	3.02054000	-3.85283800	1.59541200
C	1.97281800	-1.09856400	4.69121000
C	2.36935000	0.21454400	4.46797500
O	3.88993200	-5.81627100	1.21261500
O	3.81579000	-3.99091000	2.62635500
O	1.51343700	-3.15715800	4.17607500
O	1.73399300	-1.60513500	5.87083700
H	0.73671400	-4.10275600	-1.52276800
H	2.36422100	-5.86617100	-0.99409900
H	2.49039900	0.89800300	5.29867300
H	2.94013600	1.65998200	3.03282700
H	4.98603800	1.70925100	-0.70374100
H	1.47299400	2.79740800	1.37919200
H	2.69604000	-1.17156200	-2.02566400
H	5.20491900	-0.28610700	1.26451900
H	-2.30865300	-3.58819900	-0.14213600
H	-0.24669400	-1.07195400	-2.87830700
H	-1.04818800	-3.76832900	1.63161400
H	-0.83806200	0.44502700	1.62900500
H	0.38534800	-3.19464600	5.94137500
H	2.13791900	-3.64110100	6.11233100
H	4.10503900	-5.95308700	3.28659000
H	5.50254200	-5.22112900	2.40319300
C	2.61545800	0.64315900	3.16206800
C	2.43880300	3.10410500	1.00863900
C	1.41658200	-2.97315800	5.59506900
C	4.39710500	-5.28960800	2.44453900
C	2.88600000	4.44031000	1.11071000
C	4.19051200	4.73609000	0.61793600
C	4.88537900	3.79269300	-0.19540800
C	4.42843800	2.46214500	-0.17849500
C	3.68917800	-1.24103700	-1.60933400
C	4.73623400	-1.89761500	-2.29698800
C	6.03854400	-1.81483500	-1.73379100
C	6.20726100	-1.37682800	-0.30720100
C	5.11644000	-0.72072400	0.27828000
C	-2.17208400	-3.23947600	-1.14157400
C	-3.05161000	-3.69619600	-2.13193900
C	-2.75068000	-3.28773300	-3.54363700
C	-1.83227500	-2.22991000	-3.78985200
C	-1.01832000	-1.81148200	-2.71372200
C	-1.24918000	-0.43317900	2.10163800
C	-1.98229900	-0.34455900	3.30421900
C	-2.60319200	-1.53036300	3.77876800
C	-2.15336100	-2.86859300	3.25488600
C	-1.38309900	-2.84796800	2.08108600
O	-3.71824800	-1.42818100	4.61982200
O	7.15662300	-1.98081000	-2.55610700
O	-3.20670600	-4.08378300	-4.59643400

O	4.82748900	5.91203000	1.03436900
C	-2.33871400	-5.18321800	-4.87349000
H	-2.76123400	-5.77127500	-5.71344600
H	-2.24873400	-5.84915300	-3.99031400
H	-1.33042900	-4.82433200	-5.16969800
C	7.71699600	-0.73491800	-2.98113600
H	8.58379200	-0.93751100	-3.64251100
H	8.06886100	-0.13579800	-2.11800700
H	6.97227500	-0.13768900	-3.54915900
C	-4.97141800	-1.61725500	3.94901800
H	-4.85838600	-1.88385200	2.87464700
H	-5.57341400	-0.68921200	4.01140400
H	-5.54594500	-2.41876100	4.45462500
C	-1.67015700	-1.51528700	-5.16088400
C	-4.27634800	-4.54295800	-1.70472900
C	-5.07128500	-3.79918800	-0.59998700
H	-5.97581000	-4.37431200	-0.30565600
H	-5.40015100	-2.80341400	-0.95846500
H	-4.47651000	-3.65168600	0.32358200
C	-5.26736000	-4.81839500	-2.85993000
H	-4.85310200	-5.54459700	-3.58871700
H	-5.54090400	-3.87501600	-3.37953100
H	-6.20525000	-5.27530700	-2.47576800
C	-3.79658800	-5.89908400	-1.14598600
H	-3.22305300	-6.45875500	-1.91364700
H	-4.66232200	-6.52402700	-0.83660500
H	-3.14250500	-5.76537800	-0.25964700
C	-0.25367500	-1.76672300	-5.71505300
H	-0.14718200	-1.34160400	-6.73671700
H	-0.03804100	-2.85475400	-5.76443300
H	0.51173600	-1.28370000	-5.07729600
C	-2.69159700	-1.95730800	-6.23806200
H	-2.47463800	-2.97876300	-6.61213200
H	-2.63924300	-1.29476000	-7.12945600
H	-3.72999000	-1.90369200	-5.84523500
C	-1.86842500	0.00844400	-4.97176200
H	-2.87329400	0.21672500	-4.54762200
H	-1.10199400	0.44832000	-4.30262300
H	-1.78571900	0.54565400	-5.94144600
C	-2.10008600	0.99601900	4.07779100
C	-2.50823700	-4.22436300	3.92191800
C	-1.17799600	2.10289200	3.50485000
H	-1.49919900	2.40210700	2.48580000
H	-0.12090100	1.76229800	3.47273800
H	-1.21489200	3.01722700	4.13627900
C	-3.54729500	1.53346100	4.03585300
H	-3.58489600	2.60405600	4.33300900
H	-4.20412100	0.99909900	4.74805800
H	-3.96799200	1.44607600	3.01210600
C	-1.66879800	0.77718300	5.54639400
H	-2.33223300	0.05974200	6.07062400
H	-1.70342900	1.73170500	6.11518600
H	-0.63074300	0.38190700	5.58919500
C	-3.68147000	-4.87433900	3.16424200
H	-4.58470600	-4.23585300	3.18941100

H	-3.94501400	-5.85488300	3.61675700
H	-3.41094700	-5.04591100	2.10128700
C	-2.88544300	-4.06282900	5.41572300
H	-2.90087800	-5.04455000	5.93662300
H	-3.90203800	-3.64967200	5.54868600
H	-2.15147200	-3.41172400	5.93704500
C	-1.31248400	-5.21308600	3.87370500
H	-0.40577800	-4.75431200	4.30515600
H	-1.08696400	-5.55718700	2.84388900
H	-1.53297700	-6.13540000	4.45378200
C	1.95961100	5.55647700	1.67078300
C	6.08294900	4.14806300	-1.11937400
C	1.74293700	6.62339400	0.57482800
H	2.69536400	7.11791900	0.29236500
H	1.04528000	7.41519900	0.92466600
H	1.31541800	6.15715800	-0.33901900
C	2.57244000	6.21958400	2.92570600
H	1.80250900	6.77180200	3.50741300
H	3.33543900	6.97636400	2.66050000
H	3.02221800	5.45303000	3.59296600
C	6.25063500	5.67068500	-1.36248900
H	5.27642000	6.13641400	-1.62577000
H	6.95644800	5.86880700	-2.19807200
H	6.69017900	6.19056600	-0.49264700
C	7.38142200	3.58764300	-0.50886200
H	8.25330500	3.82428300	-1.15641200
H	7.32311600	2.48381800	-0.39887800
H	7.56387500	4.02416800	0.49478800
C	5.87653800	3.51388100	-2.52353000
H	6.65081000	3.86127500	-3.24149700
H	4.87869300	3.78894300	-2.92910700
H	5.96181600	2.40822400	-2.50853800
C	7.44856200	-1.68066500	0.56355100
C	4.47649000	-2.67847300	-3.61491200
C	8.11723300	-0.36315900	1.01099100
H	9.01635500	-0.57214900	1.63032600
H	7.42774600	0.25824900	1.61986700
H	8.43642900	0.23545100	0.13397700
C	7.00211900	-2.46691700	1.81644600
H	6.31222100	-1.87938200	2.45711500
H	7.87352900	-2.74665800	2.44744700
H	6.48473700	-3.39440200	1.50211000
C	8.50981000	-2.54995900	-0.15206000
H	9.03830800	-1.98479500	-0.94455800
H	8.04754800	-3.46365800	-0.58420900
H	9.29637000	-2.87832400	0.56180600
C	5.17661200	-2.00298700	-4.81559300
H	4.81075300	-2.42461700	-5.77712100
H	6.27050900	-2.17152400	-4.81219500
H	4.97090000	-0.91096200	-4.82003900
C	4.99590600	-4.12498200	-3.45844900
H	6.09223300	-4.15180100	-3.29132700
H	4.78578100	-4.72193100	-4.37243000
H	4.50182300	-4.62114800	-2.59483400
C	2.97193900	-2.77359800	-3.96793400

H	2.56438600	-1.76579700	-4.18810600
H	2.39580300	-3.23428300	-3.14045600
H	2.80995000	-3.39921700	-4.87276300
C	0.56016100	5.03029300	2.07963000
H	-0.10084700	5.86073400	2.41118700
H	0.64570800	4.31416600	2.92441900
H	0.05649000	4.53401700	1.22571900
C	5.78627400	5.70431600	2.08460200
H	5.86223600	4.64125300	2.40995300
H	5.51035300	6.31321900	2.96801000
H	6.78794000	6.04323800	1.75483400
C	-4.07987900	2.06692600	-0.06470400
H	-3.28920500	1.65328600	0.55919600
C	-5.38124500	1.47299100	0.09674900
C	-6.54893900	2.12402900	-0.34109700
C	-7.78285300	1.52608900	-0.18218200
C	-7.87066000	0.25434700	0.44090600
C	-6.70660200	-0.40768300	0.84555100
C	-5.47746500	0.19145200	0.67848500
H	-6.50273400	3.10609000	-0.79235500
H	-4.58078000	-0.31584200	1.00837800
H	-6.81001300	-1.37701700	1.31468300
O	-9.04784800	-0.34377500	0.66700300
O	-8.89137400	2.18168100	-0.60610100
C	-10.06251100	1.35266600	-0.73809300
C	-10.22270100	0.48137900	0.48468900
H	-10.90434600	2.03258400	-0.84783900
H	-9.96993500	0.74446300	-1.64203900
H	-11.05709000	-0.20830500	0.38013200
H	-10.36092100	1.08487700	1.38447100
O	-4.00084900	3.39658700	-0.30140900
C	-3.11337700	4.14409700	0.60021600
H	-2.11017000	3.71827700	0.52047800
H	-3.48259400	3.99262500	1.61683600
C	-3.11279100	5.59339300	0.22821800
C	-1.98852300	6.17571700	-0.36020200
C	-4.22814300	6.39128900	0.49509600
C	-1.97038500	7.53163100	-0.67237300
C	-4.21857900	7.74191400	0.17008300
C	-3.08952300	8.31484100	-0.41073700
H	-1.10565000	5.57443900	-0.54647100
H	-5.10229200	5.95722000	0.96602700
H	-1.08197400	7.97940700	-1.10039200
H	-5.08538800	8.35369100	0.38462000
H	-3.07855400	9.37147100	-0.64554700

(*c*) Transition state **TS$_4$-A2**, using acetal **4**

Ni	-0.57653100	0.56896600	0.73987600
S	-1.02394600	2.43561800	1.87241900
C	0.47222600	3.16002000	2.22155200
O	0.43161800	4.35725800	2.74551300
C	1.79447400	4.87389300	2.92043900
C	2.65258300	3.61691900	2.86628900
N	1.74147100	2.68399400	2.13445300
C	2.09382600	1.37864800	1.76970200

O	1.26566900	0.63882300	1.17378000
H	1.82178600	5.39964900	3.86945200
H	1.98111000	5.55765500	2.09418100
H	2.85849800	3.20536300	3.85560600
H	3.57303900	3.76793600	2.31252000
C	3.43174100	0.94332500	1.99453000
H	4.04131100	1.54674100	2.65518800
N	3.54290400	-0.46545500	2.15700100
N	4.49013600	-0.84832500	2.86099700
N	5.29335900	-1.33079600	3.48441200
P	-2.48676600	0.72114500	-0.44507800
P	-0.17074600	-1.61850300	0.28821600
C	-2.35158000	-0.08088200	-2.08729200
C	-2.81861700	2.50064100	-0.74472000
C	-4.01230400	0.02528200	0.25977000
C	-1.64551800	-2.66536700	0.06917100
C	0.74783600	-2.28085900	1.72936800
C	0.81894500	-1.91333300	-1.21502900
C	-2.52879200	-2.53609200	-1.13593100
C	-1.93380000	-3.68077500	1.00131700
C	-2.27105300	-1.57483600	-2.24138200
C	-2.07819200	-2.06129800	-3.54439600
C	-2.99891800	-4.55886700	0.78179400
C	-3.76413400	-4.42677200	-0.37405200
C	-3.55489800	-3.48854200	-1.26354900
C	-2.00232500	-1.27954900	-4.59024100
C	-2.12324100	0.10334100	-4.50921300
O	-4.76814000	-5.29075400	-0.76010600
O	-4.41228300	-3.61905600	-2.34095200
O	-1.98212500	-3.39088700	-3.90480600
O	-1.84143800	-1.99338600	-5.75982100
H	-1.33340800	-3.81666900	1.88893500
H	-3.20590500	-5.35345300	1.48661900
H	-2.07648100	0.70902400	-5.40486700
H	-2.40678500	1.77419900	-3.23243500
H	-4.73399000	2.51978100	0.19751000
H	-0.84709900	2.79545800	-1.44364400
H	-3.02669200	-0.61890200	2.01075100
H	-5.13976600	0.44201400	-1.49770200
H	1.80395700	-3.79791100	0.66225600
H	-0.09056000	-0.79217200	2.99405100
H	0.56575200	-4.01880100	-1.13943900
H	0.94354000	0.15473600	-1.60248200
H	-0.86105400	-3.83070600	-5.61619900
H	-2.66221100	-3.91432800	-5.81068000
H	-4.97882100	-5.57385300	-2.81949600
H	-6.25779500	-4.56927700	-2.03165100
C	-2.30776700	0.70235600	-3.26015800
C	-1.77827300	3.29577800	-1.22519200
C	-1.82613400	-3.36235900	-5.33203200
C	-5.17421200	-4.80134000	-2.04602600
C	-1.92769000	4.68910700	-1.39486500
C	-3.21790600	5.24464900	-1.15673600
C	-4.20329300	4.49868400	-0.44307900
C	-3.99239400	3.11351300	-0.30433700

C	-3.98915400	-0.55537800	1.52682600
C	-5.16980800	-1.00268700	2.16445300
C	-6.40314400	-0.75864900	1.49991200
C	-6.40577600	-0.38212800	0.04507600
C	-5.18767900	0.06015000	-0.48710400
C	1.62952200	-3.35633600	1.62004800
C	2.32373400	-3.86028200	2.73008800
C	1.97204300	-3.27965900	4.06951700
C	1.22222800	-2.07342300	4.13826700
C	0.57351500	-1.64499300	2.96141900
C	1.23042000	-0.81773900	-1.97093400
C	1.97471500	-0.96356400	-3.16322600
C	2.40938800	-2.27323100	-3.50622300
C	1.77827200	-3.46810700	-2.83991000
C	1.02363200	-3.21019600	-1.68479500
O	3.49055400	-2.43176600	-4.38251600
O	-7.58829500	-0.71936000	2.24008100
O	2.19105800	-4.03578800	5.22290600
O	-3.52253300	6.50129500	-1.68782900
C	1.12618600	-4.94758600	5.49497700
H	1.36605500	-5.51463800	6.41735000
H	1.00165200	-5.67052300	4.66179900
H	0.17226400	-4.40282900	5.65709700
C	-7.98500500	0.61363800	2.57552600
H	-8.91420500	0.57101500	3.17935900
H	-8.18650500	1.21466700	1.66742100
H	-7.19968100	1.12418800	3.17249900
C	4.72969800	-2.74117200	-3.73069700
H	4.63153100	-2.84205500	-2.62676100
H	5.47050000	-1.94650100	-3.94266600
H	5.14330400	-3.68415600	-4.13923100
C	1.06581600	-1.21010700	5.42073100
C	3.40826000	-4.94204200	2.49993400
C	4.43844200	-4.43659100	1.45970700
H	5.23679900	-5.19175800	1.29212200
H	4.91955100	-3.50407500	1.81841500
H	3.97886300	-4.23587400	0.47039100
C	4.20500100	-5.30188700	3.77598400
H	3.58800700	-5.88098500	4.49277500
H	4.59969800	-4.38631100	4.26709800
H	5.07328800	-5.95104100	3.52939100
C	2.74740500	-6.23027600	1.96503300
H	1.98860200	-6.60716200	2.68224500
H	3.50731600	-7.02727000	1.81299300
H	2.24380700	-6.05595300	0.99155800
C	-0.41426800	-1.18167400	5.85384700
H	-0.53321600	-0.64279000	6.81887300
H	-0.80718800	-2.21252000	5.97863200
H	-1.03827300	-0.65512800	5.10479300
C	1.90911200	-1.69758500	6.62465200
H	1.49773000	-2.63132900	7.05983600
H	1.89342600	-0.95011700	7.44763500
H	2.97065600	-1.84609200	6.33055000
C	1.52587800	0.23837300	5.12177900
H	2.58447500	0.24665400	4.78661300

H	0.90455300	0.72094300	4.34052600
H	1.44670900	0.87695500	6.02822900
C	2.27543600	0.25728500	-4.07748000
C	1.92180800	-4.92642000	-3.35178600
C	1.55815700	1.54965200	-3.60991600
H	1.95081400	1.88631800	-2.62647900
H	0.46156800	1.39301800	-3.53653000
H	1.72488700	2.38133800	-4.32879200
C	3.78625800	0.57046700	-4.11800000
H	3.97381500	1.56876300	-4.56982700
H	4.34155600	-0.14985500	-4.74590500
H	4.20897000	0.57704300	-3.09326400
C	1.77296400	-0.04146900	-5.50826100
H	2.30655800	-0.90082500	-5.96272500
H	1.93205300	0.83398100	-6.17466200
H	0.68779100	-0.27766100	-5.49539100
C	3.01091300	-5.64679300	-2.53370900
H	3.99718800	-5.16023400	-2.65335400
H	3.11116600	-6.70450300	-2.86053700
H	2.75611800	-5.63947900	-1.45240200
C	2.27628600	-4.98951900	-4.85873000
H	2.13559700	-6.01451100	-5.26476700
H	3.33691400	-4.74952900	-5.05207800
H	1.62983500	-4.29962800	-5.44220500
C	0.59862700	-5.72118000	-3.18013300
H	-0.24815900	-5.17213700	-3.63052800
H	0.36660100	-5.93351800	-2.11644900
H	0.66001600	-6.71572300	-3.67284200
C	-0.70897600	5.59149400	-1.74014500
C	-5.44019900	5.11668600	0.26873000
C	-0.57714500	6.70727200	-0.67735000
H	-1.45136700	7.38879600	-0.67855700
H	0.32254900	7.33185800	-0.86844100
H	-0.48422800	6.26353600	0.33804600
C	-0.86146700	6.22345200	-3.14057100
H	0.09572200	6.67767500	-3.47757400
H	-1.60379500	7.04436600	-3.14756400
H	-1.15657300	5.45199000	-3.88403900
C	-5.50400300	6.66373900	0.22167600
H	-4.54836600	7.11079000	0.57097200
H	-6.31041900	7.04652300	0.88449400
H	-5.75383700	7.03792200	-0.78993100
C	-6.73571200	4.55928000	-0.35860400
H	-7.63078800	5.00998000	0.12245300
H	-6.80626500	3.45952800	-0.23080500
H	-6.77528900	4.78303600	-1.44452900
C	-5.40604200	4.73233000	1.77148700
H	-6.23588500	5.21889400	2.32877100
H	-4.44477200	5.05232800	2.22919100
H	-5.52300900	3.64103300	1.93211200
C	-7.62105800	-0.53081300	-0.90152600
C	-5.12270700	-1.73837800	3.53293000
C	-8.05406700	0.85376300	-1.42868500
H	-8.92105100	0.75567000	-2.11744700
H	-7.23396500	1.35079400	-1.98821400

H	-8.35491600	1.51907800	-0.59494400
C	-7.22279200	-1.41692900	-2.10429900
H	-6.42519500	-0.95436900	-2.72163200
H	-8.08952800	-1.59448700	-2.77740500
H	-6.85870700	-2.39587200	-1.73853800
C	-8.84312700	-1.20728100	-0.23614700
H	-9.33308800	-0.54028000	0.49986800
H	-8.54898600	-2.15992000	0.25500200
H	-9.62222900	-1.44609600	-0.99246300
C	-5.79210000	-0.90026500	4.64525000
H	-5.56057200	-1.31636200	5.64995200
H	-6.89610600	-0.90575800	4.56720500
H	-5.42398800	0.14778200	4.61556600
C	-5.84516000	-3.09791100	3.40286700
H	-6.91915900	-2.97076100	3.15630700
H	-5.79007100	-3.66823200	4.35552100
H	-5.37634600	-3.70909800	2.60126900
C	-3.67809000	-2.03739000	4.00504800
H	-3.13424600	-1.09268400	4.21095500
H	-3.12448000	-2.62631600	3.24696700
H	-3.67759600	-2.62838300	4.94689100
C	0.63283900	4.81630300	-1.73312600
H	1.48987900	5.50246800	-1.90483500
H	0.65662400	4.06310300	-2.54754800
H	0.79722400	4.31647800	-0.75352100
C	-4.23542800	6.41483500	-2.92591400
H	-3.64624000	5.86138300	-3.68766500
H	-4.42572300	7.43997000	-3.30381200
H	-5.21050500	5.90398300	-2.79307500
C	4.39906100	1.31712800	0.23327200
H	3.57559900	1.07089800	-0.43792400
C	4.80578800	2.72306800	0.24667000
C	6.00710500	3.12771500	0.84200300
C	6.39105100	4.45948200	0.81158000
C	5.55246500	5.41898900	0.19740100
C	4.37118000	5.00697400	-0.42117900
C	3.99677800	3.67324700	-0.39500500
H	6.67899900	2.40789200	1.29109800
H	3.10050900	3.35897100	-0.91477700
H	3.77863600	5.75325100	-0.93405500
O	5.86996000	6.72817700	0.16023800
O	7.57385300	4.81273600	1.37054900
C	8.08457200	6.08227300	0.91681200
C	6.99522400	7.12732100	0.97424400
H	8.46649800	5.96948400	-0.10117700
H	8.90684700	6.33393600	1.58258300
H	7.33292500	8.07937700	0.57054000
H	6.64919000	7.27375300	2.00056800
O	5.37543800	0.41715900	0.28712800
C	5.22613500	-0.85275400	-0.44626900
H	4.38605100	-0.72939900	-1.13332900
H	4.96901300	-1.62417000	0.27358900
C	6.50118200	-1.18265000	-1.15532200
C	7.03908300	-0.31440100	-2.10950700
C	7.13218100	-2.40062300	-0.90409400

C	8.18657800	-0.66637400	-2.80556400
C	8.27650900	-2.75777100	-1.60999200
C	8.80344600	-1.89170700	-2.56060300
H	6.56541000	0.63966000	-2.30914700
H	6.72676300	-3.07904900	-0.16227400
H	8.59841300	0.00856400	-3.54496300
H	8.75621400	-3.70807000	-1.41439400
H	9.69425600	-2.16788200	-3.11012700

(*d*) Transition state **TS$_{15}$-A1**, using acetal **15**

Ni	-1.72064000	0.52718700	0.69042500
S	-2.26536600	2.40723200	1.75580500
C	-0.81705700	3.15281400	2.24933300
O	-0.94122400	4.27440900	2.91245300
C	0.37910100	4.84143000	3.21351500
C	1.34887600	3.70429400	2.89437100
N	0.47400000	2.77141400	2.12113700
C	0.91685100	1.53796000	1.62738000
O	0.11746700	0.75745700	1.03314400
H	0.36775000	5.14002900	4.25724600
H	0.50807300	5.70622900	2.56719700
H	1.70850200	3.19488600	3.78839400
H	2.18105900	4.02970800	2.27662100
C	2.30217400	1.23422100	1.70088900
H	2.92041400	1.88224800	2.30782900
N	2.57167600	-0.15953200	1.79244400
N	3.62252900	-0.45301600	2.37921500
N	4.53436900	-0.84566800	2.91014000
P	-3.71775600	0.44904700	-0.37431200
P	-1.12780700	-1.63244900	0.25256300
C	-3.61436000	-0.34127500	-2.02821800
C	-4.32524300	2.16159400	-0.63769300
C	-5.07323600	-0.43320700	0.45665400
C	-2.49294000	-2.82590100	0.08612700
C	-0.05670000	-2.20879300	1.62806000
C	-0.19192100	-1.81706600	-1.30387800
C	-3.41654300	-2.79639900	-1.09380200
C	-2.64424500	-3.86267100	1.02616100
C	-3.30946700	-1.80384800	-2.19494600
C	-3.09240300	-2.25467700	-3.50718400
C	-3.61116400	-4.85444800	0.83223400
C	-4.41050800	-4.81402000	-0.30847900
C	-4.32790000	-3.86011300	-1.20174100
C	-3.19632000	-1.47490800	-4.55200500
C	-3.55638700	-0.13586100	-4.46009700
O	-5.31500100	-5.78904200	-0.67717600
O	-5.18282400	-4.09075300	-2.26416500
O	-2.80066700	-3.55217600	-3.87816500
O	-2.97915700	-2.15630100	-5.73142800
H	-2.00560600	-3.92882800	1.89509600
H	-3.71071100	-5.66608100	1.54097600
H	-3.66705400	0.46258800	-5.35504100
H	-4.07793600	1.46016400	-3.17065700
H	-6.06987400	1.94431100	0.57186800
H	-2.55116100	2.69710600	-1.64520100

H	-3.87701600	-0.88845600	2.13433200
H	-6.38399600	-0.22092500	-1.20858400
H	1.02615000	-3.64519600	0.48082900
H	-0.92161400	-0.80185600	2.96779300
H	-0.23496600	-3.94189200	-1.26759700
H	-0.30961000	0.25578200	-1.67594400
H	-1.70319300	-3.80393900	-5.64374000
H	-3.47293300	-4.18619900	-5.75337000
H	-5.51782200	-6.09988900	-2.73377400
H	-6.89728900	-5.25231800	-1.93290300
C	-3.78085100	0.42711500	-3.20120700
C	-3.50132600	3.07250400	-1.29814200
C	-2.71819400	-3.50226100	-5.31140900
C	-5.79406900	-5.35444900	-1.95836200
C	-3.90652700	4.40530900	-1.53253400
C	-5.19330900	4.79388300	-1.05772300
C	-5.90643000	3.96220600	-0.14433400
C	-5.49519000	2.62142600	-0.03270800
C	-4.87588200	-0.95967000	1.73232100
C	-5.93895100	-1.51169400	2.48428400
C	-7.24264900	-1.43330500	1.92427900
C	-7.40891600	-1.12496700	0.46408500
C	-6.30172900	-0.56415800	-0.18642900
C	0.91307800	-3.19357000	1.44070300
C	1.78494500	-3.58291300	2.46566500
C	1.51038800	-3.03000800	3.83273700
C	0.62899900	-1.92310600	3.98071200
C	-0.17721500	-1.58031900	2.87197100
C	0.07187800	-0.67736900	-2.05980100
C	0.80181400	-0.73115000	-3.26683700
C	1.38730900	-1.97555800	-3.62079800
C	0.89948200	-3.24127900	-2.96703100
C	0.13041400	-3.08114200	-1.80277100
O	2.49956400	-1.98997700	-4.47200500
O	-8.35785800	-1.47783300	2.76588100
O	1.95136400	-3.73481000	4.95471400
O	-5.79857100	5.94124500	-1.58615800
C	1.05074000	-4.77204200	5.34469300
H	1.46300200	-5.28943100	6.23475100
H	0.92889200	-5.51739000	4.53172800
H	0.05813600	-4.35229100	5.61223900
C	-8.86782000	-0.17685100	3.07279300
H	-9.73486300	-0.28245500	3.75608800
H	-9.20603500	0.34955900	2.15819900
H	-8.09590800	0.44342900	3.57627100
C	3.75068200	-2.15552000	-3.79170000
H	3.63522900	-2.32304000	-2.69831500
H	4.37910500	-1.25483300	-3.93763000
H	4.29923800	-3.01508800	-4.22567800
C	0.49881300	-1.07770900	5.27910000
C	2.97210300	-4.51432400	2.11601100
C	3.78356800	-3.91595300	0.93814000
H	4.63781400	-4.57579800	0.67750300
H	4.18626400	-2.91845500	1.20593900
H	3.17995000	-3.80523900	0.01574100

C	3.96572100	-4.71266100	3.28475400
H	3.53443300	-5.34944600	4.08385700
H	4.27829700	-3.73397700	3.70815400
H	4.88299600	-5.23737300	2.93881500
C	2.43502200	-5.89924500	1.69728200
H	1.84660200	-6.35684400	2.51923800
H	3.27289700	-6.58600100	1.44799000
H	1.77947900	-5.82842600	0.80479900
C	-0.92134500	-1.23011600	5.85930100
H	-1.00793100	-0.70682200	6.83629700
H	-1.17094300	-2.30103900	6.01249600
H	-1.67517800	-0.78561800	5.18097000
C	1.51284700	-1.44871300	6.38962800
H	1.26601900	-2.42244600	6.85987300
H	1.48764200	-0.70360400	7.21453400
H	2.54978800	-1.46594100	5.98972600
C	0.74384000	0.41434700	4.94726200
H	1.75107600	0.54941400	4.49979300
H	-0.01456800	0.81440400	4.24501500
H	0.68679700	1.04226500	5.86279100
C	0.95533300	0.52109600	-4.17088000
C	1.21282800	-4.66657800	-3.49653900
C	0.05760100	1.70112600	-3.71834000
H	0.38331100	2.09569100	-2.73431300
H	-1.00686200	1.38913400	-3.65557500
H	0.11669400	2.54525300	-4.43947900
C	2.41516900	1.02459600	-4.17248900
H	2.48112100	2.05694200	-4.57964200
H	3.06292600	0.40238300	-4.81843200
H	2.82686300	1.03465000	-3.14132000
C	0.52463200	0.16711000	-5.61333800
H	1.17449100	-0.61136000	-6.06168700
H	0.58127400	1.05982500	-6.27340400
H	-0.52145500	-0.20901600	-5.62255900
C	2.36968300	-5.27281700	-2.67939600
H	3.29167200	-4.66765900	-2.76816400
H	2.60263000	-6.30064500	-3.03287900
H	2.09789900	-5.33095000	-1.60468100
C	1.58816500	-4.66539400	-4.99951000
H	1.57346000	-5.69413900	-5.42005200
H	2.61556900	-4.29763300	-5.17469300
H	0.87047200	-4.04860900	-5.58187300
C	-0.01081100	-5.61021700	-3.34997400
H	-0.90442000	-5.16963100	-3.82563500
H	-0.24314100	-5.84371500	-2.29106900
H	0.18099600	-6.59178500	-3.83529700
C	-2.95428600	5.42496400	-2.21897100
C	-7.07567900	4.45079700	0.75444200
C	-2.68660000	6.59387100	-1.24519700
H	-3.61835500	7.14637300	-1.00440100
H	-1.97041800	7.32103300	-1.68607400
H	-2.25881600	6.21296600	-0.29278100
C	-3.56597000	5.97153000	-3.52937600
H	-2.78847700	6.43326200	-4.17622800
H	-4.30034000	6.77689200	-3.33638400

H	-4.05044300	5.15398600	-4.10565100
C	-7.19025500	5.99509900	0.84122900
H	-6.19730800	6.45238800	1.04191600
H	-7.87514100	6.30160800	1.66135700
H	-7.62733700	6.43726900	-0.07174800
C	-8.40200500	3.87458500	0.22358000
H	-9.25445500	4.20684400	0.85479100
H	-8.38178900	2.76391400	0.22860300
H	-8.58730600	4.21062300	-0.81754200
C	-6.86473300	3.95775200	2.21340400
H	-7.61598300	4.40141200	2.90222200
H	-5.85234800	4.24127100	2.57474500
H	-6.98311600	2.85959800	2.31341100
C	-8.66651200	-1.46858100	-0.36720000
C	-5.69507500	-2.17562400	3.86755900
C	-9.29322900	-0.17816800	-0.93765200
H	-10.20522400	-0.41449700	-1.52763900
H	-8.58873600	0.35567700	-1.60914900
H	-9.58243300	0.51445100	-0.12141100
C	-8.25660400	-2.38845400	-1.53908900
H	-7.55133400	-1.89017400	-2.23622000
H	-9.14184200	-2.69687800	-2.13658200
H	-7.76917200	-3.29939000	-1.13856000
C	-9.75147900	-2.22610400	0.43465700
H	-10.25248100	-1.56835200	1.17172700
H	-9.31814500	-3.10998700	0.95063300
H	-10.55533100	-2.59311300	-0.24005700
C	-6.35368600	-1.36208300	5.00435100
H	-5.99458500	-1.70670800	5.99855700
H	-7.45356800	-1.48423200	5.02431600
H	-6.10377100	-0.28391600	4.90442600
C	-6.27354600	-3.60815000	3.85259600
H	-7.37212100	-3.60609500	3.69882000
H	-6.07570700	-4.12491200	4.81679900
H	-5.81059900	-4.20277900	3.03512800
C	-4.19138100	-2.29883500	4.21555000
H	-3.74119800	-1.29264700	4.33805700
H	-3.64412200	-2.85696800	3.42943200
H	-4.04364700	-2.84423100	5.17318800
C	-1.57874500	4.81272400	-2.58736200
H	-0.89742500	5.58127200	-3.01351900
H	-1.69999200	4.01435200	-3.35015900
H	-1.07722600	4.39343300	-1.69206400
C	-6.78124200	5.65825700	-2.59599200
H	-6.89711100	4.57034500	-2.80758500
H	-6.50068700	6.16227500	-3.54179400
H	-7.76569400	6.06343100	-2.29003000
C	2.93809000	1.87283300	-0.11518100
H	2.12323500	1.43122600	-0.68787400
C	4.21761200	1.23855800	-0.27434900
C	5.42528800	1.90430200	0.02312400
C	6.64790900	1.27777400	-0.13222300
C	6.68242400	-0.08194600	-0.60294400
C	5.46915600	-0.74297400	-0.86812800
C	4.26188000	-0.09797200	-0.71585900

H	5.40539400	2.94021700	0.33112200
H	5.50140700	-1.76807300	-1.20533300
H	3.34048200	-0.62083700	-0.93821900
O	7.86116200	-0.63859800	-0.76060600
O	2.89419500	3.23186000	-0.00517700
C	2.06876400	3.90353800	-1.01022600
H	1.04605500	3.52828500	-0.91746600
H	2.45726600	3.63242800	-1.99451900
C	2.11140600	5.38514500	-0.79914500
C	1.01656100	6.05851500	-0.25399200
C	3.23886000	6.11960000	-1.17412300
C	1.03902500	7.44016500	-0.09049200
C	3.26964000	7.49775600	-1.00008700
C	2.16963300	8.16050200	-0.46095600
H	0.12354000	5.50507900	0.01468500
H	4.09019900	5.61319000	-1.61363000
H	0.17224900	7.95530400	0.30500200
H	4.14493700	8.05928900	-1.30057100
H	2.18931600	9.23648700	-0.34418500
Si	8.57568900	-1.99241000	-1.60307700
C	10.17737700	-1.26036400	-2.24471100
H	9.96976000	-0.46162800	-2.95984600
H	10.77845800	-0.84063400	-1.43750700
H	10.77628100	-2.01668700	-2.75640600
C	7.45474300	-2.49184000	-3.03777200
H	8.03624100	-3.08451100	-3.74806300
H	6.59777700	-3.09676900	-2.73899600
H	7.08820400	-1.61460000	-3.57552400
C	8.81367700	-3.38134400	-0.30828200
C	9.69341100	-2.88101400	0.85255000
C	9.50673300	-4.57420900	-0.99994700
C	7.45330200	-3.84095700	0.24628700
H	9.23753100	-2.03756300	1.37570800
H	9.83797600	-3.68249200	1.58391300
H	10.68427200	-2.57283400	0.51242900
H	9.65609800	-5.38445600	-0.27962400
H	8.91203700	-4.97951500	-1.82246200
H	10.49087400	-4.30929800	-1.39286200
H	7.59636300	-4.64608200	0.97392500
H	6.92569500	-3.03346300	0.76051000
H	6.80325400	-4.23381700	-0.53995400
O	7.80826200	1.91234200	0.04647200
Si	8.62738800	2.58478700	1.40677200
C	9.06384100	1.16689900	2.56549300
H	9.71779100	0.44121600	2.08107600
H	9.57407400	1.53766900	3.45754700
H	8.16464500	0.64161500	2.89736600
C	7.44611600	3.77353700	2.28533300
H	7.11230800	4.58226100	1.63257000
H	6.56705900	3.24483900	2.66265100
H	7.94271000	4.22563700	3.14694300
C	10.13257400	3.45985700	0.62945200
C	9.65802400	4.52113200	-0.38209900
C	10.95359600	4.14395300	1.74170400
C	11.01893900	2.42910100	-0.09628900

H	9.05402900	5.29975200	0.09115600
H	10.52204000	5.01642100	-0.83580900
H	9.07129900	4.07828300	-1.18992500
H	10.38012500	4.90861700	2.27173800
H	11.32800100	3.43009200	2.47961900
H	11.82530800	4.64170100	1.30598800
H	11.87327700	2.93342300	-0.55849000
H	11.42042200	1.67909800	0.58942600
H	10.47331000	1.90990800	-0.88647700

(*e*) Transition state **TS_{15}-A2**, using acetal **15**

Ni	-1.21902500	0.39348400	0.70298900
S	-0.60070700	2.19558100	1.85630700
C	1.05064900	2.01967900	2.21466200
O	1.62357200	3.02799500	2.81638100
C	3.05960700	2.76707400	2.98520400
C	3.17546000	1.26178400	2.77045400
N	1.89596700	0.97126500	2.05321500
C	1.54096700	-0.29486100	1.58366700
O	0.43254700	-0.47824400	1.01089900
H	3.33199100	3.10214700	3.98097300
H	3.57669100	3.35017900	2.22656000
H	3.18952800	0.70195200	3.70642300
H	4.02819300	0.99970100	2.15178500
C	2.50960800	-1.34022000	1.64907500
H	3.37557800	-1.16497900	2.27482000
N	1.93226100	-2.64169300	1.75002800
N	2.59574700	-3.46413100	2.39770000
N	3.09118300	-4.30146100	2.96462400
P	-2.90482300	1.52255900	-0.30237200
P	-1.95271200	-1.69613600	0.17527900
C	-3.28637200	0.88311600	-1.97911000
C	-2.40128200	3.27712100	-0.49949100
C	-4.52754200	1.56654400	0.51843900
C	-3.75605800	-1.89807700	0.01902800
C	-1.39977700	-2.81000000	1.52400400
C	-1.29710700	-2.32970600	-1.40522600
C	-4.51696700	-1.27781800	-1.11285200
C	-4.45770800	-2.71785800	0.92353400
C	-3.87674200	-0.48221800	-2.19170300
C	-3.98064100	-0.92228600	-3.52035700
C	-5.82354400	-2.95835600	0.74452300
C	-6.47857400	-2.39116900	-0.34616500
C	-5.87919900	-1.60832500	-1.20777000
C	-3.64122200	-0.18012000	-4.54214300

C	-3.17125700	1.12132400	-4.40527800
O	-7.79113300	-2.63434500	-0.69551600
O	-6.73592200	-1.23291000	-2.22648700
O	-4.48814100	-2.13899500	-3.93051800
O	-3.87215600	-0.81697000	-5.74387000
H	-3.95883000	-3.19016200	1.75709900
H	-6.35845000	-3.60501000	1.42766600
H	-2.93688600	1.71228700	-5.28109700
H	-2.65636500	2.67350600	-3.05563600
H	-3.90972100	4.05792500	0.79267400
H	-0.64877300	2.72030300	-1.53457300
H	-3.86036000	0.38915400	2.13778600
H	-5.42797900	2.62069000	-1.09801500
H	-1.27932700	-4.57885700	0.33518000
H	-1.33661000	-1.18702500	2.89667400
H	-2.51876100	-4.06762400	-1.38487300
H	-0.24235900	-0.53383300	-1.75432000
H	-3.75967300	-2.90118100	-5.73856600
H	-5.43373600	-2.20442800	-5.79339600
H	-8.20070400	-2.63168500	-2.74465500
H	-8.79468300	-1.19345900	-1.82578500
C	-3.00704800	1.65860500	-3.12517000
C	-1.20175700	3.56363500	-1.15052600
C	-4.41641600	-2.08855600	-5.36401500
C	-7.96277700	-1.91858100	-1.92729600
C	-0.74213400	4.89000100	-1.30997500
C	-1.57103100	5.93682800	-0.80895900
C	-2.64236500	5.64192900	0.08731300
C	-3.07375400	4.30458800	0.16245100
C	-4.70184300	0.94523200	1.75429700
C	-5.90313400	1.07366500	2.49072000
C	-6.90346200	1.93524000	1.96283000
C	-6.82443600	2.38065600	0.53048200
C	-5.58506800	2.22628800	-0.10343900
C	-1.15769700	-4.16778400	1.31340200
C	-0.71991200	-5.01155900	2.34425000
C	-0.66473000	-4.42256800	3.72357600
C	-0.73659100	-3.01222200	3.88967500
C	-1.17079500	-2.24970400	2.78436800
C	-0.46063800	-1.50915400	-2.16113600
C	0.07082300	-1.92165600	-3.40354200
C	-0.15250500	-3.26961700	-3.79198200
C	-1.24740000	-4.06226500	-3.12988000

C	-1.75071500	-3.54172800	-1.92739200
O	0.72277300	-3.87707100	-4.70121700
O	-7.85951900	2.48754000	2.82014000
O	-0.76164200	-5.26601700	4.83222800
O	-1.35987400	7.24394700	-1.25774900
C	-2.11315900	-5.57860200	5.17094600
H	-2.11579100	-6.25099800	6.05283500
H	-2.62107800	-6.09901900	4.33226000
H	-2.67740600	-4.65813700	5.43079900
C	-7.52732500	3.81648300	3.23291200
H	-8.31087400	4.18411600	3.92625300
H	-7.47572600	4.50613000	2.36768000
H	-6.55087400	3.83598800	3.76216400
C	1.67923500	-4.75613800	-4.09423100
H	1.55218600	-4.85328400	-2.99260700
H	2.70270900	-4.38462200	-4.29428900
H	1.60347800	-5.76301500	-4.55030900
C	-0.37472600	-2.26655500	5.20440700
C	-0.29524700	-6.45850400	1.99036700
C	0.77482700	-6.43024700	0.87028200
H	1.11868900	-7.45776400	0.62205000
H	1.65611500	-5.84303400	1.19706000
H	0.39161700	-5.98785600	-0.07159200
C	0.32495200	-7.22924500	3.17945900
H	-0.44015000	-7.49568400	3.93679500
H	1.13819700	-6.63797800	3.65291700
H	0.76411900	-8.19252800	2.83989500
C	-1.52259000	-7.24937400	1.49040600
H	-2.31312000	-7.27798300	2.26906400
H	-1.24084800	-8.29594700	1.24328100
H	-1.95685400	-6.79278800	0.57694500
C	-1.62615900	-1.56312100	5.76737500
H	-1.41118500	-1.10664900	6.75795900
H	-2.46163200	-2.28424700	5.88856300
H	-1.95796000	-0.74691900	5.09588200
C	0.20510700	-3.17648400	6.31542600
H	-0.57623400	-3.82659900	6.75969900
H	0.60431200	-2.56907500	7.15681900
H	1.04269300	-3.79403200	5.92486100
C	0.70556100	-1.19772100	4.90649300
H	1.61188700	-1.67307900	4.47573300
H	0.34216700	-0.42395000	4.20067200
H	1.00483100	-0.66339600	5.83419900

C	0.84324100	-0.93213700	-4.31842200
C	-1.82507600	-5.38595300	-3.69676400
C	0.73529600	0.53677500	-3.83644800
H	1.26302600	0.68041400	-2.87144400
H	-0.32608800	0.83987900	-3.72227900
H	1.20546000	1.23155500	-4.56611100
C	2.34425600	-1.28266500	-4.37739800
H	2.91567500	-0.47932600	-4.89130700
H	2.53073200	-2.20719700	-4.95266200
H	2.75926300	-1.39312100	-3.35450600
C	0.24467600	-0.97437200	-5.74404200
H	0.38044200	-1.96471500	-6.22308300
H	0.73600700	-0.22602200	-6.40313600
H	-0.84348900	-0.75019500	-5.71250800
C	-1.20191700	-6.57590200	-2.94237900
H	-0.10308900	-6.60592700	-3.07116100
H	-1.61598700	-7.53732900	-3.31629300
H	-1.41731400	-6.50481200	-1.85517200
C	-1.56244600	-5.54123700	-5.21565400
H	-2.17686000	-6.35937000	-5.64983300
H	-0.51633100	-5.82090600	-5.43471800
H	-1.81459900	-4.60277500	-5.75453700
C	-3.36441800	-5.45868900	-3.50935500
H	-3.85220800	-4.56142200	-3.93131700
H	-3.65866000	-5.56343400	-2.44529800
H	-3.78862300	-6.34848700	-4.02320100
C	0.64902300	5.18676100	-1.94131600
C	-3.30231200	6.66804600	1.05162000
C	1.50304500	6.00160000	-0.94354800
H	1.05340200	6.99100400	-0.72450200
H	2.51959600	6.18715300	-1.35412500
H	1.60794900	5.44892900	0.01556000
C	0.50998900	5.97100600	-3.26477400
H	1.46916200	5.97626300	-3.82710800
H	0.25951400	7.03539000	-3.09427400
H	-0.26420400	5.50561100	-3.91196800
C	-2.65519800	8.07542100	1.03476900
H	-1.55477200	8.00759800	1.17456300
H	-3.05551900	8.70432000	1.85962400
H	-2.88853500	8.62881000	0.10493400
C	-4.80123600	6.81809500	0.71202900
H	-5.27990900	7.57248200	1.37342800
H	-5.34667100	5.86136200	0.84947300

H	-4.93692200	7.14320400	-0.33978700
C	-3.16954900	6.15078400	2.50824800
H	-3.56865200	6.89168400	3.23471500
H	-2.10192300	5.96202400	2.75434100
H	-3.73723800	5.21290300	2.67729400
C	-8.02278000	2.90407700	-0.29610300
C	-6.12518400	0.30282700	3.82188200
C	-7.76370400	4.35949200	-0.74128600
H	-8.61866900	4.74289100	-1.33937200
H	-6.85193000	4.43746000	-1.36982100
H	-7.63468700	5.02521600	0.13550200
C	-8.19351300	2.02123500	-1.55381800
H	-7.31601200	2.08054300	-2.23048300
H	-9.08069000	2.33345100	-2.14647900
H	-8.33024700	0.96513600	-1.24994200
C	-9.36779600	2.85649600	0.46700000
H	-9.41469700	3.62149700	1.26659200
H	-9.54187900	1.84754300	0.89940200
H	-10.21642300	3.07997700	-0.21561300
C	-6.21563000	1.27054900	5.02327000
H	-6.14667000	0.71718900	5.98515100
H	-7.18252800	1.80808500	5.05669800
H	-5.38396900	2.00685700	4.99134100
C	-7.42854200	-0.52067800	3.72019700
H	-8.31687200	0.13026000	3.58761400
H	-7.59191100	-1.11874100	4.64295900
H	-7.37823700	-1.21768700	2.85548600
C	-4.98350800	-0.69521700	4.13498000
H	-4.03371600	-0.15181900	4.31395700
H	-4.84680700	-1.41725900	3.30593800
H	-5.20395000	-1.28326900	5.05255700
C	1.44798600	3.89938200	-2.26384100
H	2.45707000	4.14444700	-2.65826900
H	0.93236900	3.30340600	-3.04566800
H	1.58713400	3.28203400	-1.35058000
C	-2.26219100	7.61829300	-2.30439800
H	-2.16153200	6.94191200	-3.17937500
H	-2.02724800	8.65233900	-2.62917500
H	-3.31472100	7.59089200	-1.95812800
C	3.38461200	-1.30317800	-0.14801200
H	2.46890400	-1.22478700	-0.73562000
C	4.25937300	-0.13305500	-0.20533700
C	5.59530600	-0.18161900	0.22761000

C	6.41904500	0.93777100	0.16344500
C	5.87673800	2.17142500	-0.33213400
C	4.54635100	2.19778300	-0.76383000
C	3.74339600	1.06810900	-0.69734900
H	6.00571800	-1.11402800	0.58854100
H	4.15572400	3.11242600	-1.18421900
H	2.72635200	1.11229700	-1.06547400
O	6.68359600	3.22227900	-0.37343700
O	3.97740900	-2.50081500	-0.18447600
C	3.29906300	-3.58654200	-0.92971000
H	2.77331600	-3.11568300	-1.76017700
H	2.57067600	-4.04535700	-0.26613200
C	4.31501200	-4.56906500	-1.40870400
C	5.05190500	-4.30801700	-2.56695000
C	4.53096200	-5.75996200	-0.71404700
C	5.98942900	-5.22458400	-3.02304300
C	5.46913500	-6.67891600	-1.17061500
C	6.19807300	-6.41156100	-2.32480400
H	4.88482400	-3.38928000	-3.11839300
H	3.96393000	-5.97615800	0.18399500
H	6.55095900	-5.02089700	-3.92582300
H	5.62651000	-7.60368200	-0.63044500
H	6.92365500	-7.13004900	-2.68454900
Si	6.48686700	4.89737300	-0.77968800
C	5.16848800	5.58778800	0.38974900
H	4.21971700	5.05676500	0.28553100
H	5.48931500	5.52518700	1.43155700
H	4.97319100	6.63907000	0.16748800
C	5.92390400	5.02201100	-2.57508400
H	6.02726000	6.05189200	-2.92530500
H	6.53037000	4.39097400	-3.22726500
H	4.87824200	4.74212100	-2.71244000
C	8.21391800	5.63766600	-0.47814000
C	8.67174500	5.34662900	0.96430000
C	9.22233800	5.02163100	-1.46824400
C	8.14809200	7.16353500	-0.69662700
H	8.71948400	4.27499700	1.16652900
H	9.67153300	5.76134100	1.12580600
H	8.01300800	5.80557100	1.70616900
H	10.21965600	5.43349100	-1.28613400
H	9.28908600	3.93739500	-1.35834200
H	8.96805400	5.24352900	-2.50725100
H	9.13755200	7.60290500	-0.53965400

H	7.84010300	7.42539600	-1.71183800
H	7.46528900	7.65369900	0.00190000
O	7.68405700	0.94855500	0.56905200
Si	9.04270300	-0.12603500	0.56128100
C	10.43836800	0.91973600	-0.13188600
H	11.38462400	0.37464000	-0.11349600
H	10.23256000	1.18931100	-1.16994500
H	10.56887600	1.84185500	0.43587300
C	8.67460300	-1.57709300	-0.58663200
H	7.96688500	-2.29962300	-0.17854000
H	8.28661200	-1.22421900	-1.54504600
H	9.60409800	-2.11309000	-0.79328200
C	9.32555600	-0.64785600	2.38124000
C	9.61520400	0.59593200	3.24368900
C	10.53285700	-1.60614700	2.44566600
C	8.08235400	-1.36621700	2.93928300
H	8.79307000	1.31546100	3.21260100
H	9.76065600	0.30302300	4.28852900
H	10.52284700	1.11222000	2.92510000
H	11.44970900	-1.13781600	2.08064400
H	10.71757500	-1.90624900	3.48192800
H	10.36840100	-2.51956600	1.86869800
H	8.25790200	-1.66764800	3.97711700
H	7.20379700	-0.71399800	2.93499900
H	7.84016800	-2.27346800	2.37917800

6. References

[1] Teloxa, S. F.; Kennington, S. C. D.; Camats, M.; Romea, P.; Urpí, F.; Aullón, G.; Font-Bardia, M. *Chem. Eur. J.* **2020**, *26*, 11540–11548.

[2] Teloxa, S. F.; Mellado-Hidalgo, M.; Kennington, S. C. D.; Romea, P.; Urpí, F.; Aullón, G.; Font-Bardia, M. *Chem. Eur. J.* **2022**, *28*, e202200671.

[3] Mellado-Hidalgo, M.; Conejos-Jalencas, J.; Teloxa, S. F.; Suárez-Herrera, A.; McCall, L.; Costa, A. M.; Romea, P.; Urpí, F.; Aullón, G.; Puigjaner, C. *J. Org. Chem.* **2025**, *90*, 8519–8530.

[4] Liu, X.; Li, X.; Yang, H.; Shi, X.; Yang, F.; Jiao, X.; Xie, P. *Synth. Commun.* **2018**, *48*, 594–600.

[5] Frisch, M. J.; Trucks, G. W; Schlegel, H. B.; Scuseria, G. E.; Robb, M. A; Cheeseman, J. R.; Scalmani, G.; Barone, V.; Mennucci, B.; Petersson, G. A.; Nakatsuji, H; Caricato, M.; Li, X.; Hratchian, H. P.; Izmaylov, A. F.; Bloino, J.; Zheng, G.; Sonnenberg, J. L.; Hada, M; Ehara, M.; Toyota, K.; Fukuda, R.; Hasegawa, J.; Ishida, M.; Nakajima, T.; Honda, Y.; Kitao, O; Nakai, H.; Vreven, T; Montgomery, J. A. Jr.; Peralta, J. E.; Ogliaro, F.; Bearpark, M.; Heyd, J. J.; Brothers, E.; Kudin, K. N.; Staroverov, V. N.; Keith, T.; Kobayashi, R.; Normand, J.; Raghavachari, K.; Rendell, A.; Burant, J. C.; Iyengar, S. S.; Tomasi, J.; Cossi, M.; Rega, N.; Millam, J. M.; Klene, M.; Knox, J. E; Cross, J. B.; Bakken, V.; Adamo, C.; Jaramillo, J.; Gomperts, R.; Stratmann, R. E.; Yazyev, O.; Austin, A. J.; Cammi, R.; Pomelli, C.; Ochterski, J. W.; Martin, R. L.; Morokuma, K.; Zakrzewski, V. G.; Voth, G. A.; Salvador, P.; Dannenberg, J. J.; Dapprich, S.; Daniels, A. D.; Farkas, O.; Foresman, J. B.; Ortiz, J. V.; Cioslowski, J.; Fox, D. J. *Gaussian 09 (Revission B.1)*; Gaussian Inc.: Wallingford CT **2010**.

[6] (*a*) Maseras, F.; Morokuma, K. *J. Comput. Chem.* **1995**, *16*, 1170–1179. (*b*) Dapprich, S.; Komáromi, I.; Byun, K. S.; Morokuma, K.; Frisch, M. J. *J. Mol. Struct.-Theochem.* **1999**, *461*, 1–21.

[7] (*a*) Becke, A. D. *J. Chem. Phys.* **1993**, *98*, 5648–5652. (*b*) Lee, C.; Yang, W.; Parr, R. G. *Phys. Rev. B* **1988**, *37*, 785–789.

[8] Schäfer, A.; Huber, C.; Ahlrichs, R. *J. Chem. Phys.* **1994**, *100*, 5829–5835.

[9] Tomasi, J.; Mennucci, B.; Cammi, R. *Chem. Rev.* **2005**, *105*, 2999–3094.

[10] Llunell, M.; Casanova, D.; Cirera, J.; Alemany, P.; Alvarez, S. *SHAPE (version 2.0)*, Barcelona, **2010**.

7. Copies of the NMR Spectra

^1H NMR (400 MHz, $CDCl_3$)

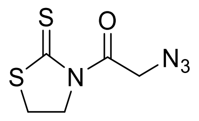

3

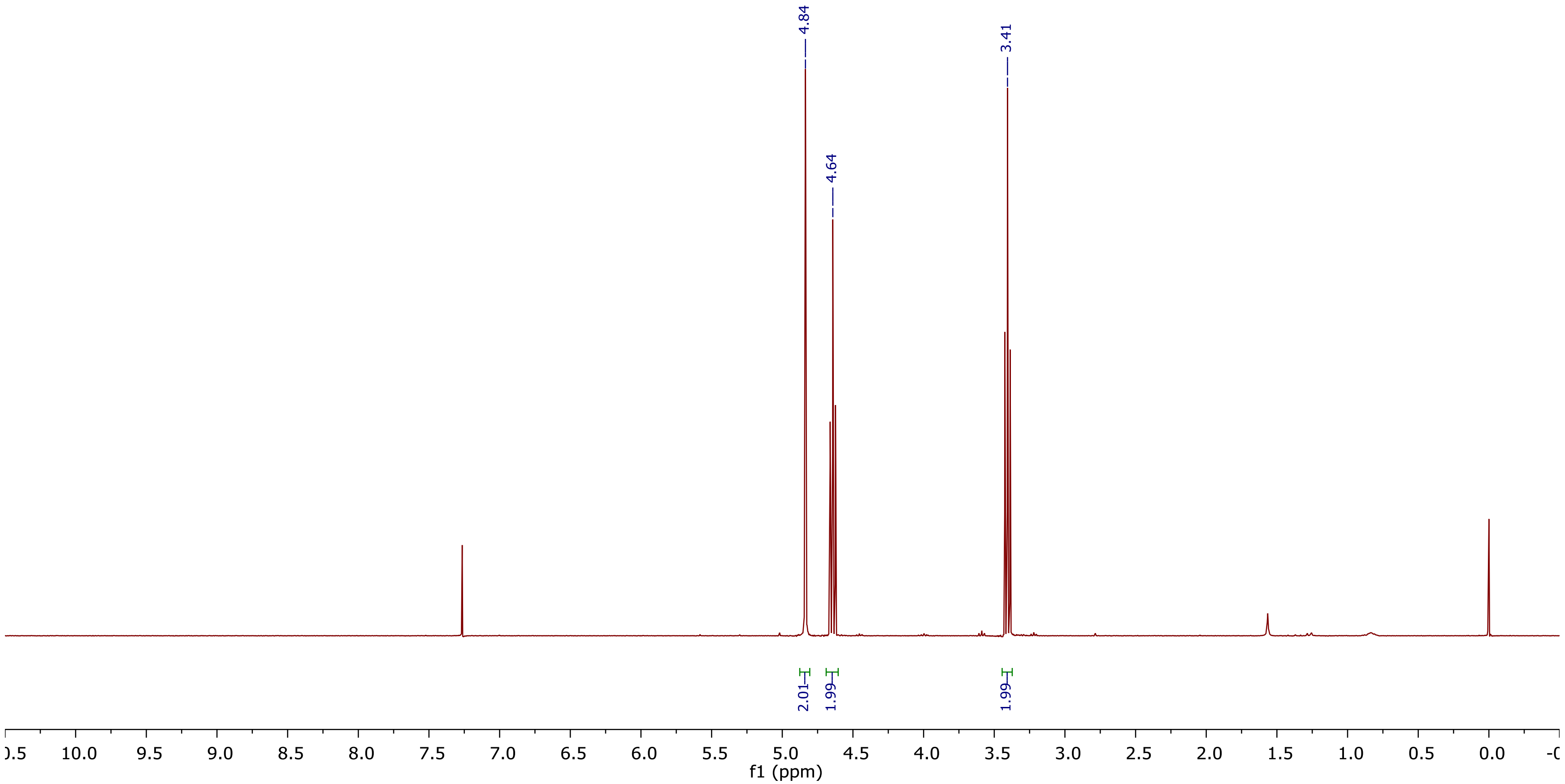

$^{13}C\{^1H\}$ NMR (100.6 MHz, $CDCl_3$)

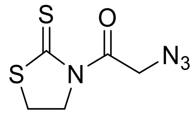

3

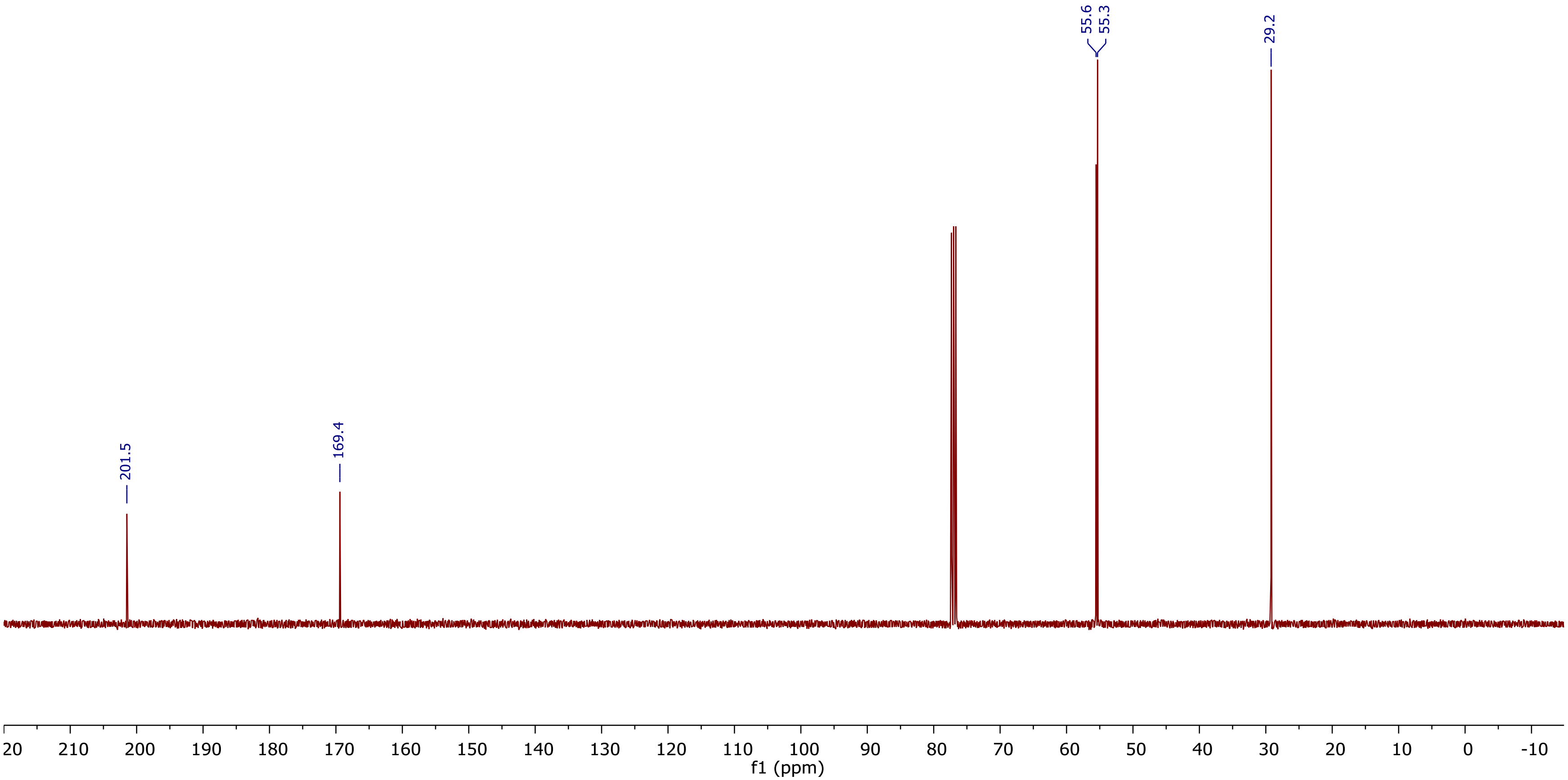

^1H NMR (400 MHz, $CDCl_3$)

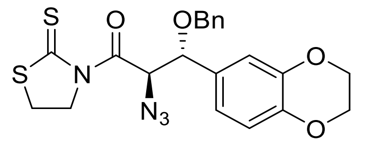

5

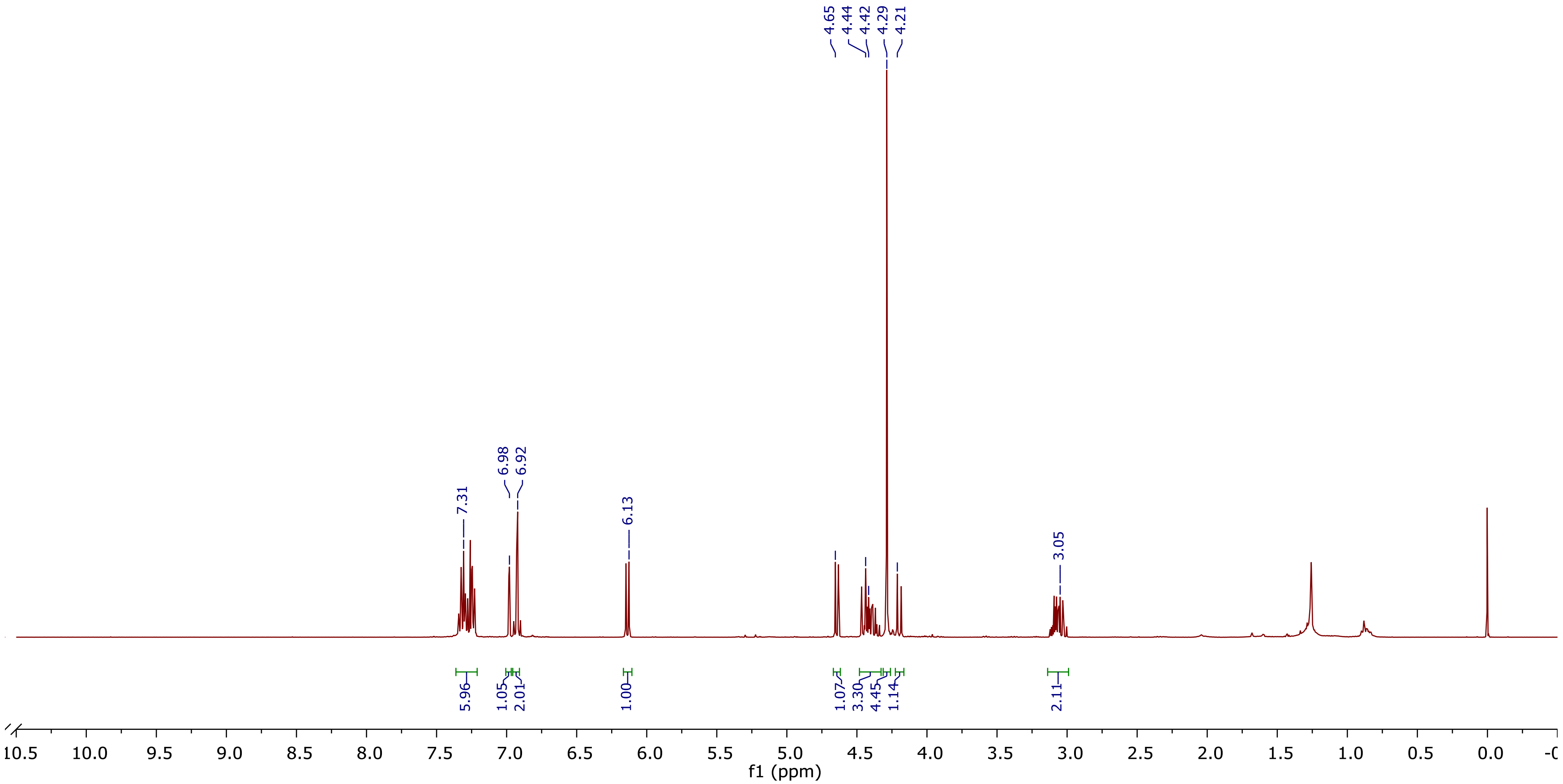

$^{13}C\{^1H\}$ NMR (100.6 MHz, $CDCl_3$)

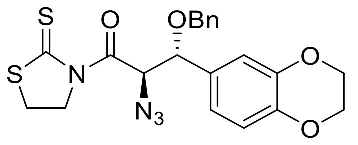

5

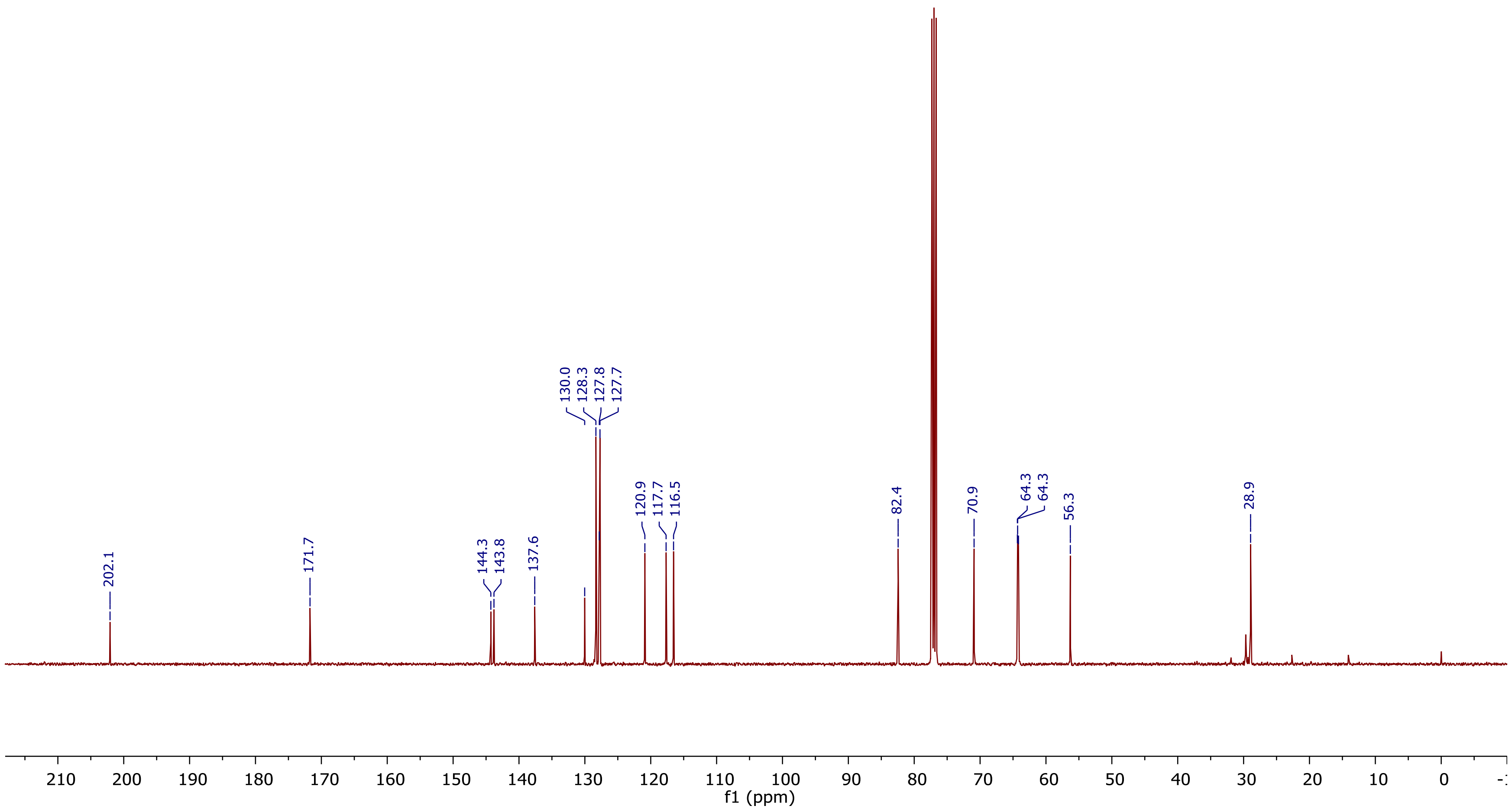

^1H - ^1H COSY NMR (400 MHz, $CDCl_3$)

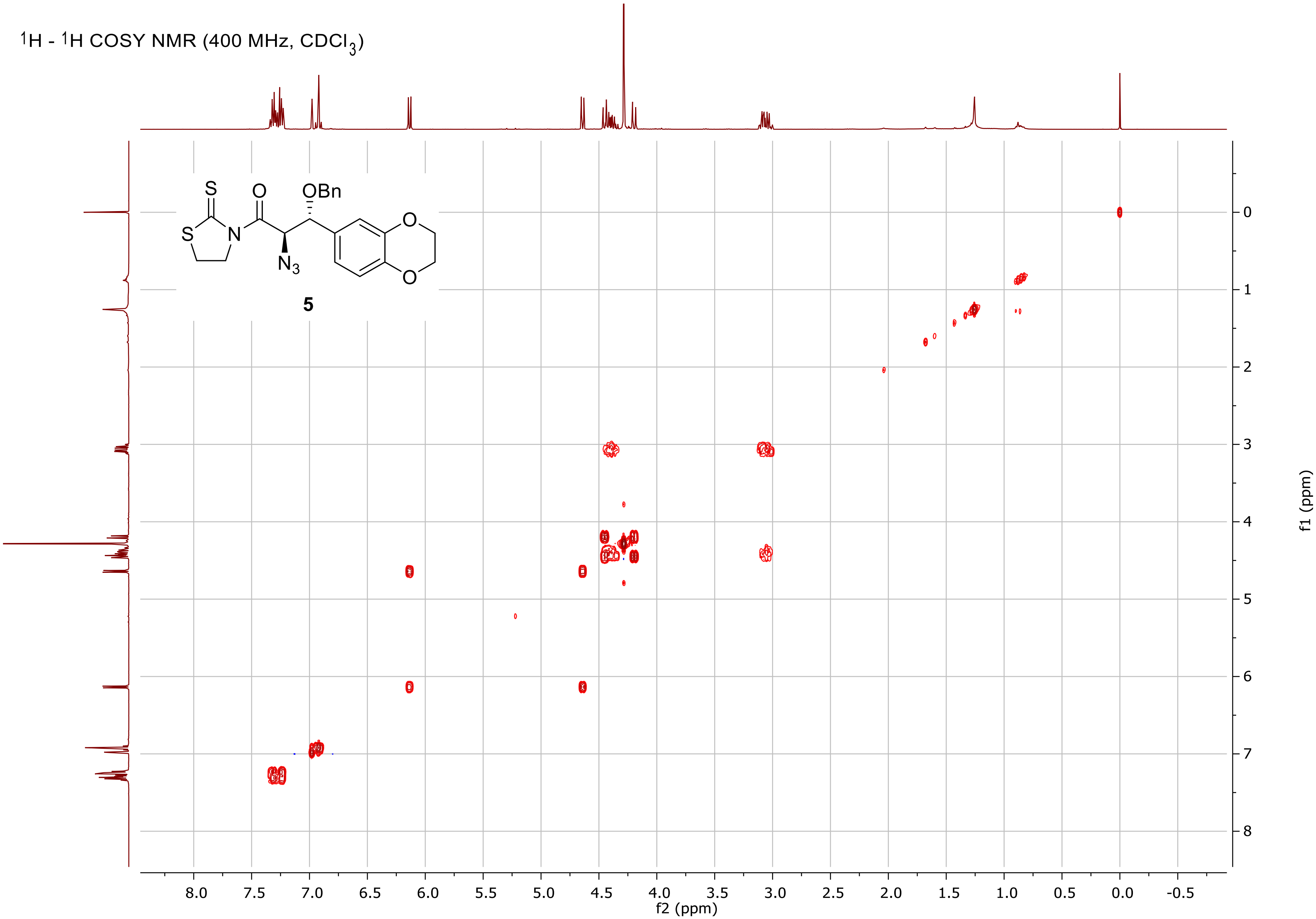

^{1}H - ^{13}C HSQC NMR (400 MHz, $CDCl_3$)

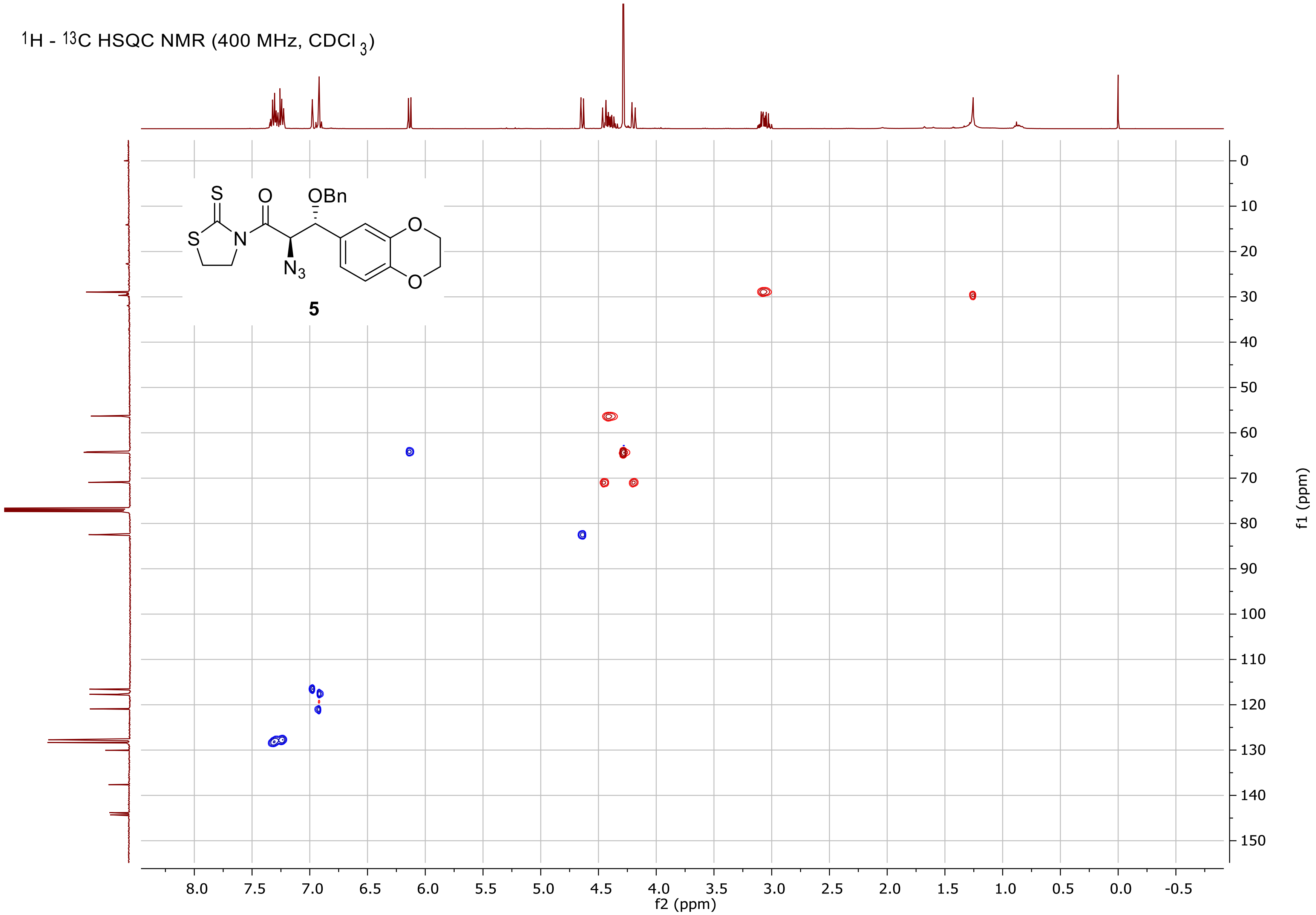

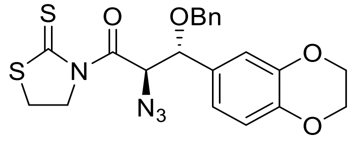

5

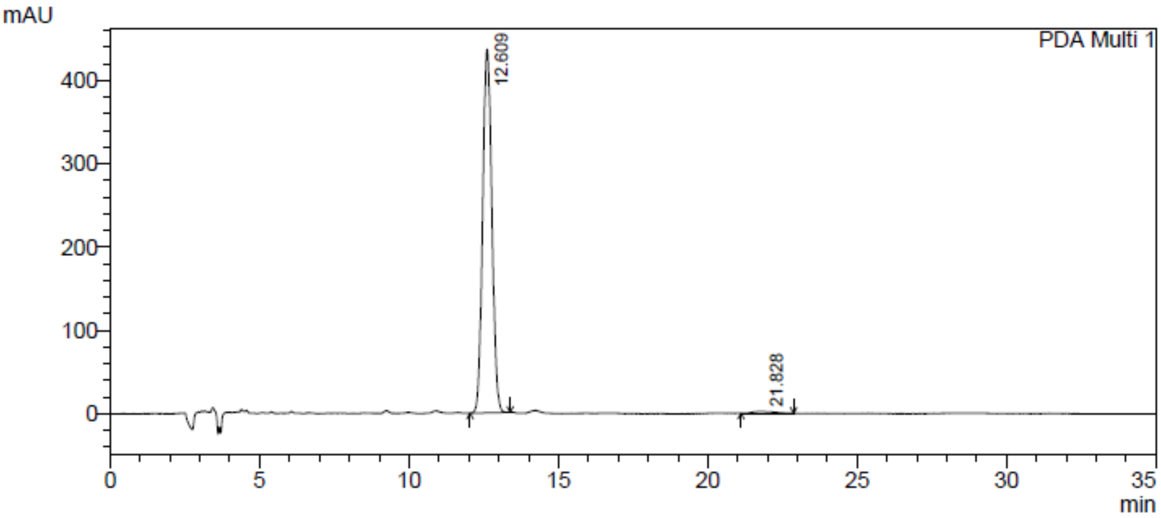

1 PDA Multi 1/254nm 4nm

PeakTable

PDA Ch1 254nm 4nm

Peak#	Ret. Time	Area	Height	Area %	Height %
1	12.609	9366188	436224	98.896	99.474
2	21.828	104528	2305	1.104	0.526
Total		9470716	438530	100.000	100.000

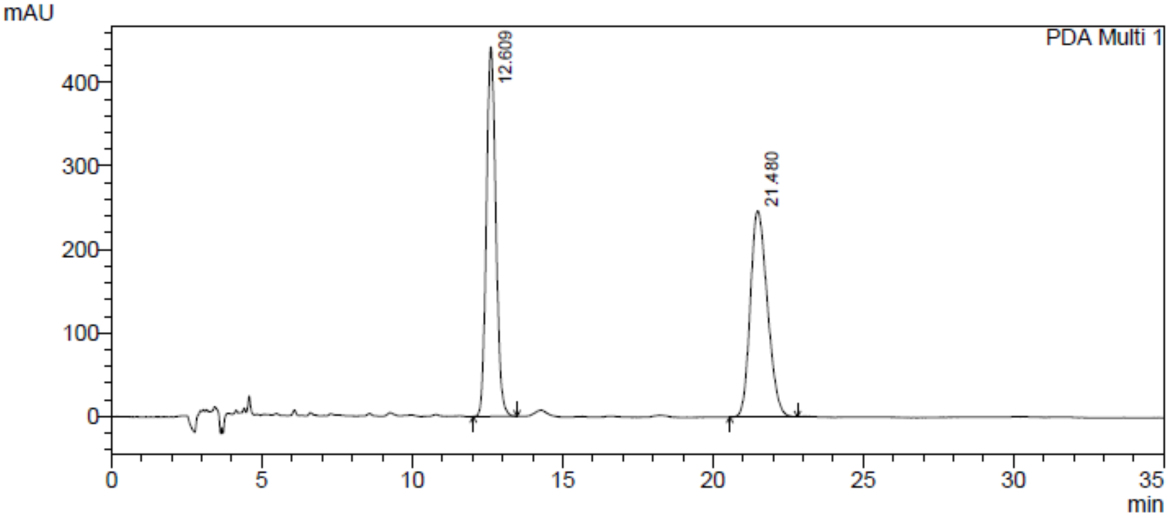

1 PDA Multi 1/254nm 4nm

PeakTable

PDA Ch1 254nm 4nm

Peak#	Ret. Time	Area	Height	Area %	Height %
1	12.609	9670046	442031	49.766	64.172
2	21.480	9760870	246792	50.234	35.828
Total		19430916	688823	100.000	100.000

^1H NMR (400 MHz, $CDCl_3$)

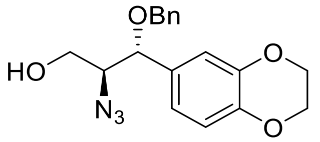

11

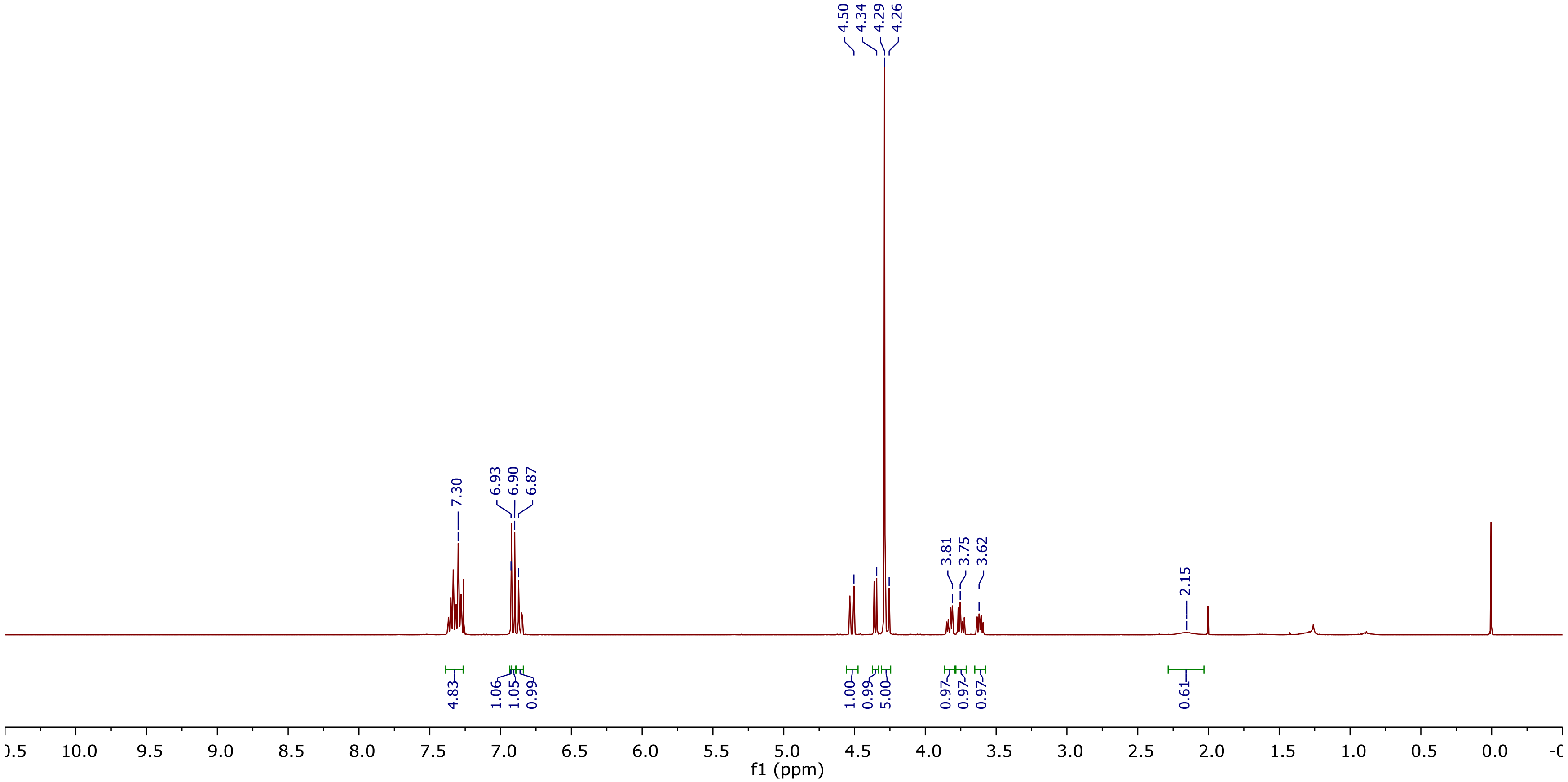

$^{13}C\{^{1}H\}$ NMR (100.6 MHz, $CDCl_3$)

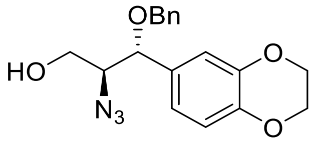

11

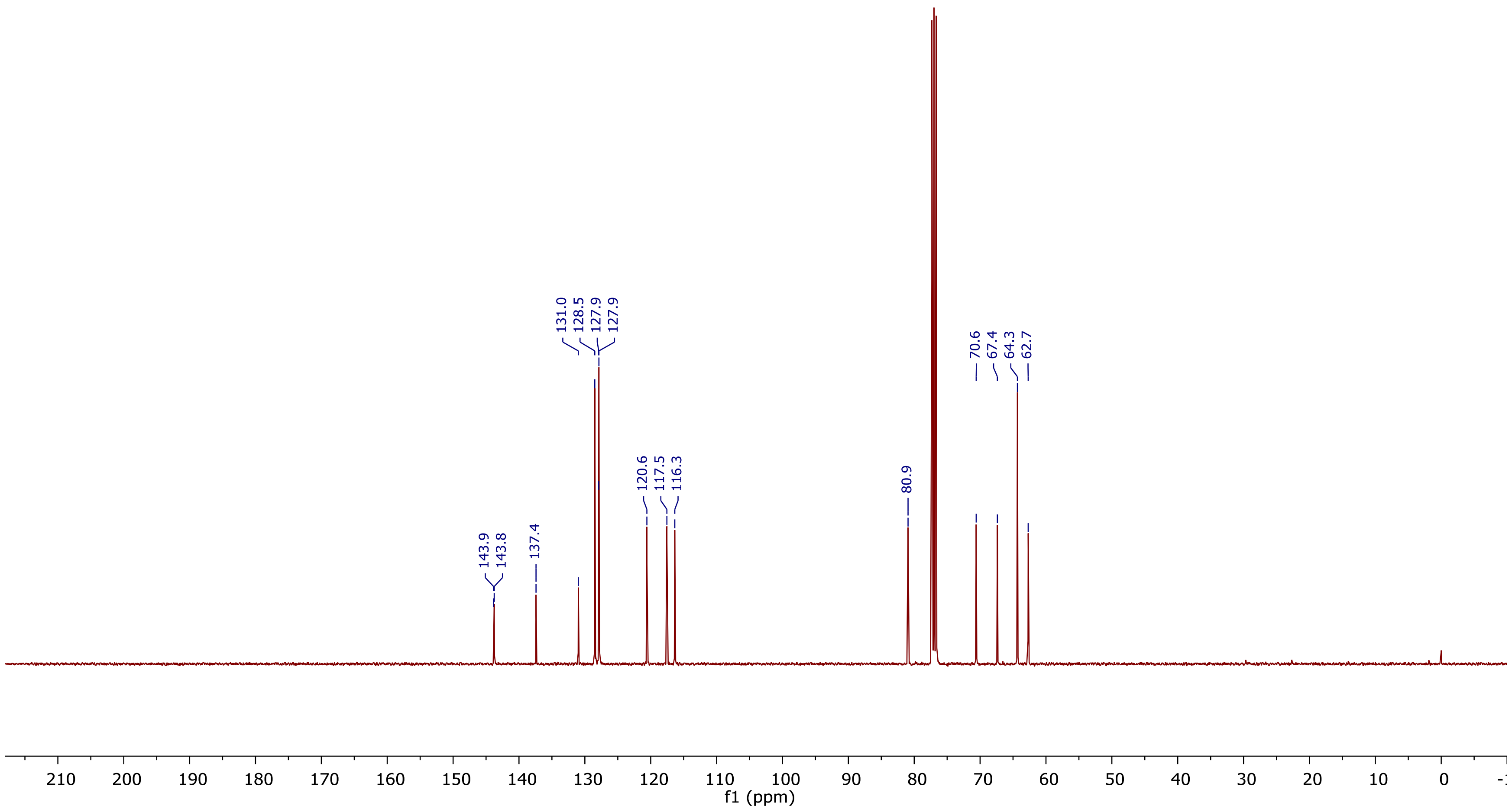

^{1}H - ^{1}H COSY NMR (400 MHz, $CDCl_3$)

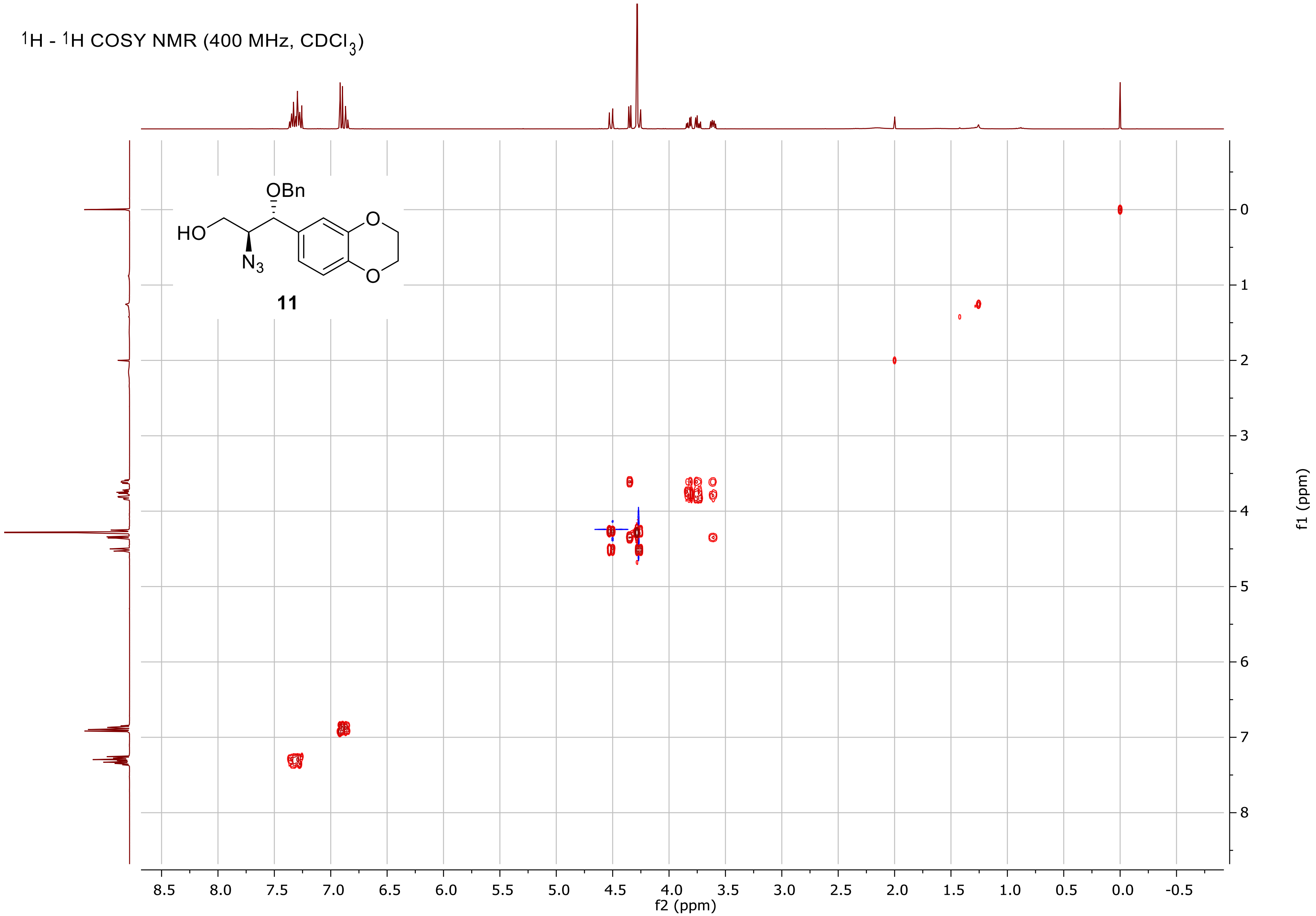

^1H - ^{13}C HSQC NMR (400 MHz, $CDCl_3$)

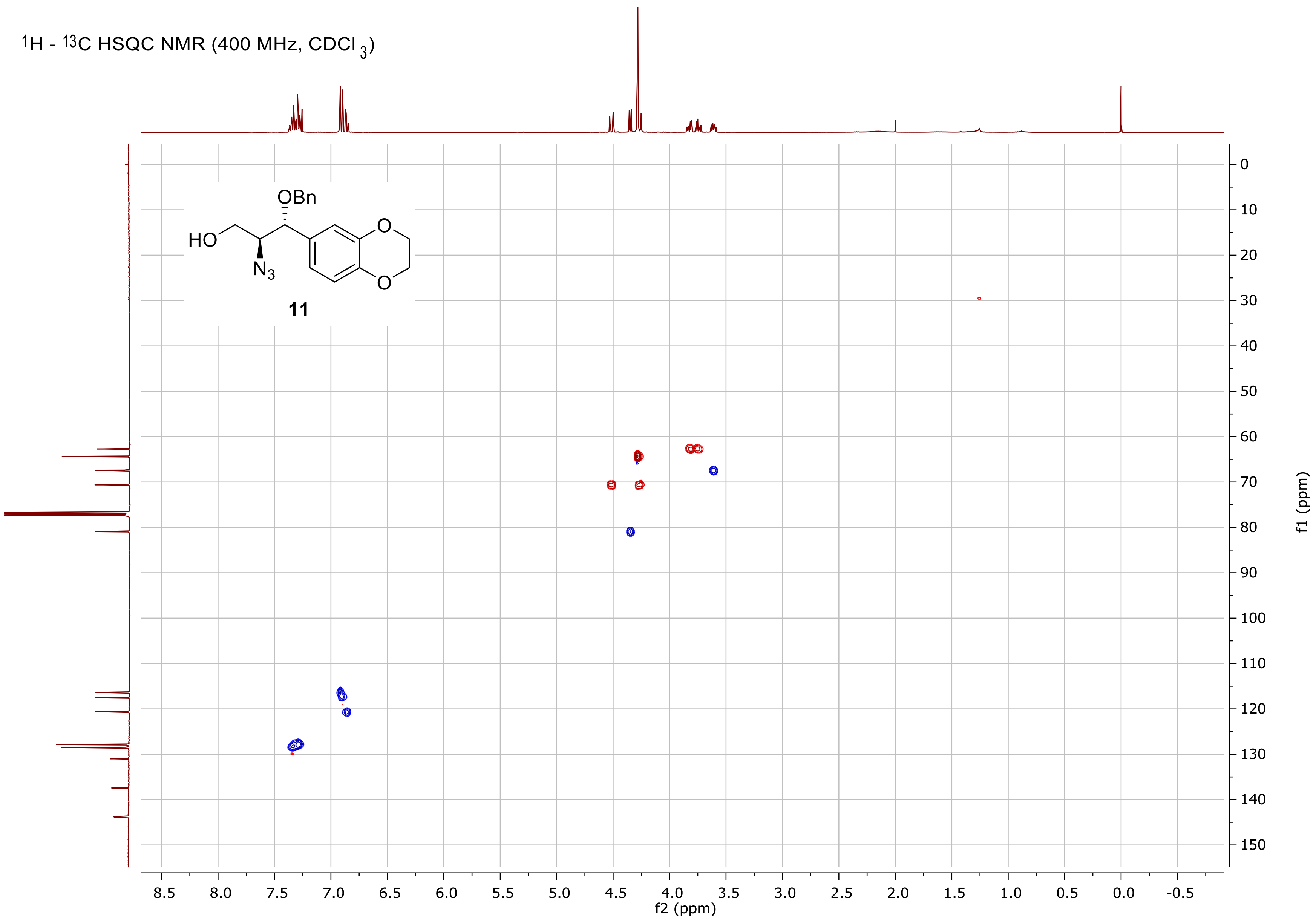

^{1}H NMR (400 MHz, $CDCl_3$)

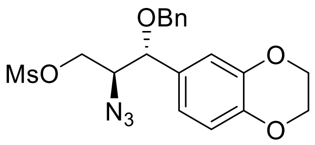

12

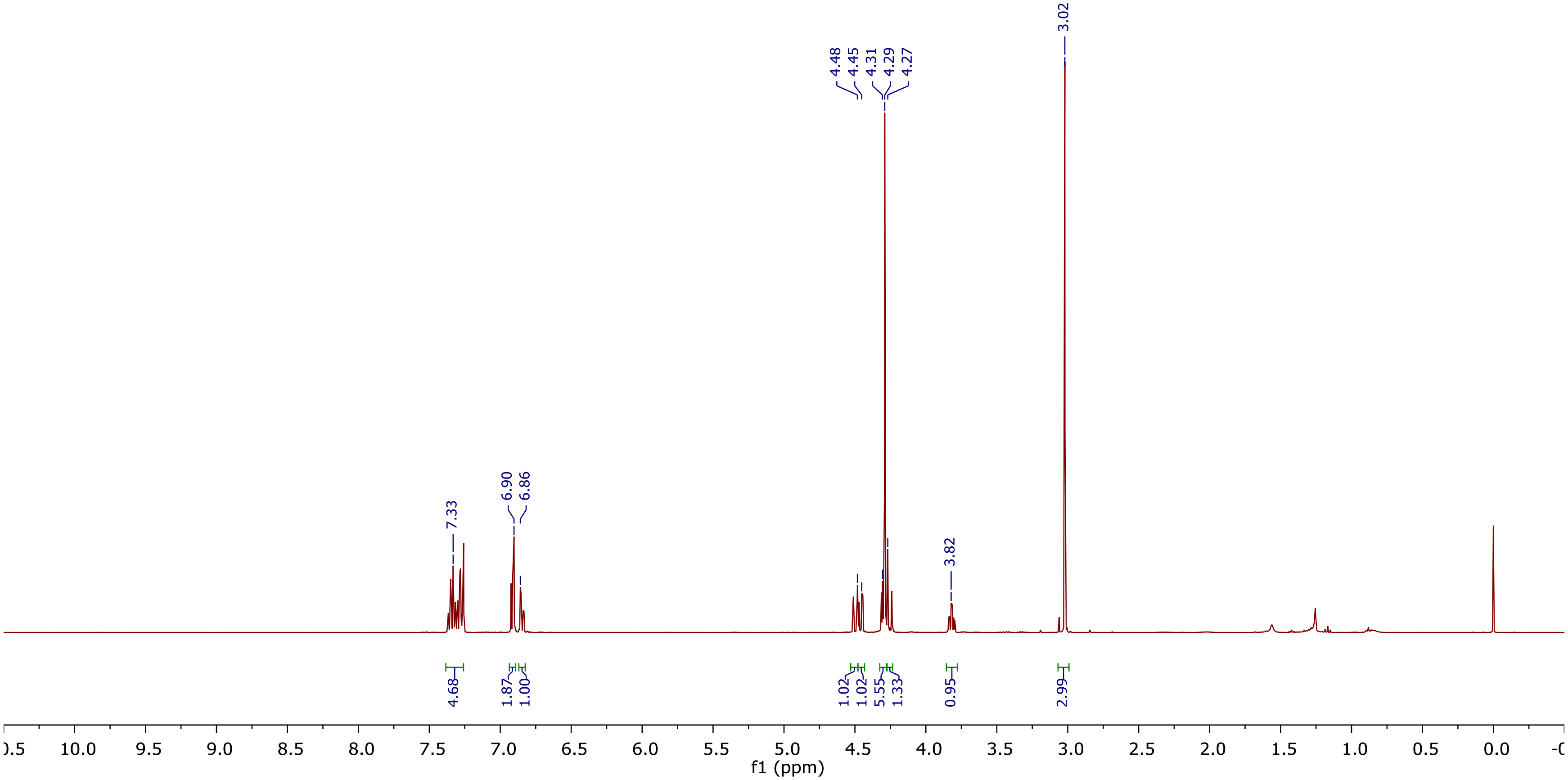

$^{13}C\{^{1}H\}$ NMR (100.6 MHz, $CDCl_3$)

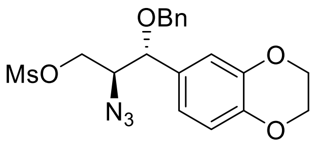

12

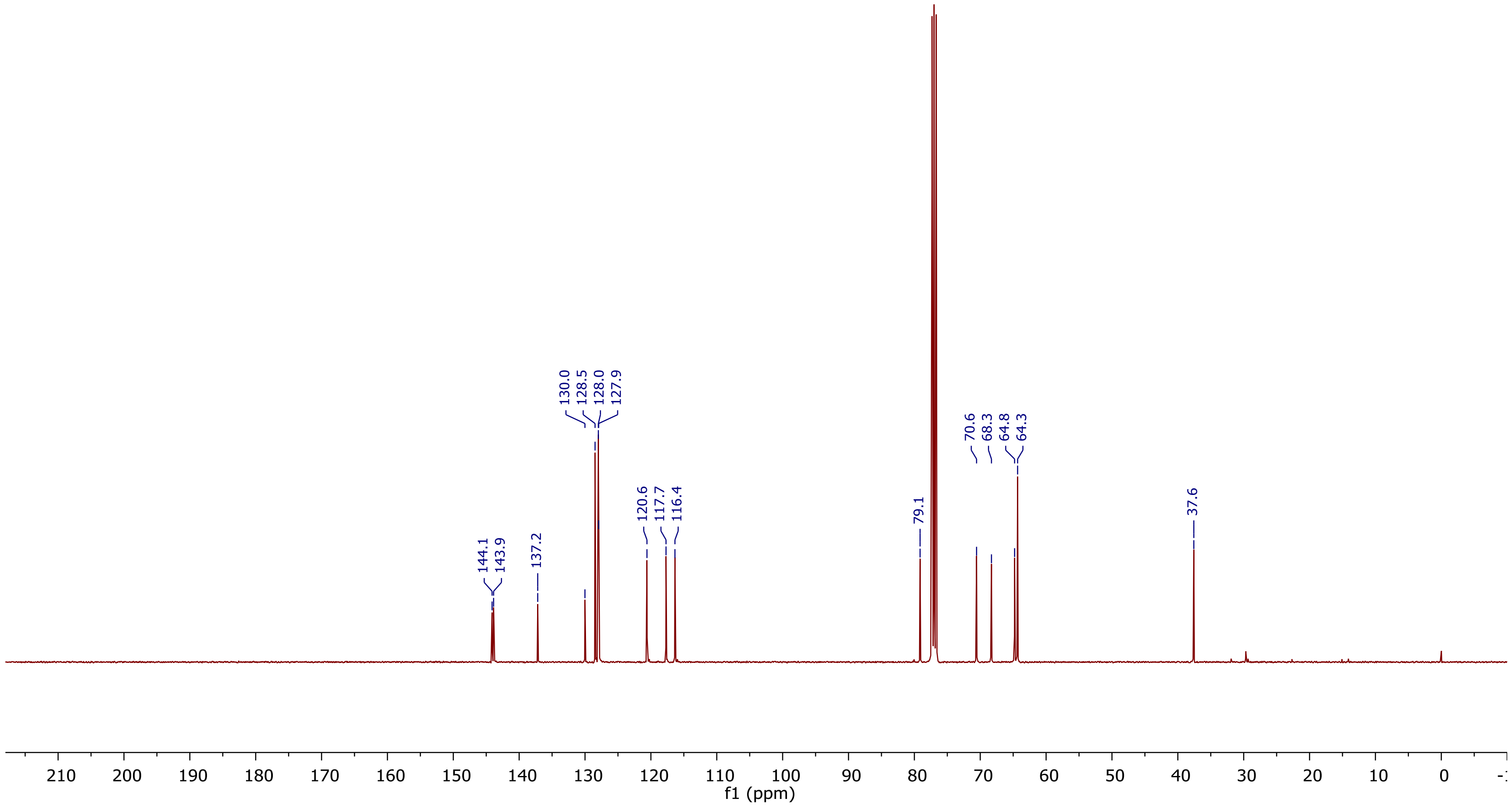

^1H - ^1H COSY NMR (400 MHz, $CDCl_3$)

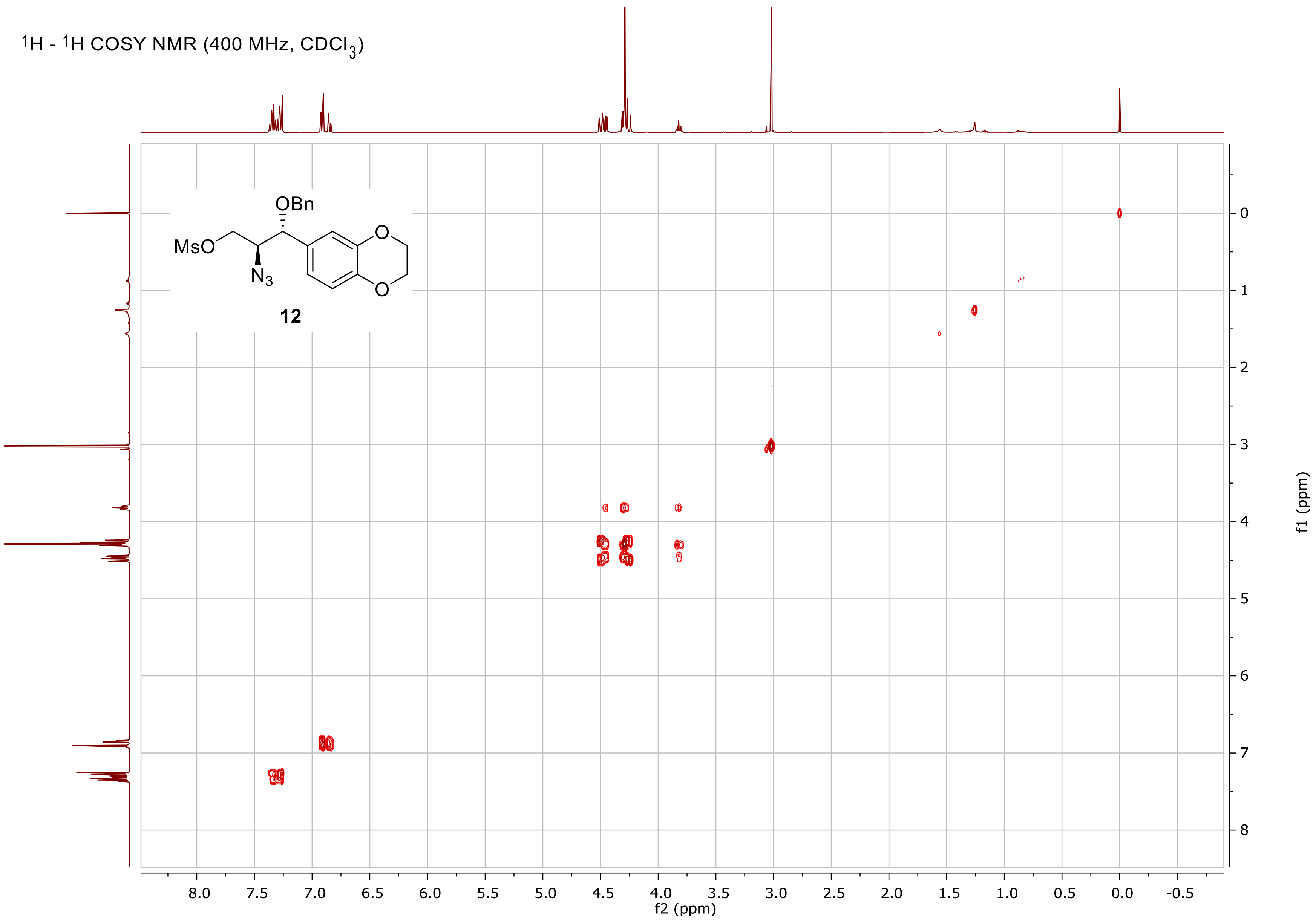

^{1}H - ^{13}C HSQC NMR (400 MHz, $CDCl_3$)

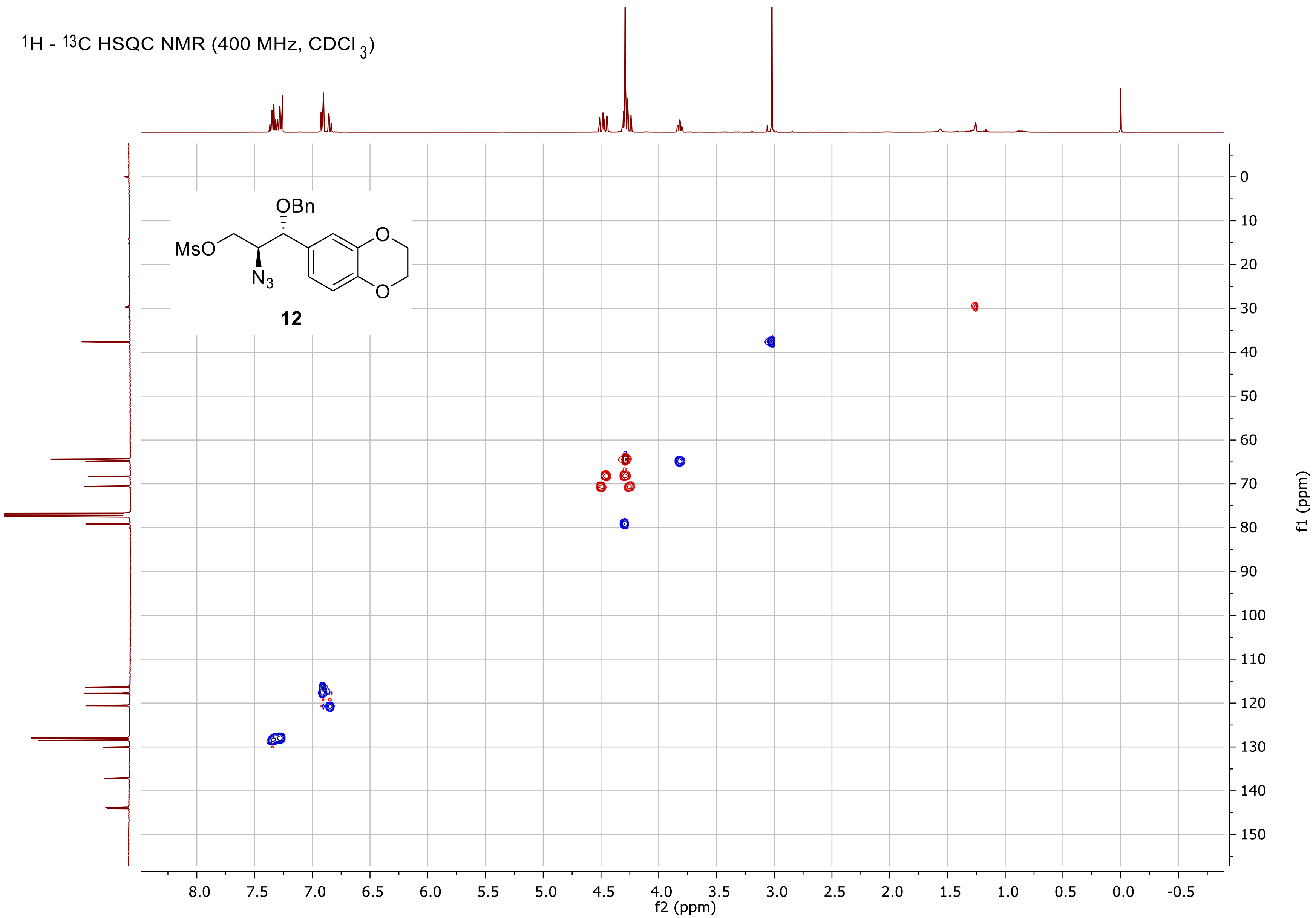

^{1}H NMR (400 MHz, $CDCl_3$)

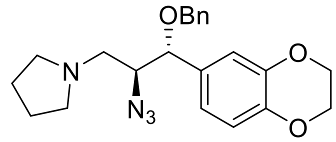

6

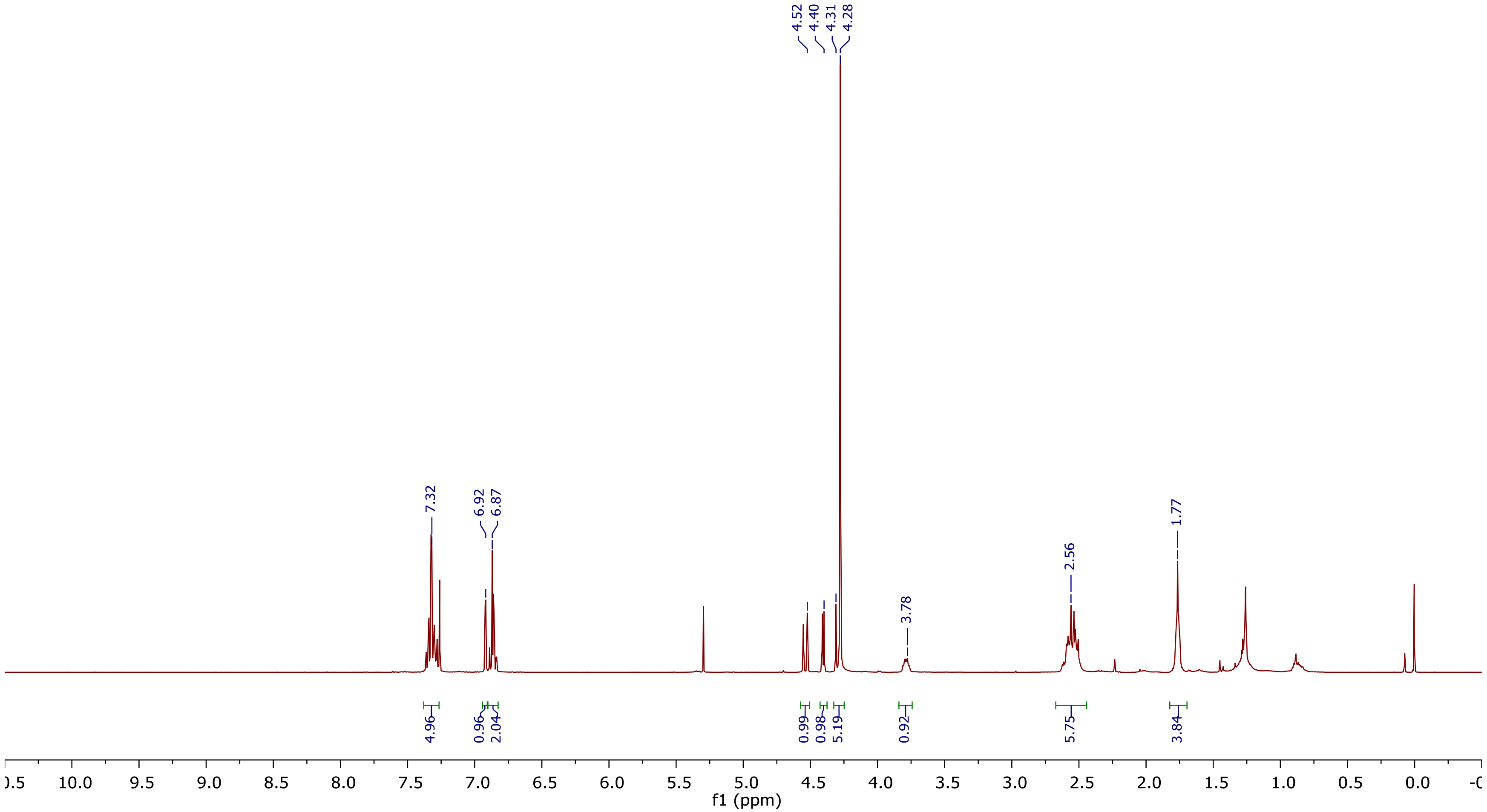

$^{13}C\{^1H\}$ NMR (100.6 MHz, $CDCl_3$)

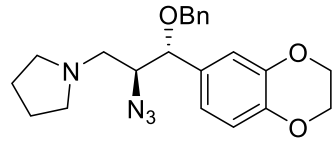

6

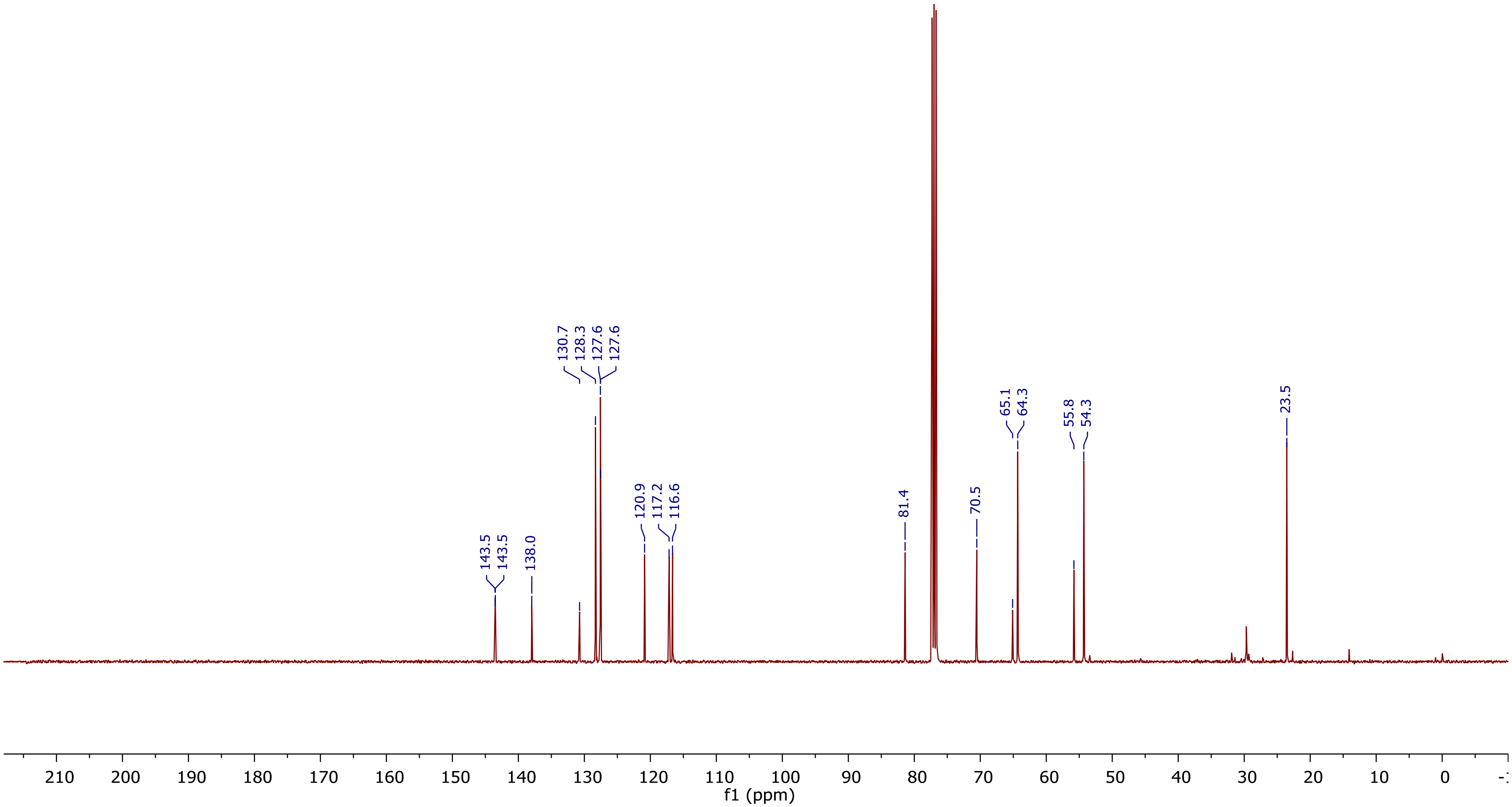

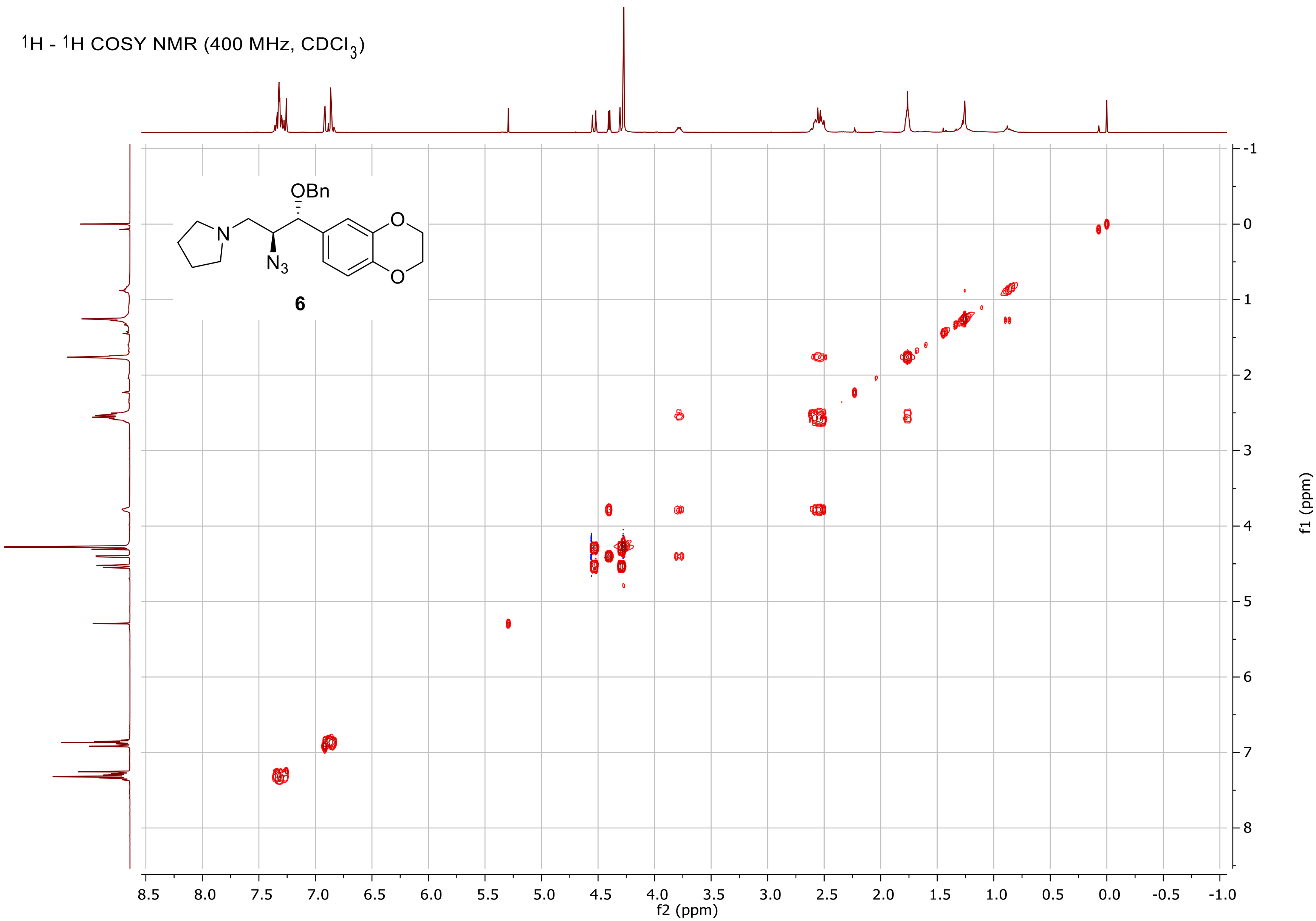

1H - 1H COSY NMR (400 MHz, CDCl3)
OBn
N
N3
O
O
6
f2 (ppm)
f1 (ppm)

^{1}H - ^{13}C HSQC NMR (400 MHz, $CDCl_3$)

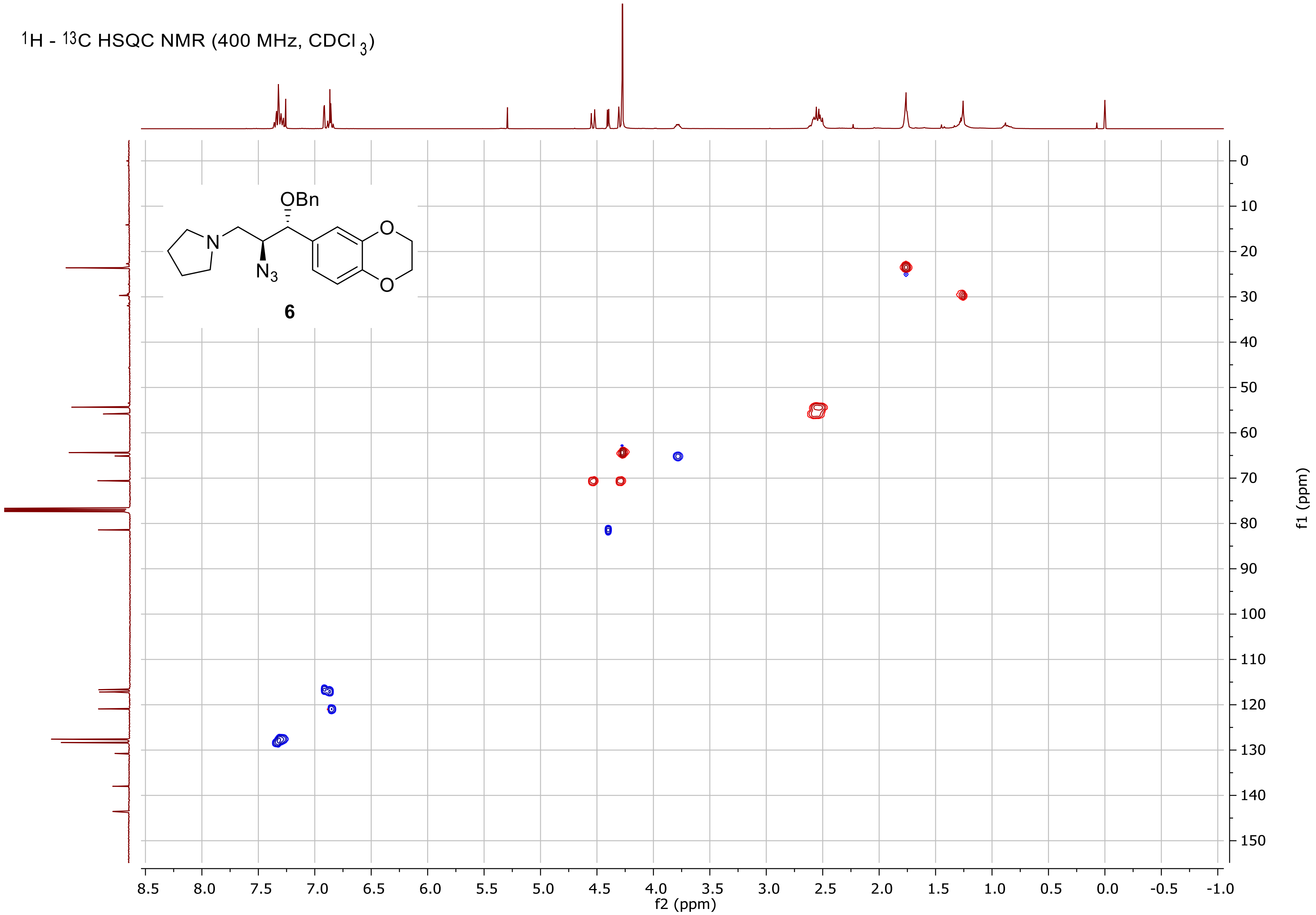

^{1}H NMR (400 MHz, $CDCl_3$)

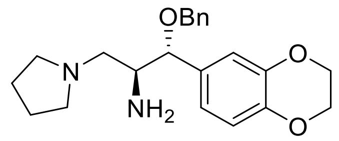

A

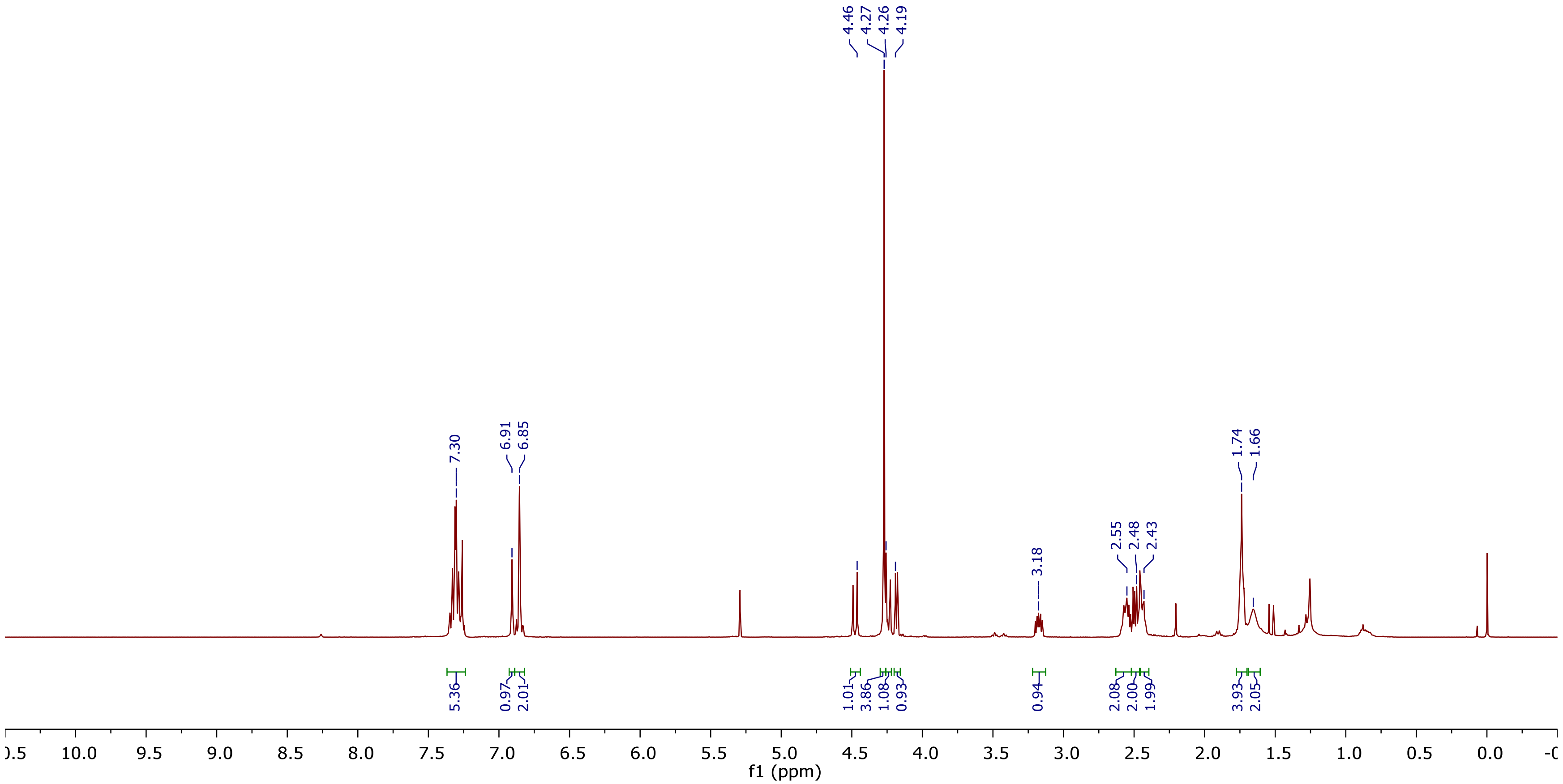

$^{13}C\{^1H\}$ NMR (100.6 MHz, $CDCl_3$)

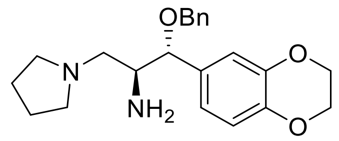

A

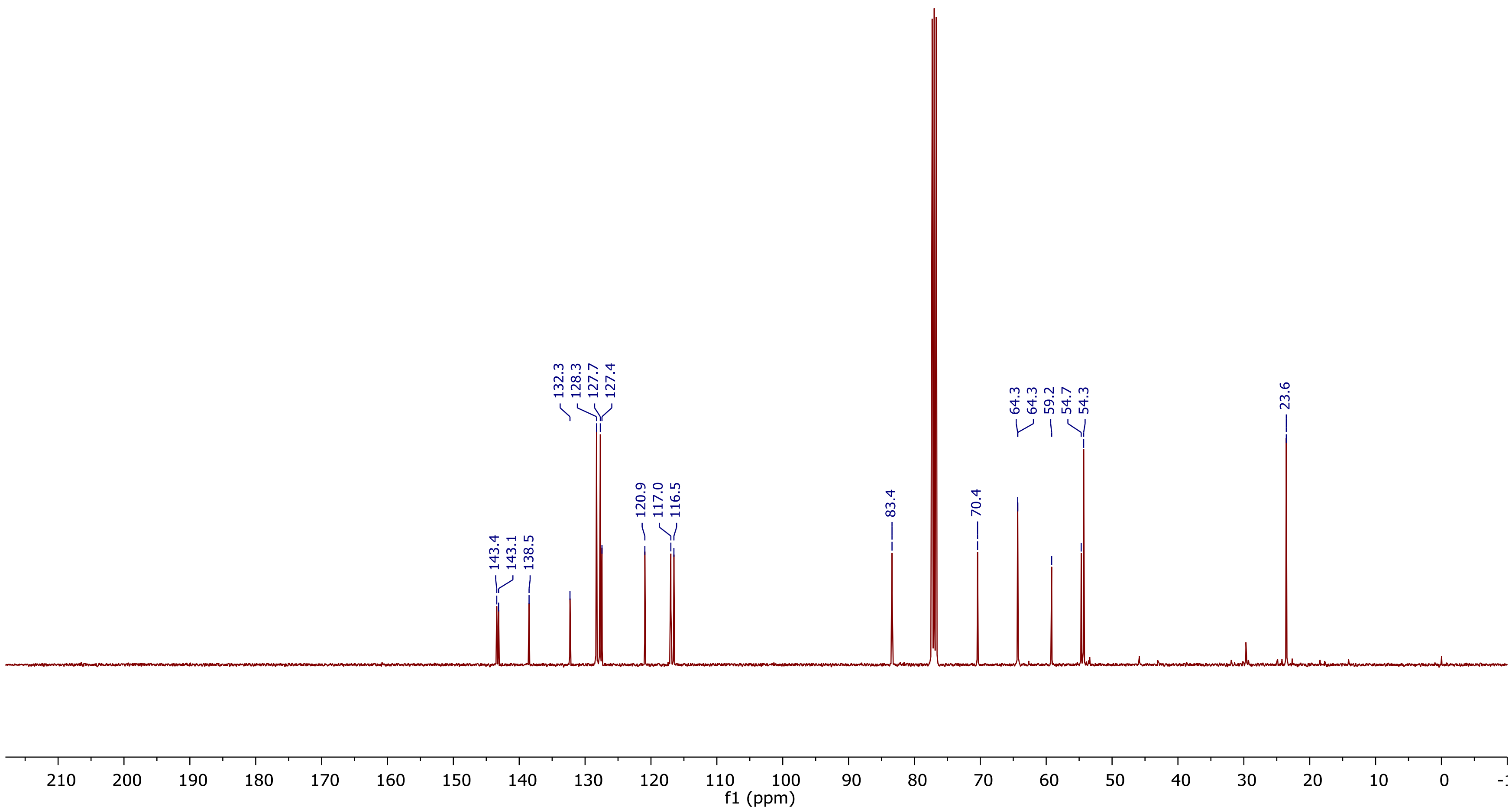

^{1}H - ^{1}H COSY NMR (400 MHz, $CDCl_3$)

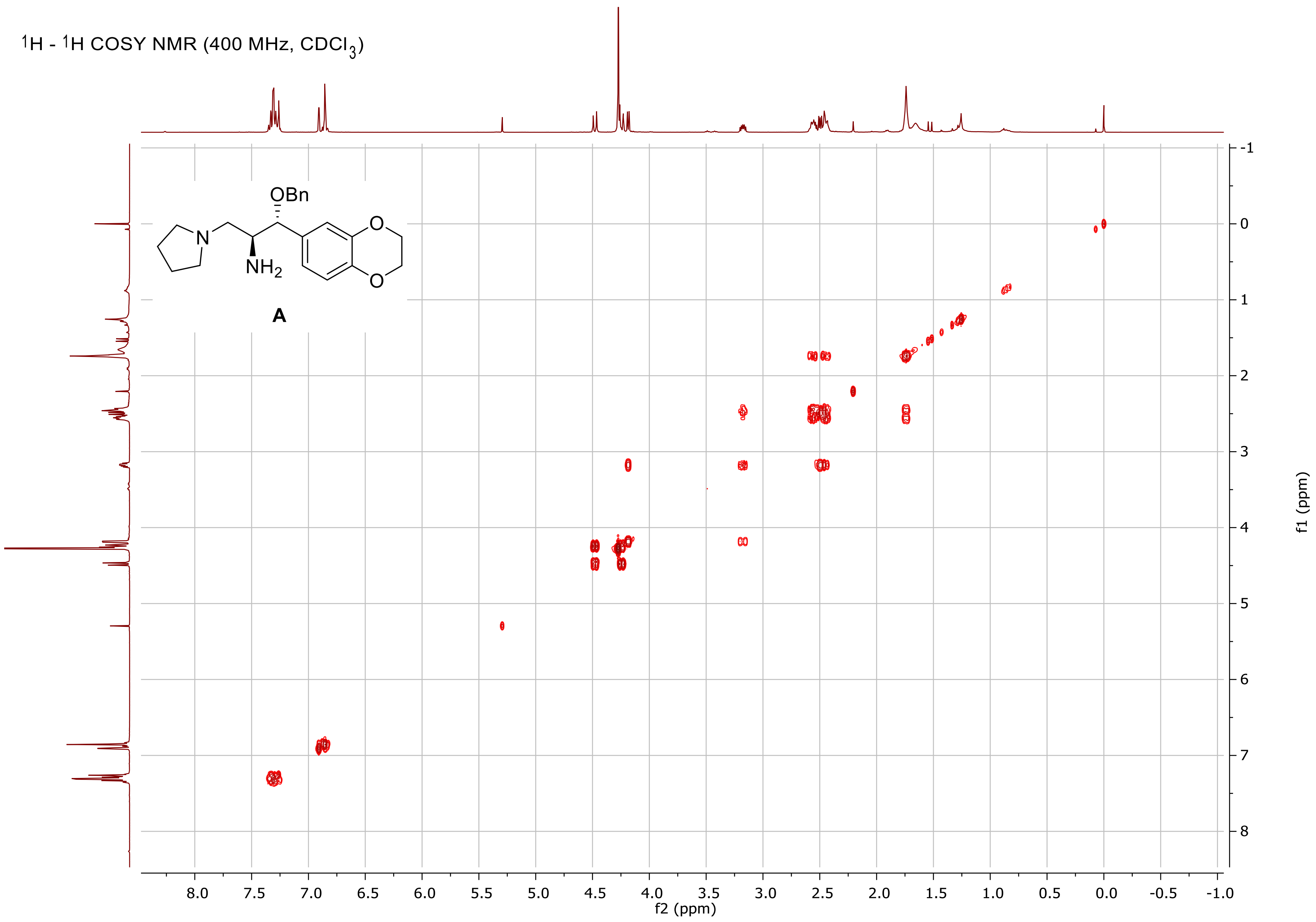

^{1}H - ^{13}C HSQC NMR (400 MHz, $CDCl_3$)

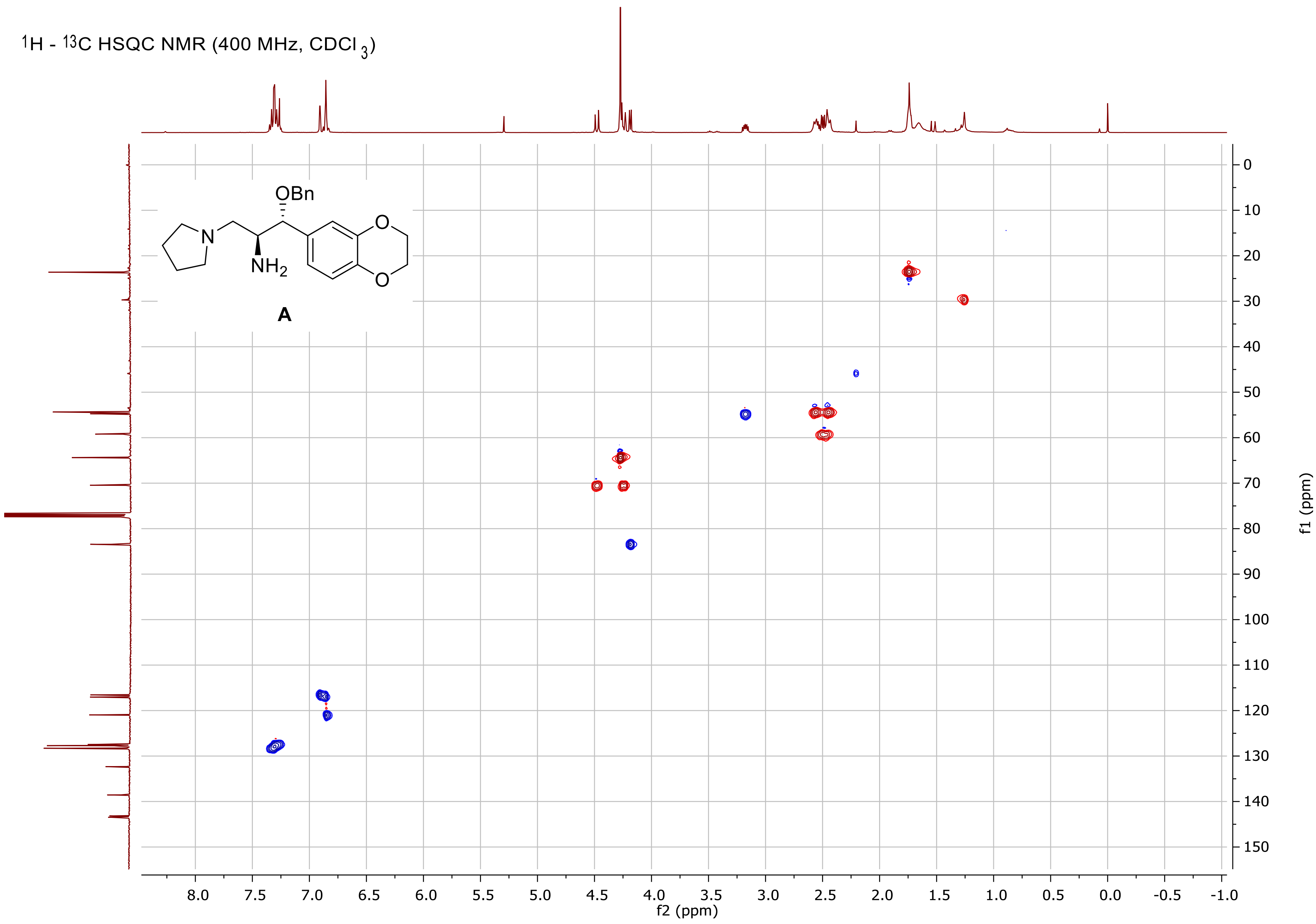

^{1}H NMR (400 MHz, $CDCl_3$)

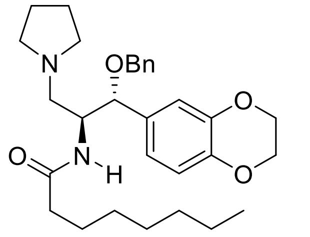

13

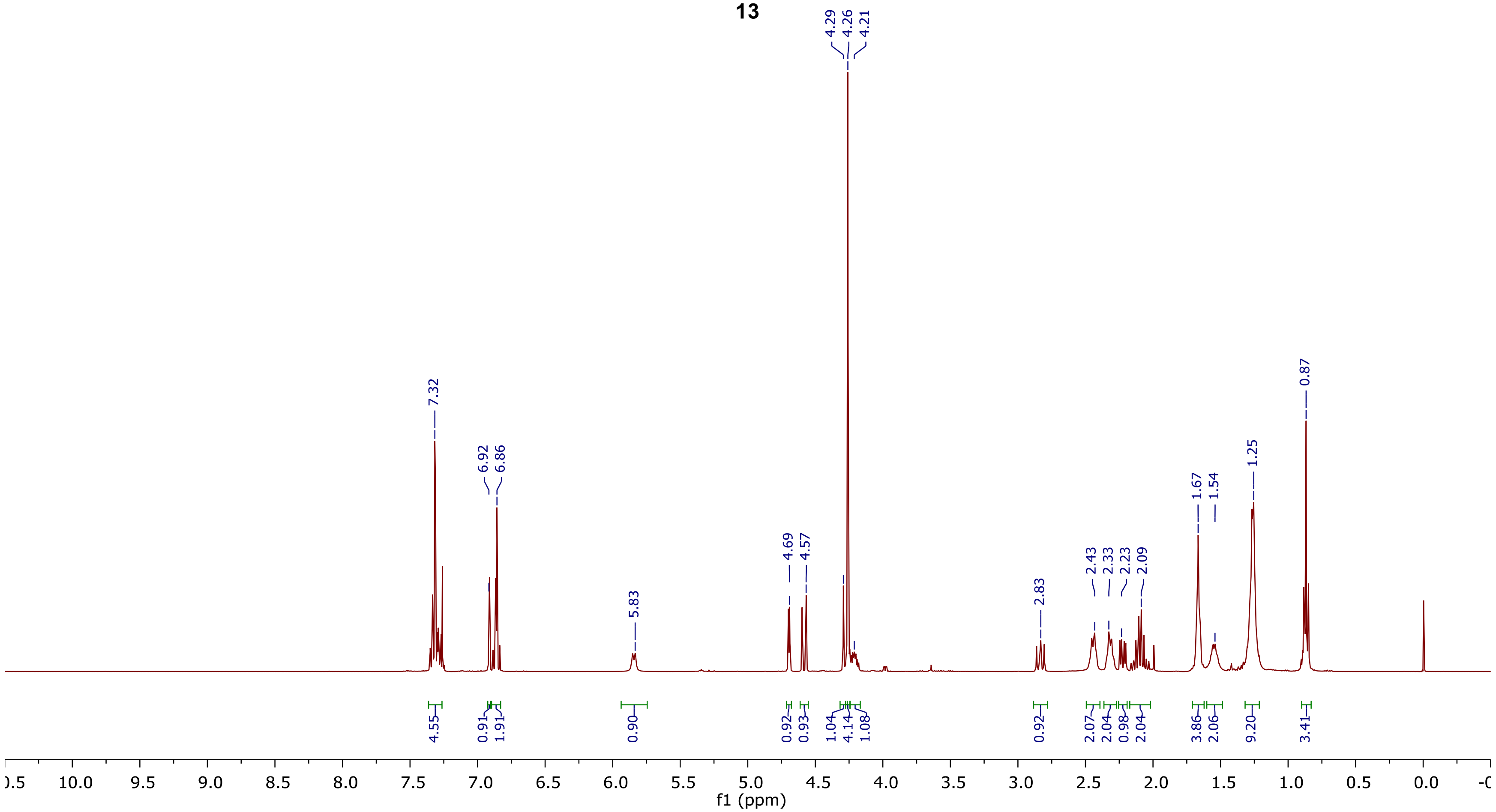

$^{13}C\{^1H\}$ NMR (100.6 MHz, $CDCl_3$)

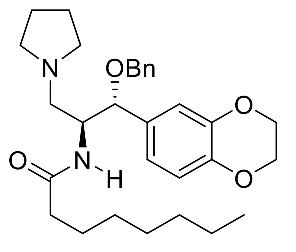

13

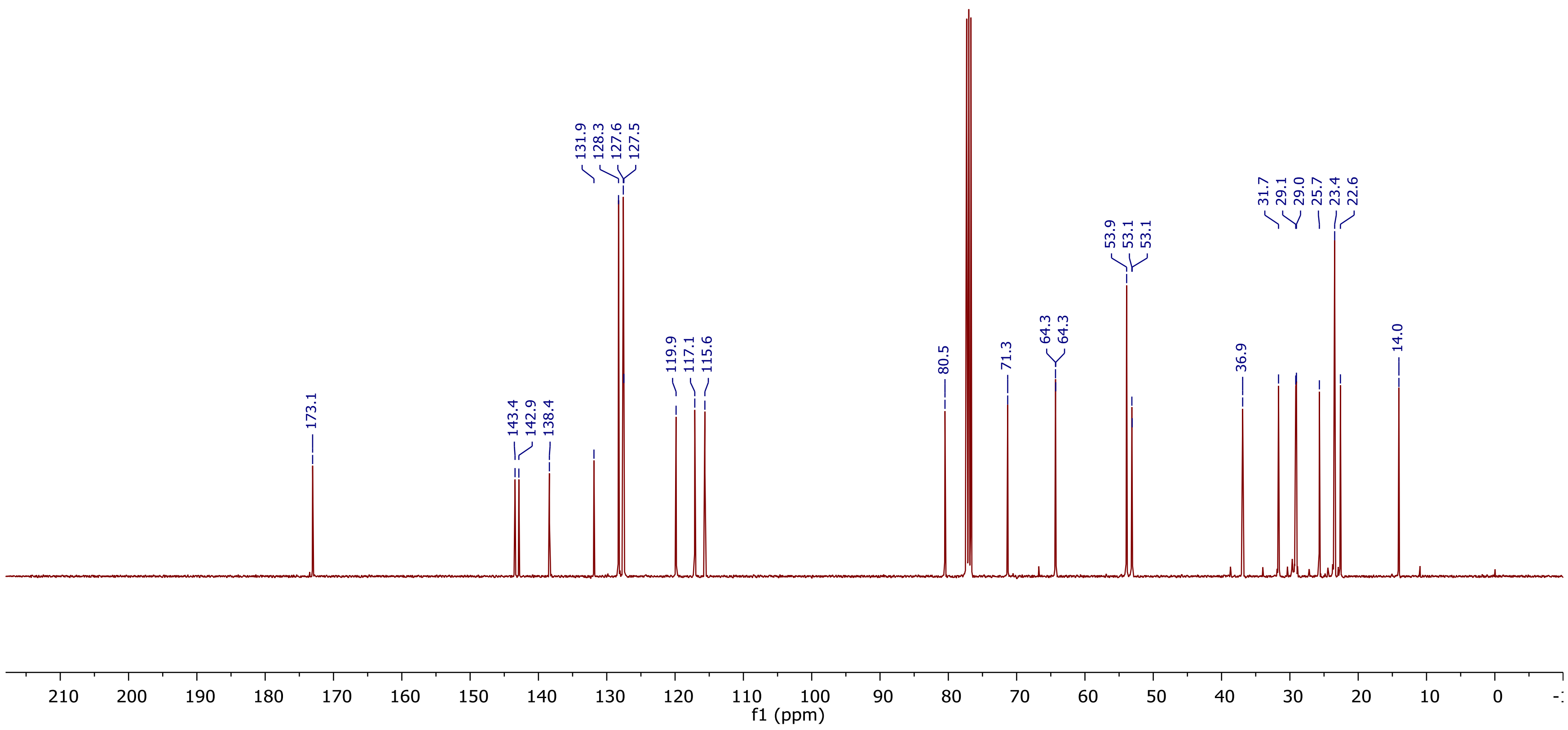

^{1}H - ^{1}H COSY NMR (400 MHz, $CDCl_3$)

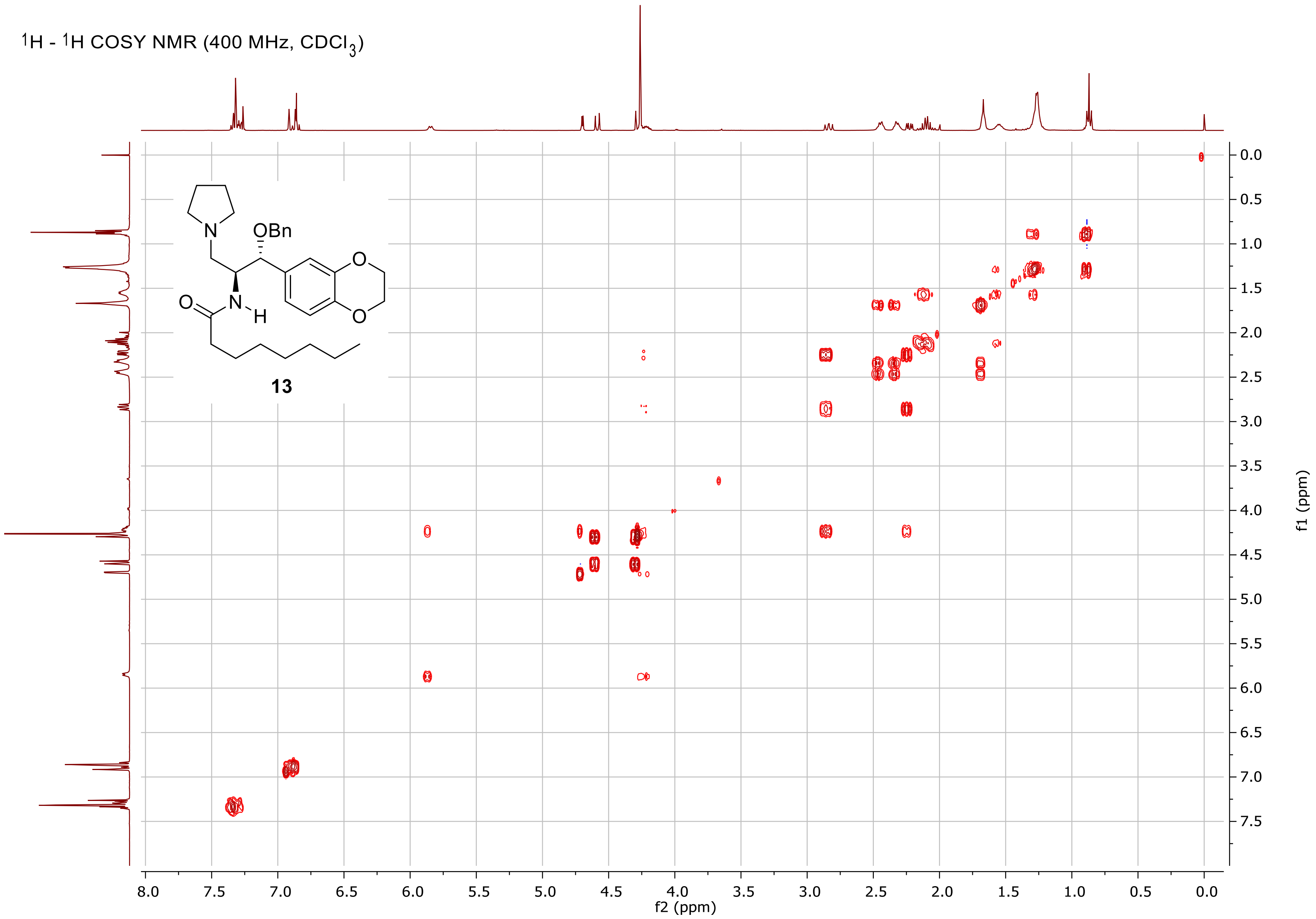

^{1}H - ^{13}C HSQC NMR (400 MHz, $CDCl_3$)

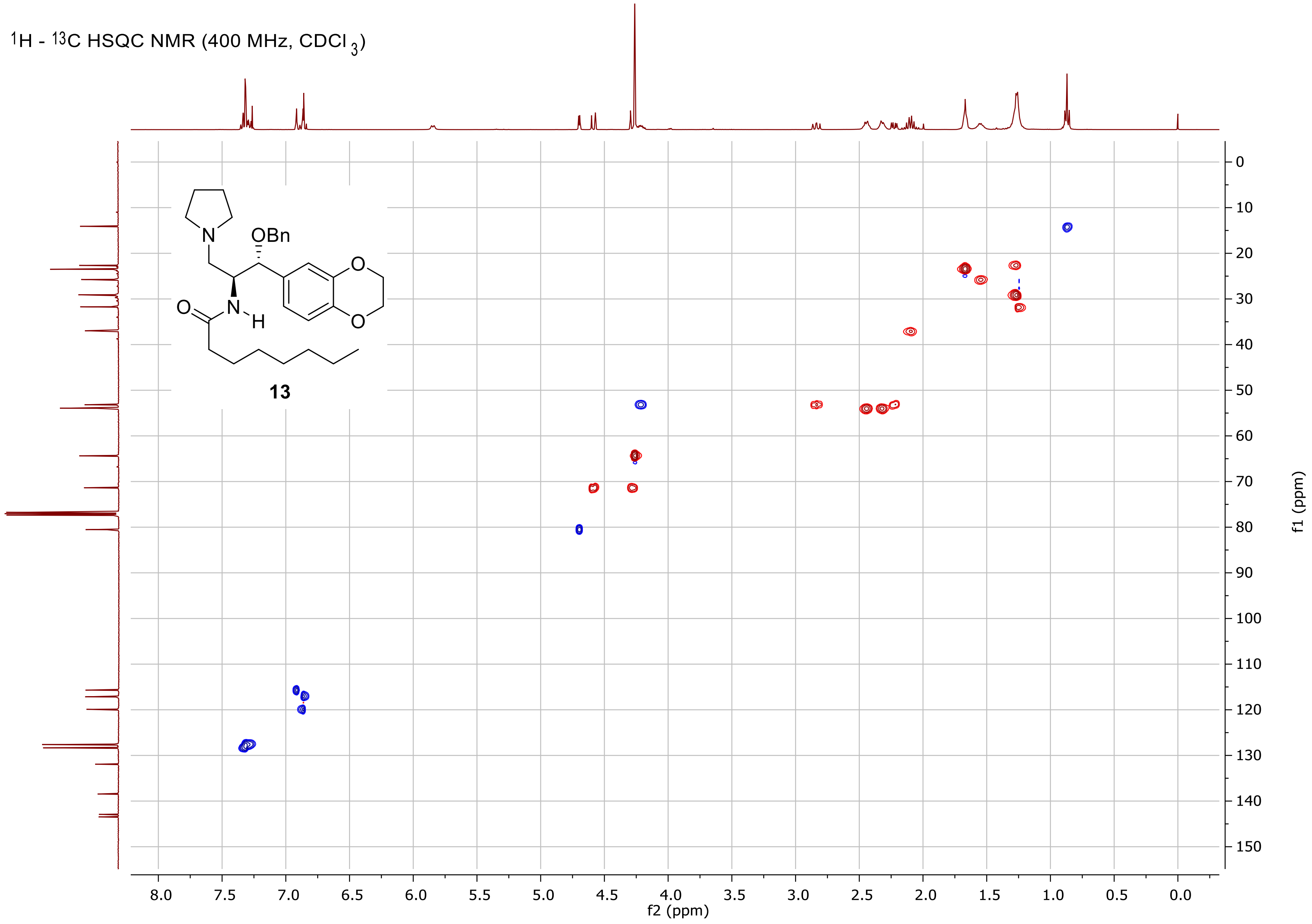

^1H NMR (400 MHz, $CDCl_3$)

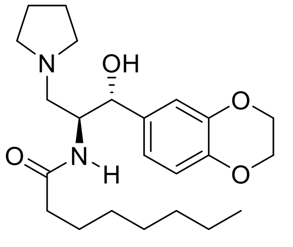

2

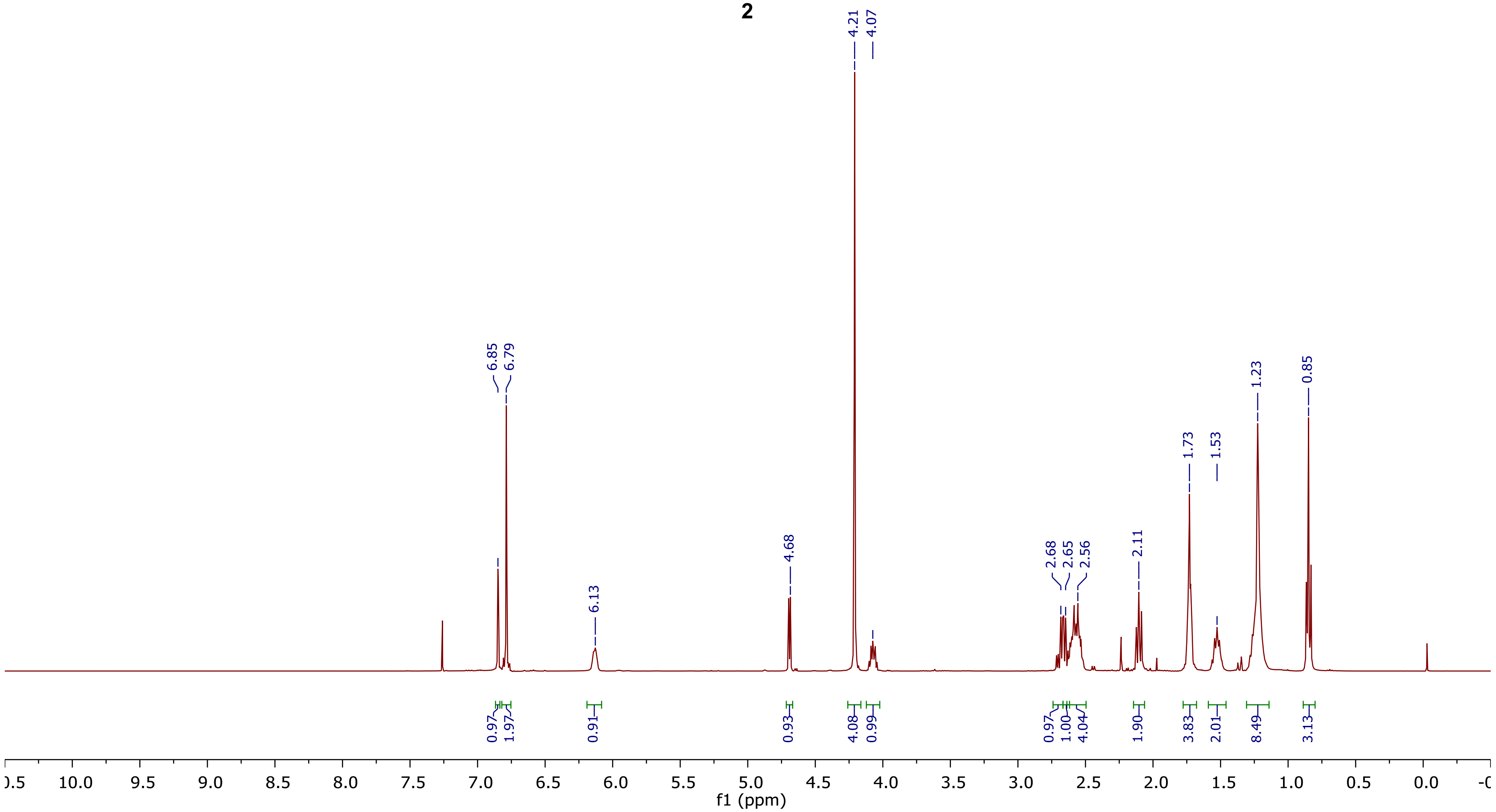

$^{13}C\{^{1}H\}$ NMR (100.6 MHz, $CDCl_3$)

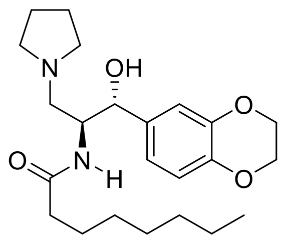

2

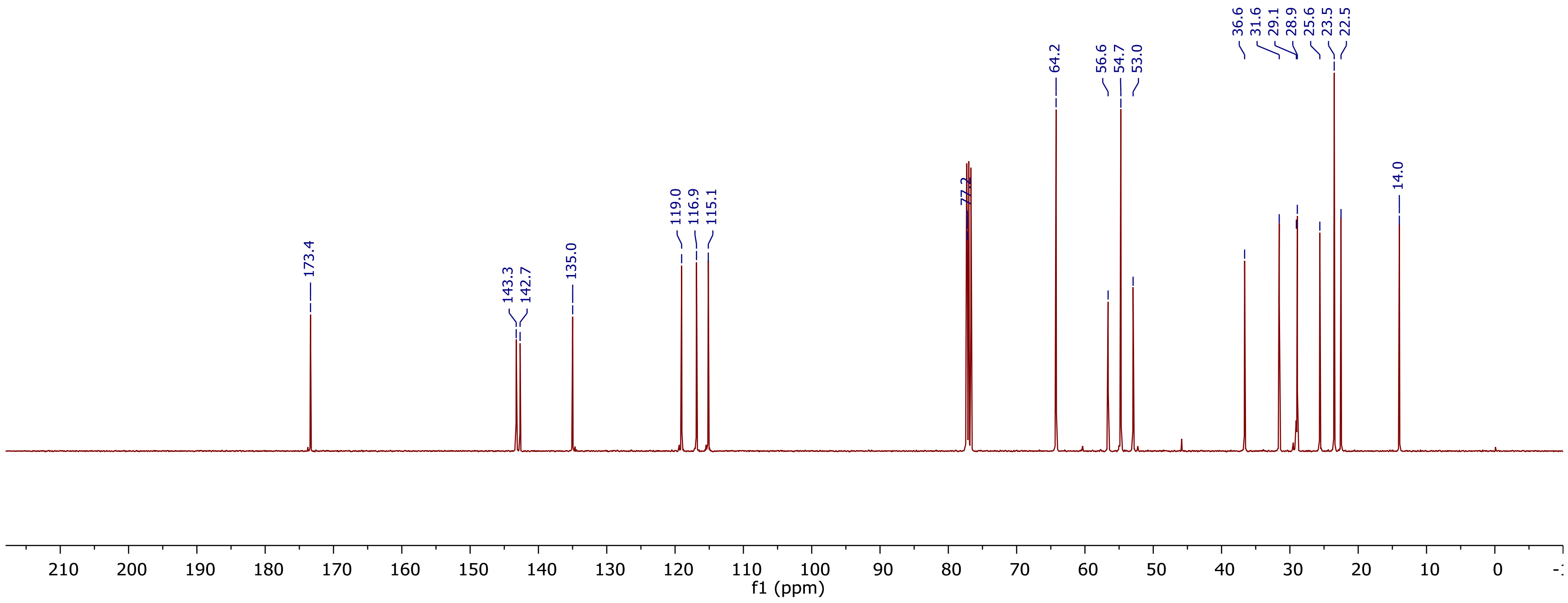

^1H - ^1H COSY NMR (400 MHz, $CDCl_3$)

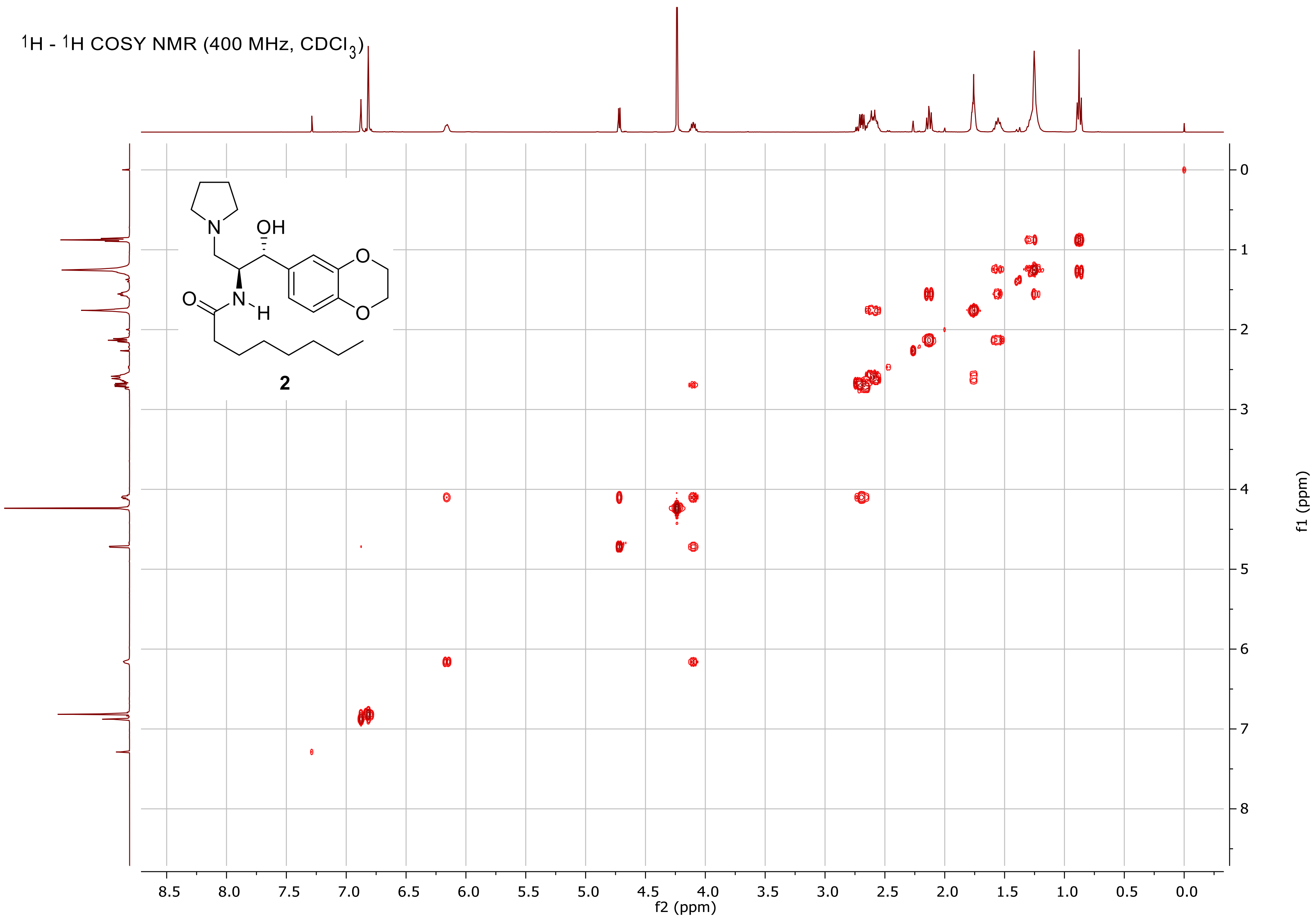

^{1}H - ^{13}C HSQC NMR (400 MHz, $CDCl_3$)

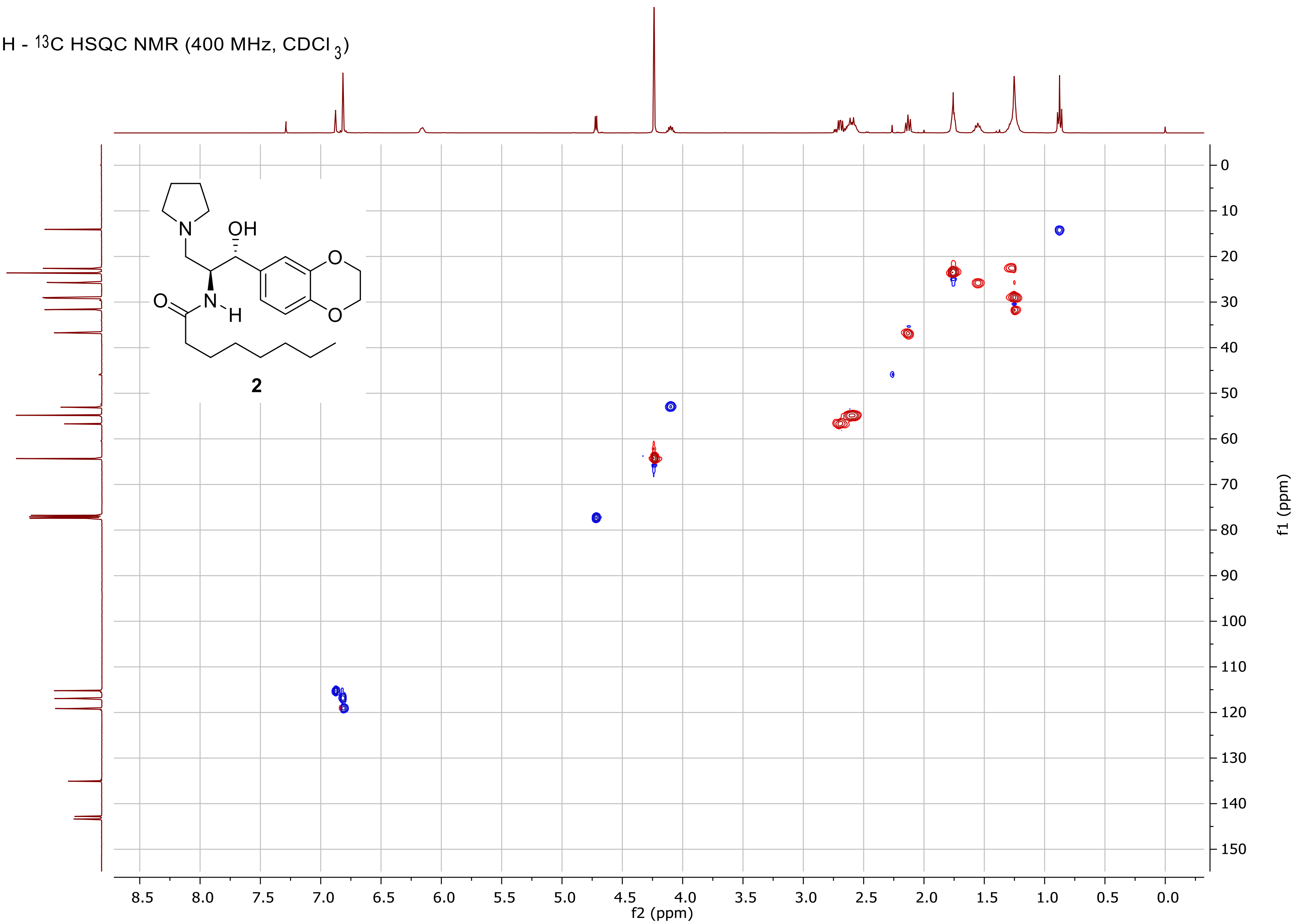

^{1}H NMR (400 MHz, $CDCl_3$)

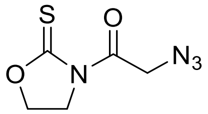

7

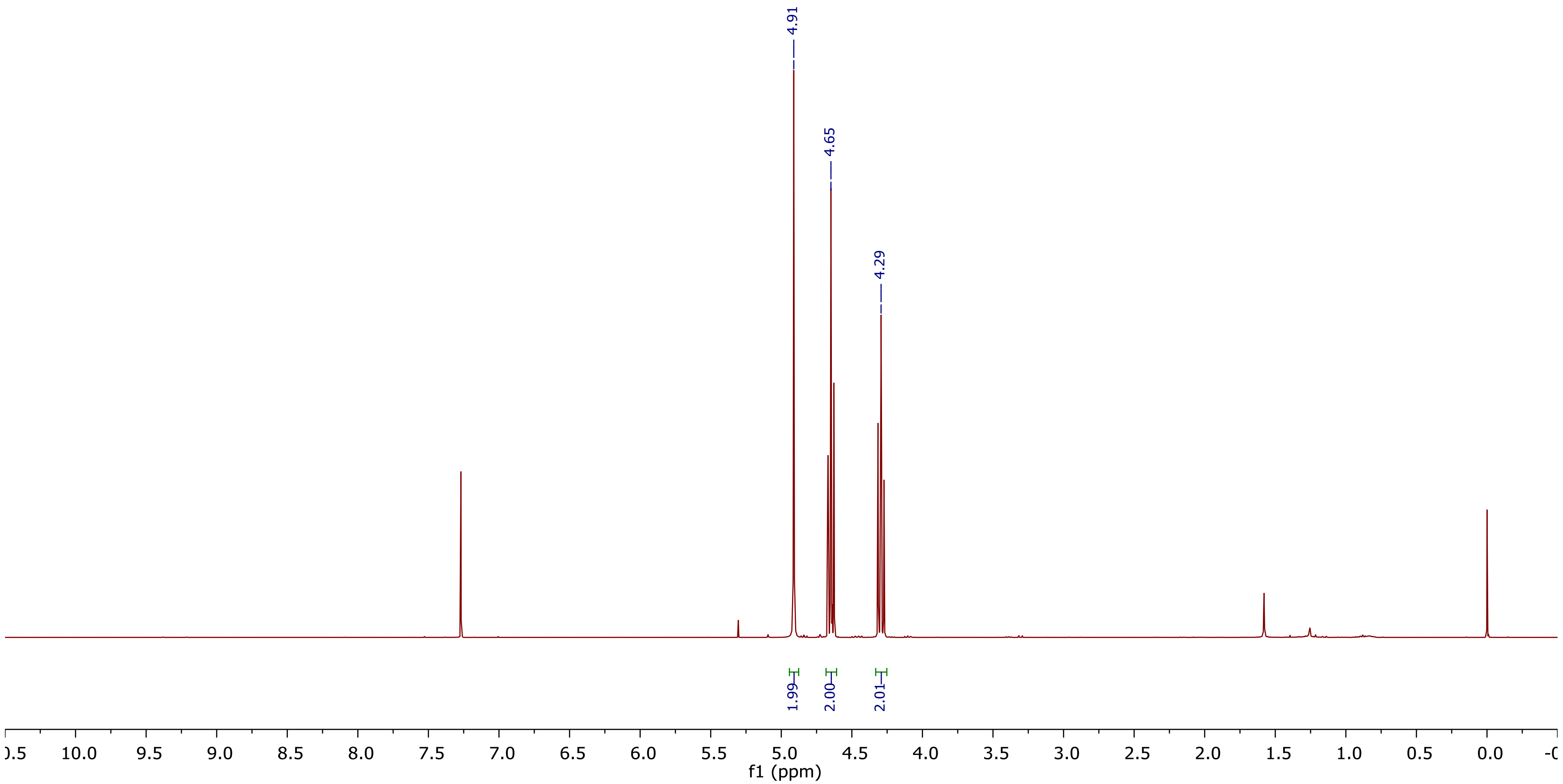

$^{13}C\{^1H\}$ NMR (100.6 MHz, $CDCl_3$)

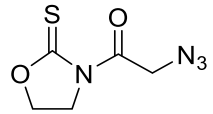

7

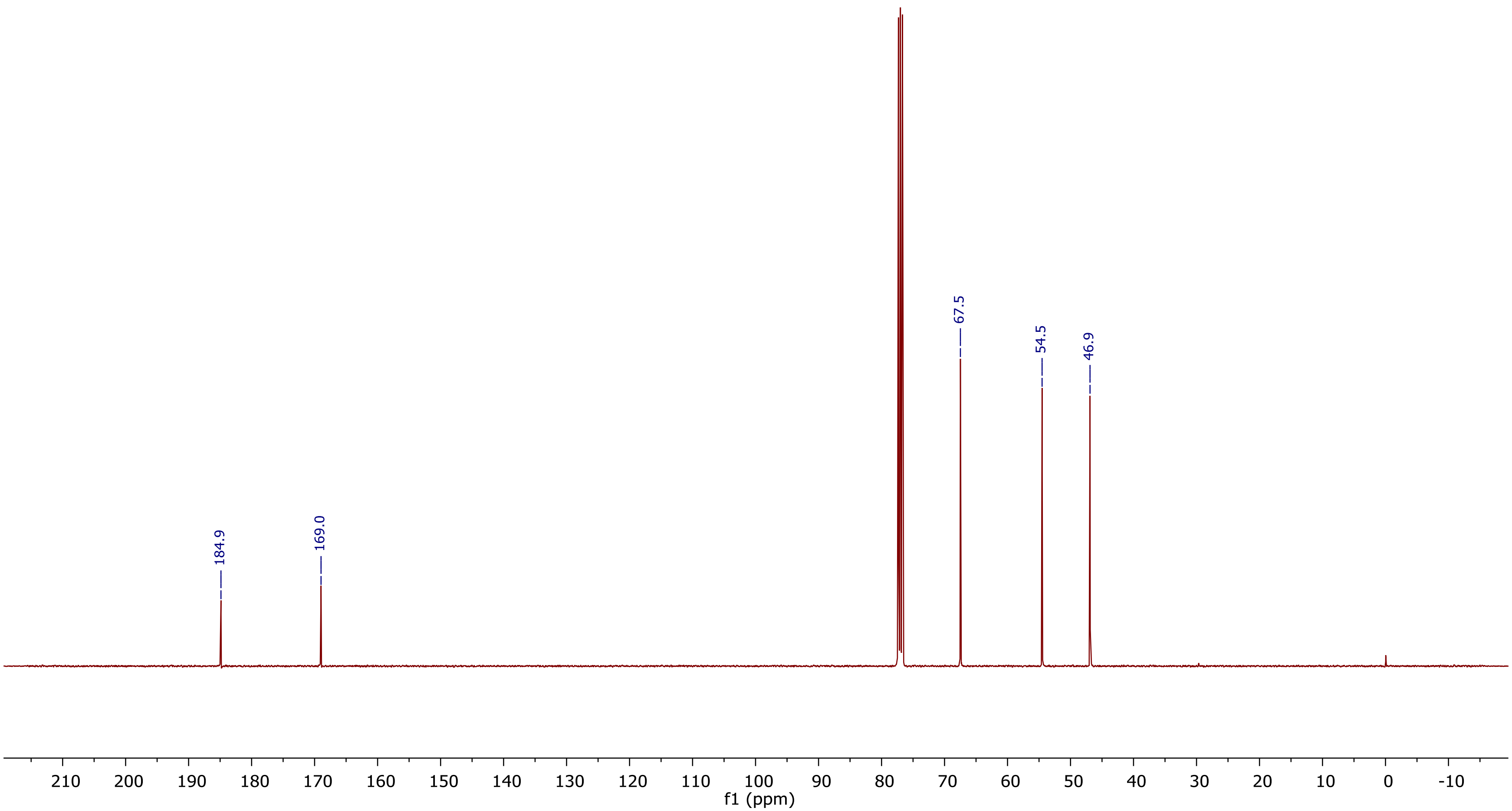

^1H NMR (400 MHz, $CDCl_3$)

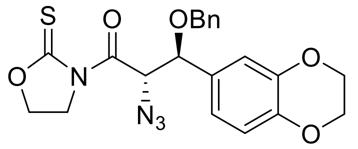

14

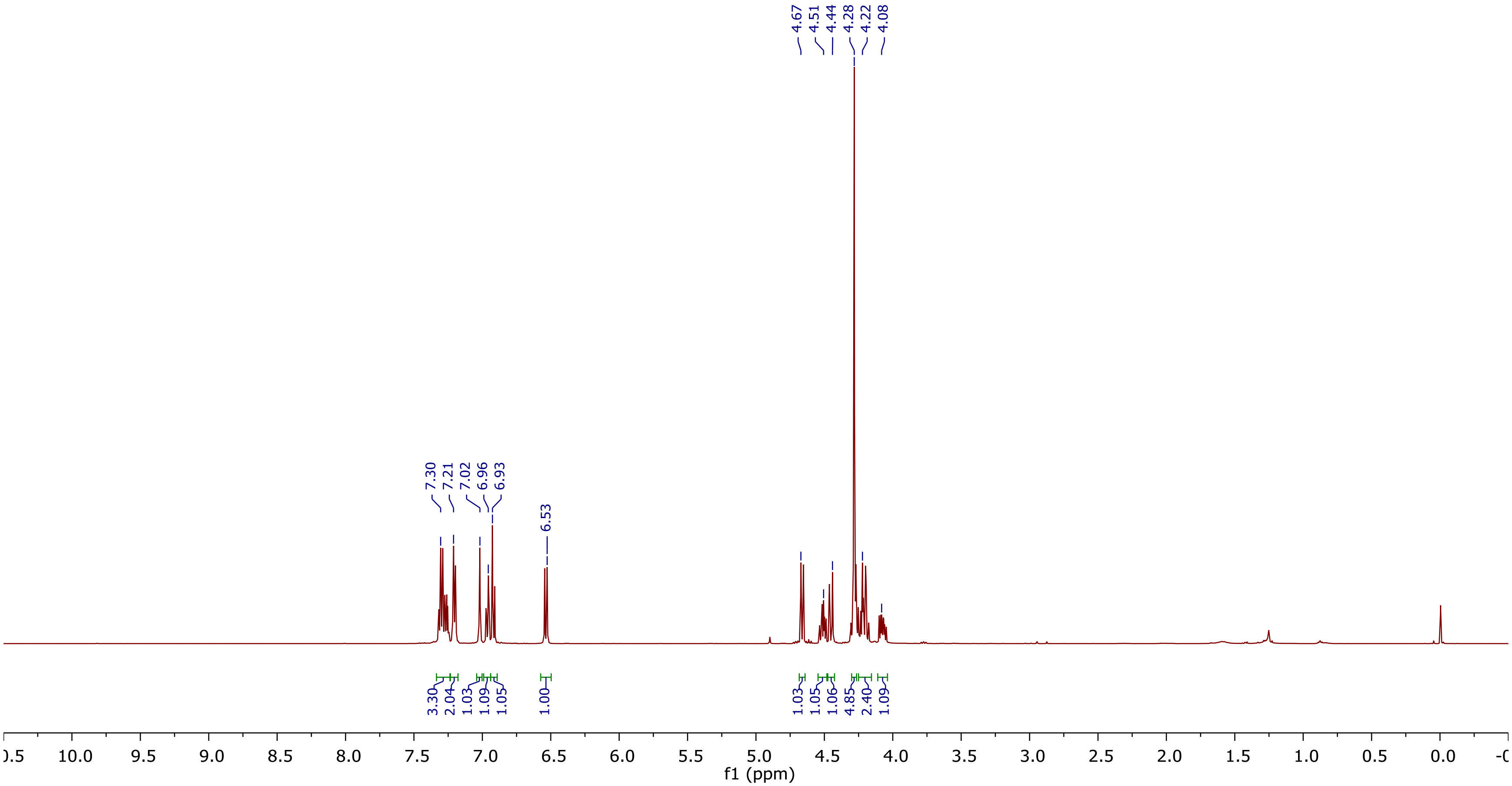

$^{13}C\{^{1}H\}$ NMR (100.6 MHz, $CDCl_3$)

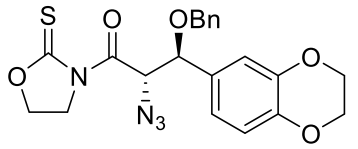

14

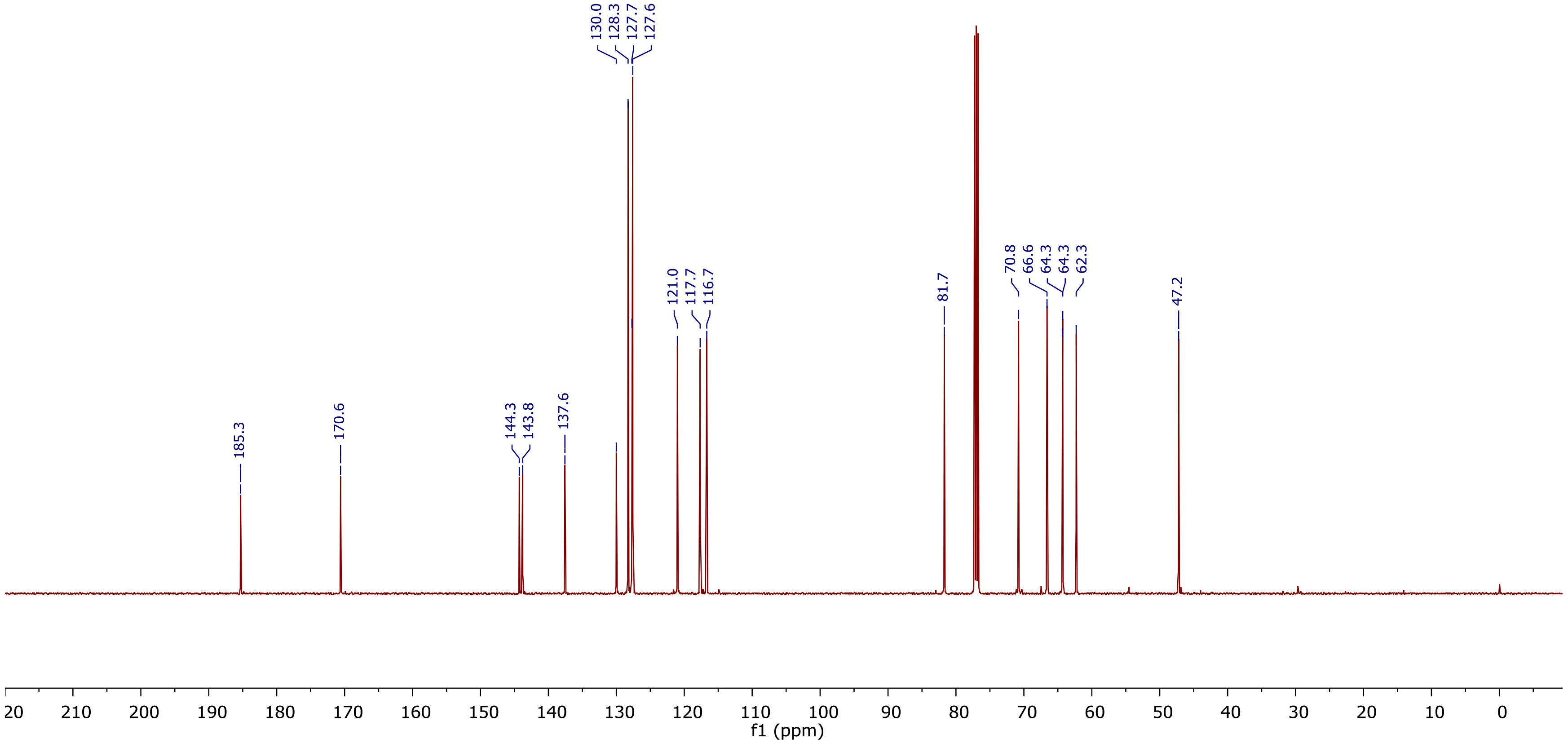

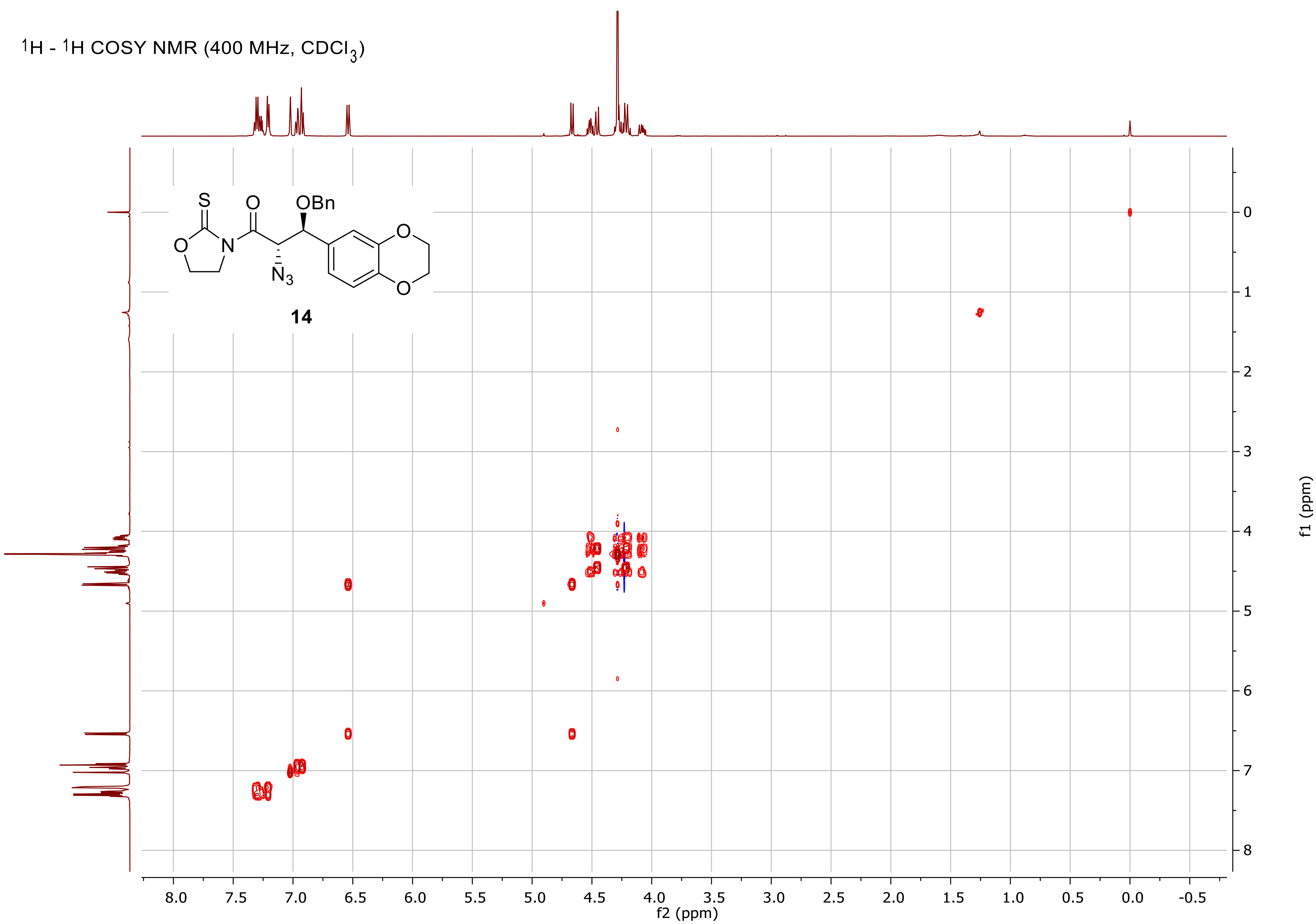

^{1}H - ^{1}H COSY NMR (400 MHz, $CDCl_3$)
S
O
OBn
O
N
N_3
O
O
14
f2 (ppm)
f1 (ppm)

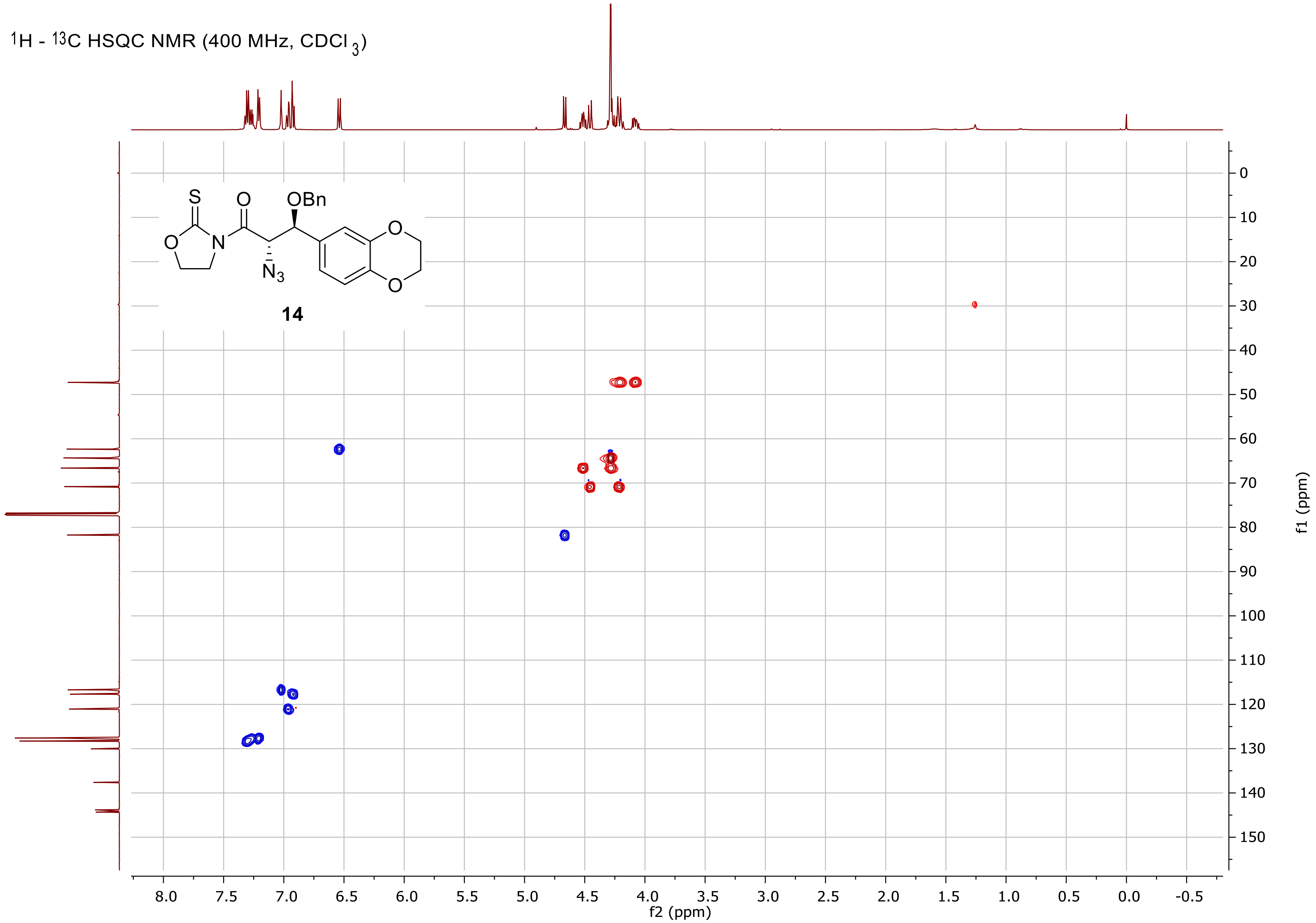
^1H - ^{13}C HSQC NMR (400 MHz, $CDCl_3$)
14
f2 (ppm)
f1 (ppm)

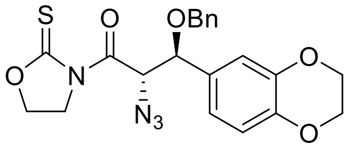

14

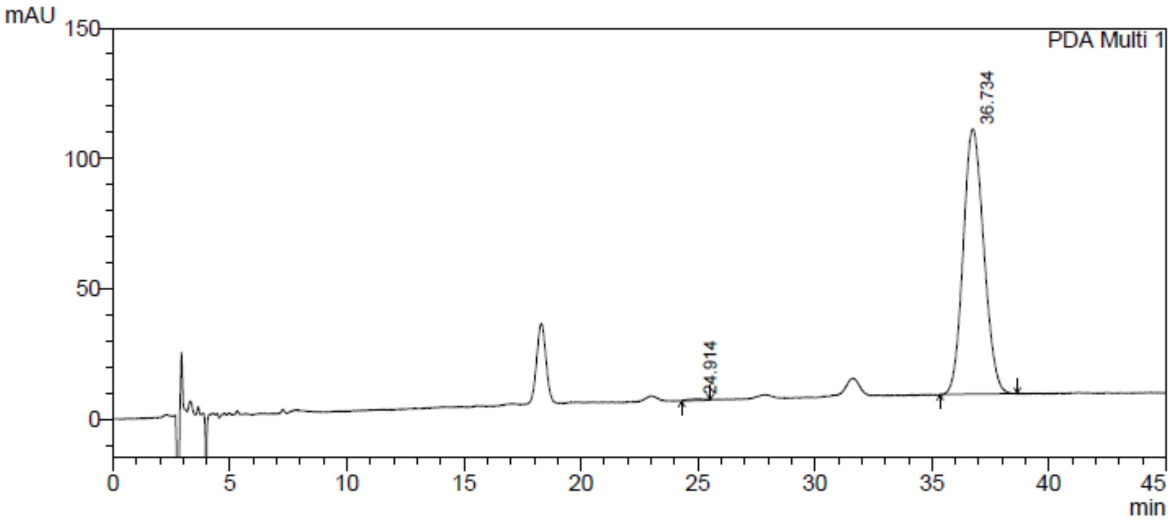

1 PDA Multi 1/254nm 4nm

PeakTable

PDA Ch1 254nm 4nm

Peak#	Ret. Time	Area	Height	Area %	Height %
1	24.914	26821	719	0.428	0.702
2	36.734	6233969	101772	99.572	99.298
Total		6260790	102491	100.000	100.000

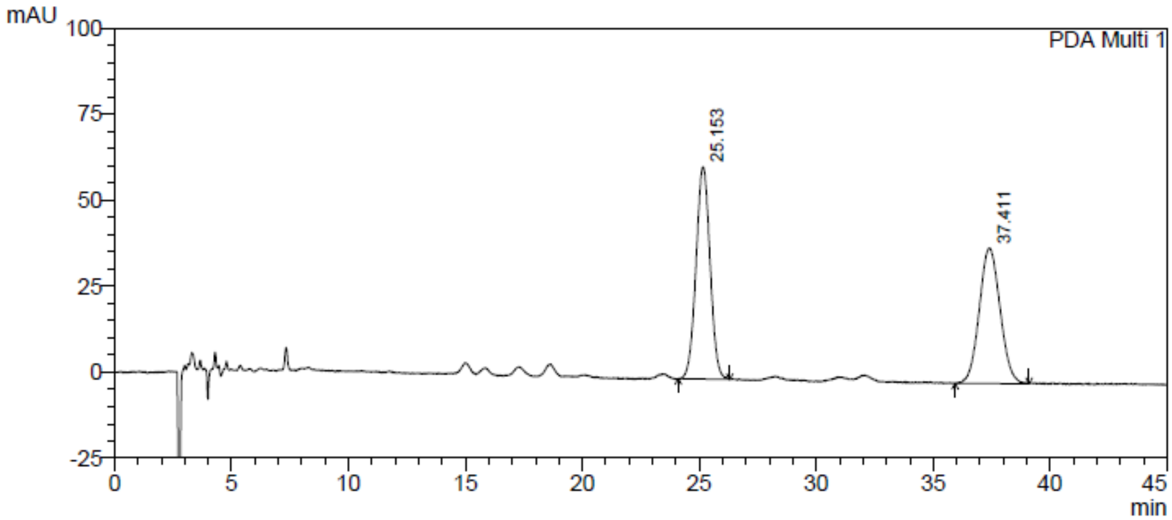

1 PDA Multi 1/254nm 4nm

PeakTable

PDA Ch1 254nm 4nm

Peak#	Ret. Time	Area	Height	Area %	Height %
1	25.153	2574417	61699	51.389	61.060
2	37.411	2435273	39347	48.611	38.940
Total		5009690	101046	100.000	100.000

^{1}H NMR (400 MHz, $CDCl_3$)

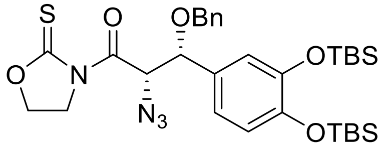

16

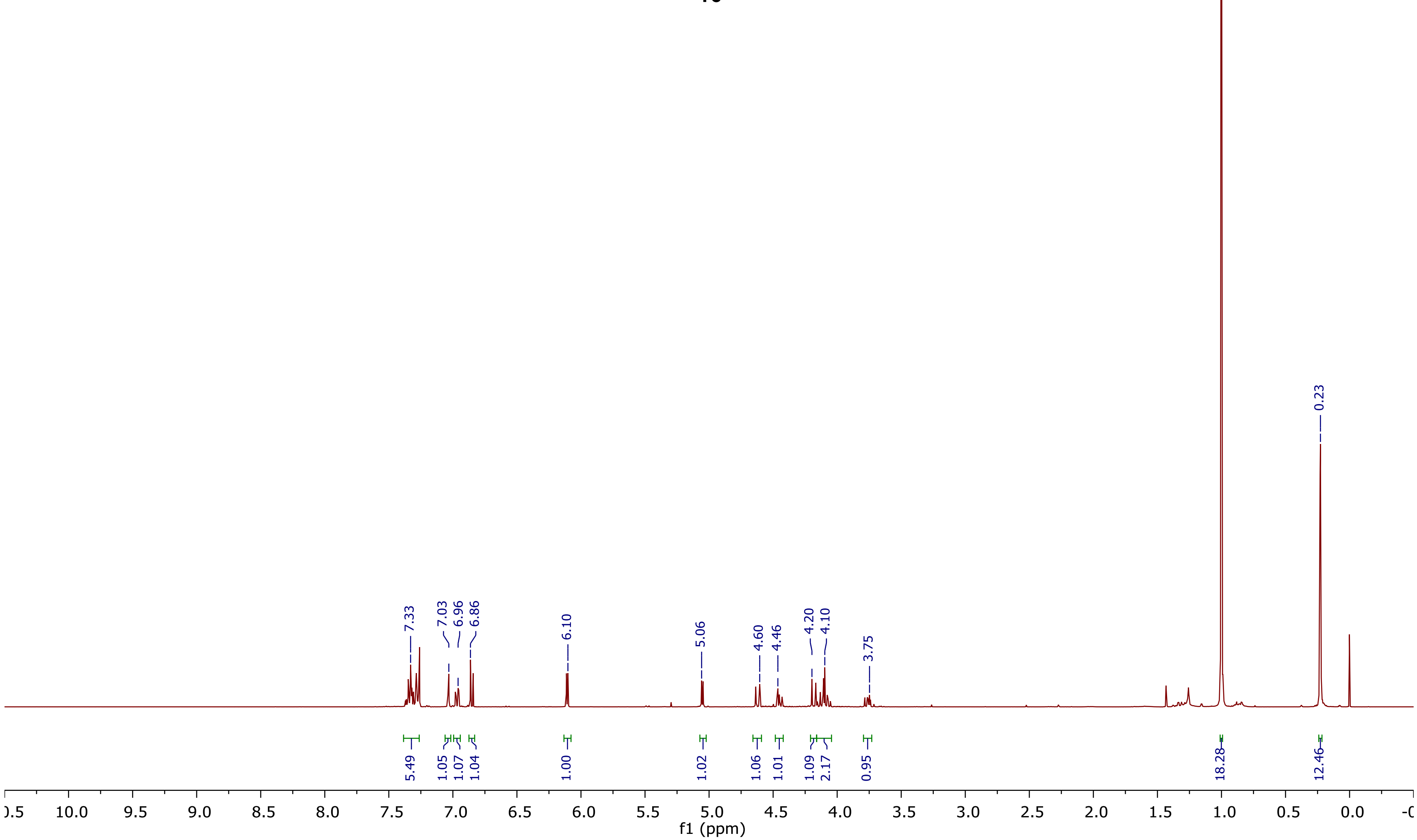

$^{13}C\{^{1}H\}$ NMR (100.6 MHz, $CDCl_3$)

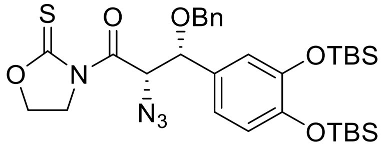

16

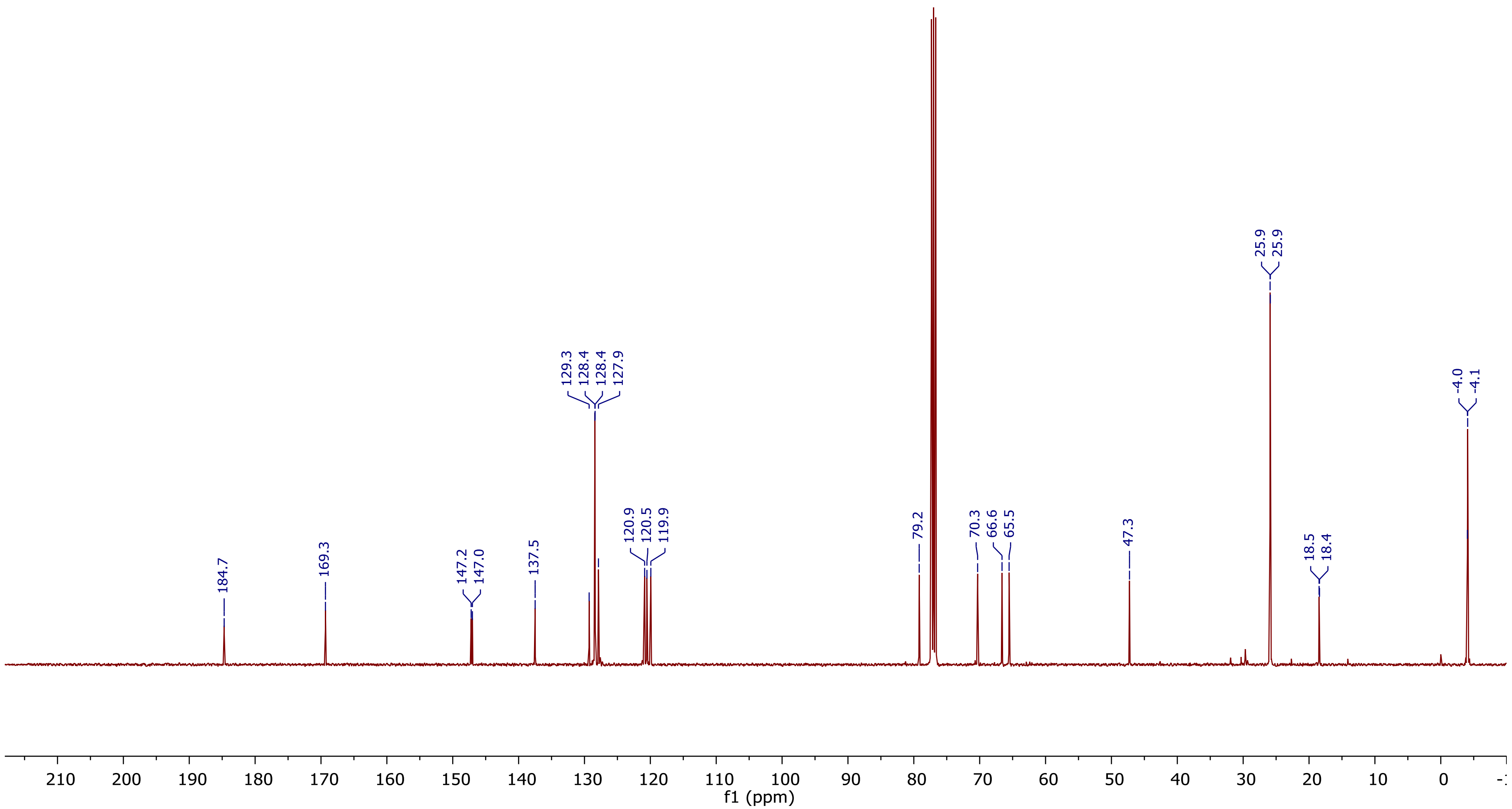

^1H - ^1H COSY NMR (400 MHz, $CDCl_3$)

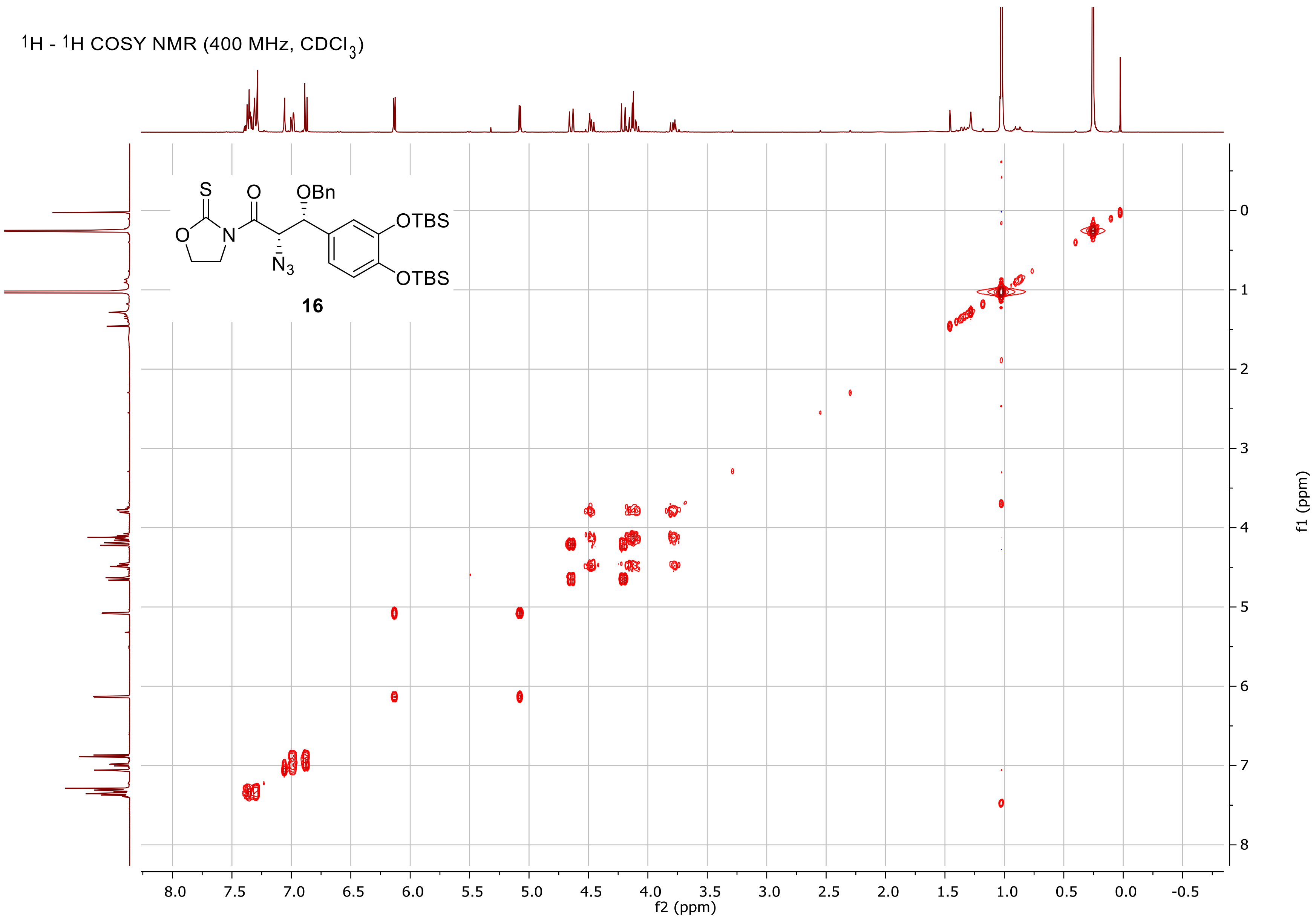

^1H - ^{13}C HSQC NMR (400 MHz, $CDCl_3$)

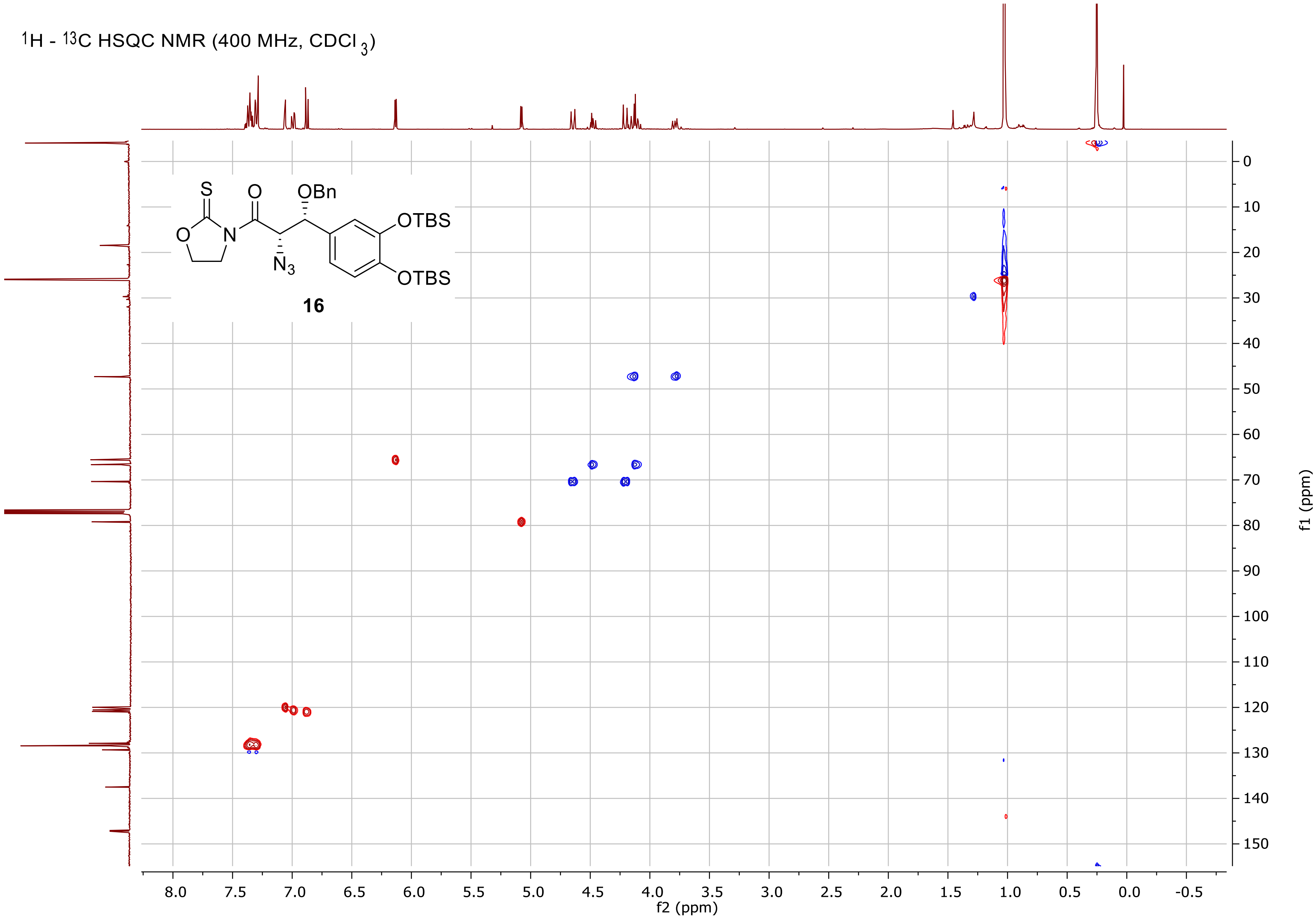

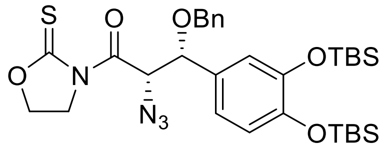

16

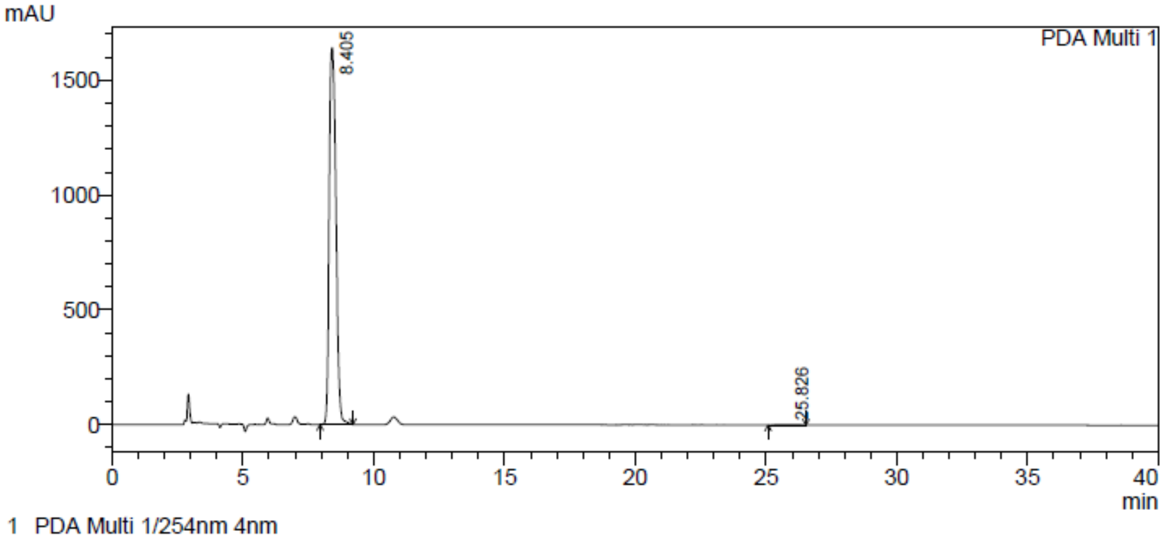

1 PDA Multi 1/254nm 4nm

PeakTable

PDA Ch1 254nm 4nm

Peak#	Ret. Time	Area	Height	Area %	Height %
1	8.405	29403364	1640347	99.838	99.930
2	25.826	47727	1154	0.162	0.070
Total		29451091	1641501	100.000	100.000

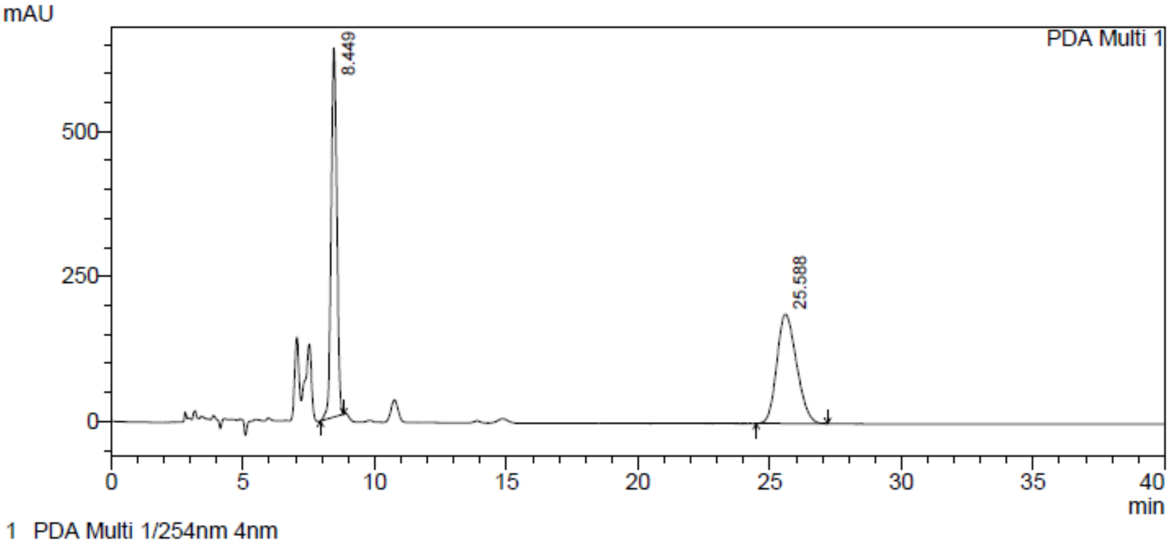

1 PDA Multi 1/254nm 4nm

PeakTable

PDA Ch1 254nm 4nm

Peak#	Ret. Time	Area	Height	Area %	Height %
1	8.449	9517578	636263	49.174	77.160
2	25.588	9837198	188340	50.826	22.840
Total		19354776	824602	100.000	100.000

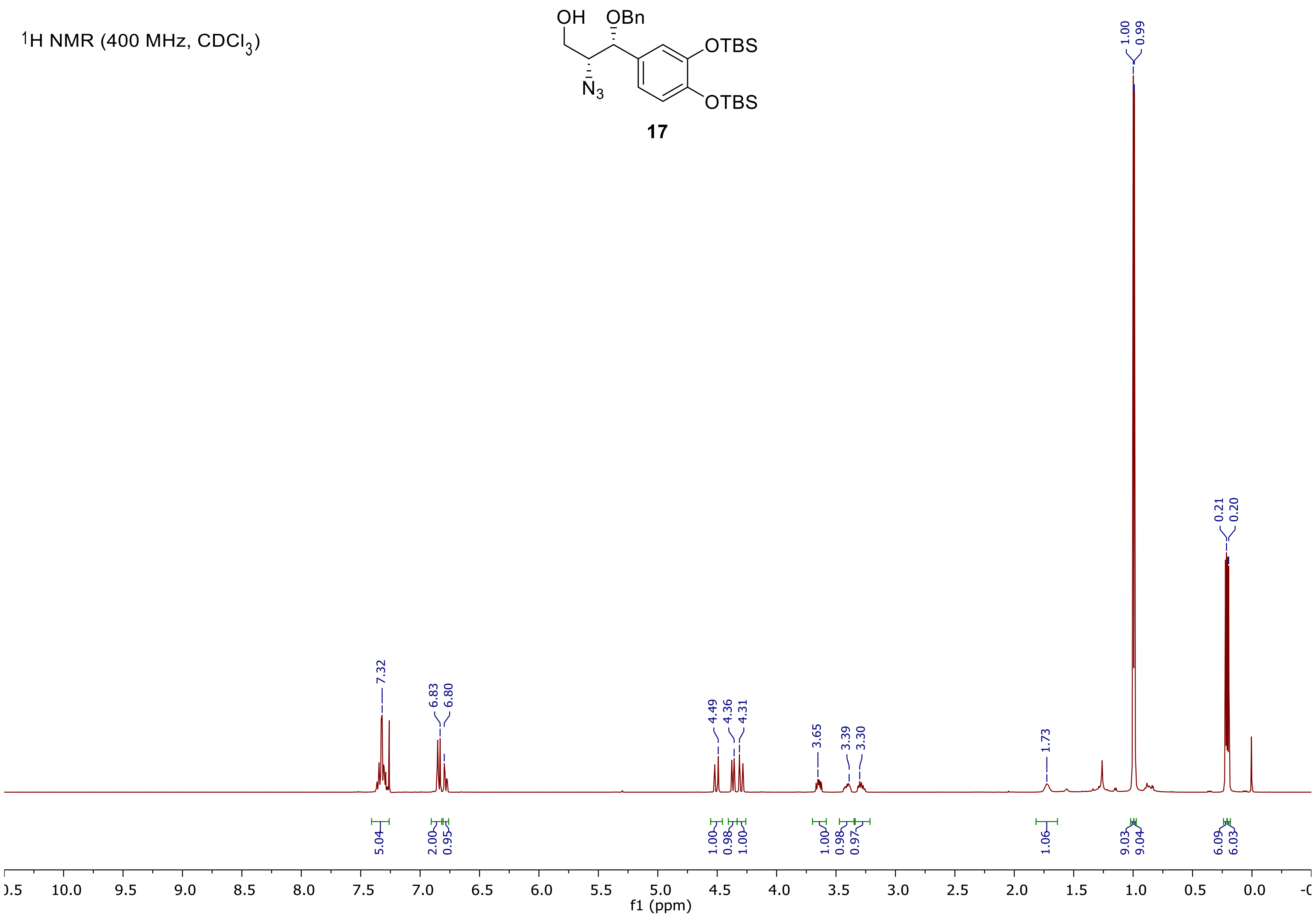

^{1}H NMR (400 MHz, CDCl$_3$)
OH
OBn
OTBS
OTBS
N$_3$
17
1.00
0.99
0.21
0.20
7.32
6.83
6.80
4.49
4.36
4.31
3.65
3.39
3.30
1.73
5.04
2.00
0.95
1.00
0.98
1.00
1.00
0.98
0.97
1.06
9.03
9.04
6.09
6.03
10.0
9.5
9.0
8.5
8.0
7.5
7.0
6.5
6.0
5.5
5.0
4.5
4.0
3.5
3.0
2.5
2.0
1.5
1.0
0.5
0.0
f1 (ppm)

$^{13}C\{^{1}H\}$ NMR (100.6 MHz, $CDCl_3$)

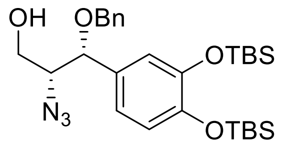

17

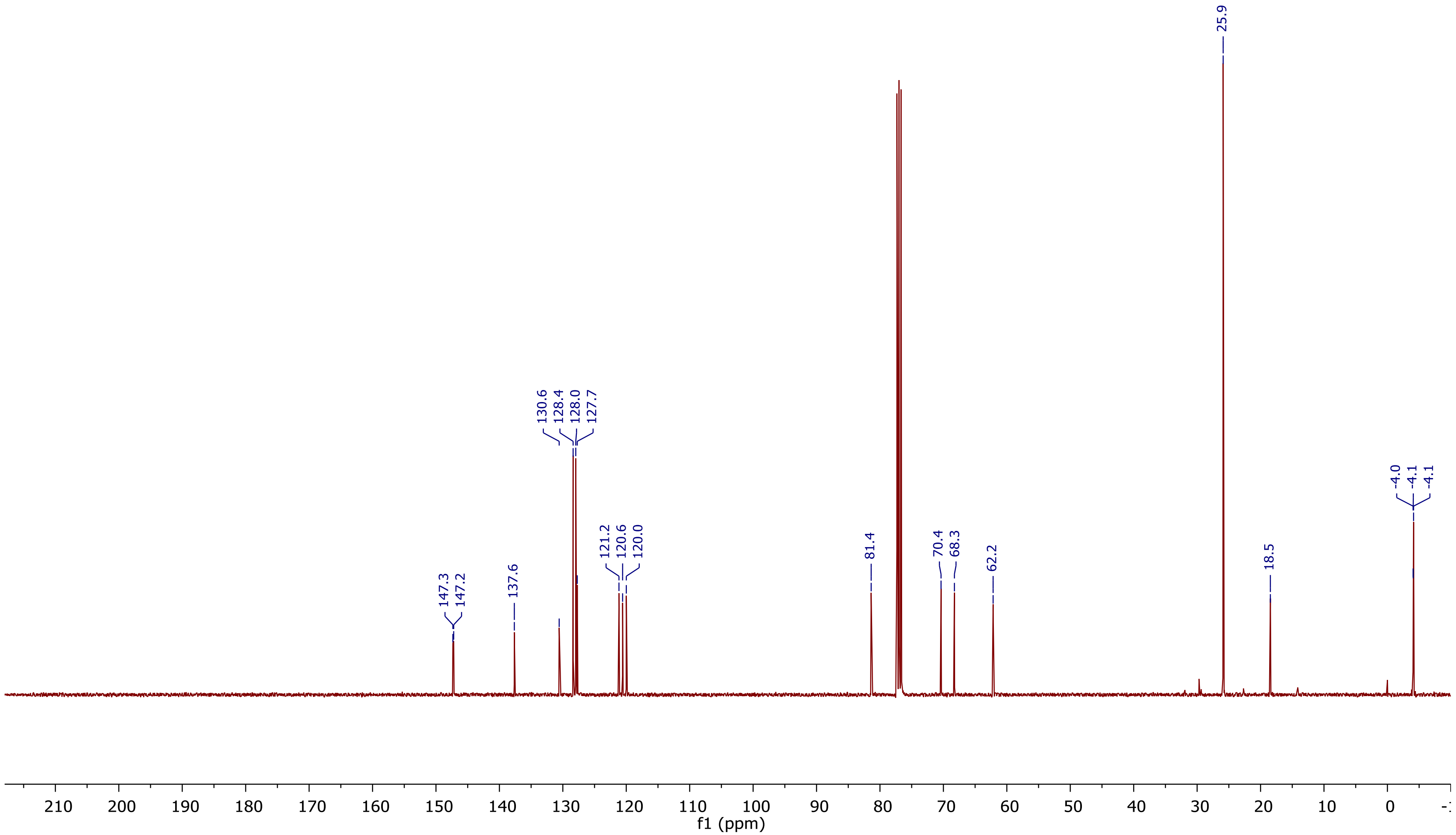

^{1}H - ^{1}H COSY NMR (400 MHz, $CDCl_3$)

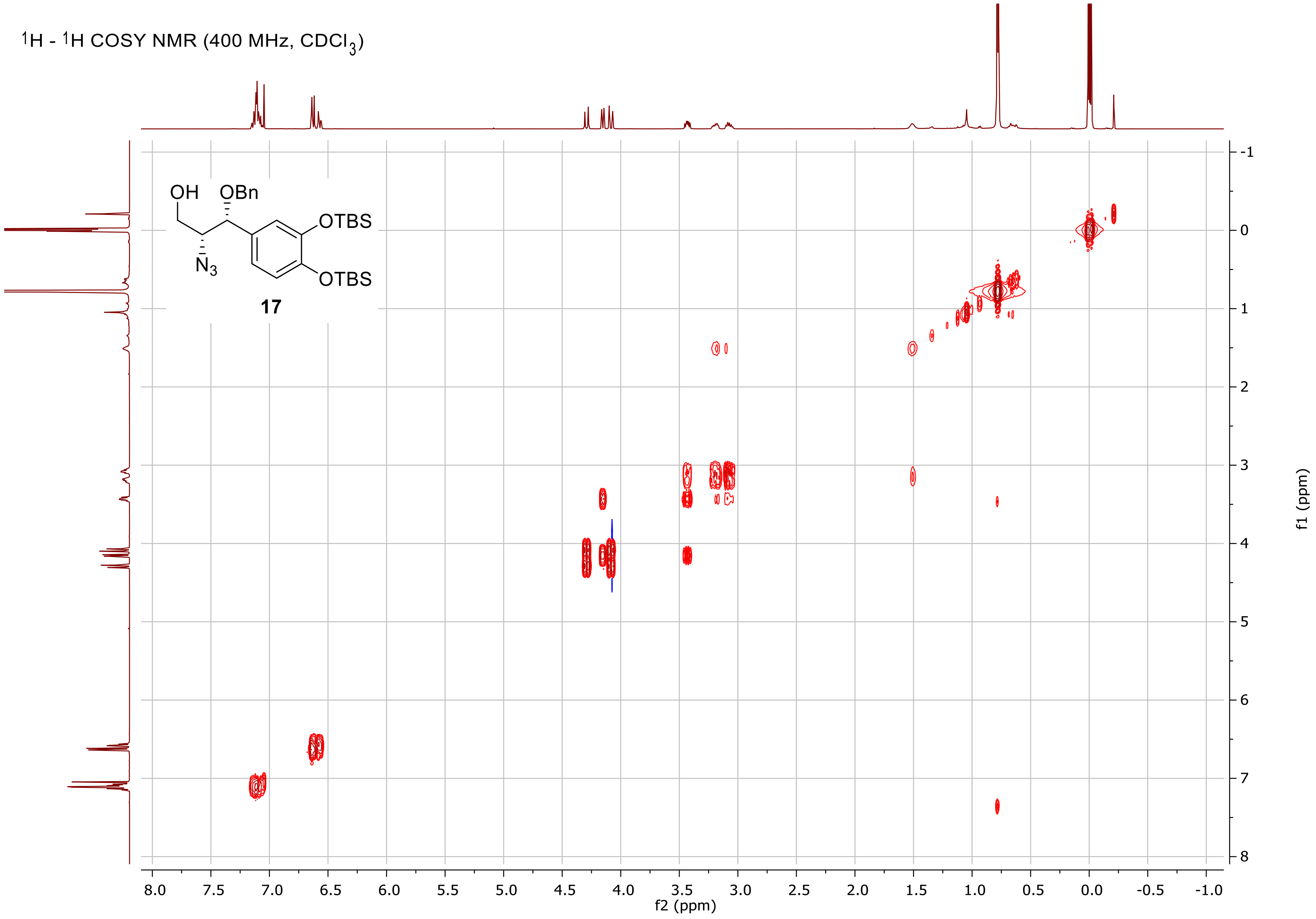

^{1}H - ^{13}C HSQC NMR (400 MHz, $CDCl_3$)

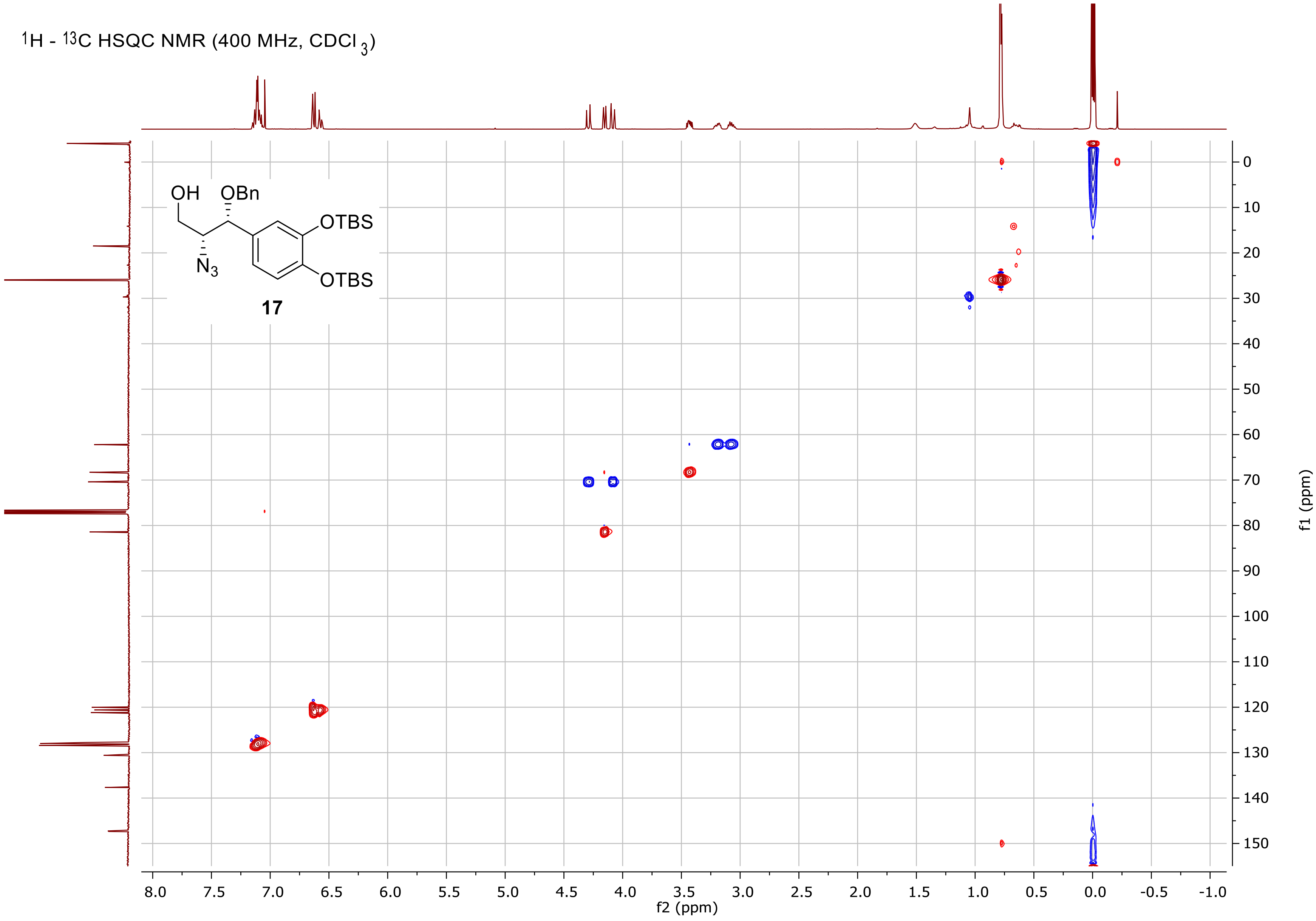

^{1}H NMR (400 MHz, $CDCl_3$)

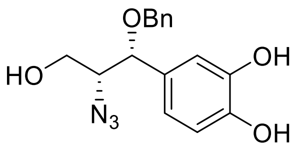

18

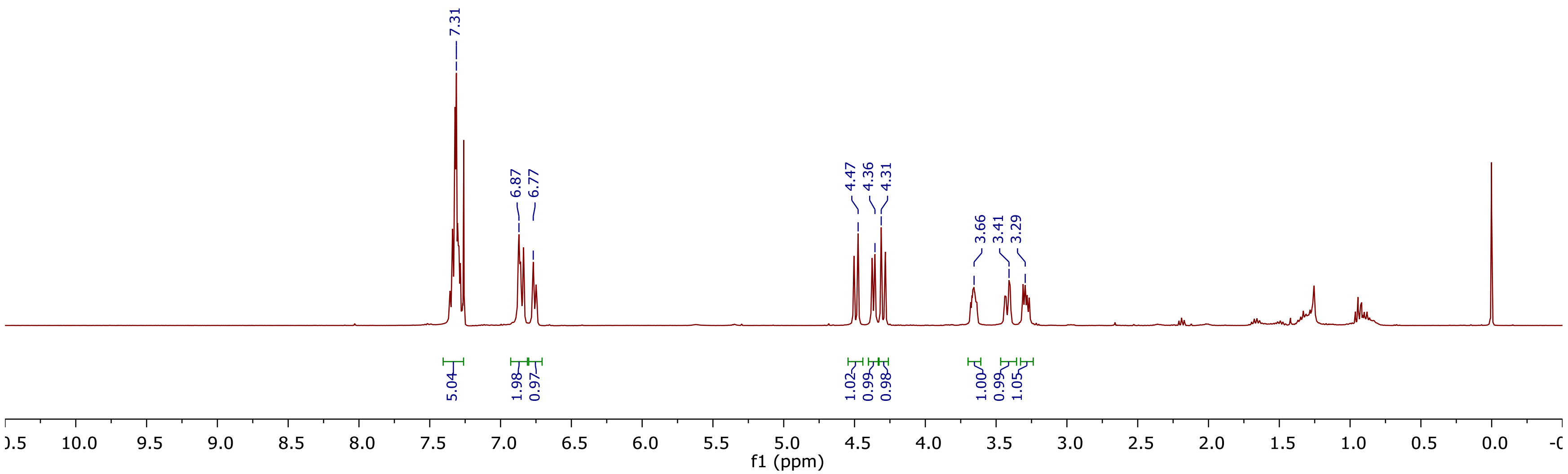

$^{13}C\{^1H\}$ NMR (100.6 MHz, $CDCl_3$)

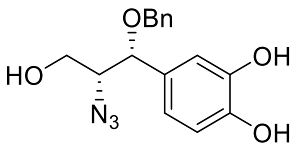

18

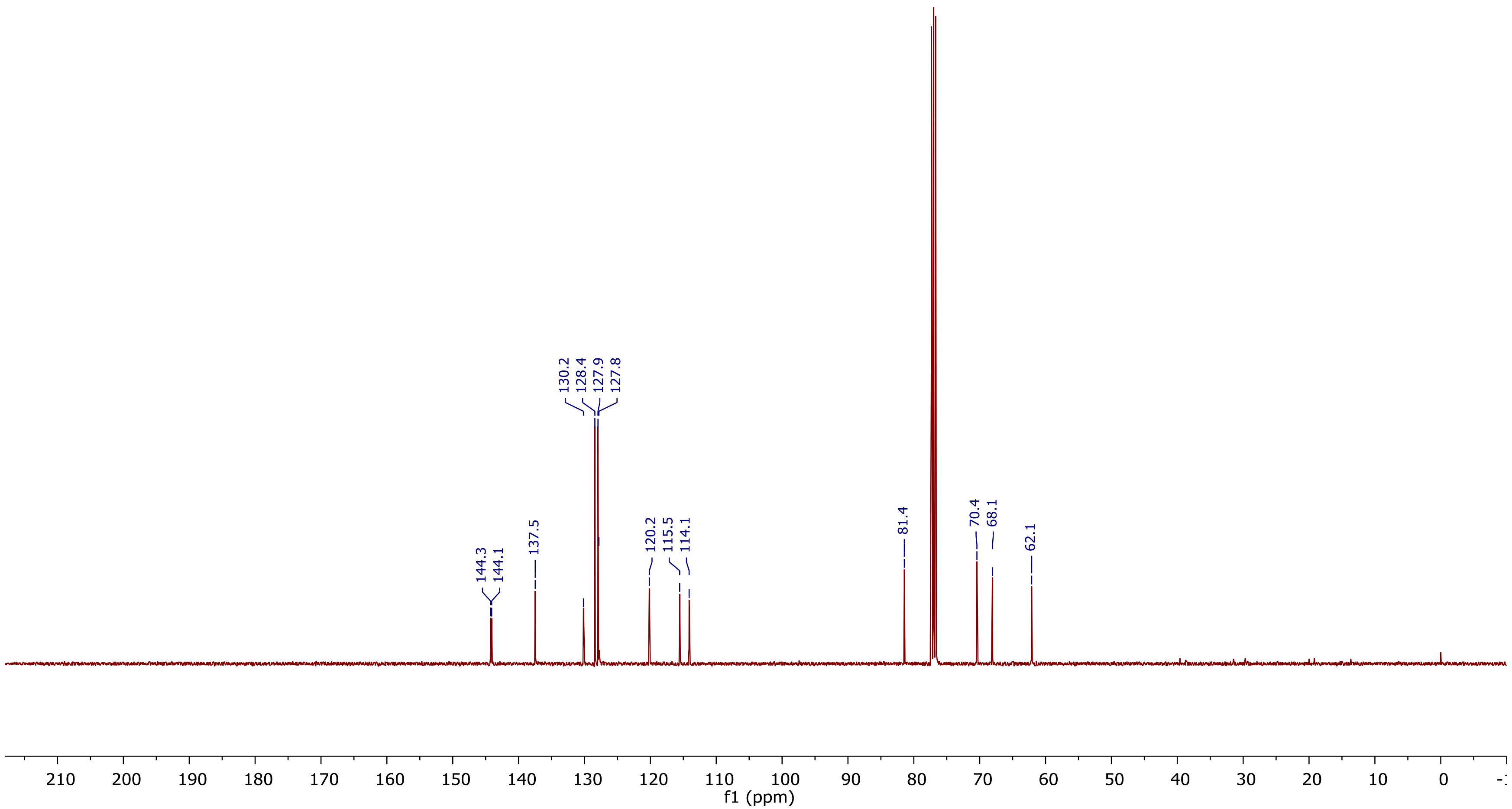

^1H - ^1H COSY NMR (400 MHz, $CDCl_3$)

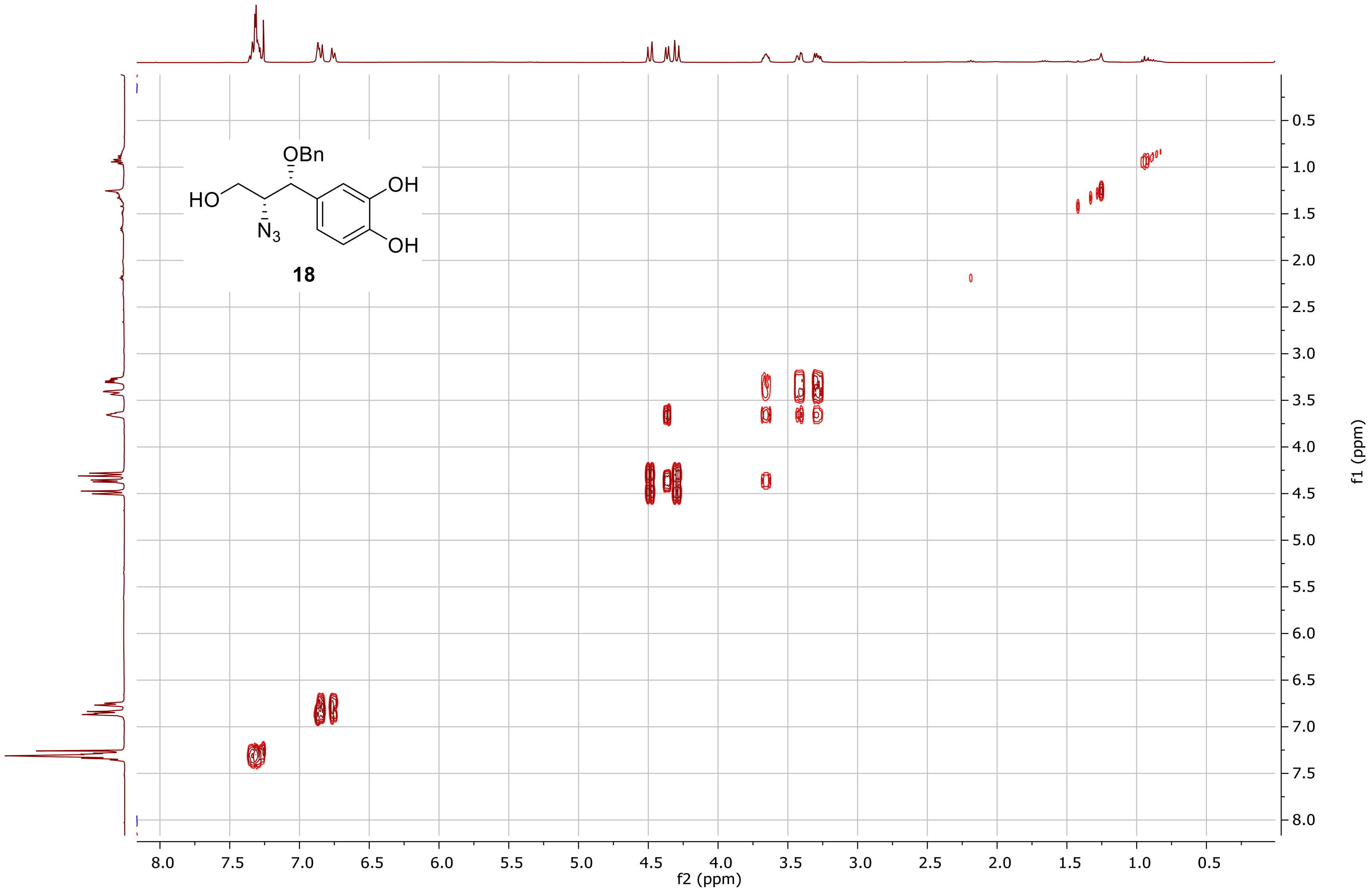

^{1}H - ^{13}C HSQC NMR (400 MHz, $CDCl_3$)

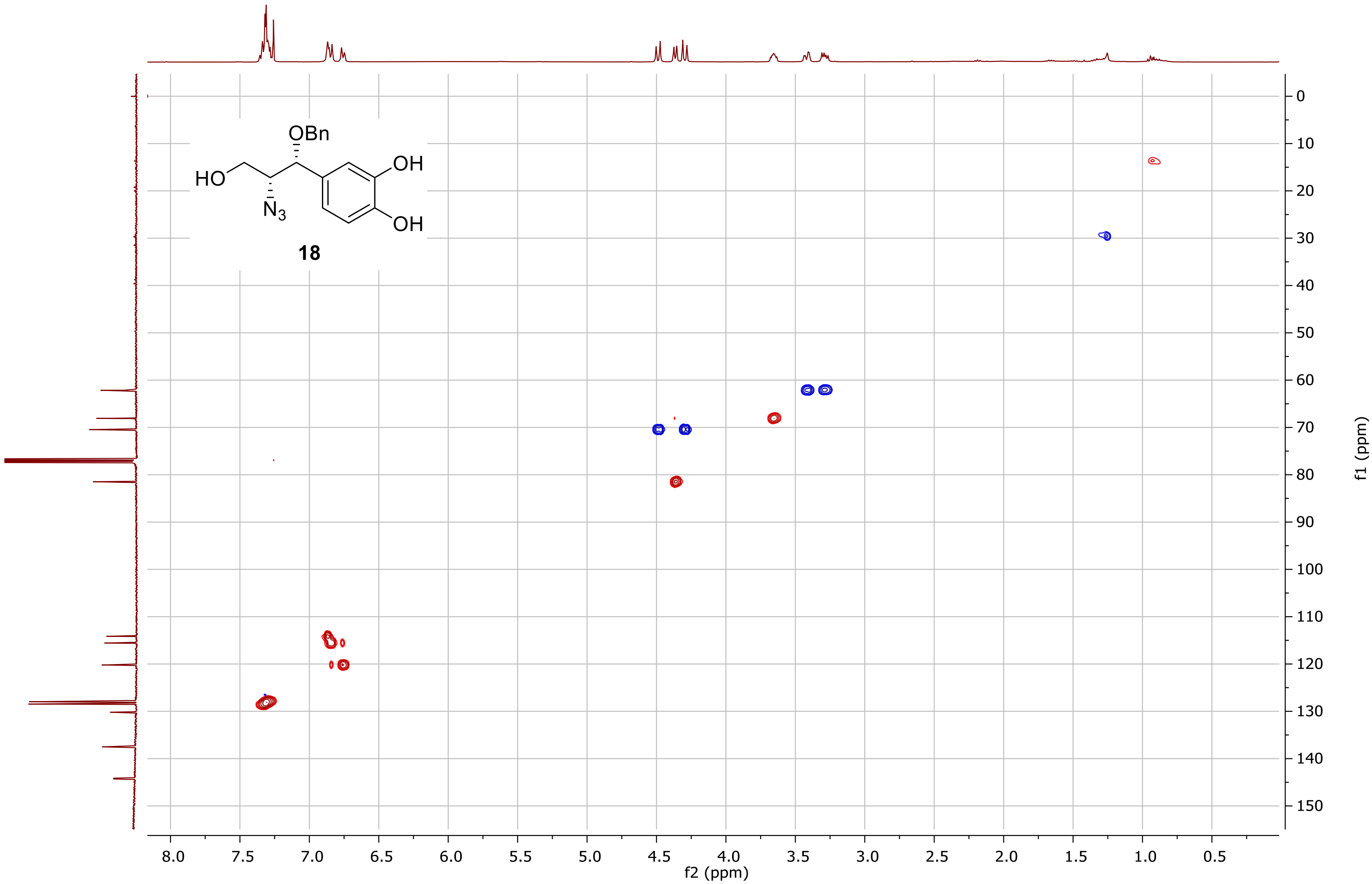

^{1}H NMR (400 MHz, $CDCl_3$)

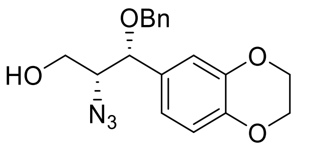

19

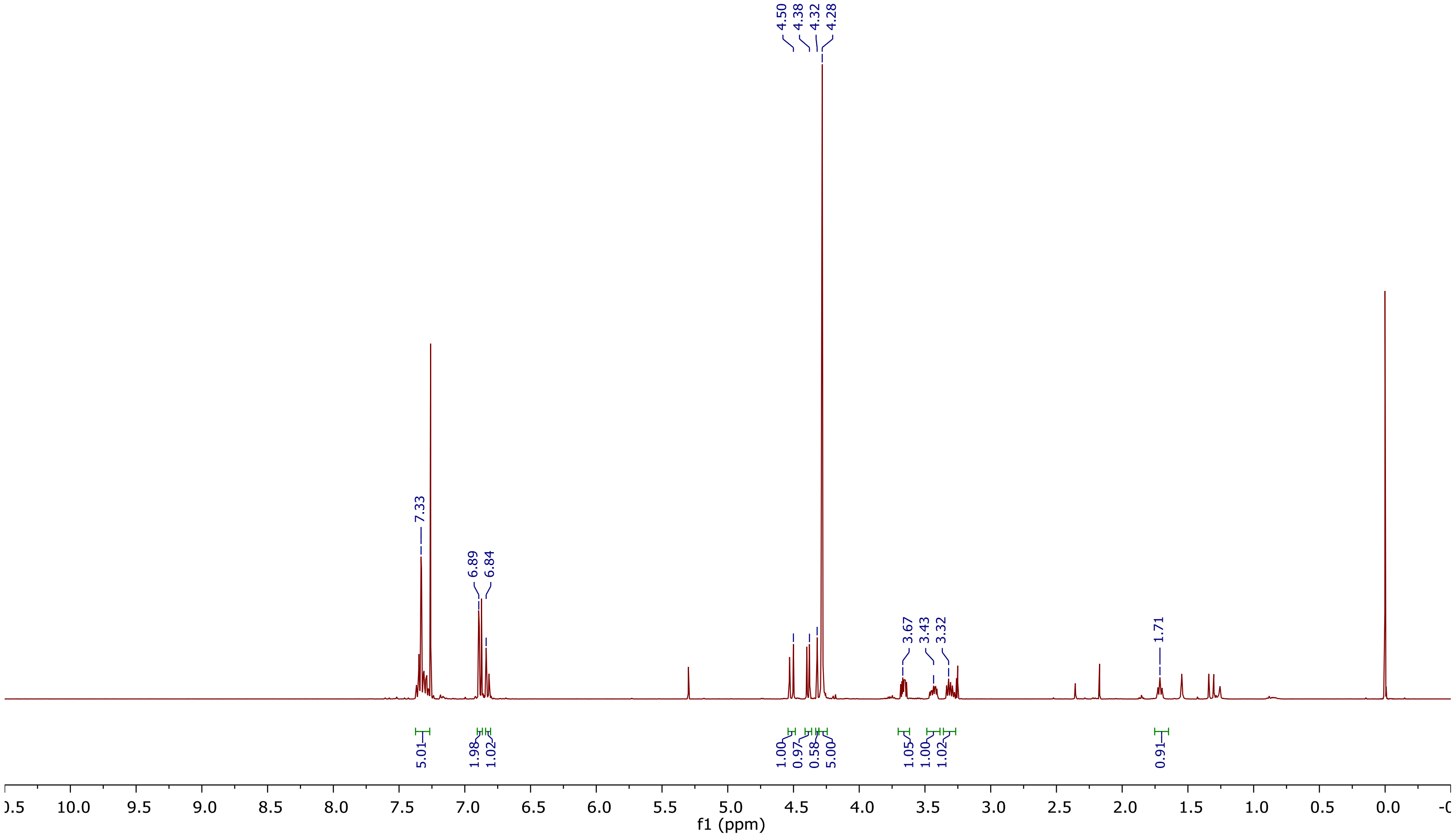

$^{13}C\{^1H\}$ NMR (100.6 MHz, $CDCl_3$)

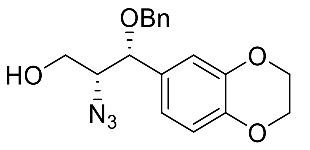

19

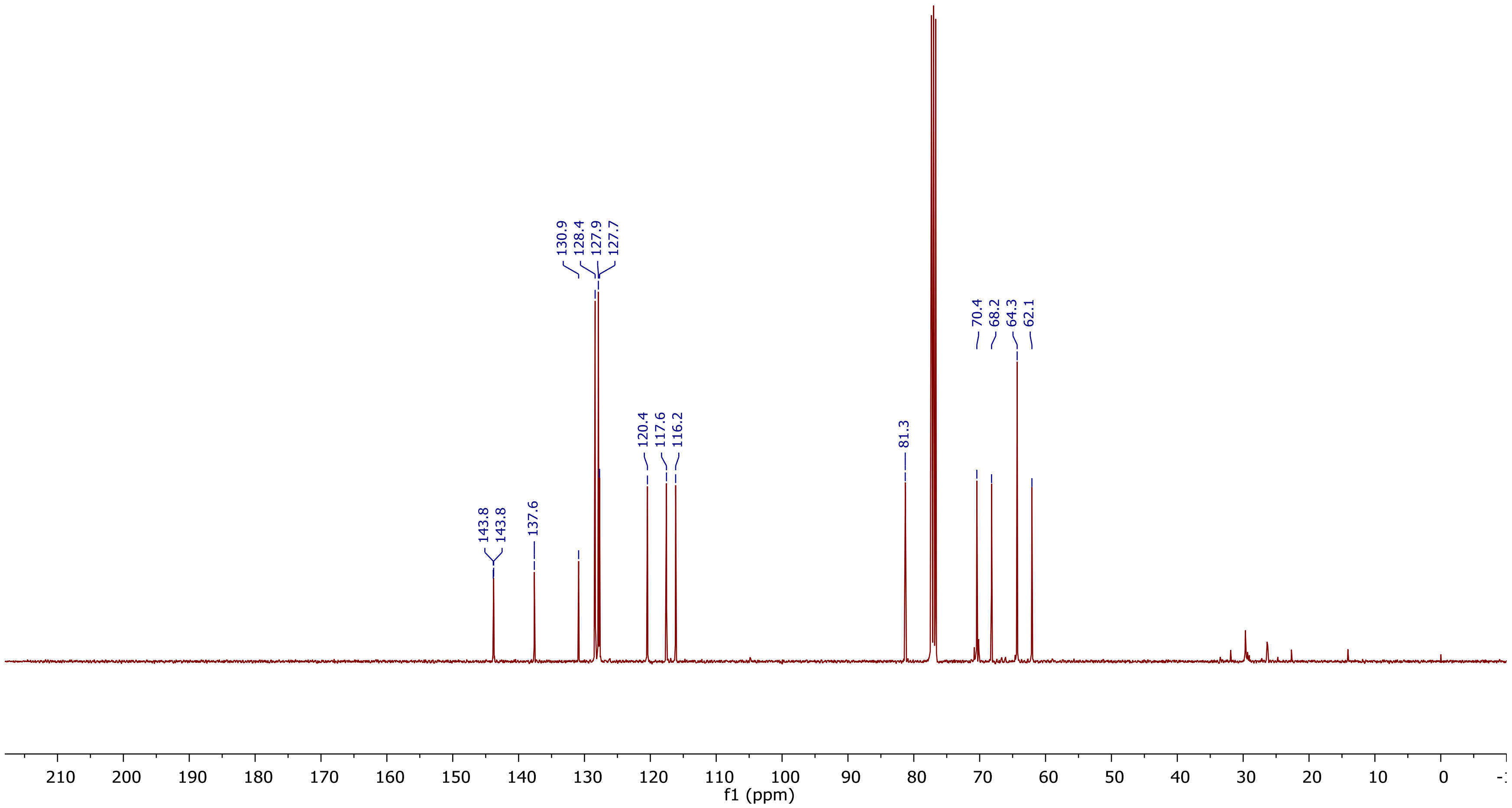

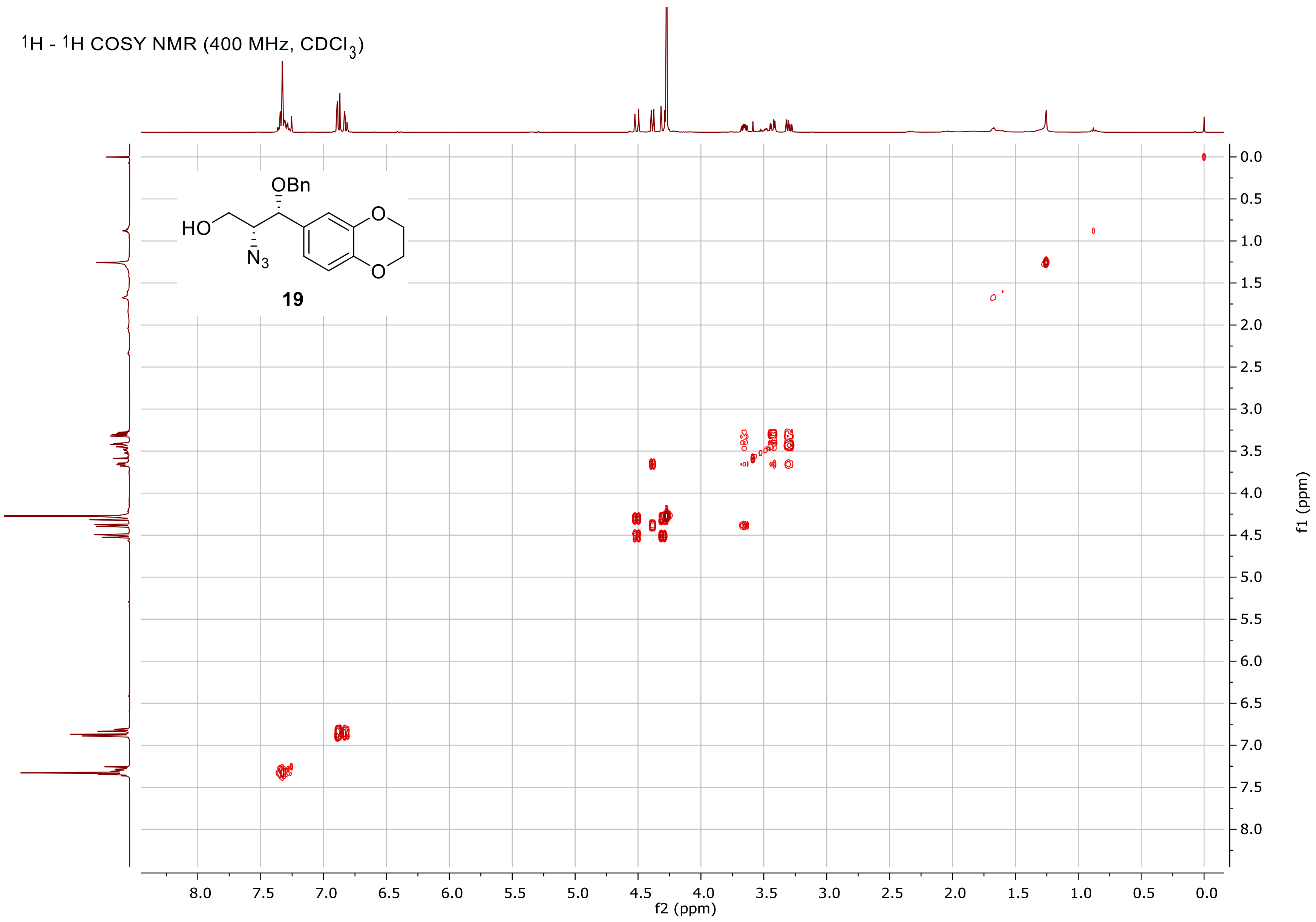

^{1}H - ^{1}H COSY NMR (400 MHz, $CDCl_3$)
OBn
HO
N_3
O
O
19
f2 (ppm)
f1 (ppm)

^{1}H - ^{13}C HSQC NMR (400 MHz, $CDCl_3$)

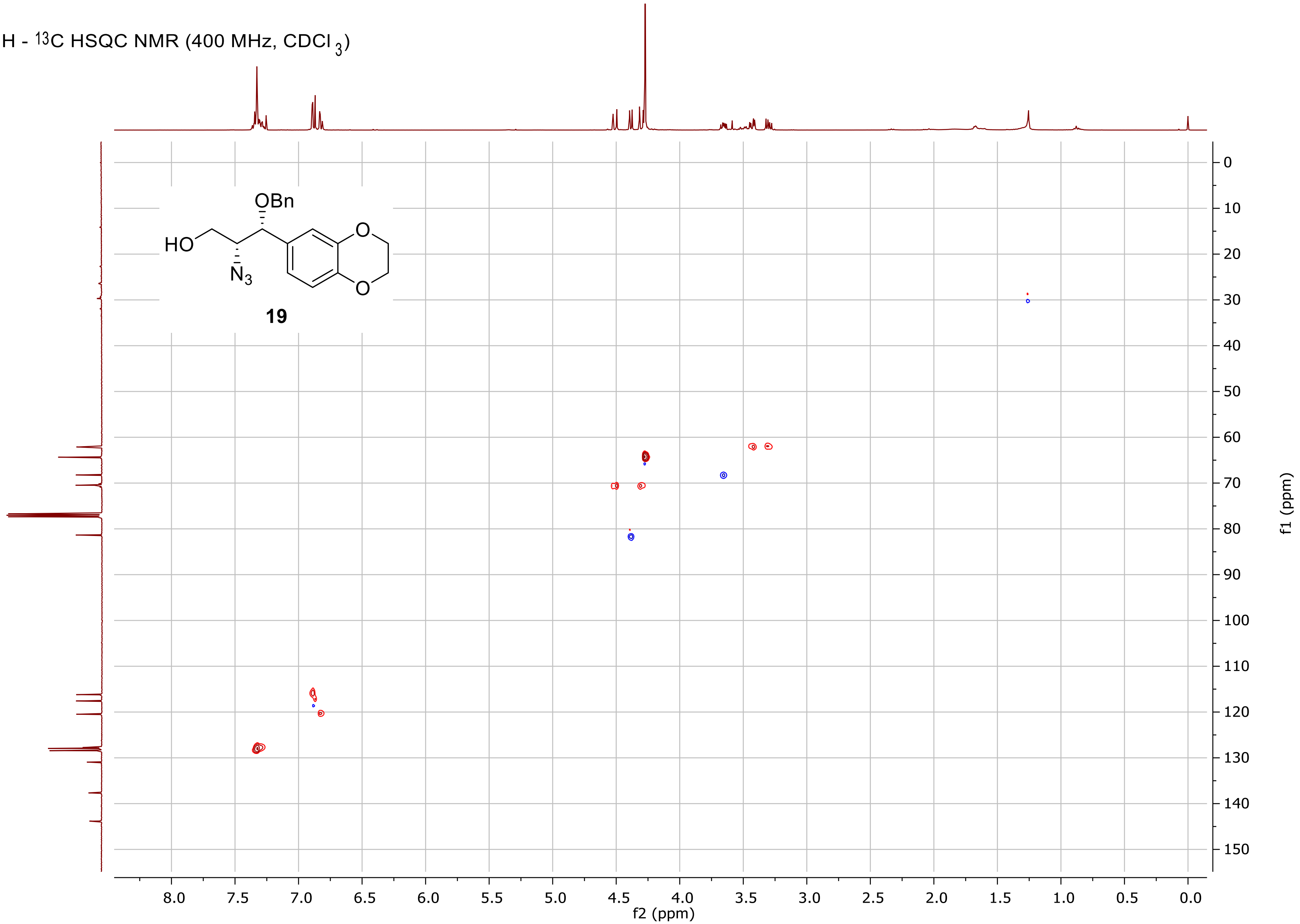

^{1}H NMR (400 MHz, $CDCl_3$)

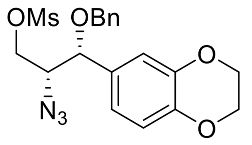

B

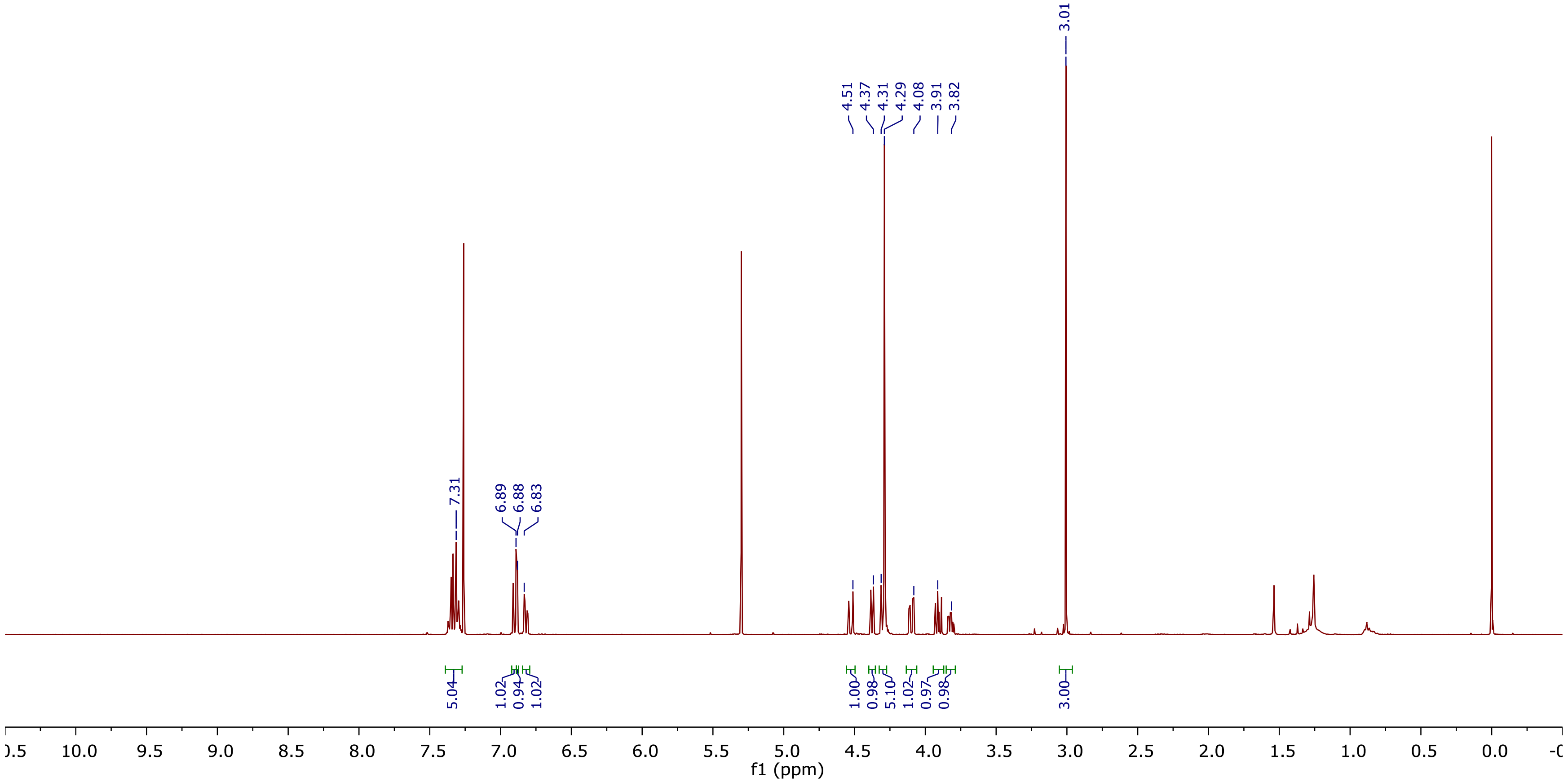

$^{13}C\{^{1}H\}$ NMR (100.6 MHz, $CDCl_3$)

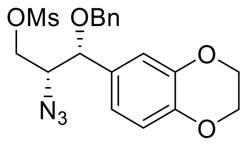

B

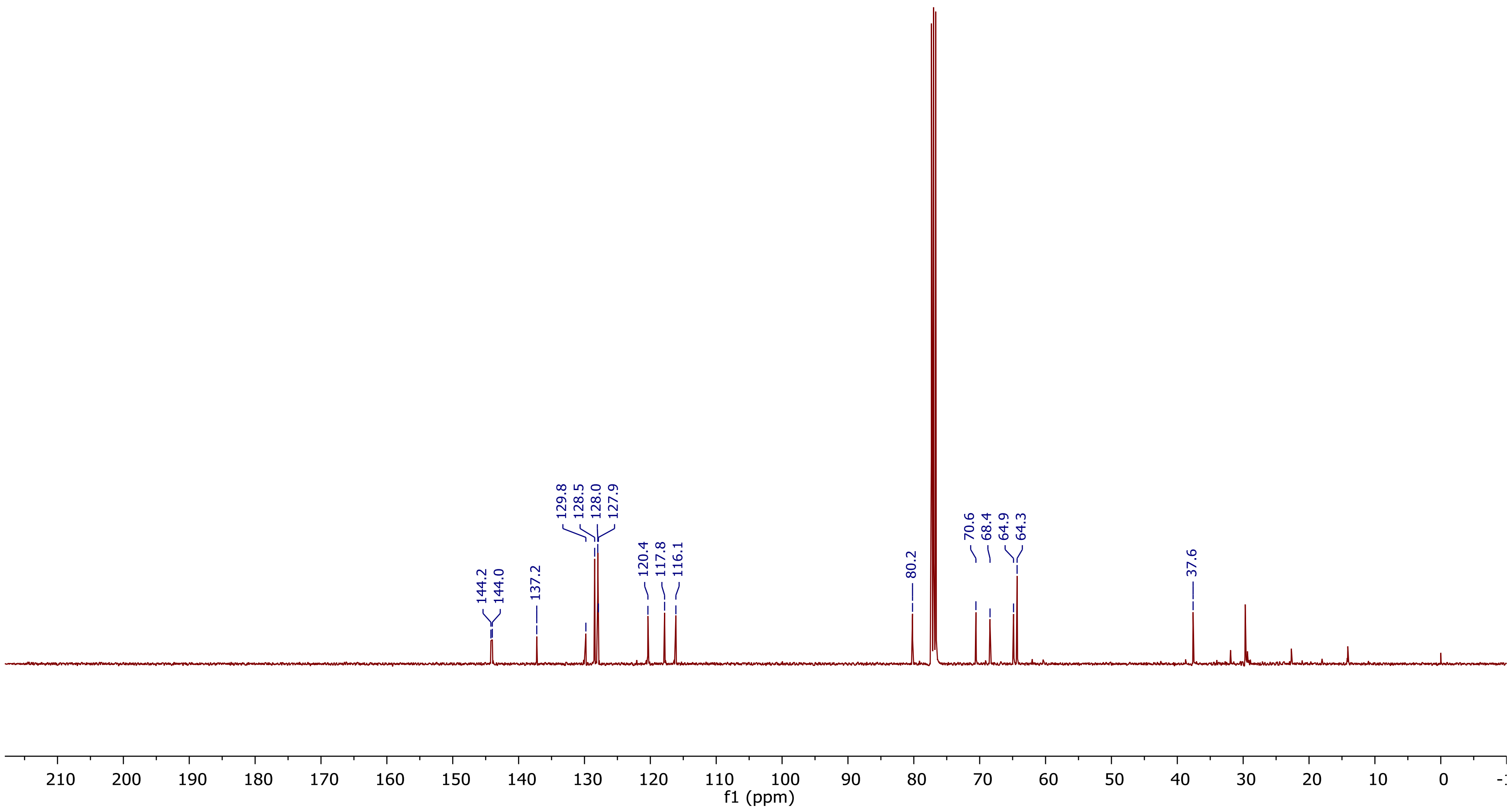

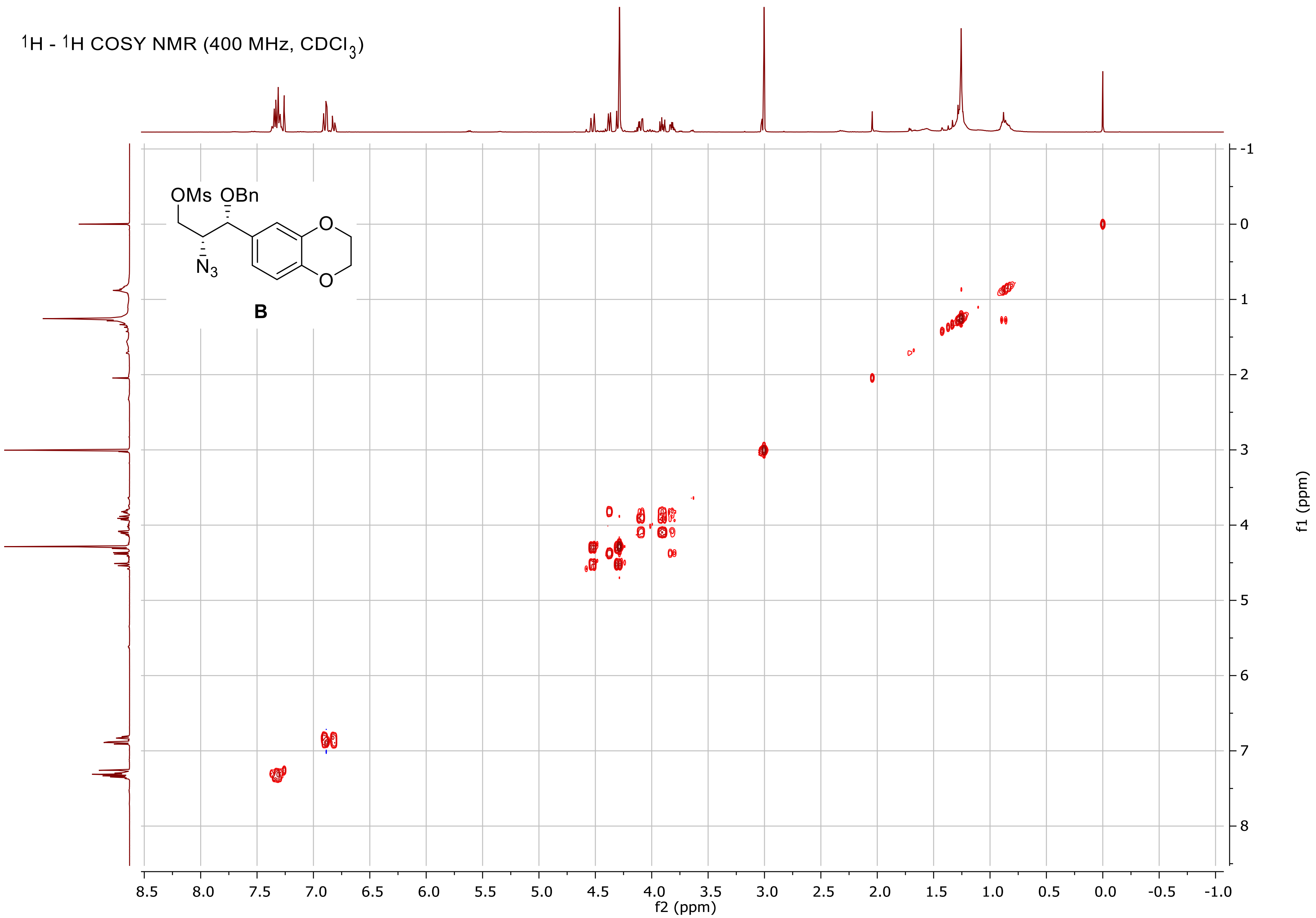

^{1}H - ^{1}H COSY NMR (400 MHz, $CDCl_3$)
OMs
OBn
N_3
O
O
B
f2 (ppm)
f1 (ppm)

^1H - ^{13}C HSQC NMR (400 MHz, $CDCl_3$)

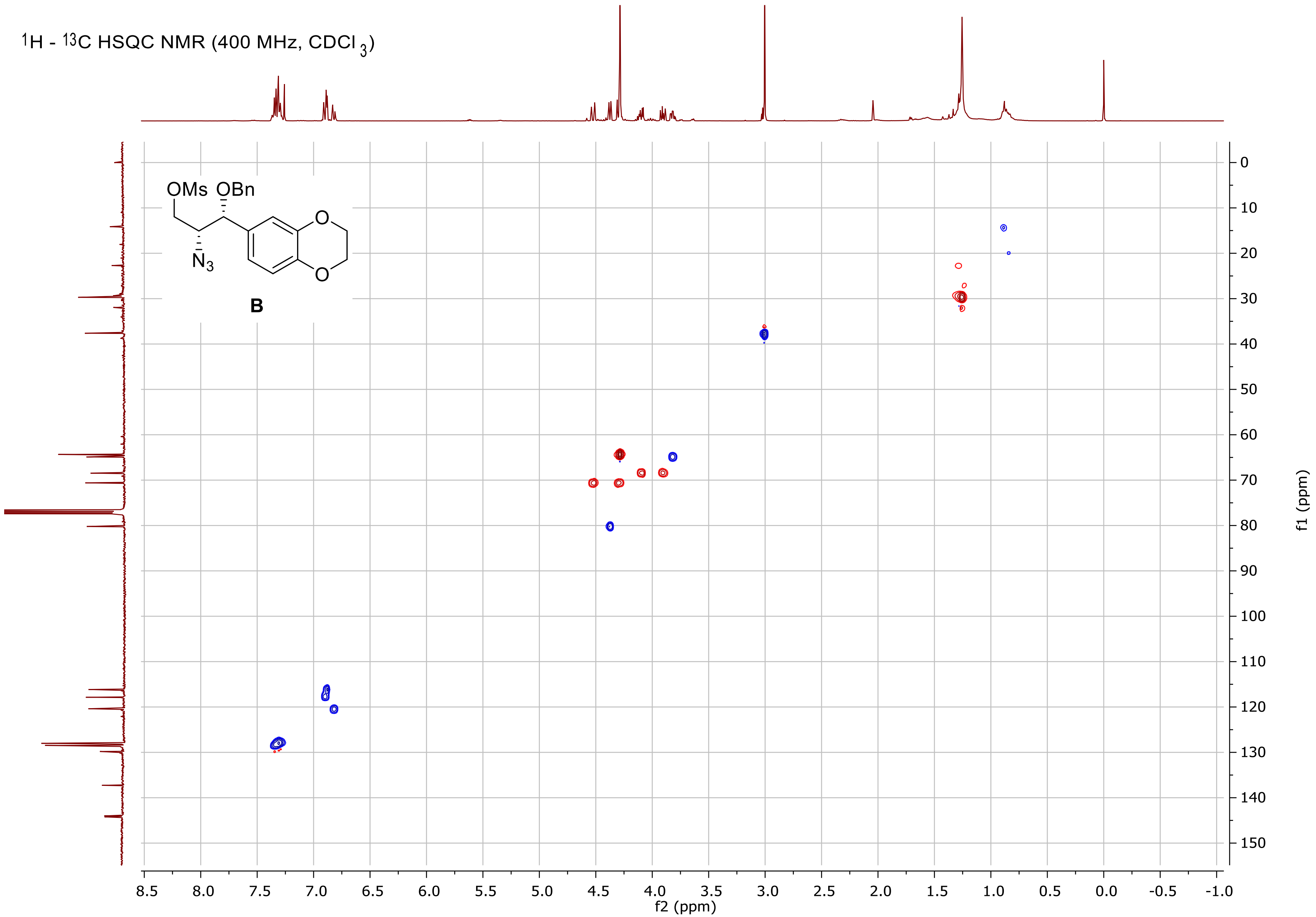

^1H NMR (400 MHz, $CDCl_3$)

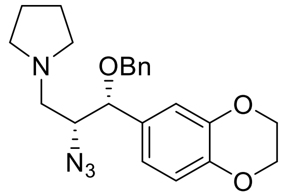

10

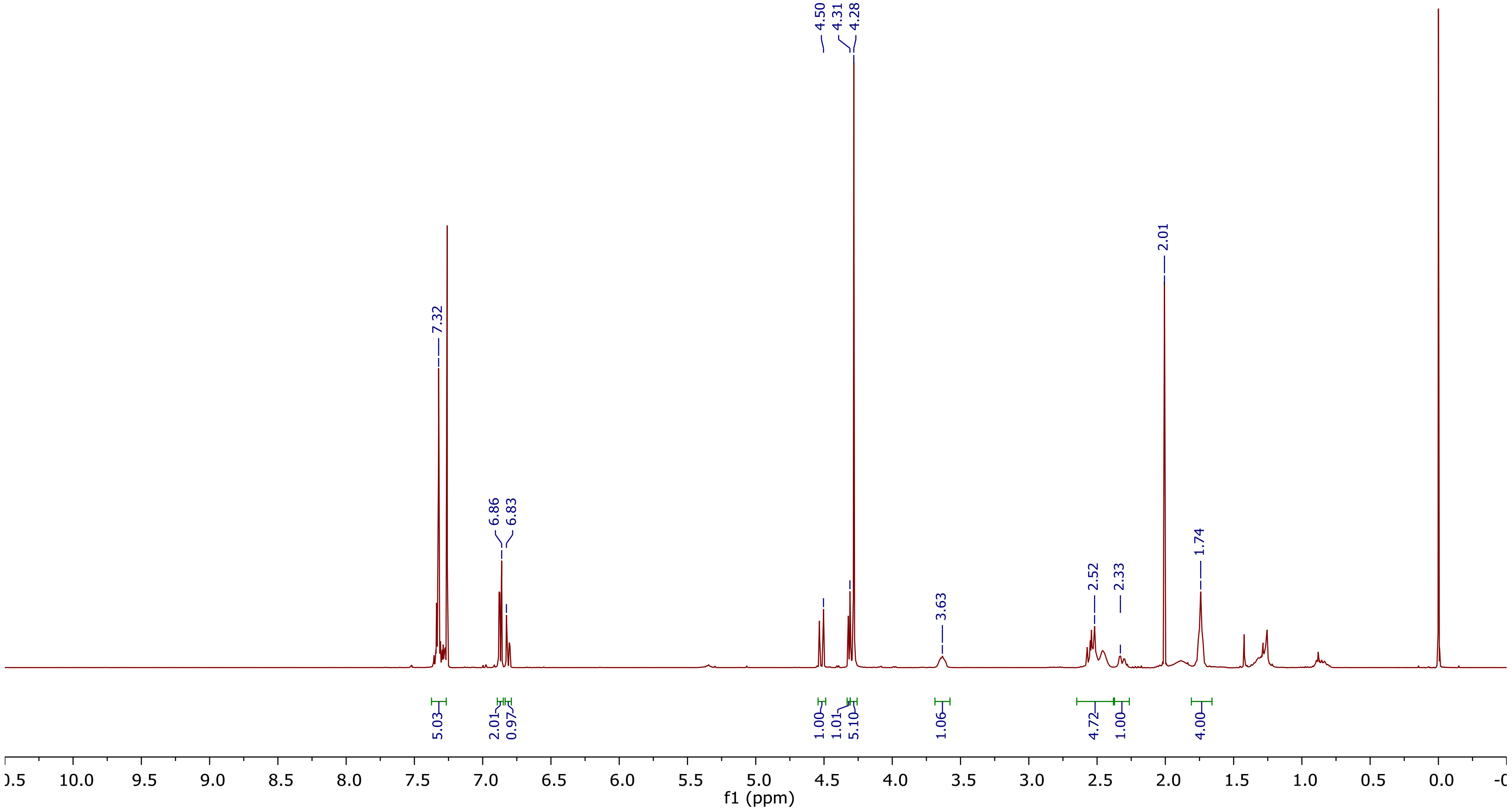

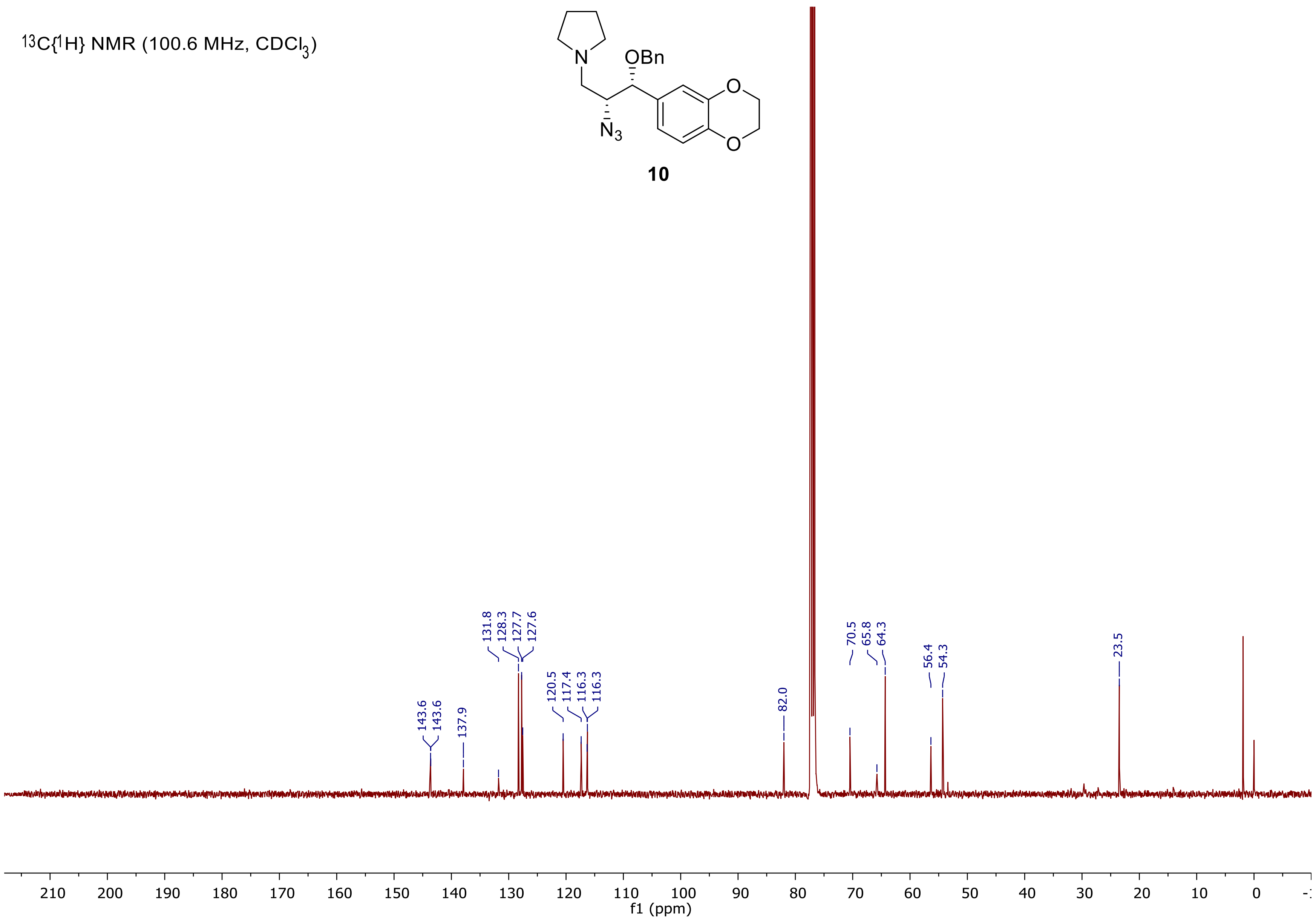
13C{1H} NMR (100.6 MHz, CDCl3)
OBn
N3
O
N
10
143.6
143.6
137.9
131.8
128.3
127.7
127.6
120.5
117.4
116.3
116.3
82.0
70.5
65.8
64.3
56.4
54.3
23.5
210
200
190
180
170
160
150
140
130
120
110
100
90
80
70
60
50
40
30
20
10
0
f1 (ppm)

^1H - ^1H COSY NMR (400 MHz, $CDCl_3$)

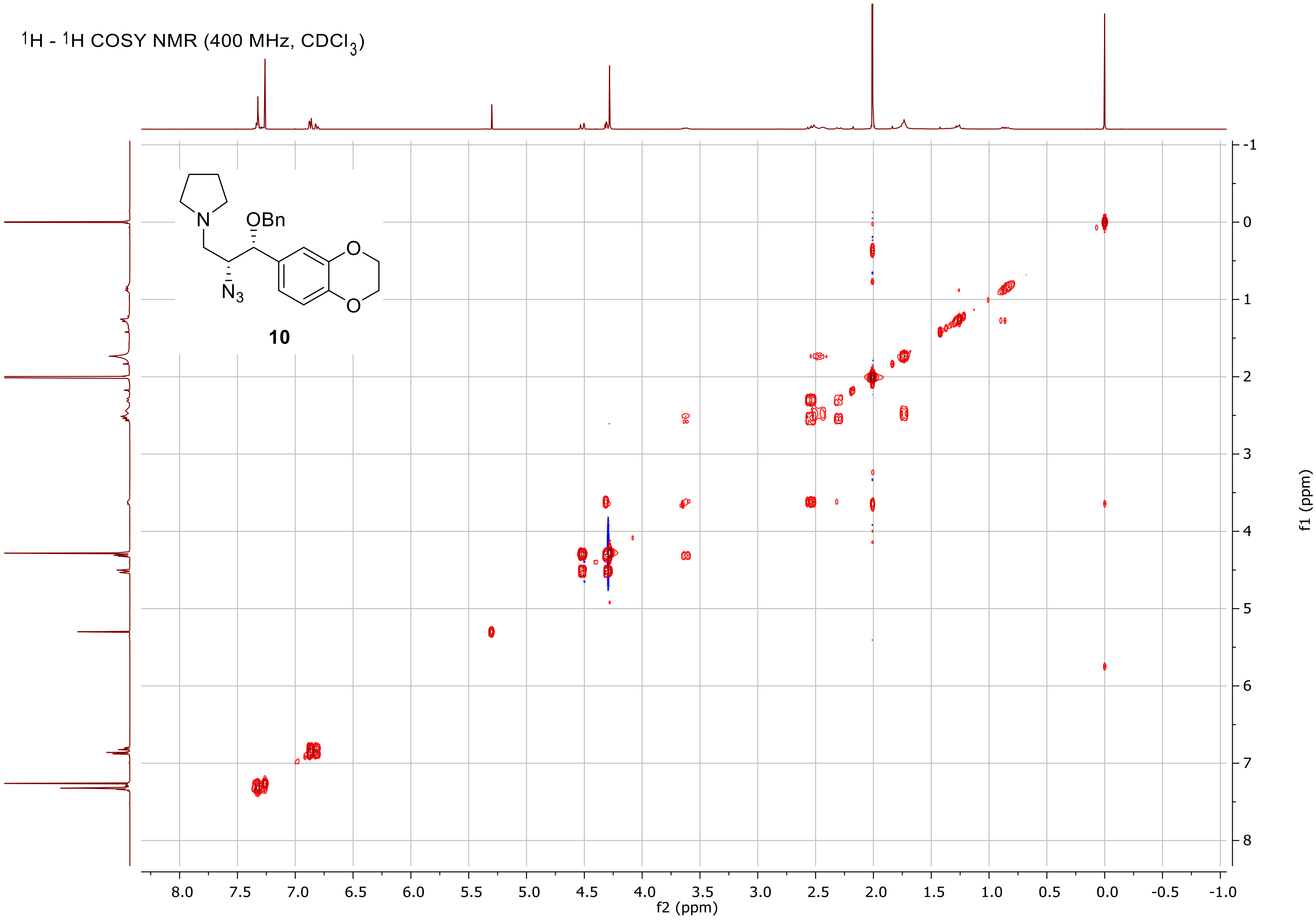

^{1}H - ^{13}C HSQC NMR (400 MHz, $CDCl_3$)

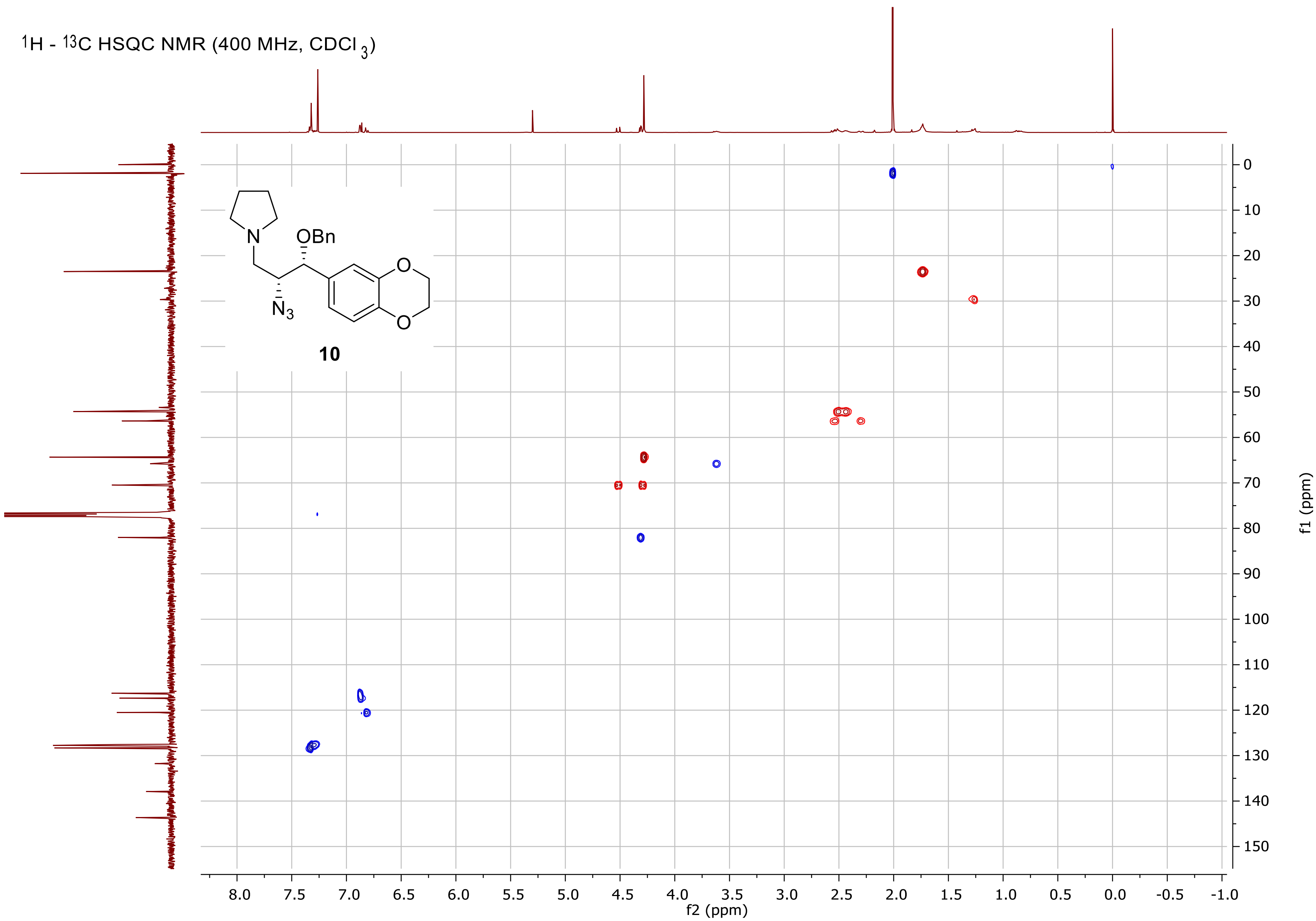

^1H NMR (400 MHz, $CDCl_3$)

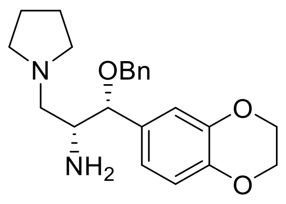

C

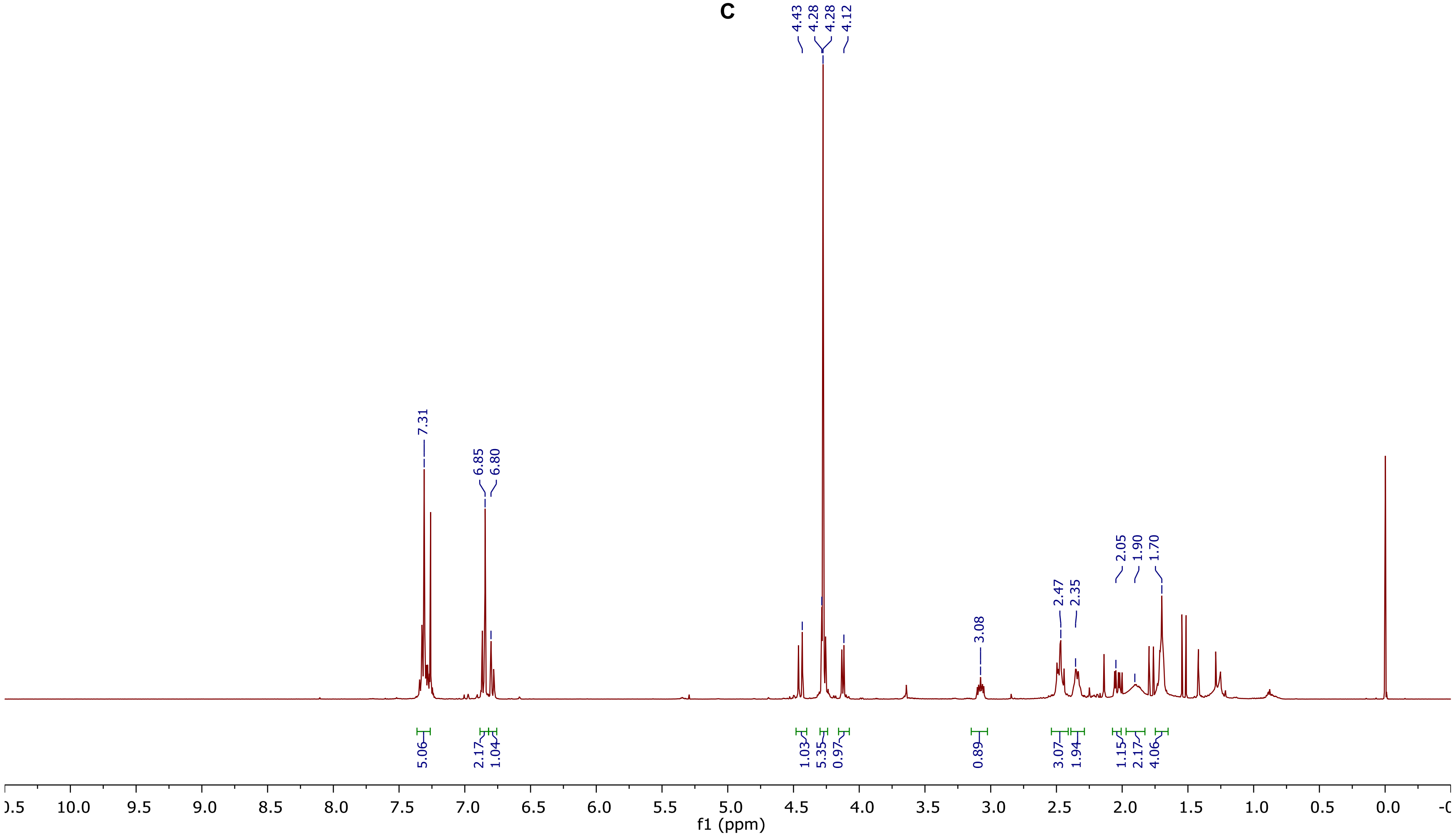

$^{13}C\{^1H\}$ NMR (100.6 MHz, $CDCl_3$)

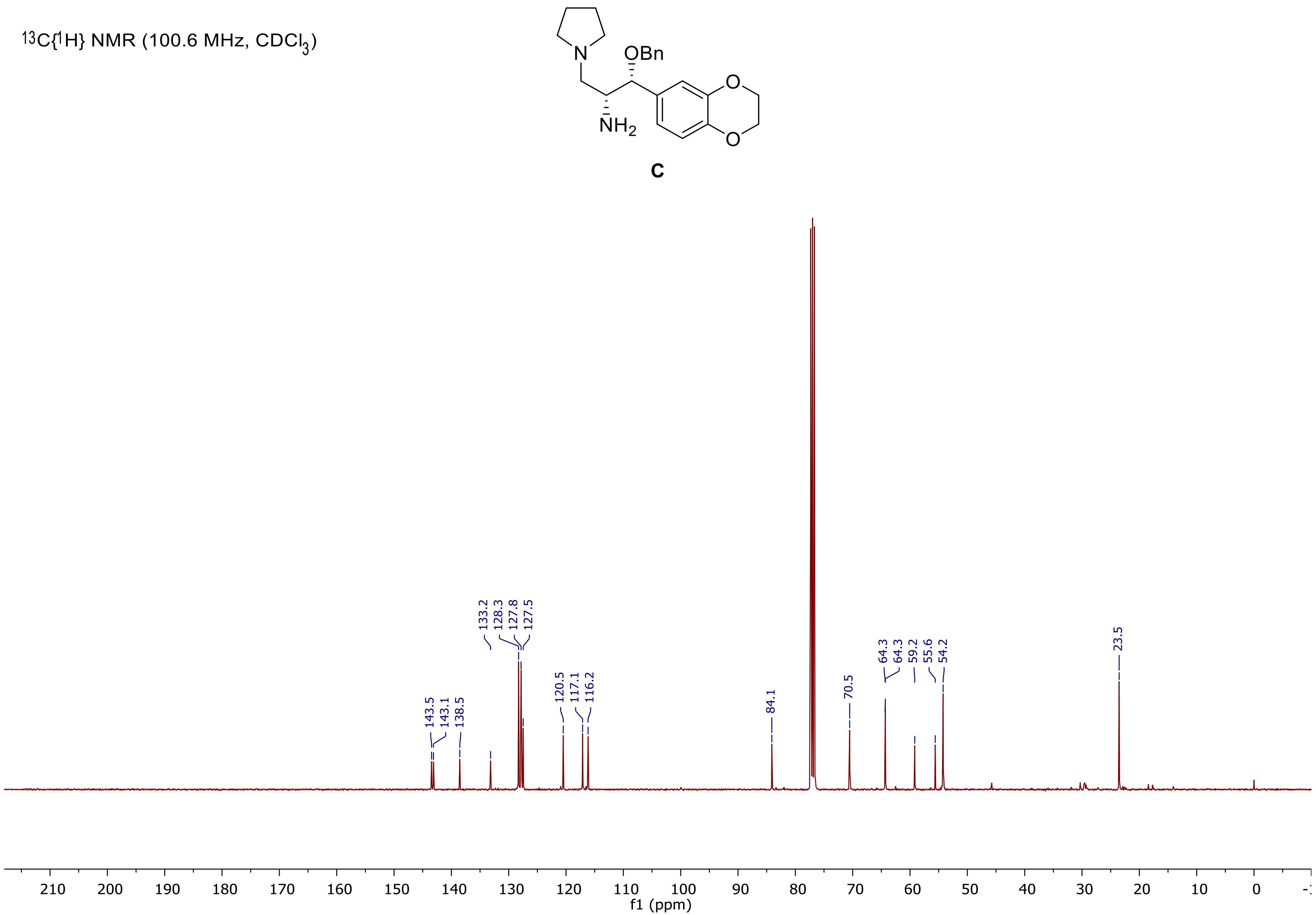

^1H - ^1H COSY NMR (400 MHz, $CDCl_3$)

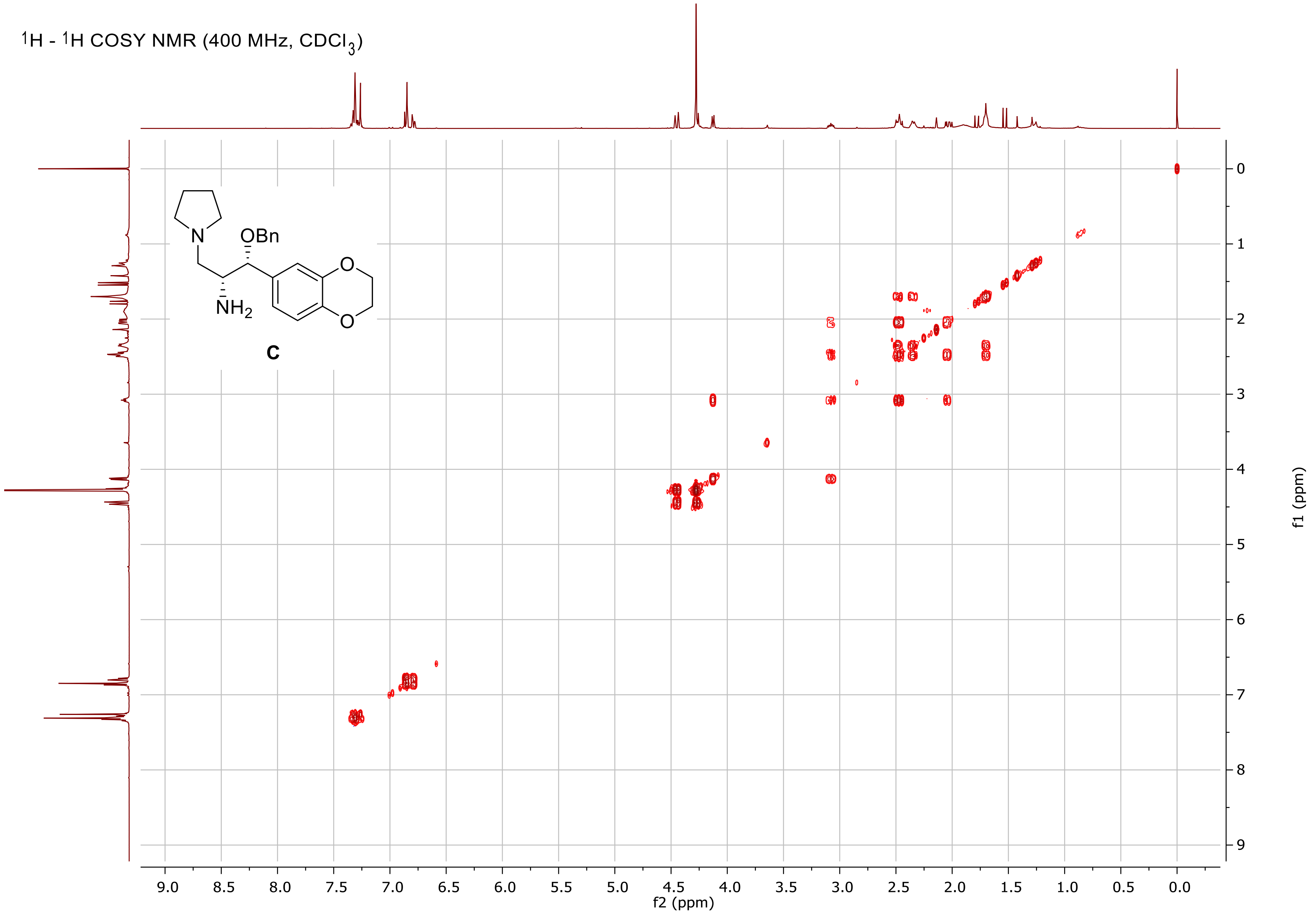

^{1}H - ^{13}C HSQC NMR (400 MHz, $CDCl_3$)

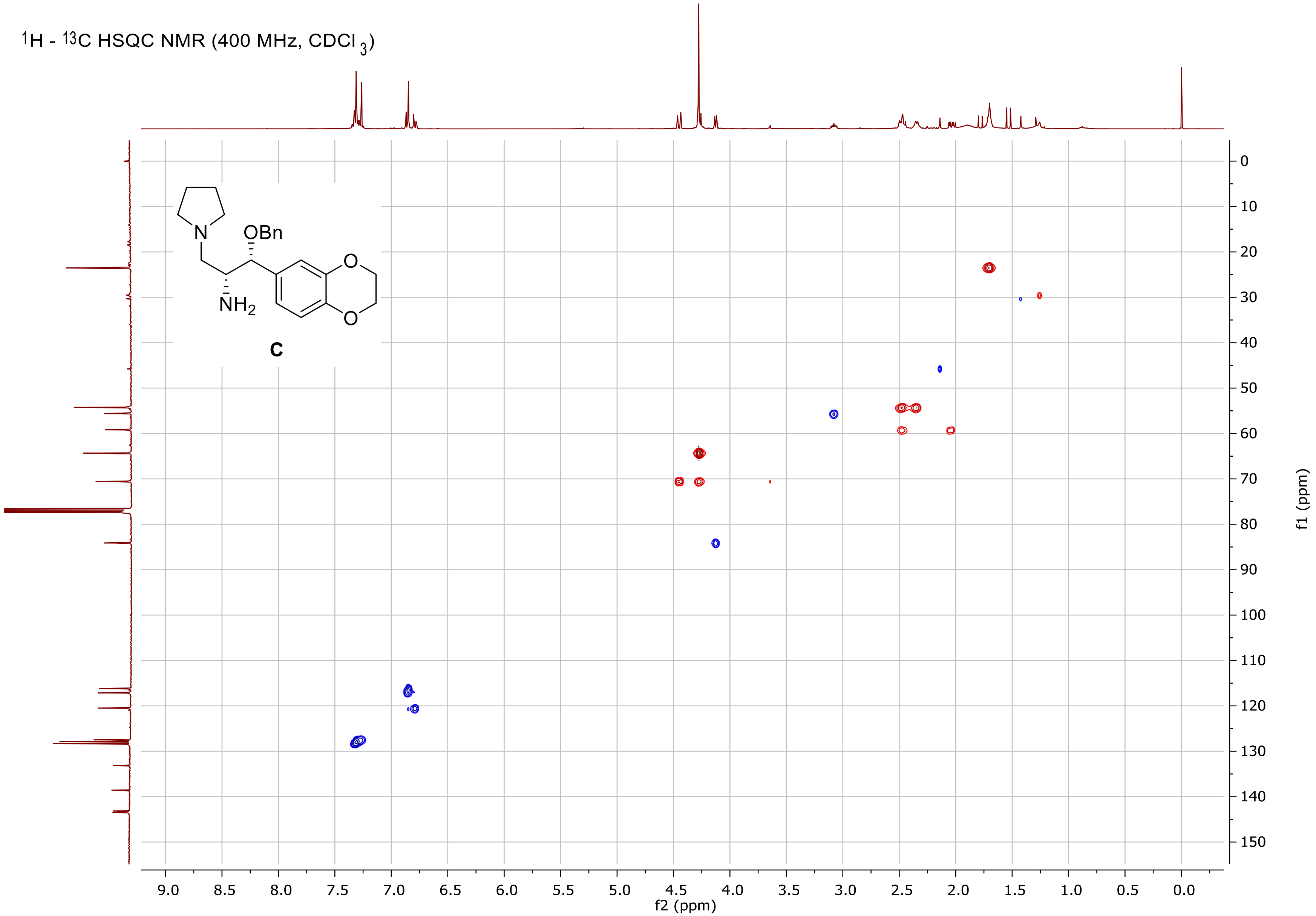

^1H NMR (400 MHz, $CDCl_3$)

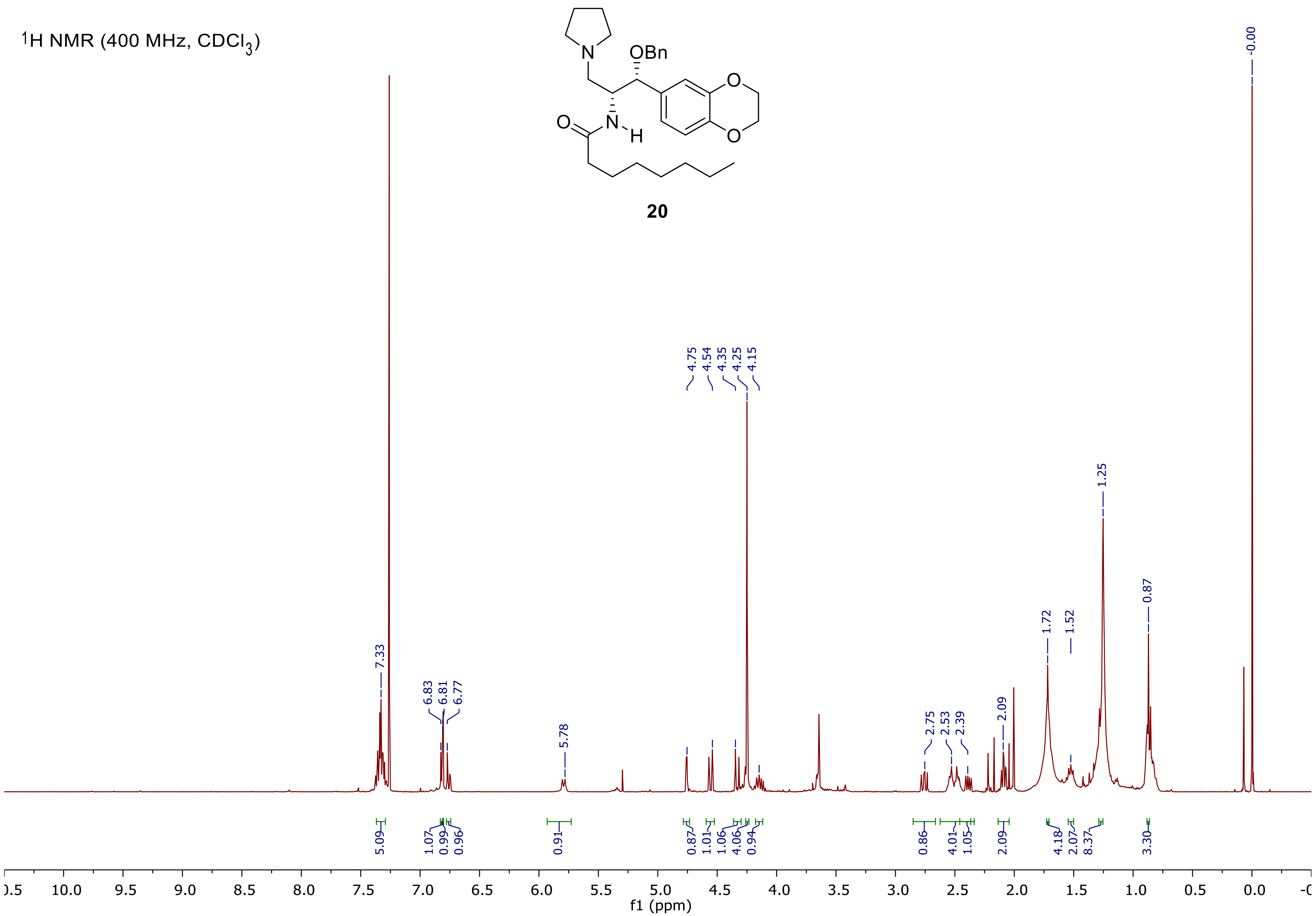

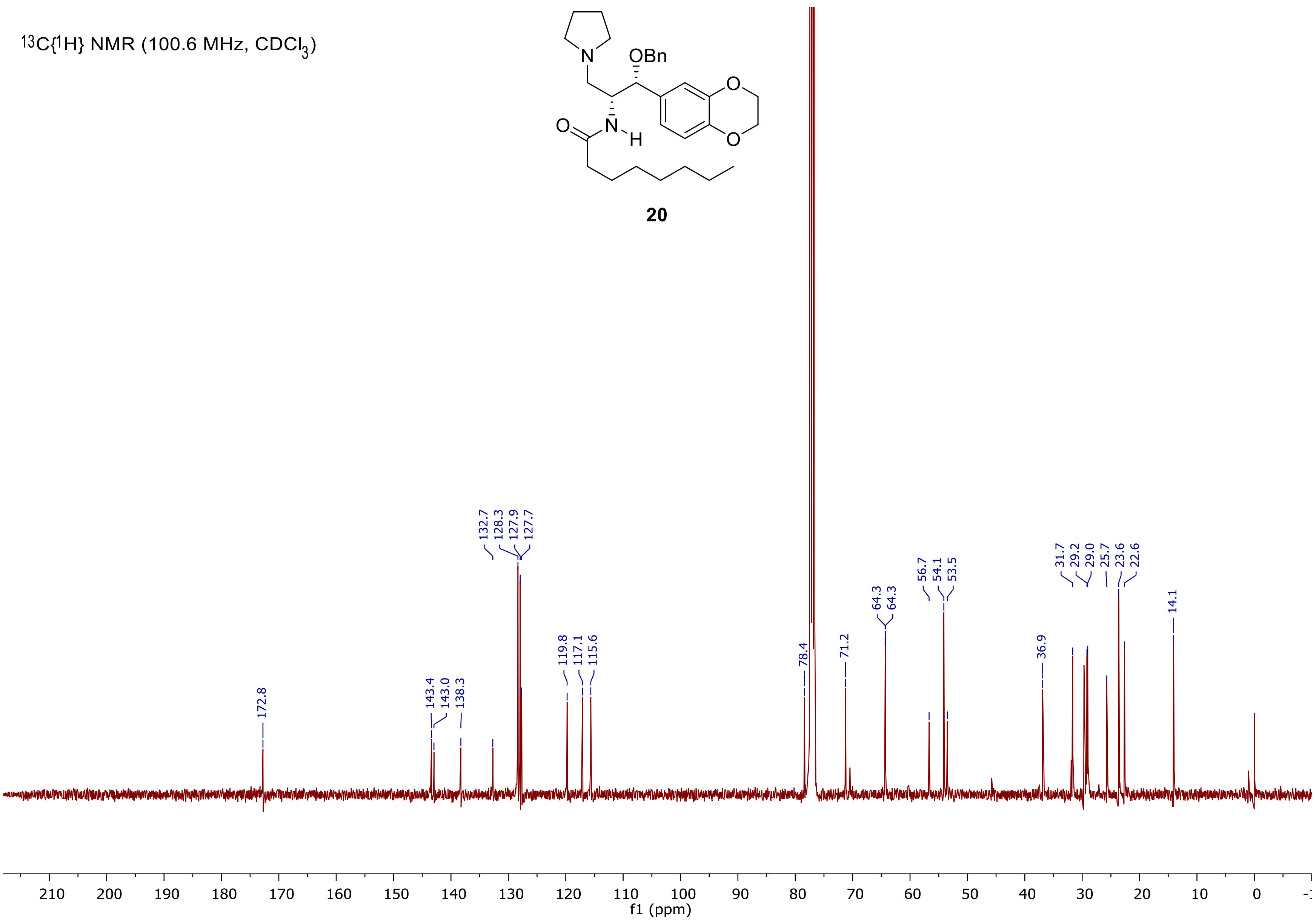

13C{1H} NMR (100.6 MHz, CDCl3)
OBn
N
O
H
20
172.8
143.4
143.0
138.3
132.7
128.3
127.9
127.7
119.8
117.1
115.6
78.4
71.2
64.3
64.3
56.7
54.1
53.5
36.9
31.7
29.2
29.0
25.7
23.6
22.6
14.1
210 200 190 180 170 160 150 140 130 120 110 100 90 80 70 60 50 40 30 20 10 0
f1 (ppm)

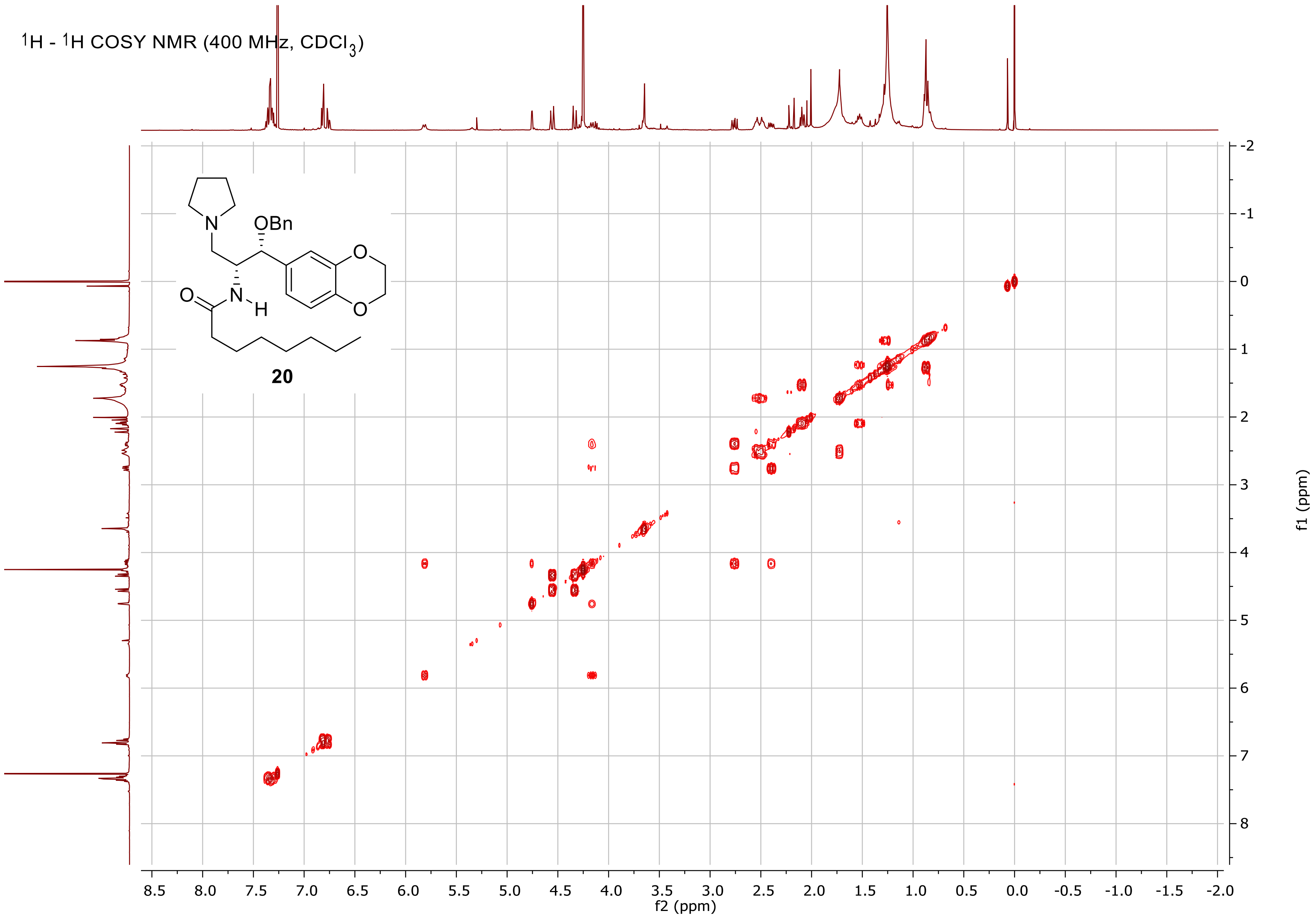

^{1}H - ^{1}H COSY NMR (400 MHz, CDCl$_3$)
N
OBn
O
O
O
N
H
20
f2 (ppm)
f1 (ppm)

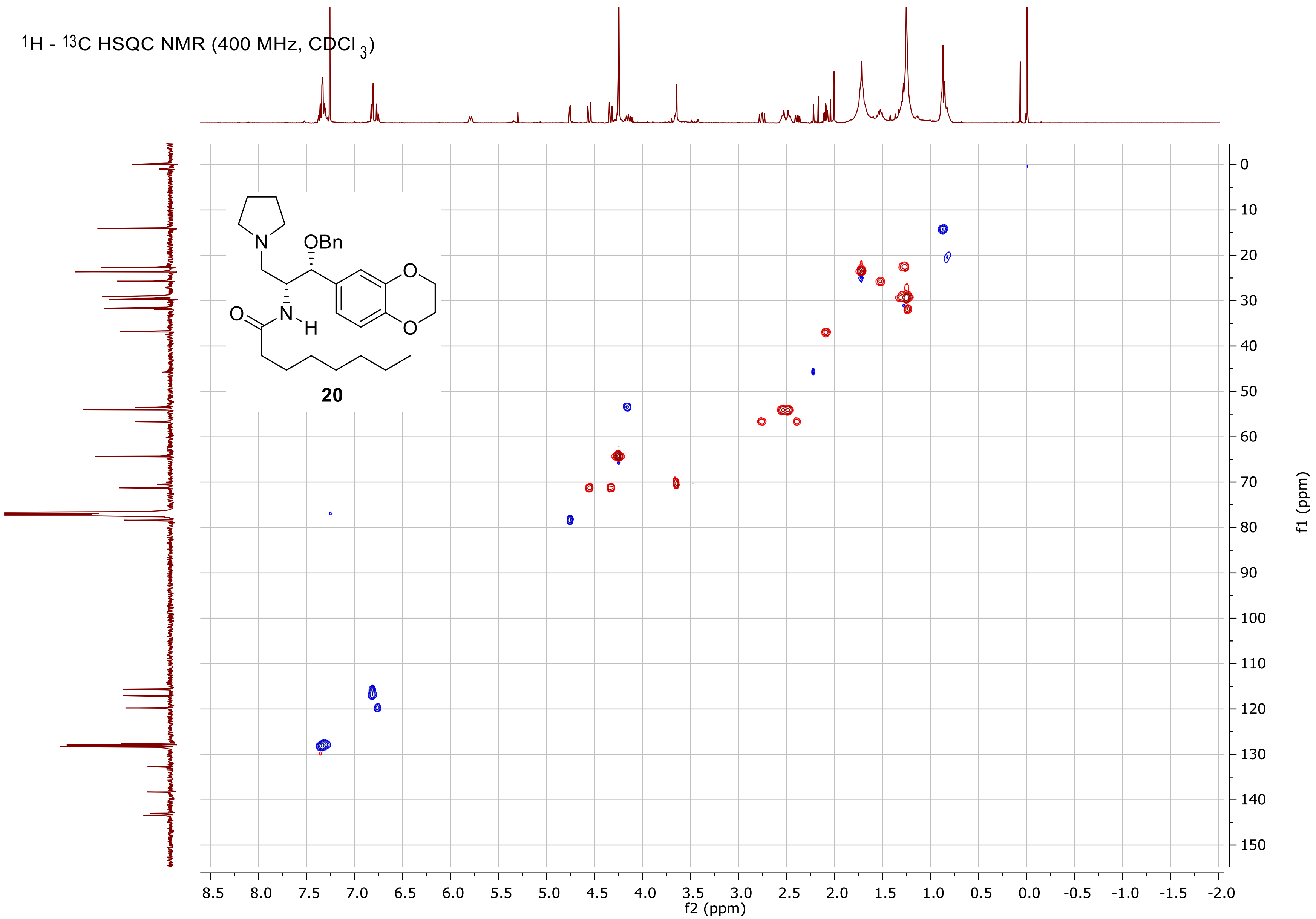

1H - 13C HSQC NMR (400 MHz, CDCl 3)
OBn
N
O
O
O
N
H
20
f2 (ppm)
f1 (ppm)

^{1}H NMR (400 MHz, $CDCl_3$)

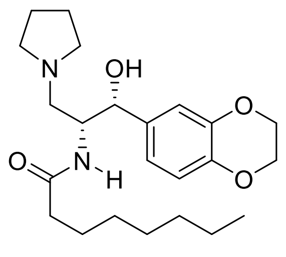

1

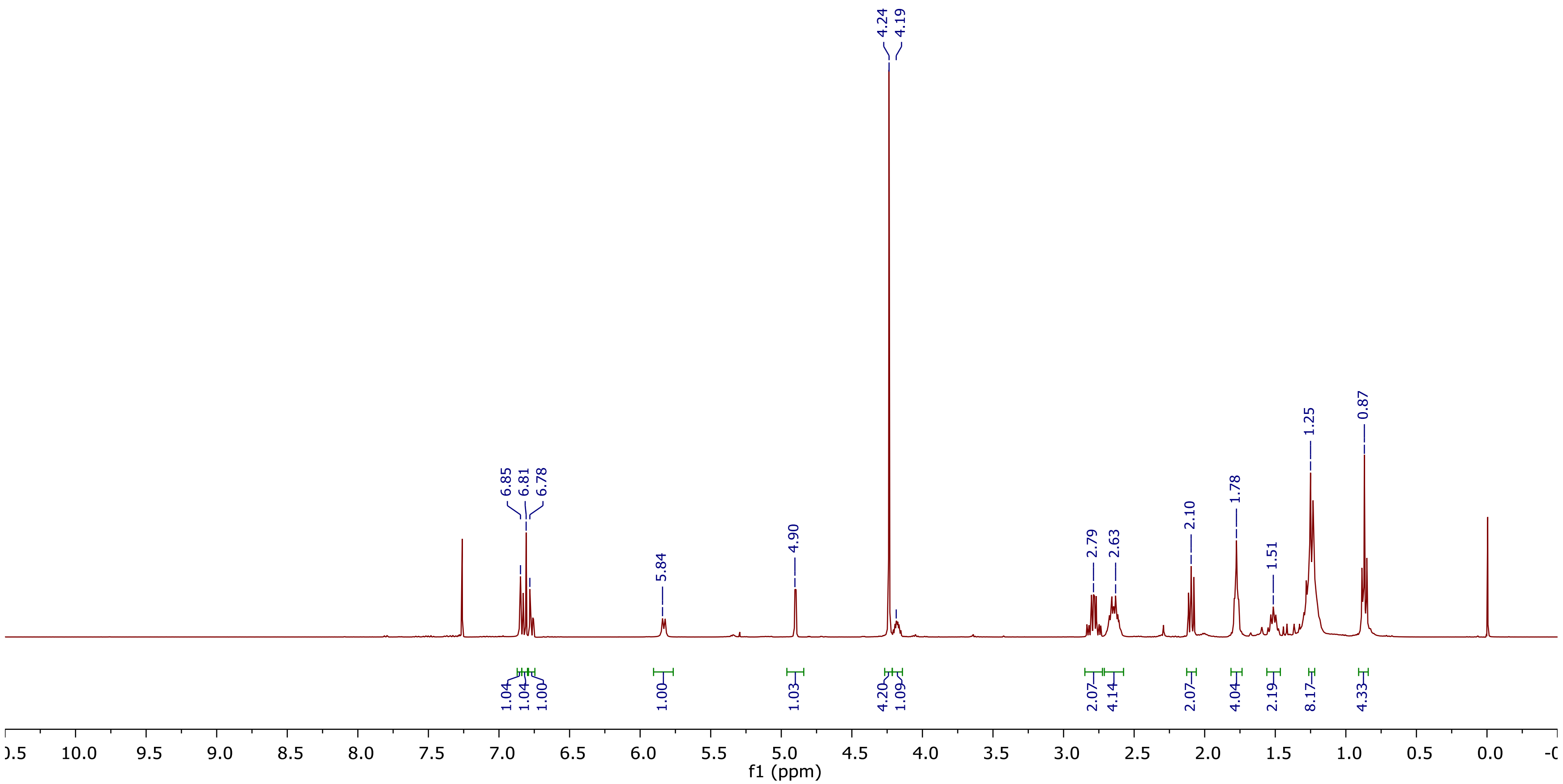

$^{13}C\{^1H\}$ NMR (100.6 MHz, $CDCl_3$)

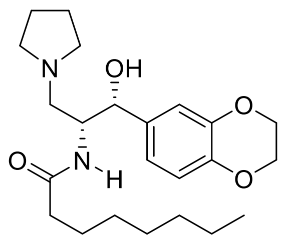

1

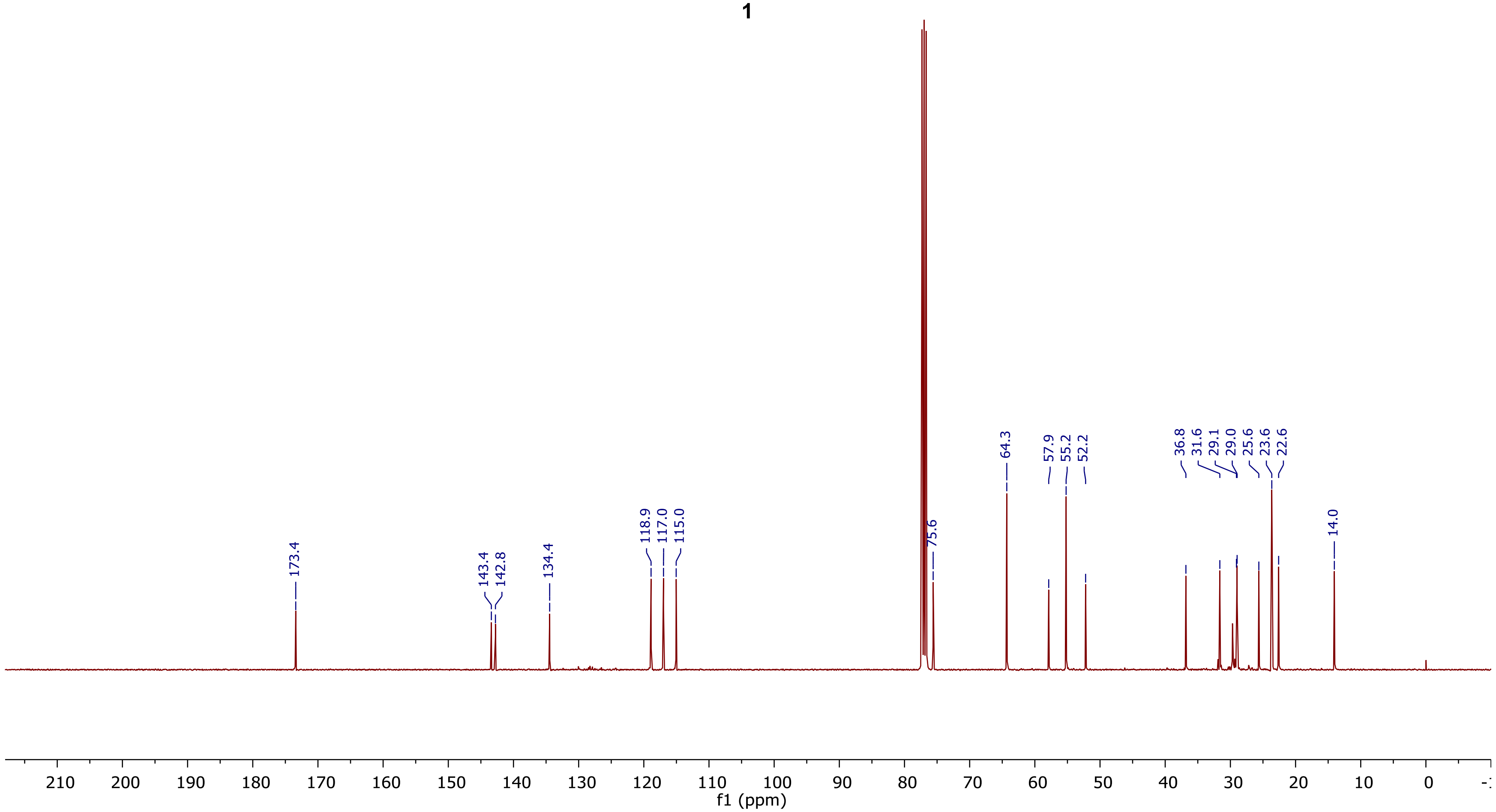

^1H - ^1H COSY NMR (400 MHz, $CDCl_3$)

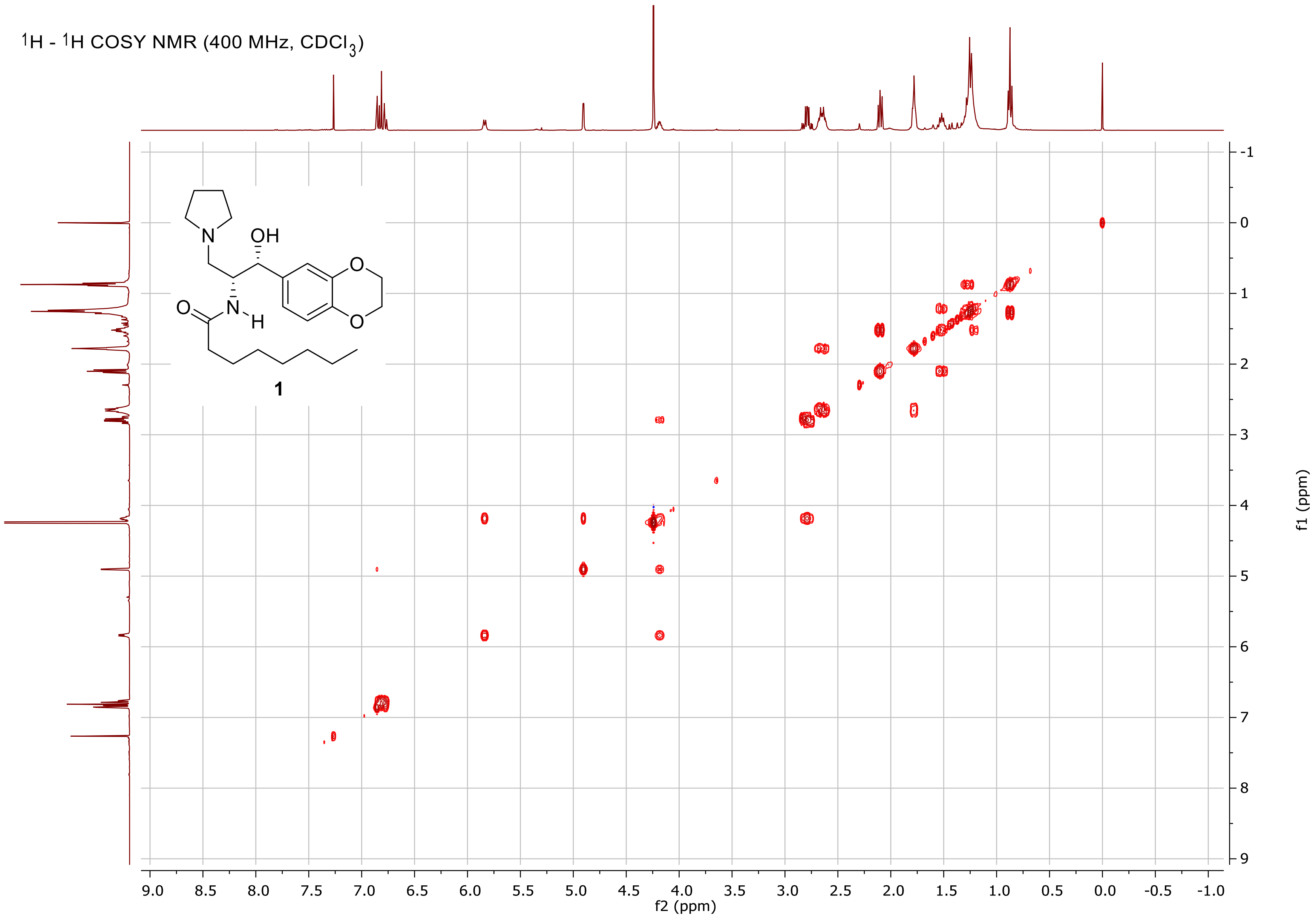

^{1}H - ^{13}C HSQC NMR (400 MHz, $CDCl_3$)

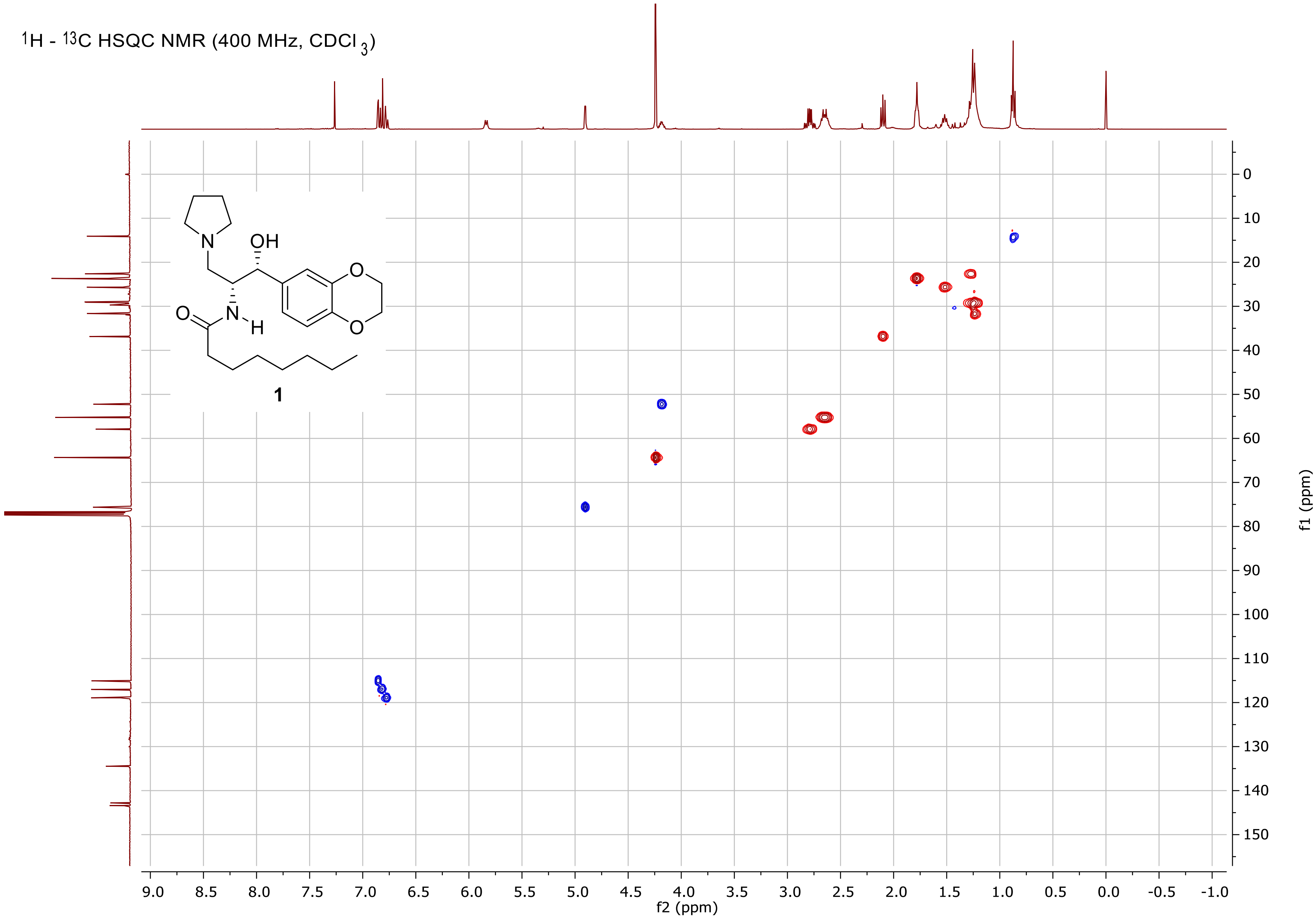

Supplement: Supplementary file 1 [file jo5c03229_si_001.pdf]
